# Abstracts DGAV

**DOI:** 10.1515/iss-2019-2001

**Published:** 2019-03-20

## DGAV: Surgical endoscopy

### Upper GI leakage: last line of defense

(Abstract ID: 60)

M. Kantowski^1^, E. Bellon^1^, T. Rösch^1^

^1^*Universitätsklinikum Hamburg-Eppendorf*

**Background:**

Endoscopic complication management of postoperative upper gastrointestinal leakage with endoluminal vacuum therapy or stent implantation is mostly successful. Presented are various endoscopic strategies to close a leakage after unsuccessful earlier attempts to threat the anastomotic leak by repeated surgical attempts, ineffective endoscopic therapy with standard endoluminal vacuum therapy or stent implantation for longer than 2 months previously.

**Materials and methods:**

Depending on the actual anatomy, presence of a fistula and planed therapeutic aim several endoscopic maneuvers were made to close the defect: leakage combined with cutaneous fistula: combination of modified endoscopic plug implantation and percutaneous vacuum therapy (n=6), giant mediastinal abscess cavity: combination of EVAC therapy using main- and adjactant sponges (n=4), residual mediastinal mini fistulas: EVAC therapy using an open pore folia drainage (n=5), bariatric leakage with cutaneous fistula and need for long time enteral nutrition: fistula closing by JET-PEG and percutaneous vacuum therapy (n=3) or combination of a modified Trelumina feeding tube and long segment sponge (=3), giant jejunal defect: trans corporal Dennis tube with bilateral vacuum suction pump (n=1). Giant gastric defect: mesh plug implantation and vacuum therapy (n=1).

**Results:**

In 22/23 cases it was possible to close the leakage within 1-2 weeks by combination of unusual endoscopic therapies successfully and permanent. Unfortunately in the patient with giant gastric defect occlusion was not reached. She died due to septic complications.

**Conclusion:**

In failure of surgical repair and standard endoscopic therapies of the leakage it was often possible to reach a positive outcome by changing the endoscopic access from endoluminal to percutaneous access with a small diameter scope, using combination of vacuum therapy and other tools (plug, PEG or Trelumina tube) or new materials (open folia drain) to gain a fast and complete occlusion in this difficult cases.

### Complicated wound healing disorder on colostoma – closure with closed negative pressure drainage

(Abstract ID: 174)

J. Müller^1^, T. Schorsch^1^, L. Braun^1^, W. Schulze^1^, C. T . Müller^1^, G. Loske^1^

^1^*Katholisches Marienkrankenhaus Hamburg gGmbH*

**Background:**

Parastomal inflammation with abscess formation of colostomy is - due to the close proximity to the stoma - difficult to treat and associated with long term morbidity. A closed subcutanous negative pressure therapy as an alternative treatment to open surgery is demonstrated.

**Materials and methods:**

Three different types of drainage were used, which differentiate in the open-pore element (oE):on the distal end of a common naso-gastral tube either an open pore PU-Sponge (OPD), a thin, double-layer drainage-film (OFD) or a PU-Sponge covered with a drainage-film (OPFD) was fixed with a suture. These materials differ in diameter and the attachment to the wound base. The therapy was started with the OPD, continued with the OPFD and ended endoscopically with the OFD.

A parastomal abscess formation was surgically opened and rinsed. Then, the open-pore drainage was constructed and the oE placed in the subcutaneous abscess formation. The tube was led out via a small incision distant from the stomy to allow usage of a conventional stoma bag. The wound was surgically closed and a vacuum established with an electronic vacuum pump (-125 mmHg, continuous suction). Regular change of the drainage system was accompanied with endoscopic inspection of the wound cavity to monitor healing. On the first changes of the treatment with large-pore OPD or OPFD (Diameter approximately 15 mm or more) the wound had to be opened again in order to renew the drainage. When the wound was clean and shrunken OFD was set in place. Using the OFD, the drainage was renewed endoscopically without requiring re-opening the wound.

**Results:**

The initial OPD caused a major debridement and reduction of the wound, but changing of the drainage was possible only by re-opening the wound. The OPFD still needed the wound to be re-openend, but due to the sheathed film, the attachment to the wound base was less intense. Finally, the OFD was installed and renewed endoscopically, a further manipulation of the skin was not necessary. This therapy did not affect oral nutrition and supports an unimpaired function of the colostomy.

**Conclusion:**

Closed subcutanous negative pressure therapy is a minimal-invasive alternative in the treatment of parastomal abscess formation.

**Picture:**
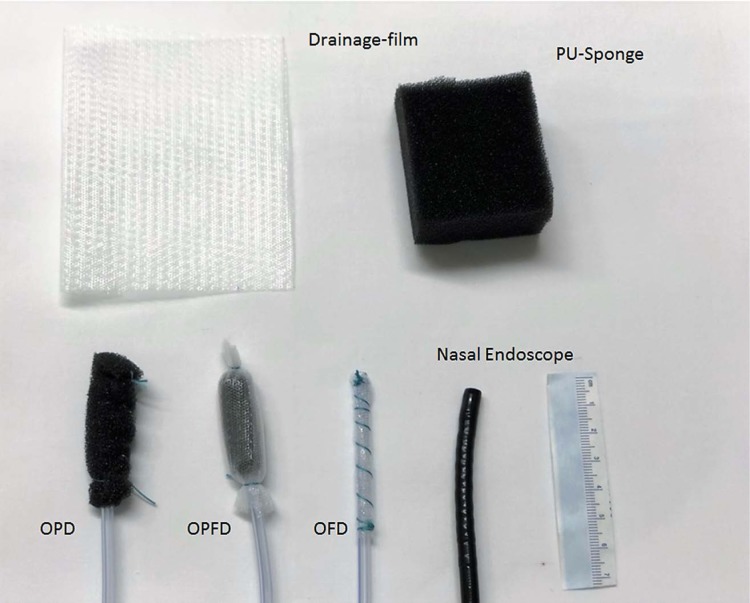
Material used for the new technique of closed negative pressure drainage (OPD: open pore PU-drainage, OPFD: open pore PU-film drainage, OFD: open pore film drainage). The diameter reduces from OPD to OFD. The OFD is set in place with a nasal endoscope.

### Management of esophageal perforation – analysis of 133 patients

(Abstract ID: 288)

A. A. Röth^1^, S.-H. Chon^1^, A. H. Hölscher^1^, T. Herbold^1^

^1^*Uniklinik RWTH Aachen*

**Background:**

Perforation of the esophagus is a life-threatening disease with a very heterogeneous etiology. There is no standardized procedure for the management of an esophageal perforation. More than 50% of all perforations are iatrogenic and although the incidence is rather low in routine endoscopic procedures, it is more and more often due to the increase of endoscopic interventions. On the other hand, owing to the broad field of endoscopic innovations in recent years, an operative treatment is less and less necessary after esophageal perforation.

**Materials and methods:**

In this retrospective study all consecutive patients treated with a perforation of the esophagus between 1998 and 2016 in a high volume center (Department of General, Visceral and Tumor Surgery, University Hospital of Cologne, Cologne, Germany) were analyzed. Patients with postoperative complications such as anastomotic leakage were excluded.

**Results:**

A total of 133 patients were treated due to an esophageal perforation. Iatrogenic injury was the most frequent cause of esophageal perforation. The prevalence of the perforation increased from the proximal to the distal esophagus. 43% of all perforations could be treated endoscopically, 22% healed conservatively. 35% of all patients needed an operative approach. The etiology of the perforation was important for treatment as iatrogenic perforations could be managed endoscopically in most cases whereas Boerhaave syndrome often needed surgical treatment due to the septic constellation. In general, the further the distance between the perforation and the upper esophageal sphincter was, the higher was the likelihood of operative treatment necessity.

**Conclusion:**

The paradigm of a surgical repair of an esophageal perforation changes. Due to innovations, endoscopic treatment gains more and more importance. An early detection, an adequate closure of the defect, a sufficient drainage of mediastinal or pleural retentions and an adjusted antibiotic and antimycotic therapy are the milestones of therapy and important for the outcome. Today, the majority of patients can be treated endoscopically. Therefore, it is necessary to be aware of all different endoscopic treatment possibilities. A close cooperation between an experienced endoscopist and upper GI-surgeon in the setting of a good intensive care unit is essential.

### Efficiency of an integrated workflow with augmented reality in 3D laparoscopic liver resection: Clinical evaluation of a new image guided surgery system

(Abstract ID: 382)

G. A. Prevost^1^, B. Eigl^2^, I. Paolucci^3^, M. Peterhans^2^, S. Weber^3^, G. Beldi^1^, D. Candinas^1^, A. Lachenmayer^1^

^1^*Universitätsspital Bern*

^2^*CAScination AG, Bern*

^3^*ARTORG, Bern*

**Background:**

To investigate technical feasibility and clinical benefit of a new augmented reality system in 3D laparoscopic liver surgery.

**Materials and methods:**

All patients (n=10) who received laparoscopic liver resection by a new image guided surgery system with augmented 3D imaging in a university hospital were included for retrospective analysis. Digitally processed preoperative imaging (magnetic resonance imaging or computed tomography) was merged to the 3D laparoscopic image using a landmark-based registration technique. Intraoperative efficiency of the procedure was measured as time needed to achieve sufficient registration accuracy. Technical accuracy was reported as fiducial registration error (FRE). Clinical benefit was assessed trough a questionnaire which was completed by the primary surgeon after each operation. Resection quality and 30 day postoperative morbidity are reported as outcome parameters.

**Results:**

From January to March 2018, ten 3D laparoscopic liver resections of a total of 18 lesions were performed using the novel augmented reality system. The mean FRE of the last registration attempt was 9.2 mm (SD 2,8). Median time for registration was 8:50 min (range 1:31 - 23:56). Average operation time was 136 minutes (SD 43). The questionnaire revealed the ease of use of the system and the benefit for resection of vanishing lesions as convincing positive aspects, whereas image registration accuracy for resection guidance was consistently judged as too inaccurate. Histology reported complete resection in all retrieved lesions. No major complications (Clavien-Dindo >= IIIb) occurred during the 30 days follow-up, although 3 patients required interventional bilioma drainage post-operatively.

**Conclusion:**

Augmented reality in 3D laparoscopic liver surgery with landmark-based registration technique is feasible with only little impact on the intraoperative workflow. The benefit for detecting particularly vanishing lesions is high. For an additional benefit during the resection process, registration accuracy has to be improved and non-rigid registration algorithms will be required to address intraoperative anatomical deformation.

### Profitability of Endoscopic Vacuum Therapy (EVT) compared to alternative therapy methods in patients with anastomotic leakage after esophagectomy

(Abstract ID: 490)

A.-K. Eichelmann^1^, S. Ismail^1^, D. Palmes^1^, A. Pascher^1^, M. G. Laukötter^1^

^1^*Universitätsklinikum Münster*

**Background:**

In the management of anastomotic leaks following esophageal resection, endoscopic vacuum therapy (EVT) has been successfully established in recent years with promising results. Our previous data indicated that EVT is more effective than endoscopic stent placement in terms of postoperative complication management. However, EVT might results in higher therapy costs due to a prolonged hospital stay in combination with a more intensive course of therapy and higher material costs and personnel expenses in contrast to stent placement or revision surgery. So far, no studies are available concerning financial aspects of endoscopic complication management following esophageal resections.

**Materials and methods:**

This retrospective study included all patients suffering from anastomotic dehiscence following esophageal resection due to malignant lesions at our institution between 2009 and 2015. Depending on the management of the anastomotic leak, patients were divided into three subgroups: EVT, stent or re-operation. EVT was introduced into our clinical routine in 2012 and has replaced stent placement as former gold standard therapy. Patients who were managed by two different treatment modalities (e.g. EVT and stent therapy) were excluded. Detailed data about the incurred costs of the treatment during hospital stay were collected. The treatment expenses were characterized by material and personnel costs, parenteral / enteral nutrition, intensive care, length of stay, imaging and costs for re-operation.

**Results:**

During the study period, 68 patients with anastomotic leakage were treated. 18 patients were excluded due to a switch of the treatment modality, resulting in following sub-groups: EVT; n=29 (mean age 61 years), stent; n=14 (68 years) and re-operation; n=7 (73 years, n=2 oversewing of the circular staple line, n=1 re-anastomosis, n=4 discontinuity resection). In the EVT group, the mean duration of therapy was 32 days, sponges were changed 7 times (mean) with 5 endoscopic procedures performed under general anaesthesia. In the stent group, the mean duration of therapy was 45 days, the stent was changed 1.5 times (mean) with 0.8 endoscopic procedures performed under general anaesthesia. The average costs for the endoscopic management were comparable (EVT: 1326€/patient, stent: 1035€/patient (p=0.7)), while costs for re-operation were significantly higher (5811€, p<0.0001). With respect to additional costs, we did not observe a difference in costs for intensive care or imaging; however, expenses for parenteral / enteral nutrition were lower in the stent group (p=0.0046).

**Conclusion:**

EVT is a safe, effective, successful, but labour-intensive therapy. However, except for nutrition, expenses for EVT are comparable with those for stent therapy, most likely due to the high costs for the stent itself. The majority of costs that occurred in the complication management of leaks after esophageal resection are attributable to the ICU length of stay. From the economic point of view and if medically justifiable, complication management strategies should be accompanied by a reduced ICU length of stay.

### Endoscopic closure of bariatric and upper gi leaks with apollo overstich ^®^

(Abstract ID: 800)

R. Zorron^1^, S. Schmidt^1^, C. Grande^1^, A.-L. Rütten^1^, H.-U. Horn^1^, F. Marusch^1^, M. Specht^1^

^1^*Klinikum Ernst von Bergmann Potsdam, Berlin*

**Background:**

Akute leaks and chronic gastrocutaneous fistulas are a rare clinical condition usually addressed by open or laparoscopic surgery. An innovative endoscopic suture system (Apollo Overstich^®^) allows full-thickness running sutures of the gastric wall. We demonstrate a case series of of successful closures gastric leaks and fistulas.

**Materials and methods:**

Patients with acute leaks and chronic gastrocutaneous fistulasare prsented. The choice for endoscopic therapy was based in clinical stability and previous laparoscopic or CT drainage. The novel endoscopic suture device was applied and connected to a double channel endoscope. The sutures (polypropylene 2-0) were placed endoscopically through a 2-channel gastroscope under direct vision. Stent therapy was not indicated in the series.

**Results:**

After application of an endoscopic sutures with the Apollo Overstich^®^ system the gastric wall defect was permanently sealed in 60 % of the patients. The remaining patients were treated with endoscopic Vacuum therapy and one patient submitted to surgery.

**Conclusion:**

The concept of endoscopic full-thickness sutures was successfully applied in acute leaks and chronic gastrocutaneous fistula. This novel technique needs further evaluation and may reduce the rates of surgical interventions in this complex disease.

### Endoscopic sealing of enterocutaneus fistulas with a new combination of VAC-Plug

(Abstract ID: 841)

M. Kantowski^1^, E. Bellon^1^, T. Rösch^1^

^1^*Universitätsklinikum Hamburg-Eppendorf*

**Background:**

Introduction: A new method to close postoperative intestinal fistulas is presented: Five patients were threatened with high output fistulas of the small intestine (two with gastro-pleurocutaneous fistulas after gastric pull up operation and three duodeno-/jejuno-cutaneous fistulas after multivisceral resection. Earlier attempts to close the fistulas by open surgery or endoscopic approach (endoluminal vacuum therapy or stent etc.) were without success. The opening of the enterocutaneous fistulas were approximately 5x5 mm in each case. Improvement of wound care and definitive closure of fistulas were our target.

**Materials and methods:**

Methods: First an endoscopic evaluation of the fistulas from intestinal and percutaneous approach was performed with the standard gastroscope and then with the fine caliber endoscope. Since established methods (surgical revision, long-term local drainage, stent or endoluminal endoscopic VAC therapy) could not achieve fistula occlusion, we decided on a combination of plug implantation and percutaneous vacuum therapy. A conventional anal fistuar plug (COOK) was modified: The inner part of the plug cone was made waterproofed with bone wax in order to achieve a longer durability of the plug compared to the secretions of the upper gastrointestinal tract, which always proved to be disappointing during other implantations in the upper GIT. The plug was placed in the fistula analogous to a PEG via a thread passage. A small drainage tube (10 chr) covered with a special folia (Suprasorb-CNP, Lohmann & Rauscher) connected with a VAC pump was placed in the fistula channel just behind the plug by percutaneous approach by a guide wire placed under endoscopic control from outside to capture residual secretion next to the plug.

**Results:**

Result: In all five patients the enterocutaneous fistula, which was refractory to therapy, was completely suspended within 1-2 weeks after VAC plug implantation. No patient developed a fistula relapse during the course of the disease for more than 1,5 years.

**Conclusion:**

Conclusion: The combination of a modified plug for fistula occlusion with percutaneous VAC drainage behind it led in all five cases to a complete healing of the high output small intestine fistulae, which were refractory to all earlier therapy attempts in apron.

### Case Report: EFTR (endoscopic full thickness resection) in synchronous colon and rectal carcinoma can reduce surgical trauma

(Abstract ID: 867)

K. Liedtke^1^, A. Schreiber^1^, C.-D. Heidecke^1^, R. Flieger^1^, A. Glitsch^1^

^1^*Universitätsmedizin Greifswald*

**Background:**

We report on a 63-year-old patient who underwent external colonoscopy. Two endoscopically non-ablatable tumors with IEN in the colon (ileocecal valve, right flexure) and a malignancy-suspicious, large polyp in the rectum were described.

**Results:**

In our interdisciplinary tumor conference, we made a case by case decision for a two-stage approach with primary EFTR of the rectal lesion. Due to the size, endoscopic removal took place first by sling followed by the basement removal using FTRD uncomplicated.Histologyrevealed a rectal carcinoma with tumor formula: pT1(sm3) G1 L0 V0 pR0. Subsequently, laparoscopic-assisted hemicolectomy in endoscopically non-removable polyps of unclear dignity was indicated. Intraoperatively, conversion was necessary and extended hemicolectomy was performed with removal of 20cm ileum and 45cm colon. Here, liver showed a highly metastatic suspicious mass that was biopsied. Furthermore, operation was uncomplicated and post-operative stay not prolonged. Histology revealed a synchronous, hepatic metastatic colon carcinoma pT3 L0 V0 pN0(0/29) cM1(HEP) R0 G2 k-ras-/-n-ras-/-. CT showed a multilocular metastatic finding in the liver without any surgical option. Chemotherapy had to be stopped due to sepsis and significant worsening of the condition. With rapid tumor progression, the patient died about six months after surgery.

**Conclusion:**

In this case a minimization of surgical radicality with local oncological R0 situation could be achieved by applying FTRD.

## DGAV: Endocrine surgery

### Response to peptide receptor radionuclide therapy (PRRT) in patients with metastasized neuroendocrine neoplasms (NEN) with and without resection of the primary tumor

(Abstract ID: 14)

D. Kaemmerer^1^

^1^*Zentralklinik Bad Berka*

**Background:**

Peptide receptor radionuclide therapy (PRRT) is a highly effective therapeutic option to treat advanced neuroendocrine neoplasms (NEN). However, it is still unclear whether resection of primary tumors improves overall survival after peptide receptor radionuclide therapy.

Aim(s): To find out whether resection of primary tumors prior to PRRT will result in better overall survival than without.

**Materials and methods:**

Retrospectivly, we analyzed the data of 889 patients with advanced NEN (G1-G3) treated with PRRT at least one cycle. In 403/889 patients (45%, group 1) primary tumors were removed before PRRT. Group 2 enfolded 486 patients (55%) with no resection previously. Overall and progression free survival was determined by 68Ga SSTR-PET/CT and EORTC response criteria in all patients.

**Results:**

Most patients had their primary tumor in pancreas (n = 335; 38%) and small intestine (n = 284; 32%), mostly well differentiated (G1: n = 123; 24%; G2: n = 348; 39%; G3: n = 56; 6%). Most patients received a combined treatment with 90Yttrium and 177Lutetium cycles (n = 486; 53%), only 177Lutetium (n = 321; 36%) and only 90Yttrium (n = 100; 11%), exclusively. Both groups received a mean of 4 cycles of PRRT with a mean of 21.9 GBq cumulative radioactivity. Median overall survival in group 1: was 134.0 months (CI: 119.7 -148.3), whereas overall survival in group 2: was 67.0 months (CI: 57.3 - 76.7; HR 2.27); p < 0.001. Likewise, median progression free survival was extended after first PRRT in group 1: 18.0 (CI: 16.0 - 20.0) months versus group 2: 13.0 (CI: 11.4 - 14.6; HR 1.55) months p< 0.001. The resected patients performed better with a significant longer median overall survival related to the primary tumor origin (pancreas, small intestine, colo-rectal) and the grading (G1-G3) than the nonresected patients in subgroup analysis.

**Conclusion:**

After surgery of primary tumors, metastasized patients have a better overall and progression free survival after PRRT. These effects are strong indicators for clinical practice that primaries should be removed when feasible before PRRT.

### Percutaneous CT-guided microwave ablation for neuroendocrine liver metastasis: A successful way to treat patients?

(Abstract ID: 65)

C. Kim-Fuchs^1^, A. Lachenmayer^1^, M. Maurer^1^, G. Beldi^1^, D. Candinas^1^

^1^*Universitätsklinik für Viszerale Chirurgie und Medizin, Bern*

**Background:**

Percutaneous CT-guided microwave ablation (MWA) is a good treatment option for hepatocellular carcinoma . This technique can be used to safely treat several metastasis simultaneously without losing a lot of healthy liver tissue. In this study we addressed the question how successful percutaneous CT-guided MWA is for liver metastasis from neuroendocrine tumours (NET) in terms of local recurrences or reduction of symptoms if used in a palliative setting.

**Materials and methods:**

Retrospective analysis of a prospective database. The indication for ablation was discussed at the multidisciplinary endocrine tumour board. Ablations were either done in a curative setting or as a means of reducing tumour load to treat symptomatic patients.

**Results:**

Between 01/2015 and 06/2018, 380 different tumours were ablated percutaneously in 203 patients. Of these, 18 (4.7%) ablations were done in seven patients (three women) with NET liver metastasis in a total of nine sessions. Median age was 69 years (54-75 years). Three primary tumours were in the pancreas, four in the small bowel and one of unknown primary. All known primary tumours had been previously resected. Six patients received one session. Per session 1-4 tumours were ablated. One patient received 3 sessions over time. One patient received a palliative ablation to reduce tumour load and the associated symptoms. The other patients were treated with a curative intent.

There were no peri-, and postinterventional complications. All patients left the hospital after one day. The first follow up with an MRI was done three months after the intervention, with an overall median follow up of 18.9 months (4.2-33.1).There was no local recurrence in patients treated with curative intent. One out of six patients developed new metastases in the liver and received two further sessions (five lesions) and is now tumour free. The patient with palliative ablation had a significant reduction of symptoms in the follow up.

**Conclusion:**

Percutaneous CT-guided MWA for patients with NET liver metastasis is a safe treatment option in experienced hands. Ablations can be used to treat metastasis in a curative setting or to reduce tumour load and symptoms in patients not amenable to definitive surgery. Especially patients with difficult to reach intrahepatic lesions (close to the hilum, hepatic vein confluens, segment I) profit for the guided ablation.

### The pathophysiology of cervical hemorrhage after thyroid surgery – a cervical compartment syndrome?

(Abstract ID: 118)

U. Wirth^1^, M. Bonleitner^2^, T. von Ahnen^2^, J. Schardey^3^, S. Schopf^2^, H. M. Schardey^2^

^1^*Uniklinik München*

^2^*Krankenhaus Agatharied, Hausham*

^3^*Universitätsklinikum Freiburg*

**Background:**

Bleeding complications are rare, but potentially life-threatening events in thyroid surgery (ca. 1,2 - 1,8%). Theories about tracheal collapse dominated the literature over decades, but they were only based on case reports and small case series available in literature. We were able to collect some basic experimental data in our previous animal experiments about the underlying pathophysiology, especially the connection between cervical hemorrhage and mortality for respiratory failure and hypoxemia.

Our aim now was to investigate the clinical course and underlying pathophysiology of cervical hemorrhage in an animal model to derive clinical and therapeutical implications.

**Materials and methods:**

We induced 14 simulated and 6 spontaneous cervical hemorrhages in seven pigs with subsequent elevation of the cervical compartment pressure. The animals were under light general anesthesia with intact respiratory drive and secured airways. Vital signs, pressure levels in the cervical compartment, aorta, inferior vena cava, internal jugular vein, the cerebral oxygenation (INVOSTM) and the intracranial pressure were measured and analyzed.

**Results:**

Over all experiments, vital signs (arterial blood pressure, heart rate, peripheral oxygenation, intracranial pressure and CVP) were stable. In case of spontaneous cervical hemorrhage, cervical compartment pressures near the levels of the mean arterial blood pressure were detected (59mmHg). There was a direct correlation between the increase of the cervical compartment pressure and of the pressure elevation in the internal jugular vein in all experiments. In addition, there was a significant decrease of cerebral oxygenation following cervical compartment pressure elevation, which was independent of the systemic arterial oxygenation. In n = 9/14 (64%) of the experiments with simulated hemorrhage, we could induce respiratory failure due to the increase of cervical compartment pressure (threshold pressure 54mmHg). In these experiments, a significant decrease in cerebral oxygenation appeared about 120 seconds before respiratory failure.

**Conclusion:**

In case of spontaneous cervical hemorrhage pressure levels in the cervical compartment nearly reached the mean arterial blood pressure. There is a direct correlation between increase of cervical compartment pressure, increase of pressure in the internal jugular vein and decrease of cerebral oxygenation. Our investigations indicate an impairment of cerebral perfusion as a cause for respiratory failure based on the elevated cervical compartment pressure in cervical hemorrhage similar to other compartment syndromes.

### Major hepatic resection following radioembolization for neuroendocrine liver metastases is safe and associated with a prolonged hepatic tumor clearance

(Abstract ID: 168)

F. Bösch^1^, M. Thomas^1^, H. Ilhan^1^, T. Knösel^2^, V. Pfahler^1^, V. Eibl^1^, P. Bartenstein^1^, C. Auernhammer^1^, C. Spitzweg^1^, M. Guba^1^, J. Werner^1^, M. K. Angele^1^

^1^*Uniklinik München*

^2^*Klinikum der LMU München*

**Background:**

Radioembolization is well established in the treatment of neuroendocrine liver metastases. However surgery is rarely performed after radioembolization, although liver resection is the gold standard in the treatment of localized neuroendocrine liver metastases. Therefore, aim of the present study was to evaluate the safety and feasibility of liver resection after radioembolization in a homogenous cohort.

**Materials and methods:**

From a prospective surgical (n=494) and nuclear medical (n=138) database patients with NELM who underwent liver resection and/or RE were evaluated. Between September 2011 and December 2017 eight patients could be identified who underwent liver resection after radioembolization (mean dose of 1746 Mbq). Overall and progression free survival were evaluated as well as epidemiological and perioperative factors. The surgical specimens were analyzed for necrosis, fibrosis, inflammation, and steatosis.

**Results:**

The mean hepatic tumor load of patients, who had liver surgery after radioembolization, was 31.4% with a mean Ki-67 proliferation index of 5.9%. The majority of these patients (7/8) received whole liver radioembolization prior to liver resection, which did not increase morbidity and mortality compared to a surgical collective. Indications for radioembolization were oncological (6/8) or carcinoid syndrome associated reasons (2/8). Mean overall survival was 25.1 months after radioembolization and subsequent surgery. Tumor necrosis in radioembolized lesions was 29.4% without evidence of fibrosis and inflammation in hepatic tissue.

**Conclusion:**

This is the first study analyzing the multimodal therapeutic approach of liver resection following whole liver radioembolization. This treatment algorithm is safe, does not lead to an increased morbidity and is associated with a favorable oncological outcome. Nonetheless, patient selection remains a key issue.

### Total versus near-total thyroidectomy in Graves’ disease – Results of the randomized controlled multicenter TONIG-trial

(Abstract ID: 203)

E. Maurer^1^, S. Wächter^1^, K. Holzer^1^, D. K. Bartsch^1^

^1^*Universitätsklinikum Marburg*

**Background:**

Some data indicate that the incidence of hypoparathyroidism after surgery of Graves’ disease is significantly lower after near-total thyroidectomy (NTT) compared to total thyroidectomy (TT). Therefore, the study evaluated the incidence of hypoparathyroidism in Graves’ disease after NTT compared to TT.

**Materials and methods:**

Patients with Graves’ disease scheduled for surgery according to the national S2-guideline were prospectively randomized to TT or NTT with a defined total remnant of less than 1g on each side. The incidence of transient hypoparathyroidism within 6 weeks after surgery was defined as primary endpoint. Secondary endpoints were permanent hypoparathyroidism, recurrent laryngeal nerve palsy, reoperations due to bleeding, recurrent disease, changes of endocrine orbitopathy (EO) and quality of life six weeks after surgery.

**Results:**

Eighteen participating centers randomized 205 patients to either TT (n=102) or NTT (n=103) between 09/2015 and 01/2017. It is of note that in 7 of 103 (7%) patients in the NTT group resp. 1 of 102 (1%) patients in the TT group did not receive the randomly allocated intervention. According to intention to treat postoperative transient hypoparathyroidism occurred in 20 (19%) patients after NTT and in 21 (21%) patients after TT (p = 0.84). The risk of transient hypoparathyroidism for patients older than 45 years was clearly lower. The hypoparathyroidism was permanent after 6 months in 2% (2 of 82) in the NTT and 4% (3 of 76) in the TT group (p = 0.59). The rates of parathyroid autotransplantation were similar in both groups (NTT 24% vs. TT 28%, p = 0.50). The rate of transient recurrent laryngeal nerve palsy with regard to the nerves at risk was similar in both groups (NTT 3.5% vs. TT 2,5%, p = 0.54) as well as the rate of reoperations for postoperative bleeding (3% vs. 0%, p = 0.07). Graves’ disease recurred in only one patient who underwent TT. An existing EO (n = 49) improved in 35.7% (10 of 28) patients in the NTT and 14.3% (3 of 21) patients in the TT group. The median SF-36 physical and mental health summary scores 6 weeks after surgery were 52 and 47 in the NTT and 51 and 52 in the TT groups (p = 0.73 and p = 0.96).

**Conclusion:**

NTT for Graves’s disease is not superior to TT with regard to postoperative hypoparathyroidism, recurrent nerve palsy, bleeding and recurrence of disease, but technically sometimes more demanding. TT should be the standard resection for Graves’ disease.

### Pilotstudy for standardization of the lymphadenectomy in patients with adrenocortical cancer

(Abstract ID: 251)

G. M. Franke^1^, W. Nimphius^2^, D. K. Bartsch^2^, J. Waldmann^2^

^1^*Universitätsklinikum Schleswig-Holstein, Kiel*

^2^*Universitätsklinikum Gießen und Marburg - Standort Marburg*

**Background:**

There is an ongoing debate on the impact of lymphadenectomy in patients with adrenocortical cancer (ACC). A german register study recently has revealed that a removal of more than 5 lymphnodes in patients with ACC is associated with a decreased risk for local recurrence and a trend towards an improved survival. To categorize the lymphatic drainage of the adrenal glands respecting anatomical and surgical principles and to establish the median number of lymphnodes which is required to define a systematic lymphadenectomy.

**Materials and methods:**

The lymphatic drainage of the adrenal gland region was divided into 8 compartments following anatomical and surgical principles. Twenty retroperitoneal sections from 20 body donors, consequently 160 lymphatic individual preparations were harvested systematically. Exclusion criteria were medical history of retroperitoneal, renal or adrenal surgery and lymphoma. The preparations were histologically assessed with HE sections for lymphnodes. Statistical analysis was performed using SPSS 20.0. All patients underwent surgery for ACC at the University Hospital of Giessen and Marburg, campus Marburg were retrospectively analysed regarding the number and distribution of lymphnodes resected applying the 8 compartments.

**Results:**

Lymphatic compartments were defined as I (interaortocaval superior), II (interaortocaval inferior), III (precaval and lateral superior right), IV (precaval and lateral inferior right), V (adrenal gland and retroperitoneal right), VI (paraaortal and lateral superior left), VII (paraaortal and lateral inferior left), VIII (adrenal gland and retroperitoneal left). A median number of 20 (range 8-34) lymphnodes were dissected, which were distributed into the 8 compartments: I (median 1, range 0-5), II (median 4, range 0-20), III (median 0, range 0-3), IV (median 3, range 0-6), V (median 0, range 0), VI (median 2, range 0-9), VII (median 8, range 0-17), VIII (median 0, range 0). The analysis for both sides seperately showed 8 LN (0-31) for the right and 15 (0-51) for the left side and a median of 5 LN (range 0-20) for the interaortocaval compartment. The compartment which includes the adrenal gland bared any lymphnodes. The vast majority of LN were localized to the compartments II, IV, VI and VII.

Lymphnodes were reported in 14/36 (39%) patients with ACC who underwent surgery. The median number of lymphnodes was 2 (range 0-21). Lymphnodes metastases were found in 3/14 (21,4%) patients when lymphnodes were resected. Analog to the results from the body domors no LN was found in compartments V and VIII. In 2/14 patients LN could be localized to compartment II, in 6/14 to compartment VI and 5/14 to compartment VII. A systematic lymphadenectomy was performed in 1 patient with a total number of 21 LN and 14 LNM.

**Conclusion:**

The LN-distribution of the adrenal region into the 8 proposed compartments were similiar in the present patient cohort and in body donors. A systematic lymphadenectomy in patients with acc should include compartments II, IV, VI and VII and a minimum number of 9 LN. A standardized discription of the lymphadenectomy should be part of the surgical report. The impact on recurrence and overall-survival have to be evaluated in a prospective study.

**Picture:**
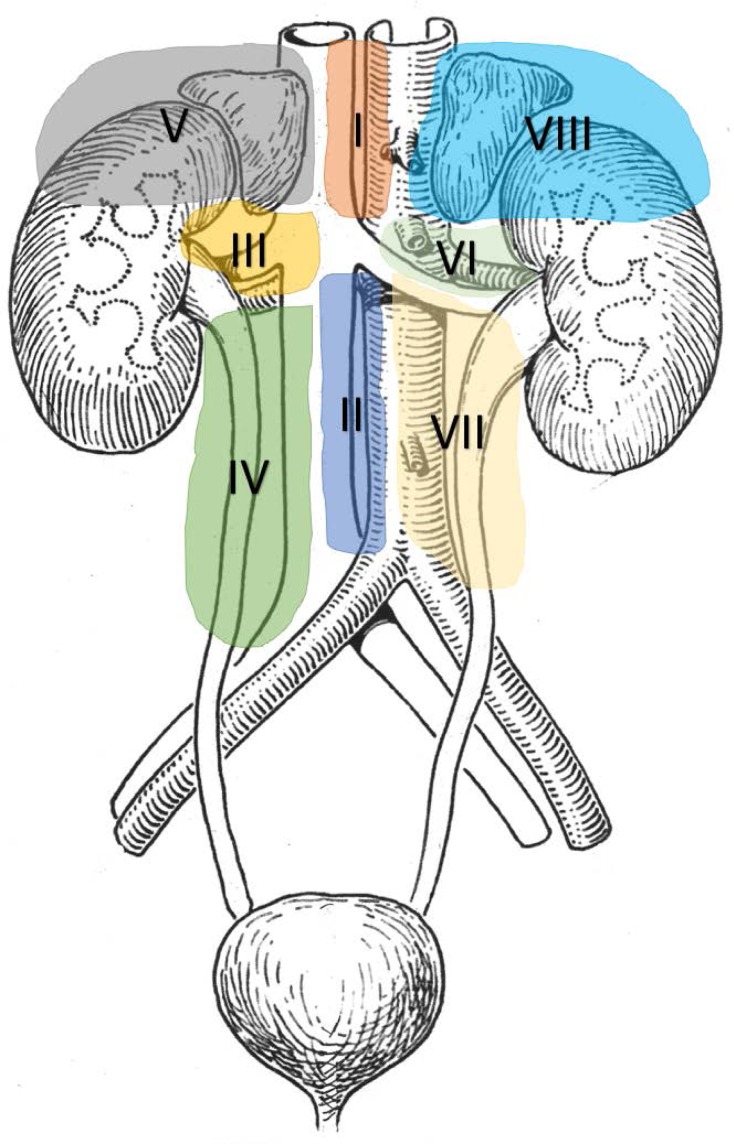
Lymphatic drainage of the adrenal gland

### Transoral endoscopic thyroid surgery via vestibular approach – initial experience and of proposal of a multicentric trial (EVA Trial)

(Abstract ID: 266)

S. Müller^1^, M. Senne^1^, E. Karakas^2^, R. Zorron^3^

^1^*Universitätsklinikum Tübingen*

^2^*Krankenhaus Maria Hilf, Alexianer GmbH, Krefeld*

^3^*Klinikum Ernst von Bergmann, Potsdam*

**Background:**

Transoral endoscopic thyroid surgery via vestibular approach (TOETVA) is a new minimal invasive and scarless access to the thyroid gland. Aim of this study is to evaluate its initial results and safety issues.

**Materials and methods:**

Prospective analysis of all consecutive patients undergoing TOETVA at a tertiary center. Procedure times, complication, early postoperative pain and postoperative swallowing problems using a validated score were assessed.

**Results:**

Up to date 79 patients were evaluated for eligibility (65 did not meet the inclusion criteria, three patients declined to participate). A total of 11 female patients underwent TOETVA (8 hemithyroidectomies and three total thyroidectomies). Median operative time was 140 minutes [93-201]. Mean thyroid nodule diameter and mean thyroid specimen weight were 2.4cm±1.3 and 20.6 g ± 10.6 respectively. No conversion to conventional thyroid surgery was necessary and no postoperative complication occurred with 30 days. Mean pain scores (VAS 0-10) on postoperative day one and two were 1.3 ± 1 and 0.8 ± 0.7 respectively. Mean postoperative swallowing scores according to Lombardi (0-24) at day 2, day 14 and day 30 were 4.2 ± 3.8, 0.7 ± 1.5 and 0 respectively. Median hospital stay was 2 days [1-2].

**Conclusion:**

TOETVA seems to be feasible in carefully selected patients. Especially a potential benefit on early postoperative pain and postoperative swallowing problems needs to be addressed closer in a proposed multicentric prospective trial (evaluation of vestibular approach thyroid surgery - EVA Trial).

### Influence of the BRAF V600E mutation on the papillary thyroid carcinoma

(Abstract ID: 306)

J. Grone^1^, N. Tabriz^1^, V. Uslar^1^, R.-P. Henke^2^, D. Weyhe^1^

^1^*Universitätsmedizin Oldenburg, Pius-Hospital, Oldenburg*

^2^*Pathologie Oldenburg*

**Background:**

The BRAFV600E mutation (BRAF+) is the most common genetic cause of papillary thyroid carcinoma (PTC) and is considered a specific diagnostic marker. Studies suggest that the mutation status is associated with aggressive tumor characteristics such as extrathyroid extension and lymph node metastasis, thereby increasing the risk of persistent and recurrent progressions. It is therefore being discussed whether a more extensive surgical strategy should be pursued in the case of preoperative mutation detection in fine needle puncture. However, the importance of molecular diagnostics remains a controversial issue.

**Materials and methods:**

A retrospective study for the analysis of histological tissue in PTC from 2007-2016 was conducted, which investigated the extent to which BRAF+ in PTC was present in our own patient's population and the influence of positive mutation detection on various outcome parameters. N=270 patients from the database of the University Clinic for Visceral Surgery at Pius Hospital Oldenburg met the inclusion criteria. The consent for the evaluation of the resected tissue stored in the pathology Oldenburg was granted by n=198 Pat. The data was analyzed with regard to diagnostic procedures, pathological characteristics, therapeutic intervention and postoperative complications.

**Results:**

n=186 (m=46; w=140) tissue samples were successfully pyrosequenced with n=98 BRAF+ and n=88 BRAF-. There is no significant difference between BRAF+ and BRAF- in gender distribution, body mass index, thyroid scintigraphy, preoperative TSH, surgical therapy, radioiodine therapy, lymph node metastasis, distant metastasis, complicated courses and recurrence. Significant differences (BRAF+ vs. BRAF-) exist in age at the time of primary treatment (49.5yrs vs. 43.9yrs, p=0.011), malignancy detection in fine needle puncture (classified as Bethesda 6: 18.9% vs. 1.8%, p=0.040), tumor size (14.4mm vs. 18.3mm, p=0.018), multifocal growth (30.6% vs. 17.0%, p=0.031) and extrathyroid extension (22.4% vs. 10.2%, p=0.026; Fig 1). In a multivariate analysis, a model with lymph node metastasis with regards to number and localization, and BRAF mutation status best describes recurrence development (p<0.001), but the BRAF mutation status alone is not significant in this model.

**Conclusion:**

BRAF+ incidence in our collective is comparable to literature. The results suggest a connection of the BRAF mutation with more aggressive tumor characteristics, as evidenced for instance by extrathyroid extension and multifocal growth and simultaneously smaller tumor size, but a sole influence of BRAF+ on recurrence rate could not be proven. However, if a BRAF+ patient is treated surgically, one should take into account the seemingly more aggressive tumor behavior.

**Picture:**
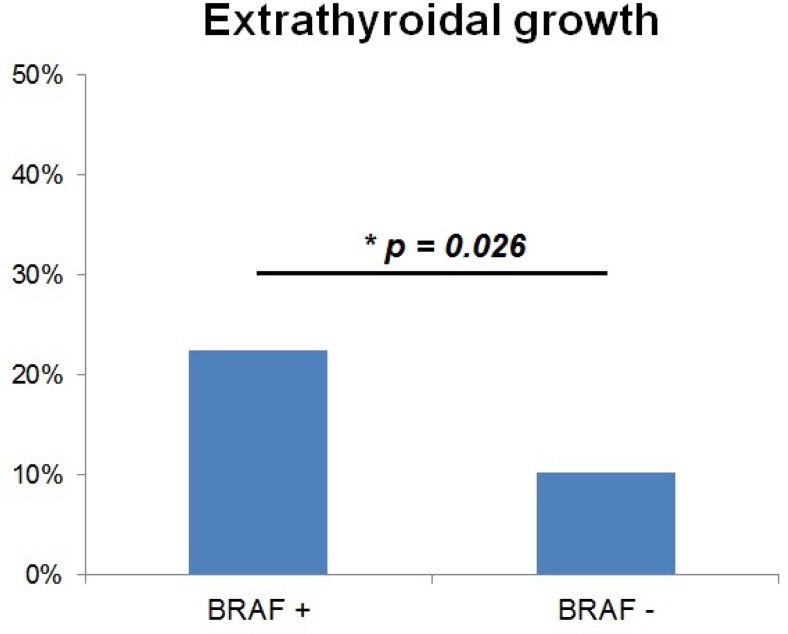
Extrathyroidal growth with regards to BRAF mutation status

### Outcomes of parathyroid carcinoma: Does radical resection as primary treatment improve disease-free and overall survival?

(Abstract ID: 616)

S. Wächter^1^, K. Holzer^1^, J. Manoharan^1^, D. K. Bartsch^1^, E. Maurer^1^

^1^*Universitätsklinikum Marburg*

**Background:**

Radical en-bloc resection of the tumor is generally recommended for parathyroid carcinoma (PC) as primary treatment strategy. However, it remains unclear, whether the removal of the ipsilateral thyroid lobe and central lymph nodes lead to a survival benefit. This study analysed the survival of PC after surgical treatment.

**Materials and methods:**

We retrospectively analysed patients with PC treated in our hospital regarding clinicopathological features, surgical treatments, disease-free and overall survival.

**Results:**

From 1977 to 2018 19 patients with PC were operated. At diagnosis 1 patient had distant metastases and 18 patients showed a local disease. Ten patients underwent initial en-bloc resection with hemithyroidectomy and ipsilateral lymphadenectomy and the other 9 patients received a parathyroidectomy or palliative resection. All patients with parathyroidectomy developed a recurrence (9/9) compared to only 2 of 10 with initial en-bloc resection. The 2 patients with en-bloc resection required 1 and 2 reoperations for recurrent disease compared to up to 16 reoperations/-interventions in the 9 patients with initial parathyroidectomy. After a median follow-up of 118 months (range, 60-305 months) the median disease-free survival time was with 92 months significantly longer after radical en-bloc resection than after parathyroidectomy (32 months, p<0.05). At the time of analysis there was no difference in overall survival benefit between groups (132 vs. 107 months), which is most likely due to the high rate of reoperations and the use of mimpara in the parathyroidectomy group.

**Conclusion:**

If there is any clinical suspicion for PC, an en-bloc resection should be performed to provide a chance of long-term cure.

### Transoral surgery vestibular approach TOETVA – two years experiences and further development TOVARA

(Abstract ID: 668)

E. Karakas^1^, A. Kounnamas^1^, S. Schopf^2^, G. Klein^1^

^1^*Krankenhaus Maria Hilf, Alexianer GmbH, Krefeld*

^2^*Krankenhaus Agatharied, Hausham*

**Background:**

Transoral (para-)thyroid surgery was first described by German study groups. Meanwhile and optimized Transoral Endoscopic Vestibular Approach (TOETVA) has been implemented by Anuwong. We report on our two years experiences, results of TOETVA in Austria and Germany and further development.

**Materials and methods:**

Since June 2017 37 TOETVA procedures and 3 transoral resections with an additional retroauricular access (TOVARA) to retrieve bulky thyroid specimens were performed in patient(s) with single thyroid nodules, sporadic primary hyperparathyroidism or thyroglossal duct cyst. TOETVA was performed using 3 laparoscopic ports, laparoscopic instruments and ultrasonic or bipolar devices inserted at the oral vestibule. Surgical outcome and complications were evaluated.

**Results:**

25 patients presented with solitary thyroid nodules, 12 had multinodular goitre with scintigraphic cold nodules and/or multifocal hyperfunctioning thyroid tissue, two patients suffered from sporadic primary hyperparathyroidism and one patient suffered from a thyroglossal duct cyst. In three patients the thyroid was removed via an additional retroauricular approach (Mean Volume:58ml+19ml). No conversion to open surgery was necessary. Average tumour size was 2.5cm. Temporary hoarseness occurred in two patients. No mental nerve injury occurred. Transient hypoparathyroidism was evident after successful parathyroidectomy and in one patient after thyroidectomy. 20 patients developed a slight postoperative chin hematoma. No infection occurred.

**Conclusion:**

TOETVA is feasible and safe. An additional retroauricular approach may be helpful to remove bulky thyroid specimen. The transoral approach shows promise for patients who are motivated to avoid a visible neck scar. After successful implementation in Austria and Germany further transoral operations are destined in selected patients.

### Adrenalectomy for progressive adrenal metastases in malignant melanoma

(Abstract ID: 724)

S. Müller^1^, F. Hieronimus^1^, M. Senne^1^, A. Kirschniak^1^, A. Königsrainer^1^

^1^*Universitätsklinikum Tübingen*

**Background:**

Adrenal metastases in malignant melanoma patients under additional therapy are often the only site of tumor progression. Aim of the study was to evaluate the outcome of adrenalectomy together with the adjuvant therapy.

**Materials and methods:**

In this study we retrospectively evaluated all consecutive patients between 2008 until 2018 with progressive adrenal metastases of malignant melanoma. Assessed were postoperative outcome parameters, overall survival, progression free survival and the type of adjuvant therapy.

**Results:**

We analyzed 23 patients (14 female) with a median age of 62 years [34-82]. In two patients the adrenal gland was the only location of metastasis while 21 patients had multiple sites of metastases. 17 patients underwent a laparoscopic resection and six an open resection. There was no surgery associated postoperative complication or mortality registered. After a median follow up of 18 months [2-64] 16 patients are still alive, 11 patients with complete remission or stable tumor disease, all of them with the adjuvant of an additional immunotherapy. In no patient a local recurrence developed in the site of adrenal resection.

**Conclusion:**

Adrenalectomy for progressive adrenal metastases in malignant melanoma seems to be a feasible and safe procedure in adjunct with immunotherapy.

### Long-term Outcome of Surgical Resection in Gastroenteropancreatic Neuroendocrine Neoplasias: Results from a German nation-wide multi-centric registry

(Abstract ID: 749)

N. Begum^1^, S. Maasberg^2^, A. Pascher^3^, U.-F. Pape^2^, C. Wurst^4^, A. Weber^5^, A. Raffel^6^, M. Krausch^6^, K. Holzer^7^, D. K. Bartsch^7^, T. J. Ausholt^8^, T. Keck^9^, M. Anlauf^10^, A. Rinke^7^, P. E. Goretzki^11^

^1^*Agaplesion Ev. Klinikum Schaumburg, Obernkirchen*

^2^*Charité - Universitätsmedizin Berlin CVK*

^3^*Universitätsklinikum Münster*

^4^*Klinikum Crailsheim, Crailsheim*

^5^*Universitätsklinikum Essen*

^6^*Marienhospital Gelsenkirchen*

^7^*Universitätsklinikum Marburg*

^8^*Universitätsmedizin Mainz*

^9^*Uniklinik Lübeck, Lübeck*

^10^*Überregionale Gemeinschaftspraxis Limburg, Limburg an der Lahn*

^11^*Charité - Universitätsmedizin Berlin CCM*

**Background:**

Neuroendocrine neoplasia (NEN) are rare and heterogenous. Clinical experience is difficult to acquire. The german NET-registry allows to study longterm outcome after surgical resection.

**Materials and methods:**

This is a retro- and prospectively collected analysis of patient data with gastroenteropancreatic NEN from the german NET-registry (1999-2012) with limited disease (LD, stage I-IIIB).

**Results:**

Data of 2239 patients were recorded, median age was 59 years, gender ratio 1:1,3(f:m), 986 (44%) had LD. 5-years-survival rate (ysr) was 77% for all and 90% in LD. 1635 (73%) had a surgical therapy as 1st to 6th line therapy. 5- and 10-ysr were 83/65% after surgery and 59/35% without surgery for all (p<.001). Resectional margin in LD were 76/16/3% for R0, R1 and R2. 10 ysr were 84, 59 and 42% for R0, R1 and R2 resection (p=.021 R0/R1, p<.001 R0/R2, n.s. for R1/R2). R0 resection rate was 63% for G1/G2 NET and 67% for G3 NEC.

**Conclusion:**

Surgical resection in NEN is associated with improved survival in LD. Complete histological tumor resection (R0) is the key issue for longterm survival. This is seen in excellent survival rates after 10 years, while the 5-ysr are similar for R0, R1 and R2 resection, possibly expressing the indolent nature of NEN. The rate of complete tumor-resection (R0) in LD is independent of tumor grading and comparable for both G1/G2 and G3-NEN. Multimodal approaches and neoadjuvant procedures should be focused on and further evaluated in order to reach this goal. Patients who are reasonable operative candidates with limited disease stage should be considered for resection

### Indocyanine green fluorescence angiography can guide intraoperative localization during parathyroid surgery

(Abstract ID: 779)

I. Karampinis^1^, G. Di Meo^2^, V. Stasiunaitis^1^, A. Gerken^1^, A. Lammert^3^, K. Nowak^4^

^1^*Universitätsmedizin Mannheim*

^2^*University Medical School “A. Moro”, Bari*

^3^*Dialyse-Praxis Grünstadt*

^4^*RoMed Klinikum Rosenheim*

**Background:**

The intraoperative localization of hyperplastic parathyroid glands (PGs) has gained increasing importance in planning surgical strategy both in patients with hyperfunctioning uniglandular and multiglandular parathyroid disease. However, the identification of the parathyroid glands can be extremely challenging even for experienced surgeons, mainly because of the variability in number and anatomy. In this study we used indocyanine green fluorescence angiography for the intraoperative detection of pathologic parathyroid glands.

**Materials and methods:**

This is a retrospective analysis of prospectively collected data. Thirty-seven consecutive patients undergoing surgery for biochemically proven hyperparathyroidism between February 2016 and March 2018 at the University Hospital of Mannheim were eligible for enrolment. Prior to surgery, all patients underwent neck US, and 99mTc-MIBI for preoperative planning, except patients with pHPT due to a single parathyroid adenoma. Analysis was conducted to determine the sensitivity of three modalities (US, 99mTc-MIBI and ICGA). The factors associated with ICG uptake were also evaluated.

**Results:**

Overall 64 lesions were resected. Final histopathologic analysis confirmed the parathyroid origin of 62 of them (96,8%). Intraoperative ICG fluorescence imaging successfully identified 59 of 62 (95, 16%) parathyroid glands. In comparison, preoperative sonography scan could only localize 42 of 62 parathyroids (67,74%) whereas 99mTc-MIBI only identified 35 of the 62 parathyroids (56,45%). Variables as age, sex, preoperative calcium and PTH, size and US positivity were not associated with the ICG uptake. However, a positive MIBI of a parathyroid gland increased 5 times the probability of a positive intraoperative fluorescence angiography according to the univariate analysis.

**Conclusion:**

Our results demonstrated that ICG fluorescence imaging could easily localize PGs and lead to a high resection rate. The analysis revealed that the ICG uptake of PTGs was associated with MIBI positivity, and was especially useful when preoperative imaging techniques failed. Furthermore, this technique has shown to be useful to verify the perfusion of the remnant in patients undergoing subtotal parathyroidectomy, and to successfully identify PGs in re-operative neck surgery or in case of ectopic locations. In conclusion, intraoperative ICG fluorescence imaging has promising application prospects in real-time PG localization increasing the therapeutic efficacy.

### Longterm outcome after total parathyreoidectomy without autotransplantation in renal hyperparathyreoidism

(Abstract ID: 792)

M. Mogl^1^, T. Skachko^1^, E. M. Teegen^1^, N. Rayes^2^, M. Marksteiner^1^, J. Pratschke^1^, P. E. Goretzki^1^

^1^*Charité - Universitätsmedizin Berlin CVK*

^2^*Universitätsklinikum Leipzig - AöR*

**Background:**

While most patients undergo either subtotal parathyroidectomy or total parathyreoidectomy with autotransplantation total parathyreoidectomy has been suggested as alternative treatment option in renal hyperparathyreoidism. Especially recurrence and reoperations hamper the benefit of leaving any parathyroid remnant. This might also lead to late complications like cardiovascular events or impaired kidney function after transplantation.

**Materials and methods:**

We retrospectively analyzed our single-institution results from our database between 1998 and 2018. All patients undergoing total or completion parathyroidectomy after persistence or recurrence were analyzed.

**Results:**

Between 2001 and 2015 we found 66 patients undergoing total parathyroidectomy (67 operations). 15 patients were operated for recurrence/persistence. Average parathormone (PTH) preoperatively was 858.3 ng/mL and dropped to 50.7 at discharge. Further on we saw that patients with PTH < 75 ng/mL at discharge remained stable within the normal range during longterm follow-up. Calcium was within the normal range preoperatively and during longterm follow-up, mostly due to adequate medication. 13 patients died during follow-up, 6 following infectious complications, 4 after cardiovascular events.

**Conclusion:**

Longterm results after total parathyreoidectomy revealed stable PTH- and calcium-levels after successful operation. Death from Cardiovascular events was comparable to patients with subtotal parathyroidectomy. This might even allow for successful kidney transplantation after total parathyreoidectomy.

### Do patients benefit from resection of the primary in stage IV small intestine neuroendocrine tumors?

(Abstract ID: 931)

J. Rütz^1^

^1^*Universitätsklinikum Marburg*

**Background:**

Primary tumor resection in stage IV small intestinal neuroendocrine tumors (SI-NETs) is controversial. Thus, we evaluated the outcome of primary tumor resection at diagnosis in asymptomatic patients with SI-NETs and unresectable distant metastases.

**Materials and methods:**

Patients with stage IV SI-NETs without abdominal symptoms were selected from a prospective database of the ENETS Excellence Center Marburg treated between 1/2004 and 12/2017. Patients were divided in those who underwent primary tumor resection at diagnosis combined with oncologic treatment (PS group) and those who underwent nonsurgical treatment or delayed surgery as needed for symptoms combined with oncologic treatment (NS group). Overall survival (OS), reoperation rates measured from baseline as well as prognostic factors in the PS group were retrospectively evaluated.

**Results:**

The PS group included 114 patients (61 male, 53 female) with a median age of 60 years, the NS group 32 patients (19 male, 13 female) with a median age of 59 years. 11 (35%) patients of the NS group needed surgery for intestinal obstruction after median 59 (range 1 to 186) months. Overall, the median OS was not statistically different between the PS (37 months) and the NS groups (45months, p=0.29). A liver metastasis burden >50% was a strong negative prognostic factor in both goups with a median survival of 29 months compared to a median survival of 39 months in patients with <50% (p<0.01). The rate of operative procedures for intestinal obstruction after initial treatment was significantly lower in the PS group (7 of 114, 6%) compared to the NS group (11 of 32 35%, p<0.01). In the PS group a R2 resection of the primary and a liver metastasis burden >50% were significant negative prognostic factors (p<0.01), whereas age, gender, Ki67 index and lymph node ratio were not significantly associated with survival.

**Conclusion:**

In patients with asymptomatic stage IV SI-NET resection of the primary tumor seems to be only beneficial, if the liver metastasis burden is less than 50%.

## DGAV: Gender medicine

### Gender differences in patients with colorectal carcinoma

(Abstract ID: 216)

S. Merkel^1^, S. Zech^1^, V. Schellerer^1^, M. Brunner^1^, J. Baecker^1^, K. Weber^1^, R. Grützmann^1^

^1^*Universitätsklinikum Erlangen*

**Background:**

Gender differences in colorectal carcinoma (CRC) were examined with special interest in incidence, age at diagnosis, tumor site, surgical and multimodal treatment, postoperative complications, quality indicators and long-term prognosis.

**Materials and methods:**

The prospectively collected data from 1998-2012 were analyzed. The following subgroups were evaluated: (1) patients with colon carcinoma, (2) patients with rectal carcinoma, (3) patients with synchronous metastatic colorectal carcinoma, (4) patients <=50 years and (5) patients >=80 years of age.

**Results:**

3475 patients with CRC were included in the study, 2173 men (63%) and 1302 women (38%). Men were significantly younger (median 65 vs 66 years, p=0.005). Women were significantly more likely to be diagnosed with colon cancer (56.0% vs 46.8%) and men with rectal cancer (53.2% vs 44.0%; p<0.001).

(1) In 729 men and in 513 women a colon carcinoma was resected curatively. Women had significantly better 5-year overall survival (OS 74.4% vs 79.3%; p=0.021) and disease-free survival (DFS 68.4% vs 73.7%, p=0.018). While women had significantly more locoregional recurrences (LR), men had more distant metastases (DM).

(2) Rectal carcinoma was resected curatively in 837 men and 430 women. In men, the lower rectal third was significantly more frequently affected (35.8% vs 27.2%, p=0.002). They were more frequently treated with neoadjuvant chemoradiation (nCRT) (47.3% vs 34.0%, p<0.001) and abdominoperineal excision (20.5% vs 15.3, p<0.001). Men had significantly higher postoperative morbidity, especially anastomotic leak (6.4% vs 3.4%, p=0.048) and bladder dysfunction (p=0.009). In men, a significantly higher 5-year LR rate was observed (9.2% vs 6.4%, p=0.038). In patients without nCRT, women had better survival (5-year OS 72.5% vs 75.0%, p=0.034, DFS 62.7% vs 68.6%, p=0.009). In patients with nCRT followed by surgery, no significant gender-specific prognostic differences were found.

(3) Among the 674 patients (414 men, 260 women) with synchronous metastatic CRC, women were older (median 66 vs 63 years, p=0.033), more likely to get cancer of the right colon (37.7% vs 24.2%, p<0.001) and more frequently had emergency surgery (11.5% vs 7.2%, p=0.057). They were less frequently R0-resected (23.8% vs 27.8%, p=0.259) and less frequently treated with multimodal therapy (75.2% vs 83.1%, p=0.005). Women >=65 years had a significantly shorter OS than men (median 12 vs 18 months, p=0.031).

(4) Among the 393 patients <=50 years with CRC, women (n=153) were younger than men (n=240, median 45 vs 46 years, p=0.010). Young men were significantly more likely to develop tumors of the lower rectal third (39.9% vs 23%), while in women tumors of the upper third were more frequently (24% vs 9.8%, p=0.003). Therefore, men were significantly more likely to receive nCRT (52.1% vs 35%, p=0.022). Young women with rectal cancer had significantly better OS than men (82.1% vs 73.5%, p=0.007) and, after curative resection, fewer LR (2.9% vs 11.4%, p=0.010).

(5) Among the 365 patients >=80 years of age (175 men and 190 women) with CRC, women were older (median 82 vs 83 years, p=0.045) and had significantly more frequently DMs (10.3% vs 22.6%, p=0.002). After curative resection of colon carcinoma women had a better OS, after curative resection of rectal carcinoma men had a better OS.

**Conclusion:**

Gender-specific differences were found in various subgroups between men and women with CRC. The quality of treatment was very good in both sexes.

## DGAV: Hernia surgery

### Postoperative-treatment following open incisional hernia repair: A survey and a review of literature

(Abstract ID: 34)

C. Paasch^1^, S. Anders^1^, M. W. Strik^1^

^1^*Helios Klinikum Berlin-Buch*

**Background:**

Incisional hernias of the abdominal wall are frequent complication after laparotomy (9-20%). Open incisional hernia repair with sublay mesh placement (SMP) on the posterior rectus sheath is described as being a sufficient method for repairing incisional hernia. In order to ensure wound healing and to therefore prevent recurrence, carrying an abdominal binder (AB) or a pressure dressing (PD) and physical rest for a certain time is the common postoperative recommendation, though the evidence for post-operative treatment is low. Hence, we conducted a survey to reveal the different recommendations given by surgical departments (SD).

**Materials and methods:**

We conducted a survey among 65 German SDs of the HELIOS Hospital Group. The SDs were interviewed about the number of open incisional hernia repair with SMP in the time frame of 2013-2014, the known recurrence rate (RR), their recommended prescription of the AB/PD and the time of physical rest.

**Results:**

The head physicians of 48 surgical departments answered the questionnaire. The survey revealed 42 different recommendations of postoperative-treatment. The majority of the SDs advices 4 weeks (20,5%) of physical rest and no prescription of the AB (29,5%). No correlation between the known RR and the duration of physical rest was detected. No head physician’s prescribes a PD.

**Conclusion:**

Due to our findings we assume that a short period of physical rest is a considerable postoperative treatment following an open incisional hernia repair with SMP. By reducing the individual incapacity for work and immobility this would have a social-economic impact. The use of a PD may prevent seroma formation. Further investigations with randomised clinical trials are mandatory to support our hypothesis.

### Totally extraperitoneal (TEP) inguinal hernia repair in patients with prior lower abdominal surgery: A Meta-Analysis

(Abstract ID: 331)

D. Prassas^1^, W-T. Knoefel^1^, A. Krieg^1^

^1^*Universitätsklinikum Düsseldorf*

**Background:**

Prior lower abdominal surgery is generally considered as a relative contraindication for laparoscopic totally extraperitoneal (TEP) inguinal hernioplasty. We conducted a meta-analysis of studies comparing the feasibility and safety of TEP inguinal hernia repair between patients with (PS) and without history of lower abdominal surgery (NS).

**Materials and methods:**

A systematic literature search for studies comparing the outcome of TEP inguinal hernioplasty in patients with and without previous lower abdominal surgery was conducted. Data regarding postoperative outcomes were extracted and compared by meta-analysis. The Odds Ratio and Standardized Mean Differences with 95% Confidence Intervals (CI) were calculated.

**Results:**

Seven comparative cohort studies were identified, involving a total of 1657 cases (PS: n=326, NS: n=1331). There was a statistically significant difference noted between PS and NS favoring the NS group with regard to both primary outcomes intraoperative morbidity (OR=2.85; 95% CI [1.19, 6.80]; p=0.02; 7 studies; I2=33%) and postoperative morbidity (OR=2.14; 95% CI [1.28,3.58]; p=0.004; 5 studies; I2=0%) in the multi-port sub-group.

For the secondary endpoints, conversion rate, peritoneal tears, major intraoperative bleeding, postoperative haematoseroma, operative time, anddelay in return to normal activities a statistically significant difference favoring the NS group was noted.

**Conclusion:**

Current evidence suggests that patients with previous lower abdominal surgery undergoing TEP inguinal hernia repair do not have the same benefits as those with no history of surgery.

### Fibrin glue vs. staple fixation of extra-light titanized meshes in laparoscopic inguinal hernia repair (TAPP): A single-center experience of 612 cases

(Abstract ID: 399)

U. Wirth^1^, M.-L. Saller^2^, T. von Ahnen^3^, H. M. Schardey^3^, S. K. Schopf^3^

^1^*Uniklinik München*

^2^*Schön Klinik Vogtareuth*

^3^*Krankenhaus Agatharied, Hausham*

**Background:**

Inguinal hernia repair is one of the most frequently performed surgical procedures. Endoscopic techniques like TAPP and TEP have become standard of care together with the conventional open techniques. Especially in endoscopic inguinal hernia repair, there is a confusing amount of different meshes and fixation techniques with an impact on patients’ perioperative and long-term outcome. We present the first single-center data on the use of titanized extra-lightweight meshes and fibrin glue fixation compared to staple fixation regarding long-term outcome, especially chronic pain.

**Materials and methods:**

A clinical trial with retrospective analysis of patient- and procedure-related data and prospective questionnaire-based follow-up (Herniamed) was conducted. 612 inguinal hernia repairs performed in a specialized hernia center in 501 patients were analyzed. A standard TAPP technique was used with placement of TiMesh extralight (16g/m2) and either fibrin glue or staple fixation. Procedure- and patient-related data are compared between groups with regard to perioperative complications and long-term outcome.

**Results:**

612 TAPP procedures were performed in 501 patients. Fibrin glue was used in 519 (85%) and staple fixation in 93 (15%) cases. There were significant differences between groups regarding the distribution of hernia size according to the EHS groin hernia classification (χ^2^; p=0.001), mesh size (p<0.001), duration of the surgical procedure (p<0.001), and pain on POD1 (p=0.024). No between-group differences were noted regarding perioperative complications such as seroma or hematoma formation and need for re-laparoscopy. During a mean follow-up of 30.85 ± 20.74 month with a follow-up rate of 80%, there was no difference in long-term outcome, especially for rate of recurrence (χ^2^; p=0.098) and development of chronic pain (χ^2^; p=0.985). The overall rate of recurrence was 2.8% (n=17), and in 2.1% of surgical cases (n=13; 2.1% vs. 2.2%) patients complained of chronic pain.

**Conclusion:**

Inguinal hernia repair using extra-lightweight titanized meshes and fibrin glue fixation is safe and feasible compared to staple fixation regarding the long-term outcomes recurrence and development of chronic pain. As expected, the rate of patients developing chronic pain was extremely low at 2.1%, independent of the type of fixation.

### Anatomical and functional reconstruction of incisional hernias without component separation – the corset technique

(Abstract ID: 462)

A. Kehrer^1^, S. Geis^1^, C. Taeger^1^, F. Brennfleck^1^, M. N. Scherer^1^, L. Prantl^1^

^1^*Universitätsklinikum Regensburg*

**Background:**

Incisional hernia developement following laparotomy represents one of the most frequent complications, with an incidence described in the literature of up to 13%. Mesh repair alone has shown recurrence rates as high as 36%. Commonly, hernia reconstruction with mesh repair results in persistent rectus diastasis. While component separation, as described by Ramirez, may achieve successful medialising of the rectus muscles, simultaneous weakening of abdominal wall integrity occurs. Our retrospective study describes a new method which enables the surgeon to anatomically and functionally reconstruct (recurrent) large incisional hernias without component separation and with preservation of abdominal wall layer integrity

**Materials and methods:**

Between 2013 - 2018 a total of 17 patients with (recurrent) incisional hernias were operated on using the corset technique. In 6 cases (multiple) previous mesh repairs had failed. In 5 other patients resorbable mesh and split thickness skin grafts (STSG) had been applied for abdominal closure. In some of these patients previous non-resorbable mesh repair had been unsuccessful. The remaining 6 cases had not received mesh repair. Rectus diastasis in this patient series was measured 3-20 cm. Follow-up was 24 months.

**Results:**

The great majority of patients demonstrated very promising functional outcomes. Complete mid line union of rectus muscles was achieved in 15/17 cases (88%). Failure was seen in 2 desolate cases with persistent fistula. Recurrence rate of functional relevant hernia formation was seen in 3/17 patients (18%). A supplemental mesh implantation was used in 6/17 of cases. The corset technique successfully centralised the rectus muscles anatomically in the midline without persistent rectus diastasis. Prerequisites for successful reconstruction should include intact rectus abdominis muscles and strong sutures.

**Conclusion:**

The corset technique for incisional hernia repair achieves an anatomical recconstruction with adequate functional outcomes and relinquishment of component separation.

### Laparoscopic extraperitoneal endoscopic staple-based sublay operation (LEESS) with mesh – interims analysis of an initial consecutive patient cohort

(Abstract ID: 700)

D. Hashim^1^, F. Meyer^2^, N. Albayrak^1^

^1^*St. Anna Hospita , Herne*

^2^*Universitätsklinikum Magdeburg*

**Background:**

Background Patients with symptomatic midline abdominal hernia (umbilical, infraumbilical, Port, and/or epigastric hernias) and concomitant rectus abdominis diastasis represent a growing clinical problem. The optimal management of this complex hernia situation is the subject of an ongoing debate in the literature.

This paper reports on the early results of an innovative surgical technique aimed at managing this hernia situation.

Aim To analyze feasibility and safety, in particular, early postoperative outcome characterized by morbidity (in particular, by intraoperative, specific and general complication rate) and mortality based on a unicenter observational study to reflect daily surgical practice in hernia surgery using a novel surgical approach such as LEESS.

**Materials and methods:**

Methods Laparoscopic Extraperitoneal Endoscopic Staple-based Sublay operation (LEESS) with mesh is a surgical technique recently known in the literature for its good outcome for midline hernia repair via transperitoneal route (Brazilian Technique) and Endoscopic Component Separation Techniques. The early postoperative outcome results for the first consecutive 50 patients are presented here in this systematic clinical unicenter observational study on quality assurance and reflecting daily surgical practice in a consecutive patient cohort (study design).

**Results:**

Results In 5 out of 50 (10 %), a symptomatic subfascial seroma was observed (minor complication). As a major complication, two patients (4 %) developed postoperative complications requiring redo surgery. These were two cases of internal herniation through a defect in the posterior rectus sheath, the herniated intestine was reduced and the defect was sutured laparoscopically. All other complications were successively managed with conservative treatment. During the mid-term postoperative course, i.e., after 11 months, 4 out of 50 (8 %) patients reported occasional pain, including pain at rest in one patient.

**Conclusion:**

Conclusion The LEESS technique with mesh augmentation is an innovative, minimally invasive, feasible and safe surgical procedure for treatment of patients with a complex abdominal wall hernia comprising symptomatic umbilical, port, and/or epigastric hernias with concomitant rectus abdominis diastasis.

**Picture:**
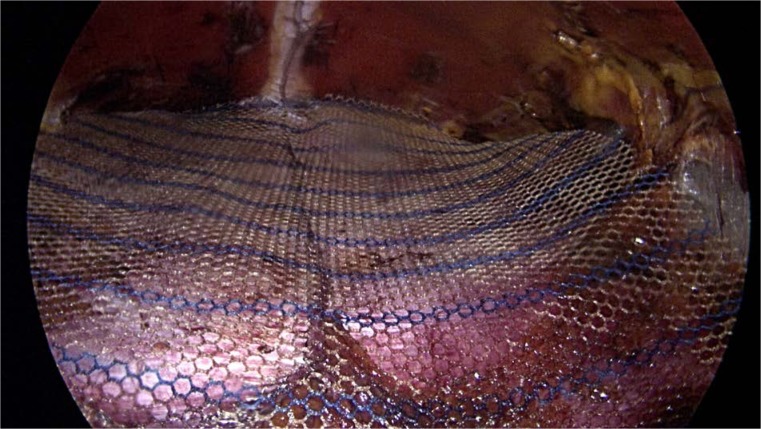
stapling rectus muscels with mesh in sublay space

### Novel Suturing Technique for Fascia Closure after Laparotomy: Spider Suture to increase the tearing force of the fascia

(Abstract ID: 706)

R. Demir^1^

^1^*MVZ PD Dr. Demir & Kollegen, Nürnberg*

**Background:**

The objective of this study was to demonstrate a new suturing technique in reference to fascia closure after laparotomy in an experimental setup to increase the tearing force of the fascia.

**Materials and methods:**

Two ordinary DIN A4 papers, which represents the fascia were sutured at the edges in a length of approx. 5 cm with the known continuous technique in the abdominal surgery and with the new technique, we called it: Spider suture. A bucket arranged on the interconnection was filled with sand until the sutures teared. Measuring the final weight of the bucket allowed comparing the carrying capacities of the paper of the two techniques.

**Results:**

A significant (Fisher’s test, p=0.017) increase of mean carrying capacity from 1776.5g (classic continuous suture) to 5101.75g (basic Spider suture) was found. The carrying capacity of the paper was increased to 287% compared to the classic continuous suture.

**Conclusion:**

Spider suture could be an effective improvement in closure of the abdominal wall in abdominal surgery to minimize the risk of developing of incisional hernia. This method allows the surgeon to perform an abdominal closure adapted to the quality of patient’s fascia. Further clinical studies will show the effectiveness of this method.

### Onlay mesh repair in huge incisional hernias > 15 cm diameter with open abdomen

(Abstract ID: 752)

N. Meiers^1^, T. Carus^1^

^1^*Asklepios Westklinikum Hamburg*

**Background:**

The management of abdominal hernias of > 15 cm diameter and the special case of the abdomen apertum with a large eventration, can be demanding. The purpose is the closure of the fascial defect and additional mesh reinforcement.

**Materials and methods:**

A 57 year old patient with abdominal hernia and evisceration was admitted for hernia repair after having undergone a large number (>14) of abdominal operations. These were for instance cholecystectomy, choledochojejunostomy, laparotomy with adhesiolysis at least four times, inflammation of the abdominal wall with numerous cycles of abdominal vacuum therapy, surgical treatment of several enterocutaneous fistulas and mesh grafts. All operations were performed between 1990 and 2017 resulting in an open abdomen only covered by mesh graft. The patient had already undergone one hernia repair with an onlay mesh one year before in another hospital. She was told that no further operations could be done. The CT scan showed the absence of the abdominal wall with a musculofascial defect of 22x24 cm.

After resection of the mesh graft and complex adhesiolysis, a total closure of the fascial defect was reached, restoring the abdominal wall anatomically without tension with non absorbable sutures. Component separation or relaxing incision of the fascia were not necessary. For reinforcement we used a 20x30 cm2 CICAT mesh in an onlay position. The mesh was fixed to the fascia with non absorbable sutures. A primary skin closure was reached with a relaxing skin incision on the left side of the lower abdomen.

**Results:**

While postoperative intensive care observation, the patient showed no symptoms of abdominal compartment or dyspnoea. The wound in the midline healed properly. Due to chronic pain and former opioid use in high doses, the patient received a peridural catheter. Transfusion of two erythrocyte concentrates was necessary. The patient could go back to the peripheral ward after 3 days. The wound of the left lower abdomen was treated with a pico vacuum therapy. The patient was discharged 10 days after the operation.

**Conclusion:**

Special treatment like compartment separation or relaxing incisions of the fascia to achieve a total closure of large musculofascial defects, which can cause further complications, is not always necessary for large abdominal hernias. Primary fascia closure with mesh enhancement is demanding, but often feasible. Since there are no guidelines for the surgical management of open abdomen, the indication for surgical management has to be taken individually.

### B.U.B.I. – Botox Supported Abdominal Wall Reconstruction in IPOM Technique – no more need for Ramirez?

(Abstract ID: 862)

N. Bohnert^1^, E. Elieyioglu^1^, A. Bär^1^, A. Ulrich^1^, B. Lammers^1^

^1^*Lukaskrankenhaus Neuss*

**Background:**

One oft he biggest problems in hernia surgery is, to get sufficient results in cases of hernias with big midline defects (W3 hernias) and/or "loss of domain" hernias.Even in cases of small defects, "loss of domain" can cause serious problems, since reposition of the hernia sac contents will result in an increased intraabdominal pressure with all its problems (compartment syndrome/respiratory failure etc) and too much tension on the reconstructed midline.To avoid dissection of healthy parts oft he abdominal wall like in the Ramirez operation, which we think is not the best option for multimorbide patients, we developed our own method of Botox supported abdominal wall reconstruction in IPOM technique, B.U.B.I

**Materials and methods:**

In this method we combine the Botox induced relaxation of the abdominal wall with the IPOM technique to achieve a full reconstruction oft he midline combined with a mesh. Inclusion Criteria were W3 and/or Loss of Domain Hernias. Exlusion Criteria were Pregnancy, Myasthenia, ALS, Breastfeeding, Urostoma and Enterostoma.2 weeks prior to surgery patients were treated with sonography guided Botox injection in both sides of the lateral abdominal wall muscles.To verify the result of the injection a low dose CT scan is done at the day before surgery.Intraoperative the size of the defect was documented.During the healing process of the midline and the ingrowth oft he mesh the Botox effect decreases 4-6 months until it is gone. During that period the abdominal cavity can get used to the situation slowly without causing any pressure related problems.

**Results:**

30 Patients have been treated with Botox injection so far and 28 of them have already been operated. In 21 of 28 cases we could do a full reconstruction oft he midline (Defect sizes have been between 6x6 cm and 30x35 cm). In 7 cases we could at least reduce the defect. Mesh sizes have been between 20x30 cm to 28x37 cm.Intraoperatively no problems occured and postoperative we had 1 SISSI. Reoperation was not necessary. 22 patients already went through follow up after 6-12 months (clinical/CT scan) and are without a hernia recurrence so far. We havent seen any Botox related problems so far.

**Conclusion:**

As known reconstruction oft he abdominal wall is a challenge especially in W3 and/or “loss of domain” hernias.So far the Ramirez operation in all its variations (open, laparoscopic, laparoscopic assisted etc.) has been one of the most common procedures to gain more tissue for closing the midline defect. Even though this method can give 7-10 cm space per side in some cases, we think it is not the best solution. Especially in multimorbide older patients the high rate of wound complications is a serious problem. Avoiding these problems and preserving the healthy lateral parts oft he abdominal wall which are dissected for example in the Ramirez operation, B.U.B.I. is a chemical component seperation technique without any risk for the patient. Our patients show, that it s even possible to do a midline recontruction in huge hernias without creating any pressure related problems. As a positive side effect, Botox reduces the postoperative pain in the lateral abdominal wall. B.U.B.I. is a safe and efficient procedure to treat patients with a W3 hernia and/or “loss of domain” and is a good alternative for the Ramirez operation.

## DGAV: Liver / bile / pancreas

### Inflammatory Myofibroblastic Tumor of the Liver – a rare case presentation with fever treated by immediate hepatic resection

(Abstract ID: 84)

A. Filips^1^, M. Maurer^1^, M. Monteani^1^, G. Beldi^1^, A. Lachenmayer^1^

^1^*University Hospital Bern*

**Background:**

Inflammatory myofibroblastic tumors of the liver (IMTL) are extremly rare neoplasms and very little is known about their pathogenesis and etiology. Due to the intermediate biological behavior and the risk for local recurrence and metastases, surgical resection is usually recommended. Unfortunately, a pre-operative diagnosis is extremely difficult to obtain and the definite diagnosis can often only be established post resection.

**Materials and methods:**

Retrospective Case report and review of the current literature of patients with IMTL.

**Results:**

We herein present a case of an otherwise healthy 32-year-old woman who presented with intermittent fever, unclear blood loss, malaise and right flank pain 4 months postpartum. Initial ultrasound examination found a liver mass in segment IVa/b of uncertain dignity, confirmed by further diagnostic work-up (CT / MR / PET-CT) that suggested an adenoma (Figure). Immediate hepatic resection was performed achieving a negative resection margin and all clinical symptoms resolved. Histological analysis diagnosed the rare finding of an inflammatory myofibroblastic tumor of the liver and revealed cytoplasmic anaplastic lymphoma kinase (ALK) expression by immunohistochemistry (Figure). A comprehensive review of the literature confirmed the rarity of this tumor entity in the liver, with only very few cases reported world-wide. Therefore, no diagnostic tools have been established so far and resection is usually recommended for unclear liver lesions.

**Conclusion:**

IMTLs are extremly rare and difficult to diagnose. Due to their intermediate biological behavior, surgical resection should be performed whenever feasible and patients should be followed-up in order to detect recurrence and metastasis as early as possible.

**Picture:**
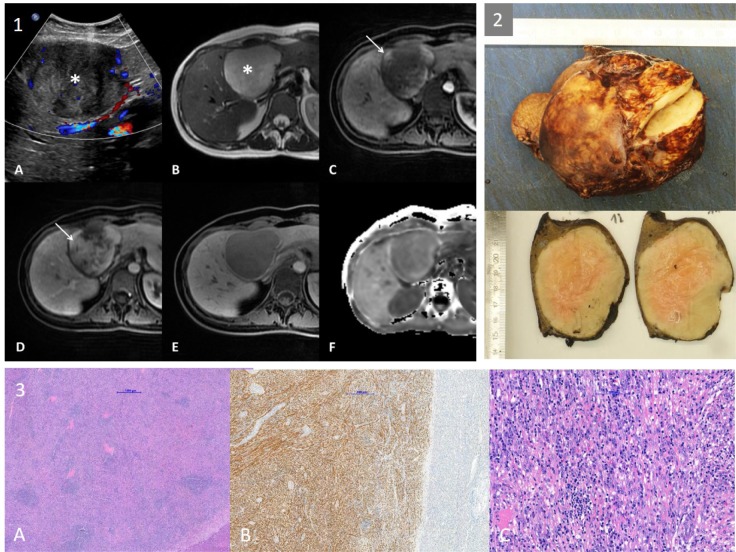
Figure: (1) IMTL imaging features: (A, asterisk) ultrasound image, (B) magnetic resonance imaging (MRI): homogeneous high signal in T2-weighted imaging (asterisk), (C) early enhancement in the arterial phase with hepatocyte specific contrast medium at the rim (arrow), (D) strong enhancement in the venous phase (arrow), (E) low intracellular uptake in the hepatobiliary phase after 20 minutes, (F) no clear diffusion restriction in the diffusion-weighted imaging (apparent diffusion coefficient, ADC). (2) Postoperative macroscopic pathology of the IMTL (3) Histology of a well demarcated firm vascularized tumor mass with spotty inflammatory infiltrate, (A) at higher magnification bland proliferation of spindle cells in broad fascicles, (B) scattered lymphocytes and plasma cells and (C) intense positivity of the spindel cells for ALK

### IgG4-related sclerosing cholangitis mimicking hilar cholangiocarcinoma (Klatskin tumor): A case report of a challenging disease and review of the literature

(Abstract ID: 88)

A. Mittelstädt^1^, P. N. Meier^1^, E. Dankoweit-Timpe^2^, B. Christ^1^, J. Jähne^1^

^1^*DIAKOVERE Henriettenstift, Hannover*

^2^*Institute for Pathology, Hannover*

**Background:**

Even though IgG4-related disease has gained increased attention worldwide, the diagnosis remains challenging. IgG4-related sclerosing cholangitis (IgG4-SC) is not well described in the western hemisphere and may mimic cholangiocarcinoma (CC), especially when occurring without other symptoms such as, e.g. concurrent pancreatitis or retroperitoneal fibrosis. We present a case to add further information to the diagnosis and treatment of this challenging disease.

**Materials and methods:**

A 60-year-old male patient presented with painless jaundice. Prior medical history showed diabetes mellitus type I, high blood pressure, and deep vein thrombosis. Diagnostic investigations were strongly suspicious of a Klatskin tumor, although biopsies were inconclusive. The tumor marker Carbohydrate Antigen 19-9 (CA 19-9) was elevated. Prior to the recommended surgery, the patient had two second opinions in two different university hospitals, both arguing for surgery as well.

**Results:**

The patient received hilar resection with right hemihepatectomy. During the postoperative course, some major complications occurred, i.e. recurrent pleural effusion, abscess in the liver resection area, sepsis, ileus, and restricted liver metabolism. Treatment with prednisolone did not show any improvement. Approximately 3 months after surgery, the patient died in consequence of acute respiratory failure. Histology showed no signs of CC, but IgG4-SC could be diagnosed.

**Conclusion:**

In the case of preoperative signs of CC, differential diagnosis of IgG4-SC needs to be considered, in particular, in cases with missing histologic proof of malignant disease.

### Minimal-invasive versus open hepatectomy for colorectal liver metastases: A bicentric comparison of postoperative outcomes and long-term survival using propensity score matching analysis

(Abstract ID: 91)

A. Andreou^1^, G. Beldi^2^, S. Knitter^1^, D. Kradolfer^2^, V. Banz^2^, A. Lachenmayer^2^, S. Wabitsch^1^, W. Schöning^1^, R. Öllinger^1^, D. Candinas^2^, M. Schmelzle^1^, J. Pratschke^1^

^1^*Charité - Universitätsmedizin Berlin - CVK*

^2^*Inselspital, Bern*

**Background:**

Minimal-invasive hepatectomy (MIH) has been increasingly performed for benign and malignant liver tumors with promising short-term results. However, the oncological results of MIH for the treatment of patients with colorectal liver metastases (CRLM) needs to be determined in order to allow widespread introduction of the technique.

**Materials and methods:**

Clinicopathological data of patients who underwent liver resection for CRLM between 2005 and 2017 at the Department of Surgery of the Charité Berlin were assessed. A validation cohort from the Inselspital Bern was additionally evaluated. Postoperative outcomes und long-term survival of patients following MIH were compared with those of patients undergoing conventional open hepatectomy (OH) after 1:1 propensity score matching.

**Results:**

During the study period, 768 patients underwent liver resection for CRLM with curative intent at the Charité Berlin. Fifty patients underwent MIH and were compared with a matched cohort of 50 patients who underwent OH. The rate of patients with preoperative chemotherapy (50% vs. 48%, p = 0.841), multiple lesions (58% vs. 58%, p = 1.00), tumor size >50 mm (18% vs. 23%, p = 0.546), major resection (54% vs. 52%, p = 0.841), and postoperative chemotherapy (32% vs. 28%, p = 0.663) was comparable between the two groups (MIH vs. OH). MIH was associated with lower postoperative major complication rates (18% vs. 42%, p = 0.011), and shorter length of hospital stay (10 vs.13 days, p = 0.008) compared to OH. Postoperative mortality was comparable between MIH and OH (0% vs. 4%, P = 0.495). After a median follow-up time of 65 months, MIH showed better 5-year disease-free survival rates (55% vs. 31%, p = 0.956) in comparison to OH, however differences did not reach statistical significance. When evaluating the validation cohort of 33 patients undergoing MIH in Bern (3% major resection), the benefits of MIH for CLM could be confirmed. MIH was associated with lower major complication rate (6% vs. 24%, p = 0.027), and shorter length of hospital stay (4 vs. 9 days, p < 0.0001) compared to OH. Postoperative mortality (0% vs. 0%, p = 1.00) and 5-year disease-free survival rates (44% vs. 28%, p = 0.118) were also comparable between MIH and OH.

**Conclusion:**

MIH for CLM is associated with lower postoperative morbidity and shorter length of hospital stay, resulting in oncologic outcomes comparable to those achieved with the established OH. Our findings suggest that MIH should be considered as the preferred method for the treatment of curatively resectable CLM.

### Inhibition of Vascular Endothelial growth factor protects against the development of oxaliplatin-induced sinusoidal obstruction syndrome in a murine model

(Abstract ID: 95)

S. Knitter^1^, G. Duwe^1^, A. S. Beierle^1^, S. Pesthy^1^, K. Führer^1^, S. Lippert^1^, P. Tang^1^, A. Reutzel-Selke^1^, P. Lohneis^1^, R. B. Schmuck^1^, M. Schmelzle^1^, I. M. Sauer^1^, M. Bahra^1^, J. Pratschke^1^, A. Andreou^1^

^1^*Charité - Universitätsmedizin Berlin - CVK*

**Background:**

Inhibition of vascular endothelial growth factor (VEGF) by bevacizumab as part of oxaliplatin-based chemotherapy in patients with colorectal liver metastases has shown protective effects against the development of Sinusoidal Obstruction Syndrome (SOS). Our aim was to evaluate the impact of an anti-VEGF treatment on SOS, liver regeneration and function after major hepatectomy in a murine model of oxaliplatin-induced SOS.

**Materials and methods:**

C57Bl/6 mice (n = 116) were treated with intraperitoneal oxaliplatin (Ox), oxaliplatin + anti-VEGF (OxAV), or glucose (Glu) over five weeks. One week after last treatment, mice were either sacrificed or subjected to major hepatectomy. Mice who underwent hepatectomy were sacrificed after 24, 36, 48 and 72 hours (n = 3 each), respectively. Liver tissue was used for the histological analysis of SOS. Plasma was collected for the analysis of alanine aminotransferase, aspartate aminotransferase, bilirubin, VEGF-A, hepatocyte growth factor (HGF) and plasminogen-activator inhibitor 1 (PAI-1). Liver regeneration was assessed by quantitative PCR for Ki-67 and immunohistochemistry for BrdU. Quantitative PCR was also performed to evaluate gene expression levels of VEGF-A, VEGF-R1 and VEGF-R2.

**Results:**

OxAV resulted in less frequent development of SOS than Ox (58% vs. 90%, p= 0.001) and was also associated with a reduced rate of hepatocellular damage (18% vs. 38%, p= 0.045). Subgroup analysis of mice that developed SOS due to Ox treatment revealed increased median plasma levels of VEGF-A (11.3 vs. 7.4 pg/ml, p= 0.002) and PAI-1 (93.0 vs. 9.3, p< 0.0001), while HGF was decreased (315.3 vs. 859.2 pg/ml, p = 0.001) in comparison to Glu. Expression of VEGF-A, VEGF-R1 and VEGF-R2 mRNA in liver tissue did not differ between Ox and OxAV (1.7 vs. 1.6, p = 0.865, 0.9 vs. 1.0, p= 0.905, and 1.0 vs. 1.2 fold-change,p= 0.208). In the Ox group, ALT was increased 36 hours after major hepatectomy in comparison to OxAV and Glu (1,784 vs. 239 vs. 618 U/l, p= 0.027). Cholestasis was experienced in 60%, 17% and 17% after Ox, OxAV and Glu treatment, respectively (p= 0.038). Expression of Ki-67 mRNA in liver tissue was higher in the OxAV group than in the Ox group 36 (2.5 vs. 0.7 fold-change, p = 0.250) and 48 hours (8.8 vs. 3.9 fold-change, p = 0.400) after hepatectomy but failed to reach statistical significance. Accordingly, BrdU ratio was lower at 72 hours after hepatectomy in the Ox group than in the Ox and Glu group (5% vs. 22% vs. 20%, p= 0.005).

**Conclusion:**

Inhibition of VEGF protects against the development of SOS in a murine model of oxaliplatin-induced SOS and improves liver regeneration after major hepatectomy. PAI-1 and HGF may present other possible key factors in the pathogenesis of SOS development.

### Progressive Myofibroblastic Tumor of the Pancreatic Head – a rare case

(Abstract ID: 99)

M. Alnabki^1^, S. Elhabash^1^, I. Dimopoulos^1^, M. Sorleto^1^, B. Gerdes^1^

^1^*Johannes Wesling Universitätsklinikum Minden*

**Background:**

A 46-year-old female patient, with a prior medical history of abdominal pain, presented to us in 07/2017 with an incidental finding of a 1.8 x 2 cm mass adherent to the pancreatic head in the computed tomography (CT ). In the last years the patient suffered from diarrhea, nausea, recurrent epigastric abdominal pain and recently from progressive back pain. Three months prior to presentation in our department, the patient was diagnosed with pancreatic insufficiency with increased size of the pancreatic head in the abdominal sonography but without biochemical evidence of acute pancreatitis. Furthermore, she reported about a positive family history for colon and lung cancer. Further diagnostic workup revealed a normal CA 19-9 tumor marker (6 U/ml), and a cystic lesion with wall calcifications poorly demarcated from pancreatic head without evidence of malignancy or cystic adenoma in the endosonographic examination. Additional workup showed no evidence of autoimmune pancreatitis or neuroendocrine tumor.

The Magnetic resonance cholangiopancreatography (MRI) showed the prior described cystic pancreatic head mass without dilatation of the pancreatic duct. A CT guided biopsy of the mass was recommended by the tumor board and was histologically consistent with an inflammatory myofibroblastic tumor (IMT). Six months later, a follow up MRI showed a significant progression of the pancreatic lesion to 3.5 x 2.6 cm, so that a pylorus preserving pancreatic head resection (PPPD) was recommended by the interdisciplinary tumor board. The histologic examination of the resected mass showed a mesenchymal tumor of the pancreatic head (6.5x5.0x3.0 cm) with a spindle shaped cells with inflammatory components, prominent calcifications and very low proliferation activity which was consistent with IMT. Postoperatively, a biochemical leak was treated conservatively.

**Conclusion:**

IMT are rare mesenchymal tumors (incidence 0.04-0.7%) which can affect a variety of organs, mainly the lung and rarely the pancreas. Patients with IMT of the pancreas can present clinically with a wide variety of symptoms. However, those lesions are mostly discovered incidentally and can be mistaken with pancreatic cancer. IMT are recently classified by the World Health Organization (WHO) as fibroblastic sarcoma or myofibroblastoma with an intermediate biological potential. Local recurrence of IMT has been reported between 2%-25%, with a rare risk of distant metastases. Surgical resection of pancreatic MIT has been favorably considered as treatment of choice (Oncol Lett. 2016 Aug; 12(2): 1546-1550).

### Stereotactic Image-Guided Microwave Ablation (SIMWA) for Hepatocellular Carcinoma – a monocentric experience using a computer-assisted navigation system

(Abstract ID: 110)

A. Lachenmayer^1^, L. Frehner^1^, P. Tinguely^1^, M. Maurer^2^, M. Knöpfli^1^, S. Weber^2^, D. Candinas^1^, V. Banz^1^

^1^*Universitätsspital Bern*

^2^*University Hospital Bern*

**Background:**

Local tumor ablation plays an important role in the treatment of hepatocellular carcinoma (HCC) and is often used as bridging or downstaging for patients listed for transplantation. While image-guided navigation technology has recently entered the clinical setting, reports of large patient series are still rare.

**Materials and methods:**

Retrospective analysis of patients treated with stereotactic image-guided microwave ablation (SIMWA) for HCC at our institution between 01/2015 and 12/2017. Each intervention was performed using CT-guidance with needle trajectory planning by landmark-based registration and an aiming device for precise needle placement. Patients were under general anesthesia with jet-ventilation to optimize registration accuracy.

**Results:**

In total 174 SIMWAs were performed in 88 patients during 119 interventions. Mean age was 66 (46-84) years, 74 (84.1%) were men and the majority of patients were Child Pugh Class A (74%). Half of the patients presented with previously treated HCCs, mainly associated to Hepatitis C (HCV), alcohol and NASH. Median tumor size was 16 (4-45) mm, 62.2% BCLC A. SIMWA was conducted with median 100 Watt for median 5 (1.5-18) minutes. Median lateral and longitudinal error of the needle placement were 3.2 (0.2-14.1) and 1.6 (0-15.8) mm. Median time for planning, navigation and validation were 10.5 (1.3-55.6), 7.1 (0.6-25.2) and 3.8 (0.1-19.6) minutes. At the time of intervention, median 1 tumor (1-4) was ablated per session. Within 30 days, 1 patient developed a Dindo grade IIIb (0.8%) complication, 3 patients showed minor complications. Median overall survival was 17.3 months after SIMWA and 23.5 months after initial diagnosis. BCLC stage, Child Pugh Class and previous treatment of HCC were significantly correlated with survival (p<0.05). Tumor size >= 3 cm, cirrhosis and development of new intrahepatic lesions were significantly correlated with local tumor recurrence (p<0.05). Twenty-two patients were transplanted after a median time of 10.7 (1.1-25.4) months, having undergone a median of one (1 - 4) SIMWA treatment. Analysis of 36 ablation zones in the explanted livers showed a complete pathological response in 22/36 (61.1%) treated lesions with a median rate of necrosis of 100% (15-100).

**Conclusion:**

SIMWA is very safe and efficient for the treatment of HCC offering a curative treatment approach especially for otherwise inoperable or conventionally unablatable lesions by accurate and precise needle positioning in a minimally invasive setting.

### In-hospital mortality and failure to rescue following hepatobiliary surgery in Germany: Does hospital volume matter?

(Abstract ID: 124)

C. Krautz^1^, C. Gall^1^, O. Gefeller^1^, U. Nimptsch^2^, T. Mansky^2^, G. F. Weber^1^, R. Grützmann^1^, S. Kersting^1^

^1^*Universitätsklinikum Erlangen*

^2^*Technische Universität Berlin*

**Background:**

Objective: We aimed to determine the unbiased mortality rates for hepatobiliaryresections at the national level using hospital discharge dataof every inpatient case in Germany. In addition, we intended to examinethe effect of hospital volume on in-hospital mortality, and failure to rescue.

Summary Background Data:Several studies have found strong volume-outcome relationships in high-risk surgery, with high mortality in low-volume facilities. However, there is a paucity of population-based outcome data on hepatobiliary surgery in European countries, including Germany.

**Materials and methods:**

Methods: We studied all inpatient cases of hepatobiliary surgery (n = 31,114) in Germany from 2009 to 2015, using national hospital discharge data. Minor resections and major resections were examined separately. We evaluated the association between hospital volume and in-hospital mortality following major hepatobiliary resections by using multivariate regression methods. In addition, we analyzed rates the failure to rescue across hospital volume categories.

**Results:**

Results: Minor hepatobiliary resections were associated with an overall mortality rate of 3.9% and no significant volume-outcome effects. In contrast,overall mortality rate of major hepatobiliary resections was 10.3%. In this cohort, risk-adjusted in-hospital mortality following major resections varied widely across hospital volume categories, from 7.4% (95% CI 6.6-8.2) in very high volume hospitals to 11.4% (95% CI 10.4-12.5) in very low volume hospitals (OR 0.59, 95% CI 0.41-0.54). Moreover, rates of failure to rescue were lower in higher volume hospitals(eg, mortality in patients with at least one complication in very high volume hospitals: 36.2% vs. very low volume hospitals: 40.6%).

**Table:** 

		Very Low (1-10)	Low (11-20)	Medium (21-40)	High (41-100)	Very High (>100)
Major Resections (Trisector-/Hemihepatectomy	Observed Mortality	11.6%	10.9%	10.4%	9.2%	9.9%
	Risk-adjusted Mortality (95%-CI)	11.4% (10.44-12.49)	10.1% (8.96-11.43)	9.2% (8.13-10-35)	8.0% (7.28-8.86)	7.4% (6.61-8.81)
Minor Resections (Multiple-/Bisegmentectomy)	Observed Mortality	4.5%	3.3%	3.0%	4.3%	3.4%
	Risk-adjusted Mortality (95%-CI)	4.5% (3.95-5.1)	3.3% (2.61-4.07)	3.3% (2.5-4.22)	4.0% (3.19-4.84)	3.3% (2.47-4.23)

Observed and Risk-adjusted Inhospital Mortality According to Hospital Volume Categories in Minor and Major Hepatobiliary Surgery.

**Conclusion:**

Conclusions: In Germany, patients who undergo major hepatobiliary resections have improved outcomes if they are admitted to higher volume hospitals. Volume-outcome associations are not present in minor hepatobiliary surgery.

**Picture:**
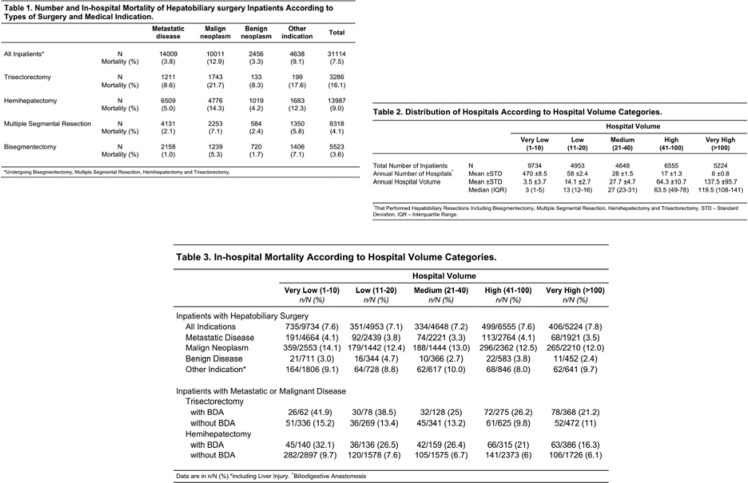


### Volume outcome relationship in pancreatic surgery – Effects of hospital and surgeon volume on mortality and postoperative pancreatic fistula

(Abstract ID: 131)

S. Acciuffi^1^, H. Ptok^1^, I. Gastinger^2^, R. S. Croner^1^, H. Dralle^3^

^1^*Universitätsklinikum Magdeburg*

^2^*Carl-Thiem-Klinikum Cottbus*

^3^*Universitätsklinikum Essen*

**Background:**

The volume effect in pancreatic surgery has gained increasing interest. Available Data for major pancreatic resections in Germany derived from hospital discharge data of every inpatient case lacking of relevant information like surgeon caseload and special surgical techniques.

**Materials and methods:**

In the framework of a prospective multicenter observational study data about pancreatic head resections from 27 German hospitals were collected from January 2006 to December 2009. We divided hospitals as well as surgeons into three volume groups. The aim was to analyze the effect of both hospital- and surgeon volume on postoperative outcome. Endpoints were mortality, length of hospital stay (LOS) and postoperative surgical complications with special regard to postoperative pancreatic fistula (POPF).

**Results:**

A total of 1.064 pancreatic head resection were performed. The overall postoperative mortality was 5.0% while the postoperative morbidity was 34.5%. There were no differences in mortality between the volume groups. Patients treated in high-volume hospitals showed a significant lower incidence of postoperative overall- (p=0.001) and specific surgical complications(p=0.021). Patients treated by high-volume surgeons had a significant lower incidence of POPF (p=0.001) and shorter LOS (p=0.007).

**Conclusion:**

Results support the request for centralization of pancreatic surgery and emphasize the importance of surgeon expertise.

### Percutaneous navigated CT-guided microwave ablation for malignant liver lesions

(Abstract ID: 136)

S. Perrodin^1^, A. Lachenmayer^1^, M. Maurer^1^, C. Kim-Fuchs^1^, D. Candinal^1^, V. Banz^1^

^1^*Universitätsspital Bern*

**Background:**

Thermal ablation has proven beneficial for hepatocellular carcinoma and, within clinical trials, for colorectal liver metastases (CRLM). Ablation could be an interesting option for other secondary liver malignancies. In addition, computer-assisted navigation techniques have been introduced to increase efficacy and broaden the indications for this minimally invasive approach. The aim of our study was to evaluate short-term clinical outcome of patients undergoing percutaneous stereotactic image-guided microwave ablation (SIMWA) for liver metastases (excluding CRLM).

**Materials and methods:**

Retrospective study including all patients undergoing SIMWA for non-CRLM liver metastases in our institution between January 2015 and December 2017. All patients were recommended for SIMWA according to a multidisciplinary tumorboard decision. Follow-up consisted of 3-monthly clinical and radiological (CT scan or MRI) check-ups, with additional oncological follow-up as needed. End-points included local recurrence rate, overall and liver-specific disease progression and post-interventional complications.

**Results:**

We included 23 patients, the majority were men (56.5%), with a mean age of 58.4 years. Twenty-five interventions were performed to treat 40 lesions. These included seventeen neuroendocrine and nine breast cancer metastases, four sarcomas, two non-small cell lung cancers, three duodenal adenocarcinomas, one esophageal adenocarcinoma, one pancreatic adenocarcinoma, one ampullary carcinoma, one prostate carcinoma, and one renal cell carcinoma metastases. Incomplete ablation rate was 2.5% (1/40) and local recurrence rate 10% (4/40). Three patients (12%) presented with minor complications, no major complications were observed. Median follow-up was 15 months (range 2-32). Overall disease progression occurred in 73.9% of patients with a median disease-free survival of 7 months (range 0-26). Overall survival was 18 months (range 2-39).

**Conclusion:**

SIMWA is a technically feasible, minimally invasive and safe treatment option for liver metastases of non-colorectal origin in selected patients. While it might offer an alternative to resection or a purely palliative strategy, the overall oncological benefit and effect on survival needs to be evaluated in a larger patient cohort.

### Successful use of the recanalized umbilical vein as a patch graft for venous reconstruction in abdominal surgery

(Abstract ID: 159)

B.-O. Stüben^1^

^1^*Universitätsklinikum Hamburg-Eppendorf*

**Background:**

Various approaches have been described for the reconstruction of the portal vein, superior mesenteric vein and the inferior vena cava. We present the use of the recanalized umbilical vein in various settings including transplantation, major liver resection and pancreatic surgery.

**Materials and methods:**

We retrospectively analyzed 4 cases, in which a recanalized umbilical vein was used for vascular reconstruction. The graft harvesting, size of the graft, technique of application, short-term results of vascular patency were studied.

**Results:**

A recanalized vein was successfully harvested in all patients with 5cm (median, range 3-7 cm) in length and 1.3 cm (median, range 1.0-1.8 cm) in width. The preparation of the recanalized umbilical vein was technically feasible and took no more than five minutes in each patient. All grafts were used as a patch for venous reconstruction. In 3 cases the graft was used for reconstruction of the portal vein and the superior mesenteric vein. In one patient, the graft was used to repair a large defect of the inferior vena cava. All vascular reconstructions were considered as successful as no bleeding or thrombosis was observed postoperatively.

**Conclusion:**

The recanalized umbilical vein is a reliable native autologous graft, which could be harvested in every patient with an intact ligament teres hepatis. We found that it is feasible to use this graft as a patch for the reconstruction of the inferior vena cava, the portal vein and the superior mesenteric vein.

### Chemotherapy and treatment at high-volume centers outperform extended lymphadenectomy in pancreatic cancer – a distinct view on lymph node yield

(Abstract ID: 178)

C. Tsai^1^, R. Warschkow^2^, N. Köhn^1^, S. Erdem^1^, B. Schmied^2^, D. Nussbaum^3^, B. Gloor^1^, D. Blazer III^3^, M. Worni^1^

^1^*Inselspital University Hospital of Bern*

^2^*Kantonsspital St. Gallen*

^3^*Duke University Medical Center, North Carolina*

**Background:**

While the importance of lymphadenectomy (LNE) is well established for patients with pancreatic cancer, the direct impact of LNE in relation to other predictive factors is ill-defined. We aimed to determine the relative effect of LNE on overall survival (OS).

**Materials and methods:**

The National Cancer Data Base from the United States from 2004-2014 was queried for patients with resected pancreatic adenocarcinoma (stage IA-IIB). Patients were dichotomized to lymph node (LN) yield of 1-14 or >=15 LNs based on prior studies on optimal LN surveillance. We performed Joinpoint regression to assess optimal LN yield, covariance-balanced propensity score analyses to assess LN yield as a continuous measure, and mediation analysis to measure the degree of effect of LNE.

**Results:**

A total of 22,910 patients were included, mean age was 65.5 years (SD: 10.5), 48.2% were female. Mean LN yield was 16.1 (SD: 9.3), 11,399 (49.8%) had 1-14 LN and 11,511 (50.2%) had >=15 LN retrieved. Likelihood of LN positivity increased by 3.9% per LN up 8 examined LN, and by 0.7% per LN above 8. Five-year OS was 16.8%. After multivariable adjustment, OS was better in the >=15 LNs group (HR 0.91, CI: 0.88-0.94, p<0.001). On a continuous scale, survival improved with increasing number of LNs collected, even when stratified by tumor stage. Mediation analysis revealed that LNE had 14.3% direct effect on improved OS, while 26.9% of the effect was due to treatment at high-volume hospitals and 31.3% due to chemotherapy.

**Conclusion:**

Higher LN yield leads to increased positive LN retrieval, which translates directly into survival benefit across all tumor stages. However, the direct effect on OS is more influenced by chemotherapy and treatment at high-volume centers than by LNE.

### Laparoscopic liver surgery – single center experiences from 350 consecutive cases

(Abstract ID: 198)

M. Schmelzle^1^, S. Wabitsch^1^, P. K. Haber^1^, F. Krenzien^1^, A. Kästner^1^, W. Schöning^1^, R. Öllinger^1^

^1^*Charité - Universitätsmedizin Berlin CVK*

**Background:**

In line with convincing results at international liver centers, the meaning of minimal-invasive techniques in complex liver surgery grew in Germany in recent years. Here, we report on our single center experiences with laparoscopic liver surgery.

**Materials and methods:**

We analyzed data of all consecutive patients undergoing laparoscopic liver surgery at the Department of Surgery, Charité - Universitätsmedizin Berlin between 02/2012 and 09/2018 with regard to patient characteristics, indications, complexity of procedures and postoperative outcomes.

**Results:**

In the last 6.5 years we performed 350 laparoscopic liver resections at our department. An annual increase of ∼ 20 operations allows us to carry out > 120 liver resections per year fully laparoscopically. After initial careful patient selection, the patients' health and performance status has significantly worsened over time and is now considered representative. Malignant tumors represent the most common indication for laparoscopic liver resection with hepatocellular carcinoma and colorectal liver metastases accounting for ∼ 60%. The percentage of complex procedures, e.g. resection of >= 3 segments or lesions in posterosuperior segments, increased significantly over time. Nevertheless, we were able to lower the rate of major complications (Dindo-Clavien > 3) below 13%.

**Conclusion:**

With growing experience less patient selection is evident in terms of patient characteristics, indications, and complexity of operations. Due to favorable postoperative results minimally invasive techniques have become firmly established even for complex procedures at our center.

### Laparoscopic liver surgery for cholangiocarcinoma – technical challenges

(Abstract ID: 199)

M. Schmelzle^1^, W. Schöning^1^, J. Pratschke^1^

^1^*Charité - Universitätsmedizin Berlin CVK*

**Background:**

Minimally invasive techniques have increasingly found their way into complex liver surgery in recent years. However, experience with laparoscopic hepatectomy for intrahepatic cholangiocarcinoma (iCC) is anecdotal to technical challenges, e.g. radical hilar lymphadenectomy.

**Materials and methods:**

Here we report on a 45-year old female patient diagnosed with a large intrahepatic cholangiocarcinoma (iCC) in the left liver lobe (diameter 10cm). We show a 4K video of an anatomical left hemihepatectomy (segments 2-4) with radical hilar lymphadectomy.

**Results:**

After exclusion of peritoneal spread, we performed an anatomical left hemihepatectomy in classical multiport technique. An ultrasound shear was used for superficial parenchym dissection. Deeper transection was performed under pringle maneuver using the waterjet technique. Both the left bile duct and the left vein were transected by using staplers. Radical hilar lymphadenectomy down the hepatic artery to the celiac trunk was performed fully laparoscopically according to established rules of open oncological surgery. No intraoperative transfusion of red blood cell concentrates was necessary. Operative time was 314 minutes. ICU stay was one day. Wound tubes could be removed on POD 2. No complications occurred postoperatively. The patient was discharged at POD 7. Microscopically tumor-free margins were confirmed histopathologically.

**Conclusion:**

We here present a video demonstrating our technique of fully laparoscopic left hemihepatectomy with radical hilar lympadencetomy for a iCC. Minimal-invasive hepatectomy should be considered a safe alternative to conventional open surgery even for iCC.

### Organization, function and gene expression of tertiary lymphoid structures in PDAC resembles lymphoid follicles in secondary lymphoid organs

(Abstract ID: 224)

M. Thelen^1^, T. Nestler^1^, S. Wagener-Ryczek^1^, J. Lehmann^1^, E. Staib^1^, F. Popp^1^, F. Gebauer^1^, P. Lohneis^1^, M. Odenthal^1^, S. Merkelbach-Bruse^1^, C. J. Bruns^1^, K. Wennhold^1^, M. S. von Bergwelt-Baildon^2^, H. A. Schlößer^1^

^1^*University of Cologne, Köln*

^2^*Uniklinik München*

**Background:**

Secondary lymphoid organs (SLO) are involved in induction and enhancement of anti-tumor immune responses on different tumor entities. Recent evidence suggests that anti-tumor immune responses may also be induced or enhanced in the tumor microenvironment in so called tertiary lymphoid structures (TLS). It is assumed that TLSrepresent a hotspot for T cell priming, B cell activation, and differentiation, leading to cellular and humoral anti-tumor immune response.

**Materials and methods:**

FFPE-slides of 50 primary PDAC patients were immunohistochemically (IHC) stained for CD20, CD3, CD8, AID, HLA-ABC and FoxP3 to analyze spatial distribution of tumor-infiltrating lymphocytes. 5-color immunofluorescence staining was performed to further investigate structural components of TLS in comparison to lymphoid follicles in SLOs. Microscope-based laser microdissection and Nanostring were used to compare gene expression in PDAC, TLS, SLOs and normal pancreatic tissue.

**Results:**

TLS were frequently detected in PDAC and were mainly localized along the invasive tumor margin. Results of TLS will be correlated with clinical parameters, immunoscore and immune escape mechanisms. 5-color Immunofluorescence staining revealed similar organization and function of TLS and SLO. Finally, gene expression analyzed by Nanostring revealed largely overlapping expression patterns in TLS and SLO.

**Conclusion:**

The results clearly demonstrate the close relationship of SLO and TLS regarding composition, function and gene expression.

### Laparoscopic versus open liver resection for benign tumors and lesions: A case matched study with propensity score matching

(Abstract ID: 257)

S. Wabitsch^1^, A. Kästner^1^, P. K. Haber^1^, A. Wabitsch^1^, C. Benzing^1^, F. Krenzien^1^, G. Atanasov^1^, C. Kamali^1^, A. Andreou^1^, W. Schöning^1^, R. Öllinger^1^, J. Pratschke^1^, M. Schmelzle^1^

^1^*Charité - Universitätsmedizin Berlin CVK*

**Background:**

In recent years, minimally invasive surgical approaches have gained an increasingly important role in hepatobiliary surgery. The aim of this study is to investigate the safety and potential benefits of laparoscopic liver resection (LLR) compared to open liver resection (OLR) for benign liver tumors and lesions.

**Materials and methods:**

Between January 2009 and December 2017, 182 patients underwent liver surgery for benign liver tumors and lesions in our center. After exclusion of 15 patients, the remaining 167 patients were divided into LLR group (n=54) and OLR group (n=113) and were compared with regard to perioperative outcomes. To overcome selection bias, a 1:1 propensity score matching (PSM) was performed. Additionally, patients undergoing major hepatectomy were divided into Major-LLR and Major-OLR groups and perioperative outcomes evaluated.

**Results:**

After PSM, 49 patients were included in the ORL group and 49 patients in the LLR group. The LLR group had a significantly shorter median intensive care unit (ICU) stay (LLR, 1d, 0-4d; OLR, 1d, 0-7d; p<0.001) and median hospital stay (LOS) (LLR, 7d, 4-14d; OLR, 9d, 5-58d; p<0.001). There were no significant differences in postoperative complications graded as Clavien-Dindo >=III (LLR, 10.2%; OLR, 4.1%; p=0.239) in both groups. Postoperative 90 days mortality rate was 0% in both groups. When comparing Major-LLR (n=8) with Major-OLR (n=59), patients in the Major-LLR group had a significantly longer median operation time (Major-LLR, 403 min., 240-501 min.; Major-OLR 221.5 min., 111-529 min.; p<0.001) and a significantly shorter median LOS (Major-LLR, 7d., 5-14d.; Major-OLR, 9d., 7-129d.; p=0.013). The rate of major complications (Dindo classification >=III) for Major-LLR was 0% and for Major-OLR 16.9% (p=0.207).

**Conclusion:**

Our case-matched study demonstrates shorter ICU and hospital stay using laparoscopic techniques while maintaining high-quality perioperative outcomes. Based on our findings, we suggest preferring the LLR over OLR for benign liver tumors and lesions regardless of the resection extent

### Features of nutritional support in patients with mild acute pancreatitis

(Abstract ID: 258)

S. Chuklin^1^, S. Chooklin^1^, G. Shershen^1^, P. Popyk^1^

^1^*Lviv Regional Clinical Hospital, Lwiw*

**Background:**

The number of patients with acute pancreatitis (AP) is increasing in the world.Approximately 85% of patients with AP have a mild course of the disease. Nutritional support isan important factor in the treatment of these patients. However, the optimal timetable forrestoring oral intake is almost not studied. The aim of this study was to determinate the possibility of early recovery of oral nutrition in patients with mild AP.

**Materials and methods:**

We examined 51 patients with mild acute pancreatitis. In 25 patients,an early oral refeeding (EORF) was used when patients experienced hunger, and 26 patientsreceived routine oral refeeding (RORF) after pain disappeared and normalized pancreaticenzymes serum levels.

**Results:**

Age, sexual, etiological and laboratory parameters, the severity of the condition ofpatients in the two groups during hospitalization were not statistically different. Before startingthe diet in the EORF group, serum concentrations of pancreatic amylase and lipase wereelevated. There was a significant difference in the duration of the hunger strike afterhospitalization between the EORF group and the RORF group. In addition, there was asignificant decrease in the total number of days of hospitalization in the EORF group comparedwith the group RORF. There were no differences in the relapse of abdominal pain, abdominaldistension, elevated serum levels of pancreatic enzymes, and severity of the condition of patientsbetween these two groups. All patients who developed relapse of pain and transient abdominaldistension did not require a change in nutrition regimen. The activity of inflammation at theconcentration of C-reactive protein significantly earlier was leveled in patients from the group ofEORF. All patients were discharged according to standardized criteria.

**Conclusion:**

In patients with mild AP, early onset of oral refeeding, which is safe, promotes afaster reduction of the inflammatory process, reduces the timing of hospitalization.

### CSI-MRI-assisted Evaluation of Fat Fraction in the Right and Left Lobe of the Liver before Liver Resection

(Abstract ID: 273)

M. Suchan^1^, G. Dieplinger^1^, T. Persigehl^1^, D. Zopfs^1^, D. L. Stippel^1^, C. J. Bruns^1^, R. Wahba^1^

^1^*Uniklinik Köln*

**Background:**

Steatosis hepatis could influence the outcome after liver resection. The standard diagnostic tool is still a liver biopsy, but fatty liver could also be quantified by imaging procedures such as ultrasound and CT and recently MRI. However, even liver biopsy can produce different results, so-called sample bias, due to localization of the sample. Therefore 'chemical shift imaging' (CSI) using MRI can assess different areas of the liver parenchyma to analyze the fat fraction (FF). The aim of this work is the evaluation and comparison of the FF in the right and left liver lobe (LL) by CSI-MRI in a surgical patient collective.

**Materials and methods:**

In this retrospective study 39 patients underwent major or minor liver resection (n = 21 vs. n = 18) were examined for the FF in CSI-MRI imaging (time period 2016 - 2017). The FF was measured separately in the right and left LL (Seg. V/VI vs. Seg. II/III) and evaluated by different possible CSI-MRI sequences (mDixon_Quandt, In-Phase/Opposed-Phase).

**Results:**

The mean FF of both LL was 4.21 % (n=39). In the right liver (Seg. V/VI) the FF was 3.86% vs 4.55 % in the left liver (Seg. II/III). The difference between the two LL is 0.69 %. Altogether a steatosis hepatis grade 1 (5 - 33 %) was shown in the right LL in 38.5 % (15/39) and in the left LL in 35.9 % (14/39). In 12.8 % (5/39) a different grade of steatosis hepatis was found for the respective LL. Excluding patients with HCC due to a pre-existing steatosis or fibrosis, the difference between right and left liver was 16.7 % .(4/24) of the examinations. In this group, 25% (6/24) of the patients had grade 1 fatty liver in both LL. Considering only patient with liver metastasis the difference in FF between right and left LL was 30.8% (4/13) and 7.7 % (1/13) had grade 1 fatty liver in the right and left LL. Mean serum bilirubin on post-op day 5 was 0.78 mg/dl and 0.85 mg/dl excluding HCC patients.

**Conclusion:**

CSI-MRT imaging shows differences in preoperative fat fraction between the right and the left liver lobe. These differences have an effect on the classification of the local steatosis hepatis. Localized preoperative FF measure by CSI-MRI adds information on the future liver remnant and volume possible to resect and should be further evaluated in liver resection planning.

**Picture:**
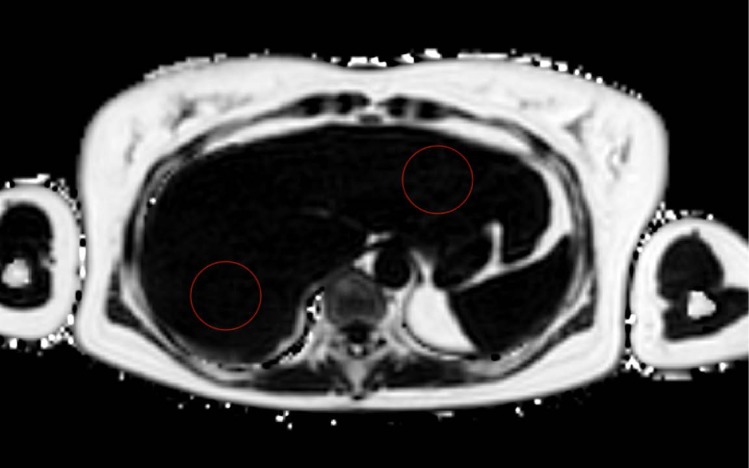
Example for the evaluation of the fat fraction using CSI-MRI (sequence: mDixon_Quandt, red circle: location of measurement)

### Prognostic impact of tumor-infiltrating leukocytes on survival of patients with primary resected pancreatic cancer

(Abstract ID: 274)

R. C. Miksch^1^, M. Weniger^1^, S. Ormanns^1^, J. Werner^1^, A. V. Bazhin^1^, J. G. D'Haese^1^

^1^*Uniklinik München*

**Background:**

In patients with pancreatic ductal adenocarcinoma (PDAC), the tumor microenvironment consists of cellular and stromal components that influence prognosis. Hence, tumor-infiltrating leukocytes (TILs) may predict prognosis more precisely than conventional staging systems. Studies on this impact of TILs are heterogeneous and further research is needed. Therefore, this study aims to point out the importance of peritumoral TILs and immune subtype classification in PDAC.

**Materials and methods:**

Material from 57 patients was analyzed with immunohistochemistry performed for CD3, CD8, CD20, CD66b, α-sma, and collagen. Hot spots with peritumoral TILs were quantified according to the QTiS algorithm and the distance of TILs hot spots to the tumor front was measured. Results were correlated with overall and progression-free survival.

**Results:**

High infiltration of peritumoral hot spots with CD3+, CD8+, and CD20+ TILs correlated significantly with improved overall (OS) and progression-free survival (PFS). Combined immune cell subtypes predicted improved OS, PFS. High infiltration of CD3+ TILs predict progression after 12 months. The location of TILs’ hot spots and their distance to the tumor front may play a role in patients’ survival.

**Conclusion:**

Peritumoral TILs and the stromal composition predict OS and PFS in PDAC.

### IRE is a safe and feasible treatment for unresectable local recurrence of pancreatic ductal adenocarcinoma

(Abstract ID: 280)

U. Heger^1^, C. Mack^1^, C. Tjaden^1^, C. Sommer^1^, S. Fritz^2^, O. Strobel^1^, M. W. Büchler^1^, T. Hackert^1^

^1^*Universitätsklinikum Heidelberg, Heidelberg*

^2^*Klinikum Stuttgart*

**Background:**

Irreversible electroporation (IRE) is emerging as treatment option for primary locally advanced pancreatic ductal adenocarcinoma (PDAC). Additionally, an increasing amount of data points towards efficacy of re-resection of local recurrence after resection of PDAC. We sought to investigate the feasibility and safety of IRE for the treatment of recurrence of PDAC.

**Materials and methods:**

We screened the records of patients prospectively followed in our single institution database who underwent IRE for locally unresectable either primary or recurrent PDAC. IRE was performed during surgical exploration for cases in which no metastases were present and the tumor was deemed locally unresectable. Endpoints were overall complication rate, death, reoperation/reintervention, pancreatitis, vascular complications (pseudoaneurysm, hepatic arterial thrombosis, and mesenteric/portal vein thrombosis), pancreatic fistula, ascites (>200 ml past pod3 or on imaging without fistula), biliary/hepatic complications, hemorrhage, intestinal perforations, SSI, timing of removal of surgical drains, length of ICU stay, length of hospital stay, emergency visits or readmission within 30 days.

**Results:**

5 (55,6 %) out of 9 patients suffered complications in the recurrence group as compared to 5 (55,6 %) out of 9 in the primary PDAC group (p=0); mortality was 0 in both groups. Complications were ascites (n=3; 33.3 %), delayed gastric emptying (n=3; 33.3 %), diarrhea (n=3; 33.3 %), pancreatitis (n=1; 11.1%); SSI (n=1; 11.1%), pneumonia (n=1; 11.1%) in the recurrence group; ascites (n=4; 44,4 %), delayed gastric emptying (n=1; 11.1%), sepsis (n=1; 11.1%); SSI (n=1; 11.1%) in the primary PDAC group. There was one intervention in the recurrence group, a CT guided drain placement für fluid collection.

**Conclusion:**

IRE for recurrence of PDAC was not more prone to intra-nor postoperative complications than in primary PDAC and can be considered a novel viable option for the treatment of local recurrence of PDAC. Mid- and long-term follow up of patients as well as randomized treatment studies are required to establish the definitive role of IRE in recurrence of PDAC.

### Prevention of postoperative bile-leakage by a newly designed tissue sealant patch

(Abstract ID: 283)

A. Heumann^1^, A. Bahar^1^, M. Touren^2^, H. de Vries^2^, J. Izbicki^1^, M. Bockhorn^1^

^1^*Universitätsklinikum Hamburg-Eppendorf*

^2^*Polyganics BV, Groningen*

**Background:**

Up to 10-15% of the patients develop a bile-leakage after liver surgery, which increases the length of hospital stay and overall mortality and morbidity. However, with no effective preventive measure at hand, the surgical community is forced to accept bile-leakage as an unavoidable post-surgery complication. To overcome this clinical challenge we developed a new tissue sealant patch to prevent postoperative bile-leakage.

**Materials and methods:**

The tissue sealant patch was developed in a multi angle approach including in-vitro comparison on tensile, burst pressure measurements and testing in a liver perfusion model with clinically relevant competitors. For in-vivo evaluation a porcine bile-leakage model was established and the tissue sealant patch was investigated in a prospective randomized animal trial with a suturing group and Veriset^®^ group as controls.

**Results:**

More than 30 different prototypes were screened in-vitro. The final selected sealant prototype showed superiority compared to clinical used competitors Tachosil, Hemopatch and Veriset in tensile and burst pressure testing (p<0.05 each). Moreover, the newly developed patch reduced the leakage rate in the liver perfusion model (p<0.05). The pre-clinical performance of the sealant patch was confirmed in a porcine bile-leakage model. 21 animals were included in the study and randomized for treatment with the sealant, Verisetäor suturing (n=7 each). After 7 days incidence of bile leakage was significantly lower in the sealant group compared to the Veriset group (p<0.05) and comparable to the suturing control group. These promising results were supported by strong bile containment and the formation of a smooth fibrous capsule by the sealant within one week. This was paralleled by the formation of neo bile ducts. Furthermore, no systemic or local side effects (e.g. bilioma) were seen.

**Conclusion:**

The new designed sealant was as effective as suturing in preventing bile-leakage in our animal model. This was due to strong bile containment and formation of a fibrous capsule by the sealant within one week. The efficacy of the sealant was also histologically proven, as formation of neo bile ducts - which indicates a biliary obstruction - was detected. More importantly, no clinical relevant side effect of the sealant became evident. To our knowledge, this is the first report of a randomized trial showing the efficacy of a tissue sealant device for preventing postoperative bile leakage.

### Surgeon versus Pathologist: Fibrosis at the Cut Margin Outperforms Pancreatic Texture as a Predictor of Postoperative Pancreatic Fistula in Pancreatoduodenectomy – a retrospective Analysis of the RECOPANC Trial

(Abstract ID: 300)

E. Petrova^1^, S. Timme^2^, M. Werner^2^, T. Keck^1^, P. Bronsert^2^, U. F. Wellner^1^

^1^*Uniklinik Lübeck*

^2^*Universitätsklinikum Freiburg i.Br., Freiburg i.Br.*

**Background:**

Postoperative pancreatic fistula (POPF) is the Achilles heel of pancreatic surgery. Pancreatic texture, as assessed by the surgeon, has been identified as the strongest predictor of POPF in many studies. However, texture is a subjective parameter with no proven reliability or internal or external validity. Therefore a more objective parameter is needed for exact risk stratification in pancreatic surgery. The aim was to evaluate fibrosis at the pancreatic cut margin as an alternative parameter.

**Materials and methods:**

The RECOPANC trial was conducted as a monitored multicenter prospective trial. Pancreatic fibrosis was assessed retrospectively from H&E stained tissue slides of the pancreatic cut margin collected centrally during conduct of the RECOPANC trial. Fibrosis was graded from 0 (no fibrosis) to III (severe fibrosis). Predictive value of fibrosis grade and pancreatic texture with regard to POPF of grade B/C was assessed by univariable and multivariable statistical modeling in R software.

**Results:**

Fibrosis grading showed strong interrater reliability (kappa=0.74) and correlated positively with hard pancreatic texture (p<0.05). In univariable analysis, area under the curve (AUC) for the prediction of POPF B/C was higher for fibrosis grade than for pancreatic texture (0.71 vs 0.59). In multivariable analysis, the following predictors were selected by elastic net regression: sex, surgeon volume, main pancreatic duct diameter and fibrosis. The final multivariable model reached an AUC of 0.78 with PPV and NPV of 0.38 and 0.92.

**Conclusion:**

Pancreatic fibrosis grade at pancreatic cut margin can substitute assessment of pancreatic texture and is a more objective and reliable parameter. Future studies might use fibrosis grade for risk stratification in pancreatic surgery.

### Prospective trial to evaluate the prognostic value of different nutritional assessment scores in liver surgery: NURIMAS Liver (DRKS00006340)

(Abstract ID: 316)

P. Probst^1^, J. Fischer^1^, M. R. Schoen^2^, G. Polychronidis^1^, C. Stravodimos^2^, A. Mehrabi^1^, M. K. Diener^1^, M. W. Büchler^1^, P. Knebel^1^, K. Hoffmann^1^

^1^*University of Heidelberg*

^2^*Municipal Hospital Karlsruhe*

**Background:**

Malnutrition is recognized as a preoperative risk factor for patients undergoing hepatic resection. To take preventive therapeutic actions before surgery, it is important to identify malnourished patients. However, there is no evidence, which existing nutritional assessment score (NAS) is suited best to predict the postoperative outcome in liver surgery.

**Materials and methods:**

All patients scheduled for elective liver resection at the surgical department of the University Hospital in Heidelberg and the municipal hospital of Karlsruhe were screened for eligibility. Before surgery, every patient was assessed to be at risk for malnutrition or not according to Nutritional Risk Index, Nutritional Risk Screening original and 2002, Subjective Global Assessment, Malnutrition Universal Screening Tool, Mini Nutritional Assessment original and SF, Short Nutritional Assessment Questionnaire, Imperial Nutritional Screening System I+II, Nutritional Risk Classification and the ESPEN malnutrition criteria. Throughout the patient’s hospital stay, postoperative morbidity and mortality was tracked prospectively. The association of malnutrition according to each score and occurrence of at least one major complication was the primary endpoint, using a multivariable logistic regression analysis including established risk factors in liver surgery as covariates.

**Results:**

The population consisted of 182 patients. The percentage of patients labelled as malnourished by the NAS varied among the different scores, with the lowest being at 2.2% (Mini Nutritional Assessment) and the highest at 52.2% (Nutritional Risk Classification). In 40 patients (22.0%) a major complication was observed. None of the scores showed a significant association with the occurrence of major complications in the multivariable analysis.

**Conclusion:**

None of the twelve NAS investigated defined a state of malnutrition which was independently associated with postoperative complications. Other measures to determine malnutrition in liver surgery should be investigated prospectively.

### A large multicenter cohort analysis of more than 200 patients undergoing pancreatoduodenectomy for ampullary cancer

(Abstract ID: 323)

L. Bolm^1^, K. Ohrner^1^, F. Rückert^2^, B. M. Rau^3^, E. Petrova^1^, D. Bausch^1^, J. Weitz^4^, T. Keck^1^, U. F. Wellner^1^, M. Distler^4^

^1^*Uniklinik Lübeck*

^2^*Universitätsmedizin Mannheim*

^3^*Klinikum Neumarkt*

^4^*Universitätsklinikum Technische Universität Dresden*

**Background:**

Ampullary cancer (AMPCA) is a rare gastro-intestinal malignancy. We aimed to evaluate long-term overall survival and prognostic factors after pancreatoduodenectomy (PD) in a large multicenter cohort.

**Materials and methods:**

Patients undergoing PD for AMPCA at 4 high-volume surgical centers from 1996 to 2017 were identified from prospectively maintained databases. Patient baseline characteristics, surgical and histopathological parameters, as well as long-term overall survival after resection were evaluated.

**Results:**

A total of 216 patients undergoing PD for ampullary cancer were included. 19.9% had a classical Whipple procedure, 79.2% underwent pylorus-preserving PD and a total pancreatectomy was performed in 0.9%. Median serum CEA was 1.9 (0.0-224.6) while median serum CA 19-9 was 33.85 (0.0-98726.0). 1.4% of the patients underwent neoadjuvant therapy. 47.7% presented with T3-4 tumors, 46.8% were diagnosed N1, and 12.0% had metastatic disease. Positive resection margins were confirmed in 5.1% of the patients. Median overall survival in all patients was 23 months. In univariate survival analysis, CEA (HR 2.239, 95%CI 1.321-3.793, p=0.003), CA 19-9 (HR 1.939, 95%CI 1.245-3.018, p=0.003), multivisceral resection (HR 4.081, 95%CI 0.985-16.906, p=0.052), T stage (HR 3.447, 95%CI 2.193-5.417, p<0.001), N stage (HR 0.023, 95%CI 0.003-0.189, p<0.001), grading (HR 1.656, 95%CI 1.086-2.525, p=0.019) and R status (HR 0.175, 95%CI 0.083-0.368, p<0.001) qualified as prognostic parameters. In multivariate analysis, T stage (HR 2.260, 95%CI 1.312-3.891, p=0.003) and R status (HR 0.338, 95%CI 0.133-0.859, p=0.023) remained independent prognostic factors.

**Conclusion:**

T stage and R status were the strongest prognostic factors for long-term overall-survival in AMPCA patients undergoing PD. Consequently, curative resection is warranted in patients with localized disease and careful preoperative evaluation of resectability should be performed.

### Preoperative biliary stenting is associated with higher postoperative morbidity and equivalent mortality in pancreatic cancer patients with hyperbilirubinemia – an analysis of the German DGAV StuDoQlPancreas registry

(Abstract ID: 326)

L. Bolm^1^, E. Petrova^1^, L. Woehrmann^1^, J. Werner^2^, W. Uhl^3^, N. Nuessler^4^, M. Ghadimi^5^, D. Bausch^1^, H. Lapshyn^1^, J. Gaedcke^5^, O. Belyaev^3^, J. G. D'Haese^2^, T. Klier^4^, T. Keck^1^, U. F. Wellner^1^

^1^*Uniklinik Lübeck*

^2^*Uniklinik München*

^3^*St. Josef Hospital, Ruhr Universität Bochum*

^4^*Klinikum Bogenhausen, München*

^5^*Universitätsmedizin Göttingen*

**Background:**

The impact of preoperative biliary stenting (PBS) prior to pancreatoduodenectomy (PD) for pancreatic ductal adenocarcinoma (PDAC) in patients with hyperbilirubinemia is controversial.

**Materials and methods:**

Patients undergoing PD in the time period from 2014 to 2016 with or without PBS for PDAC were identified from the German DGAV-StuDoQlPancreas registry. The effects of PBS in patients with and without a history of jaundice were evaluated. Furthermore, the impact of different levels of hyperbilirubinemia and subsequent PBS on postoperative morbidity and mortality were analyzed.

**Results:**

1133 patients undergoing PD for PDAC were identified from the registry, and 480 patients underwent PBS. 320 patients receiving PBS (66%) had no history of jaundice. In these patients, PBS was associated with higher rates of preoperative cholangio-sepsis (PBS 6% vs. no PBS 2%, p<0.001), grade B/C postoperative pancreatic fistula (POPF) (PBS 18% vs. no PBS 12%, p=0.053), and surgical site infections (SSI) (PBS 20% vs. no PBS 11%, p=0.001). PBS was not associated with higher postoperative morbidity in patients with a history of jaundice. Serum bilirubin levels of 15 mg/dl and higher lead to more CDC grade IIIa-IVb (24% vs. 28%, p=0.053) and higher mortality (3% vs. 7%, p<0.001). PBS in patients with serum bilirubin levels of >15 mg/dl increased grade CDC IIa-IVb complications (21% vs. 50%, p=0.001), and equivalent mortality was observed.

**Conclusion:**

The majority of PBS procedures were performed in patients with no history of jaundice and lead to a significant increase in perioperative morbidity. Serum bilirubin levels > 15mg/dl were associated with higher postoperative morbidity and mortality. PBS correlated with higher postoperative complication rates in these patients.

### Activated Stroma Index in neoadjuvantly treated patients with pancreatic ductal adenocarcinoma

(Abstract ID: 352)

A. Martens^1^, U. Heger^1^, B. Walter^1^, H. Sun^1^, S. Roth^1^, C. W. Michalski^2^, M. W. Büchler^1^, T. Hackert^1^

^1^*Universitätsklinikum Heidelberg*

^2^*Universitätsklinikum Halle (Saale)*

**Background:**

Pancreatic ductal adenocarcinoma (PDAC) is characterized by an extensive desmoplastic reaction. Pancreatic stellate cells, which are part of the normal pancreatic stroma, are activated by tumor cells and in their activated state they are the main source of collagen deposition within the tumor. The Activated Stroma Index (ASI) is the ratio of activated pancreatic stellate cells (PSCs) to collagen deposition. It has been previously described as a prognostic marker in PDAC. The usefulness of the ASI as a prognostic marker in patients after neoadjuvant chemotherapy with FOLFIRINOX has not been described before.

**Materials and methods:**

31 patients who underwent surgery after neoadjuvant chemotherapy following the FOLFIRINOX protocol for PDAC were analyzed by immunohistochemistry. α-smooth muscle antigen antibodies were used as a specific marker for activated PSCs. Anilinblue was used for collagen staining. The whole tissue sample was digitalized and the stained area was determined using computational imaging analysis. Analysis of overall survival was performed by Kaplan-Meier method.

**Results:**

The lowest quartile of ASI values (<0,322) was associated with improved overall survival (p=0,052). Patients with ASI below 0,322 had a mean overall survival of 32,7 months (95% confidence interval (CI) 27,7 - 37,6), whereas patients above had a mean overall survival of 24,1 months (95% CI 19,1 - 29,1). Patients within the highest quartile of collagen deposition values (> 42,5% of the total tissue sample area) had a mean overall survival of 32,7 months (95% CI 28,5 - 36,9), whereas patients below 42,5% had a mean overall survival of 24,9 months (95% CI 19,2 - 30,5) (p=0,072). The activity of PSCs alone did not correlate with overall survival.

**Conclusion:**

These data suggest that the ASI as well as the amount of collagen deposition could be useful prognostic markers for patients after FOLFIRINOX and resection, as survival curves showed a distinct trend in spite of remaining just below statistical significance. Main limitations are the small patient cohort (n=31) and the relatively short follow up time (mean=26 months). Potentially both markers could be useful for the establishment of response grading after neoadjuvant chemotherapy in pancreatic cancer and should therefore be further investigated.

**Picture:**
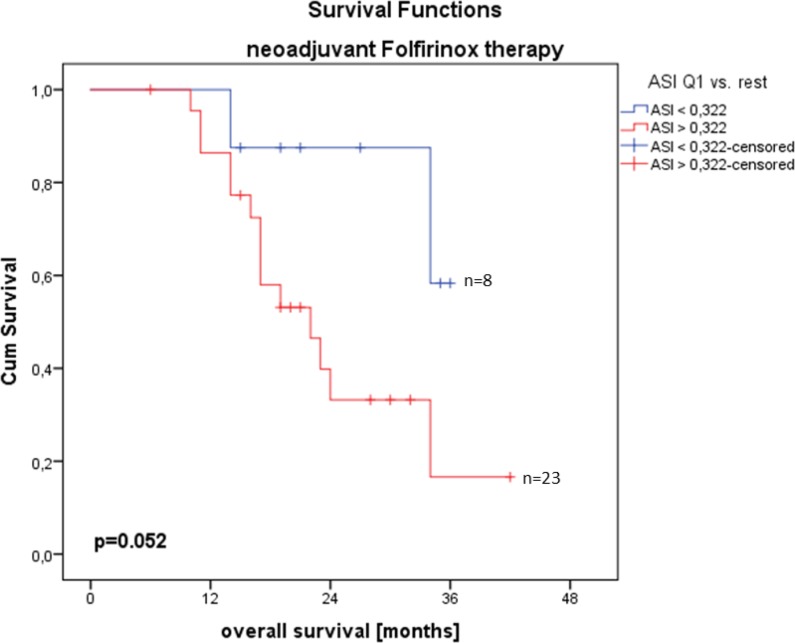
Correlation of ASI and overall survival of patients

### Relevant prognostic factors influencing outcome of patients after surgical resection of distal cholangiocarcinoma

(Abstract ID: 373)

O. Beetz^1^, M. Klein^1^, H. Schrem^1^, J. Gwiasda^1^, F. Vondran^1^, F. Oldhafer^1^, S. Cammann^1^, J. Klempnauer^1^, K. Oldhafer^2^, M. Kleine^1^

^1^*Medizinische Hochschule Hannover*

^2^*Asklepios Klinik Barmbek, Hamburg*

**Background:**

Distal cholangiocarcinoma (DCC) is a rare but over the last decade increasing malignancy and is associated with poor prognosis. According to the present knowledge curative surgery is the only chance for long term survival. This study was performed to evaluate prognostic factors for the outcome of patients undergoing curative surgery for distal cholangiocarcinoma.

**Materials and methods:**

75 patients who underwent surgery between January 2000 and December 2014 for DCC in curative intention were analysed retrospectively. Potential prognostic factors for survival were investigated including the extent of surgery using purposeful selection of covariates in multivariable Cox regression modeling.

**Results:**

Preoperative biliary stenting (Hazard ratio (HR): 2.530; 95%-CI: 1.146-6.464, p = 0.020), the extent of surgery in case of positive histological venous invasion (HR: 1.209; 95%-CI: 1.017-1.410, p = 0.032), lymph node staging (HR: 2.183; 95%-CI: 1.250-3.841, p = 0.006), perineural invasion (HR: 2.118; 95%-CI: 1.147-4.054, p = 0.016) and postoperative complications graded in points according to Clavien-Dindo (HR: 1.395; 95%-CI: 1.148-1.699, p = 0.001) were indentified as independent significant risk factors for survival. Patients receiving preoperative biliary stenting showed prolonged duration between onset of symptoms and date of operation (p = 0.048).

**Conclusion:**

Preoperative biliary stenting reduces survival possibly due to delayed surgery. The extent of surgery is not an independent risk factor for survival except for patients with concomitant histological venous invasion. Oncological factors and postoperative surgical complications are independent prognostic factors for survival.

BMC Surgery (2018 Aug 13;18(1):56. doi: 10.1186/s12893-018-0384-5)

### Influence of the Body Mass Index on postoperative outcome and long-term survival after pancreatic resections in 1692 Patients with underlying malignancy

(Abstract ID: 375)

P. Seika^1^, F. Klein^1^, M. Bahra^1^, J. Pratschke^1^, T. Malinka^1^

^1^*Charité - Universitätsmedizin Berlin CVK*

**Background:**

While the long-term survival rate among patients with periampullary and pancreatic carcinomas remains low, it can be influenced by various factors. The purpose of this retrospective study was to quantify the effects of body mass on postoperative complications and patient survival after pancreatic resections for underlying malignancy over a 20-year observation period.

**Materials and methods:**

We studied 1692 patients, 1221 patients with adenocarcinoma of the pancreas (72.2%), 205 ampullary carcinoma patients (12.1%), 235 patients with carcinoma of the lower common bile duct (13.8%) and 31 duodenal carcinoma patients (1,9%). The patients were classified into four groups (Group 1 <18.5, Group 2, 15.5-25, Group 3 25-30, Group 4 >30) according to their body mass index (BMI; kg/m2). We analyzed differences in postoperative complications, hospitalization duration, mortality, reoperations and survival rate among the groups.

**Results:**

Within a mean observation period of 773.8 (2-8500) days, 871 (64.8%) patients died. There were important differences in postoperative complications (group 1, 18,6%; group 2, 22.5%; group 3, 28.4%, group 4, 39,7%) with the type of postoperative complications also varying between the groups. The overall 1-, 5-, 10- and 15-year survival rates were 64.57%, 24.09%, 16.77%, and 12.49%, respectively with survival rates varying amongst the four groups.

**Conclusion:**

Patients with a BMI between 18.5 and 25 show better postoperative outcomes, regarding complications, hospitalization duration, and reoperation, than underweight or obese patients. Short-term survival depends strongly on postoperative complications while patients with a higher BMI show better long-term survival rates.

### Laparoscopic resection of lesions located in the posterosuperior segments of the cirrhotic liver: a single institution analysis

(Abstract ID: 376)

P. Haber^1^, S. Wabitsch^1^, F. Krenzien^1^, C. Benzing^1^, A. Andreou^1^, W. Schöning^1^, R. Öllinger^1^, J. Pratschke^1^, M. Schmelzle^1^

^1^*Charité - Universitätsmedizin Berlin CVK*

**Background:**

Lesions in the posterosuperior liver segments (IVa, VII, VIII) have been described as the most challenging to address laparoscopically. While some studies have compared minimal invasively to conventional open approaches, virtually no attention has been given to distinguish between outcomes for patients with and without cirrhosis.

**Materials and methods:**

All consecutive patients undergoing liver resection at the Department of Surgery, Charité - Universitätsmedizin Berlin for at least one lesion in the posterosuperior segments between January 2014 and July 2018 were retrospectively analyzed. Based on the presence (n=43) or absence (n=115) of liver cirrhosis, patients were divided in two groups.

**Results:**

Evaluation of preoperative patient characteristics revealed patients with cirrhosis to be older (p<0.001) and with a higher likelihood for a history of diabetes (p<0.005) and alcohol consumption (p<0.0005). With regard to preoperative liver function, as assessed by LiMAx, cirrhotic patients had a markedly poorer score (p<0.005).

While a similar percentage in both groups had anatomical resection, patients in the no cirrhosis group had markedly less major resections (p<0.0005). Surgeries were, as a result, markedly longer in the no cirrhosis group (p<0.0005). With regard to the need for conversion and perioperative transfusion rate, no significant difference was noted between the two groups.

Analysis of the postoperative course showed similar outcomes concerning ICU- (cirrhosis: 1 day vs. no cirrhosis: 1 day) and hospital stays (cirrhosis: 8 days vs. no cirrhosis: 9 days). In keeping with this, complication rate was similar as well, independent of whether all or only major complications were taken into consideration (all: cirrhosis: 27.9% vs. no cirrhosis: 24.3%; >= Grade III: cirrhosis: 9.3% vs. no cirrhosis 19.1%; p=0.157). Resection margins were similar between the two groups (R0: Cirrhosis: 83.8% vs. no cirrhosis: 86.1%).

**Conclusion:**

Our data shows that the beneficial results conveyed through minimal invasive techniques also apply for cirrhotic patients with a lesion in the posterosuperior liver segments. No significant differences with regard to both safety and oncologic sufficiency was observed, when compared to non-cirrhosis. As these procedures entail a high degree of difficulty from a technical perspective, they should be performed in specialized centers.

### Extended left versus extended right hepatectomy with hilar en-bloc resection in perihilar cholangiocarcinoma

(Abstract ID: 420)

J. Bednarsch^1^, Z. Czigany^1^, I. Amygdalos^1^, D. A. Morales Santana^1^, F. Meister^1^, J. Böcker^1^, C. Roderburg^1^, N. Gaisa^1^, U. P. Neumann^1^, G. Lurje^1^

^1^*Uniklinik RWTH Aachen*

**Background:**

Hilar en-bloc resection with portal vein resection (PVR) has emerged as the mainstay of treatment for patients with perihilar cholangiocarcinoma (PHCC). Whether liver resection should be carried out as extended left-(LH) or right-sided hepatectomy (RH) is still subject of ongoing debate. Here we evaluated perioperative complications and oncological outcome after RH or LH with hilar en-bloc resection and PVR in patients with PHCC.

**Materials and methods:**

Between 2010 and 2016, 91 patients with PHCC underwent surgery in curative intent at our institution. Perioperative and survival data from all patients undergoing surgical resection for PHCC were analyzed. PVR was carried out in all cases as well as arterial reconstruction (n=5) if necessary. Patients undergoing hepatoduodenectomy (n=8) or ALPPS (n=2) were excluded from the analysis.

**Results:**

Tumor grading, microvascular invasion, lymphovascular invasion, N-category, T-category, R-status and UICC tumor staging were equally distributed among the LH (n=36) and RH (n=45) groups. Perioperative morbidity and mortality were higher after RH compared to LH (15.6% vs. 8.3%, p=0.003). While 3-year OS was comparable between LH and RH (55% vs. 48%), we observed a non-significant difference in 5-year OS with 18% and 43% for LH and RH respectively (p=0.820, log rank).

**Conclusion:**

LH and RH hilar en-bloc resections demonstrate comparable 3-year OS. While RH hilar en-bloc resection might result in better long-term 5-year survival, this may be at cost of an increase in perioperative morbidity and mortality.

### Lymphovascular invasion and surgical complications predict clinical outcome in patients with perihilar and intrahepatic cholangiocarcinoma

(Abstract ID: 422)

J. Bednarsch^1^, Z. Czigany^1^, I. Amygdalos^1^, D. A. Morales Santana^1^, F. Meister^1^, J. Böcker^1^, C. Roderburg^1^, N. Gaisa^1^, U. P. Neumann^1^, G. Lurje^1^

^1^*Uniklinik RWTH Aachen*

**Background:**

Cholangiocarcinoma (CCC) is a relatively rare malignancy that is typically diagnosed at an advanced disease stage. Major liver resection with portal vein reconstruction has evolved as the mainstay of treatment for patients with perihilar (PHCC) and intrahepatic cholangiocarcinoma (IHCC). Despite recent advancements, the overall-(OS) and recurrence-free survival (RFS) in CCC remains lower than for most other solid tumors. Here we aimed to identify prognostic markers of clinical outcome in CCC-patients that underwent surgical resection in curative intent.

**Materials and methods:**

Between 2010 and 2016, 162 patients with CCC (PHCC: n=91, IHCC; n=71) underwent surgery in curative intent at our institution. Preoperative characteristics, perioperative data and oncological follow-up were obtained from a prospectively managed institutional database. The associations of RFS and OS with clinico-pathological characteristics were assessed using univariate and multivariate survival analyses.

**Results:**

The median OS and RFS were 38 and 36 months for PHCC and 25 and 13 months for IHCC, respectively. Lymphovascular invasion (LVI) as well as surgical complications as assessed by the comprehensive complication index were independently associated with OS for the PHCC (LVI; Exp(B)=2.28, p=0.042; CCI; Exp(B)=1.04, p<0.001) and IHCC cohorts (LVI, Exp(B)=5.08, p=0.028; CCI, Exp(B)=1.04, p=0.002), respectively. No other clinical variable including R0-status and Bismuth classification was associated with OS.

**Conclusion:**

Surgical resections for CCC are safe in experienced high-volume liver centers. Tumor and patient characteristics were not associated with clinical outcome. In patients with PHCC and IHCC, LVI and CCI are associated with OS, suggesting a similar tumor biology.

### Liver resection for intrahepatic cholangiocarcinoma: Biological and surgical predictors of outcome, Status Quo in additive therapy

(Abstract ID: 454)

A. Nickkholgh^1^, O. Ghamarnejad^1^, E. Khajeh^1^, P. Fathi^1^, B. Göppert^1^, A. Mehrabi^1^

^1^*Universitätsklinikum Heidelberg*

**Background:**

Liver resection constitutes the only therapeutic option for the intrahepatic cholangiocarcinoma (IHCCA). The aim of this work was to analyze the outcome of liver resection for patients with IHCCA, and to revisit the biological and surgical determinants of outcome, and the role of neoadjuvant and additive therapeutic modalities in our single-center cohort of patients during the last decade.

**Materials and methods:**

Using a prospectively filled database of all consecutive patients undergoing surgery due to a preoperative diagnosis of ICD-code C22.1 between December 2001 and December 2015 at the Department of General, Visceral, and Transplantation Surgery at the University of Heidelberg. Demographic, anatomical, clinical, operative, surgical pathologic and follow-up data of all patients with a final diagnosis of IHCCA were analyzed.

**Results:**

A final surgical pathologic diagnosis of IHCCA was made for 190 patients. Nineteen patients (10.2%) had undergone neoadjuvant chemotherapy. The most frequent surgical approach entailed minor hepatectomy (<= 3 segments) in 49 (25.7%), including three patients undergoing central liver resection (for Couinaud liver segments 4, 5, and 8), followed by left hemihepatectomy in 45 patients (23.7%). Locoregional lymphadenectomy was performed in 91 (48.1%) of patients. Free surgical margin was achieved in 117 patients (64.6%). The most frequent postoperative complications necessitating non-operative interventions were grade B biliary complications in 46 patients (24.2%). Median hospital stay was 14 days (range 2-92 days). The 1-, 3-, and 5-year overal survival (OS) were 75%, 57%, and 38%, respectively. By the time this study ended, recurrence was documented in 87 patients (10 ± 1 months). The mean survival time after the documentation of recurrence was 16 ± 2 months. According to the multivariate cox regression analysis, age >= 65 years (hazard ratio [HR] 2.2, 95% confidence interval [CI] 1.2-4.0, p=0.013), median tumor diameter of >= 5 cm (HR 3.0, 95% CI 1.4-6.2, p=0.004), preoperative biliary drainage (HR 2.7, 95% CI 1.2-6.4, p=0.021), and local R status (HR 2.0, 95% CI 1.1-3.7, p=0.034) were the independent determinants of OS. Furthermore, median tumor diameter of >= 5 cm (HR 1.7, 95% CI 1.1-2.7, p=0.020), high-grade (G3-G4) tumor (HR 1.6, 95% CI 1.0-2.5, p=0.034), local status R1 (HR 1.7, 95% CI 1.1-2.7, p=0.002) were the independent determinants of disease-free survival.

**Conclusion:**

Hepatectomy remains the only curative treatment for patients with IHCCA. Additive therapeutic strategies to prolong disease-free survival are still ineffective. Further, prospective studies are needed to improve the postoperative outcomes of IHCCA.

### Increased incidence of hepatic alveolar echinococcosis in immunocompromised patients – a monocentric data analysis of 136 patients revealing a significant correlation with survival

(Abstract ID: 468)

A. Lachenmayer^1^, D. Gebbers^1^, D. Candinas^1^, G. Beldi^1^

^1^*Universitätsspital Bern*

**Background:**

Alveolar echinococcosis (AE) is a zoonosis mostly infesting in the human liver and often showing a tumor-like growth pattern. The increase of infected rural and urban fox populations and the extended routine use of imaging in the daily clinical practice have been associated with the growing incidence and the increasing diagnoses of human AE, respectively, in endemic regions including Switzerland. Limited data now suggests that immunocompromised (IC) patients might be more susceptible to the infection and develop a more severe course of the disease. Therefore, we aimed to analyze the incidence of immune system modulating events in form of autoimmune diseases, intake of immunosuppressants and malignancies in our own patient cohort.

**Materials and methods:**

Retrospective data analysis of 136 patients treated for AE by either surgery or conservative treatment between 1971 and 2017 at the Department of Visceral Surgery and Medicine of the University Hospital Bern, Switzerland.

**Results:**

Our cohort consisted of 66 (48.5%) males and 70 (51.5%) females with a median age at diagnosis of 57 (16-88) years. Eighty-four (61.8%) patients received curative resections, 45 (33.1%) patients were treated with benzamidazole only due to inoperable disease and 7 (5.1%) patients had palliative surgeries in the beginning of the time frame analyzed. Interestingly, 46 (33.8%) patients had immune system modulating events in their medical history: 14 (10.3%) patients had immunosuppressive diseases including several different rheumatological autoimmune diseases, 3 (2.2%) were immunosuppressed medically post liver and kidney transplantation and 14 (10.3%) patients had malignancies including melanoma (n=3), lymphoma (n=2), liposarcoma (n=1), urothel- (n=1), breast- (n=3), skin- (n=2), colorectal- (n=1) and thyroid- (n=1) cancer. In 17 (12.5%) patients we detected other immunocompromising events in their medical history such as receiving long time steroids, or having tuberculosis or asthma earlier in their lives. Eighteen (13.2%) patients had significant risk factors documented including working as a farmer, hunter or veterinarian. No significant correlation was detected with tumor size or EM18, EM2 or EgHF levels. During the past decade we observed a significant increase (p<0.05) of immune-system modulating conditions associated to new diagnoses of AE. The overall survival of IC patients was significantly worse (p=0.01) compared with non-IC patients.

**Conclusion:**

The incidence of hepatic AE is increasing significantly, in particular in IC patients with a significant correlation with survival. Although we believe that IC patients in endemic regions should be advised about their increased risk of infection, the exact surveillance modalities and the role of serologic markers still need to be defined.

### Delayed gastric emptying does not influence cancer specific survival after pancreatoduodenectomy for pancreatic adenocarcinoma

(Abstract ID: 479)

J. Hafke^1^, S. Manekeller^1^, J. C. Kalff^1^, T. R. Glowka^1^

^1^*Universitätsklinikum Bonn*

**Background:**

Several studies demonstrate a negative impact of postoperative complications on cancer-specific survival. A recent publication showed that delayed gastric emptying (DGE) significantly worsens survival in a Japanese population following pancreatoduodenectomy (PD). The underlying study examines the impact of DGE on cancer-specific survival in out tertiary care center.

**Materials and methods:**

Between January 2008 and June 2018 267 patients underwent pancreatoduodenectomy at our department. Of these, 123 were treated for pancreatic adenocarcinoma (PDAC). Patients were analyzed regarding demographic factors, intraoperative characteristics, morbidity & mortality and long- term oncological survival.

**Results:**

Patients with and without clinically relevant DGE (°B and °C) were comparable with regard to demographic factors, intraoperative characteristics and postoperative outcome. Prognostic factors for cancer-specific survival were positive lymph node status (P=0,032) but not resection margin (P=0,59). Delayed gastric emptying had no impact on survival (P=0,926), nor did pancreatic fistula (P=0,614) or postpancreatectomy hemorrhage (P=0,104).

**Conclusion:**

Delayed gastric emptying does not influence cancer-specific survival in a European collective.

### Laparoscopic cholecystectomy can be done with only three trocars in most cases

(Abstract ID: 493)

H. Bonatti^1^

^1^*Meritus, Hagerstown*

**Background:**

The majority of patients with gallbladder disease undergo conventional laparoscopic cholecystectomy (LC) using four ports, however, new techniques such as SILS, NOTES and robotic assisted cholecystectomy are being promoted. We propose a three port technique for conventional LC with access from the left upper quadrant (LUQ) using a modified dome down technique (MDDLC) as a simple and cost effective alternative.

**Materials and methods:**

A total of 177 LCs performed between 6/2013 and 9/2018, were analyzed. The vast majority of cases was done without 1st assist and the nurse driving the 5mm 30 degree camera from the umbilical port. Trocars are placed in the LUQ (5mm), umbilicus (5 or 10-12mm), and between the two (5mm). The third troacar was replaced by a Teleflex minigrasper in 61 cases (34%). After the gallbladder (GB) serosa is incised on both sides, a window is created behind the GB midportion and widened towards fundus and infundibulum. Cystic artery and duct are dissected out obtaining the critical view (Figure 1) and after the last fundus adhesion is cut, they are secured with clips or endoloop.

**Results:**

Median age of 121 women and 56 men was 54.7 (range 16.5-89.6) years. LC was done for acute cholecystitis (n=31), acute on chronic (n=50), chronic cholecystitis (n=62), other (n=34). In 160 cases (90%), the procedure could be completed with three instruments. In 23 cases additional instruments were used, which included a Keith needle to suspend the GB to the abdominal wall in seven patients, a minigrasper in one to help with GB dissection and a fourth 5mm trocar in 14 patients (for GB retraction in 7 cases, for cholangiography canule placement (n=2) and suction irrigation in one case; in the remaining four cases the additional trocar was inserted for second procedures (paraesophageal hernia repair, cystgastrostomy, appendectomy, right hemicolectomy, extensive lysis of adhesions). Thirty-six cases were done with two five mm ports and a minigrasper only. In 60% the MDDLC was completed, in 22% the MDDLC was switched to a traditional DDLC and 24 cases were started in traditional DD technique. Only the two first cases were done with exposure and division of CA and CD first followed by GB dissection. There were no vascular injuries but one anterior bile duct injury, which was managed with laparoscopic t-tube insertion. Conversion rate in this series was 0%. 35% of cases were done as outpatient procedures another 35% of patients required 23hours observation and 30% of patients were hospitalized.

**Conclusion:**

Three instrument MDDLC with trocar placement in LUQ is feasible and safe in easy and difficult cases and may have multiple advantages over the traditional used technique. The surgical trauma is reduces and in contrast to other new techniques no significant costs are added.

**Picture:**
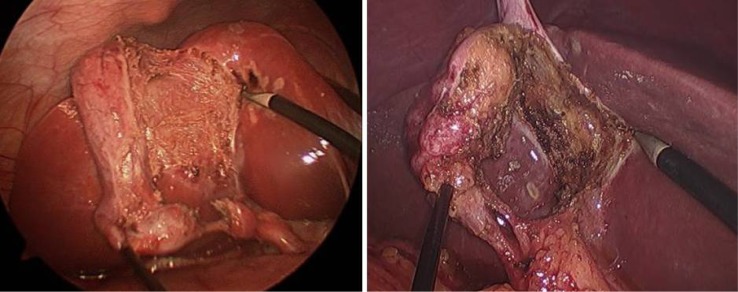
Improved critical view in modified dome down LC: Gallbladder remains suspended at the fundus, the tissue behind the gallbladder is completely dissected

### Simultaneous liver resection suppressed apoptosis of the deportalized lobe in the rat liver undergoing portal vein ligation

(Abstract ID: 502)

C. Hua^1^

^1^*Klinik für Allgemein-, Viszeral- und Gefäßchirurgie, Jena*

**Background:**

Combination of partial hepatectomy and portal vein ligation has been established as a routine procedure to treat large or multiple liver tumors which require extended liver transection. Substantial atrophy of the deportalized liver lobe after portal vein ligation (PVL) is essential for compensatory hypertrophy of the protalized liver lobe. Previous study demonstrated that the balance of proliferation and apoptosis affected the extent of atrophy in deportalized lobe. Besides, it was evidenced that autophagy was promoted by partial hepatectomy and the activation of autophagy enhanced hepatocyte proliferation. However, the effect and mechanism of simultaneous partial hepatectomy (PHx) on regulating atrophy of deportalized lobe remains unclear. We hypothesized that the simultaneous PHx abrogated atrophy of the deportalized liver lobe by inducing hepatocyte proliferation, promoting autophagy and suppressing apoptosis in a size-dependent manner.

**Materials and methods:**

Lewis-rats were subjected to experimental procedures consisting of 20%PVL+70%PHx and 70%PVL+20%PHx respectively. Control groups consisted of 20% PVL only and 70%PVL only. Rats were sacrificed on postoperative day (POD) 1, 2, 3 and 7(n=6/group/time points). Individual liver weight/body weight was calculated. BrdU staining and TUNEL staining were performed to evaluate the proliferation index and apoptotic density. qPCR for mRNA of Proliferating cell nuclear antigen (PCNA) was performed to confirm the proliferation index. Protein levels of LC3-II and caspase3/cleaved caspase3 were detected by western blot.

**Results:**

The deportalized liver lobe adjusted its size in weight differently. Interestingly, the additional small resection reduces the hepatic atrophy of the ligated lobe, whereas the large resection caused a significant increase of hepatic volume of the deportalized lobe. Simultaneous resection induced a size dependent low but substantial hepatocytes proliferation rate (maximal 6.3% and 3.6% when performing either 70%PHx or 20% PHx). Furthermore, additional resection significantly suppressed apoptotic in the deportalized liver, confirming by both TUNEL and protein levels of cleaved caspase3. Of significance, the protein levels of LC3-II elevated in additional large resection compared to small resection.

**Conclusion:**

Atrophy of the deportalized liver lobe was counteracted by the simultaneous PHx. The simultaneous PHx did not only induce mild hepatocyte proliferation, but also did suppress hepatocyte apoptosis significantly in the deportalized liver lobe in a size dependent manner. Meanwhile, autophagy was activated in deportalized lobe after additional large resection and might be involved in suppressing apoptosis.

### Clinical impact of LAPTM4B in pancreatic adenocarcinoma

(Abstract ID: 506)

S. A. Dhayat^1^, Z. Yang^1^, V. Rätzel^1^, N. Senninger^2^, A. Pascher^1^

^1^*Universitätsklinikum Münster*

^2^*Klinikum Oldenburg AöR*

**Background:**

Early detection of pancreatic ductal adenocarcinoma (PDAC) at surgically manageable stages is crucial and offers the best chance of increasing survival. Lysosome-associated protein transmembrane-4-beta (LAPTM4B) is up-regulated in a wide range of cancers associated with poor prognosis. However, the clinical impact of LAPTM4B as diagnostic and prognostic marker in PDAC remains unknown. The aim of the present study was to investigate the expression of LAPTM4B as liquid biopsy marker in PDAC.

**Materials and methods:**

Tissue and preoperative blood samples of 108 patients with PDAC UICC Stages I to IV (n=56), chronic pancreatitis (n=21), pancreatic cystadenoma (n=14), and age-mached healthy blood serum controls (n=17) were collected between 2015 and 2017 prospectively. Expression of LAPTM4B was analyzed by immunohistochemistry, Western blotting and ELISA. Statistical analysis was performed with SPSS Statistics using Mann-Whitney U test or Kruskal-Wallis test, Fisher’s two-tailed exact test, and receiver-operating-characteristic (ROC) method. Values for p<0.05 were considered to be statistically significant.

**Results:**

Expression of LAPTM4B was significantly increased in tumor tissue and in blood serum samples of patients with PDAC versus healthy controls (p=0.002; p<0.001) and versus chronic pancreatitis (p=0.02; p=0.031). Circulating LAPTM4B could well discriminate PDAC from non-PDAC with an Area under the curve (AUC) of 0.89 (95% confidence interval 0.79 to 0.99; p<0.001).

**Conclusion:**

LAPTM4B shows high potential as liquid biopsy marker in PDAC patients. Results of survival analysis and of in vitro functional studies are currently pending.

### Metabolomic Profiling of Liver Tumors and its impact on Liver Regeneration

(Abstract ID: 551)

J. P. Jonas^1^, D. Pereyra^2^, S. Holzer^2^, R. Baumgartner^2^, S. Haegele^2^, C. Brostjan^2^, T. Gruenberger^1^, P. Starlinger^2^

^1^*Sozialmedizinisches Zentrum Süd, Kaiser-Franz-Josef-Spital, Wien*

^2^*Medical University of Vienna, General Hospital, Vienna*

**Background:**

Postoperative liver dysfunction (LD) still represents a severe complication in patients undergoing liver resection and its incidence is estimated at 10-20%. As postoperative LD commonly develops as a result of delayed liver regeneration, it is most relevant to reach a comprising understanding of this process. Thus, we aimed to investigate the perioperative dynamic of circulating metabolites, as well as differences in the metabolic profile of patients with and without postoperative LD using an unbiased metabolomics approach.

**Materials and methods:**

Plasma from 95 prospectively included patients was collected preoperatively and on the first and fifth postoperative day (POD5). Per patient and time point 180 metabolites were assessed using the Biocrates p180-kit. Development of LD was prospectively recorded.

**Results:**

21 patients (19.95%) suffered from postoperative LD. We observed significant dynamics in the metabolic profile after liver surgery, that tended to normalize upon POD5. Further, we were able to document differences in the concentration of 120 metabolites between patients with and without postoperative LD. Interestingly, the family of sphingolipids showed an evident accumulation of differentially abundant metabolites in patients with LD at several time points, while the total amount of sphingolipids did not differ.

**Conclusion:**

Within this study we present the first data on the metabolic profile in patients undergoing liver resection and in patients with delayed liver regeneration. While we found a plethora of potential markers for postoperative LD at various time points, we also present hypothesis generating data and the opportunity to characterize potentially targetable pathways for improvement of postoperative liver regeneration.

### Hepatocellular Carcinoma – Are Circulating tumor cells a predictive factor for malignant dissemination?

(Abstract ID: 581)

Wehrmann^1^, J. Wagner^1^, J. Clement^1^, F. Rauchfuß^1^, U. Settmacher^1^

^1^*Universitätsklinikum Jena*

**Background:**

Late diagnosis and aggressive dissemination are main reasons why hepatocellular carcinoma (HCC) are the second most common cause of cancer-related deaths worldwide. Primary resection or, in case of liver cirrhosis or bilobary manifestation, liver transplantation are the only curative therapy-strategies. The time between diagnosis of HCC and resection or transplantation is called "bridging-time", in which tumor mass and biological activity can be down-regulated by supporting therapies like transarterial chemoembolization (TACE) or selective internal radiotherapy (SIRT). It was shown in former studies that free tumor cells after resection are associated with poor outcome in future. Aim of this study was to show that vital circulating tumor cells (CTC) can be used as a prognostic factor to prevent metastasis although supportive therapies and resection/ transplantation of the primary tumor is already done.

**Materials and methods:**

From 2012 to 2018, 186 HCC patients who underwent supportive therapy as TACE or SIRT, resection or transplantation at the Department of Surgery at the University Hospital of Jena were enrolled. Blood specimens were obtained before or after surgical or conservative intervention and analyzed for EpCAM+/ Propidium-vital CTC. Follow-up data and blood samples were taken from 52 patients. Retrospectively the data were correlated to patients ‘clinical data.

**Results:**

All 186 patients, 165 male and 21 female, could be included. 71 patients were resected or transplanted, 115 were bridged to transplantation or underwent palliative therapy. 52 patients were followed up with analysis of blood specimens. 12 of them got metastasis during follow up. EpCAM positive CTC during follow up showed in 83% (n=10) a positive correlation to malignant dissemination in future (p< 0,01).

**Conclusion:**

Blood-stream detection of vital CTC is a good tool to detect malignant dissemination and could identify high-risk patients that can profit from a more aggressive therapy after resection or during bridging therapy like similar, for example, intake of protein kinase inhibitors. Indeed, prospective studies with well evaluated protocols are needed to underline these data and to show how to treat patients during bridging therapy to prevent them from malignant dissemination.

### Liver resection for recurrence of colorectal liver metastasis after completion of ALPPS procedure

(Abstract ID: 629)

T. Reese^1^, M. H. Fard-Aghaie^1^, G. Makridis^1^, A. Kantas^1^, K. J. Oldhafer^2^

^1^*Asklepios Klinik Barmbek, Hamburg*

^2^*Asklepios Campus Hamburg*

**Background:**

About one third of the patients with colorectal cancer will develop liver metastasis within three years after diagnosis, but only 15-20% will be legible for resection. Although advanced medical therapy results in better survival, resection of liver metastasis is the only curative option. Anatomical or non-anatomical resection has no consequences for tumor recurrence. Patients that were considered as unresectable, a two-staged hepatectomy such as ALPPS provide a technique to improve resectability in about 20% of those patients. This procedure has an extensive regenerative response of the liver parenchyma.

About 57% develop recurrence after initial liver resection and re-resections are described as a safe and feasible option. Studies show a 5-year-survival rate of 33-75% with no perioperative mortality. The only difference of the repeated resection, compared to the first resection, is that the surgical technique becomes more difficult. However, re-resections after ALPPS for colorectal liver metastasis (CRLM) are not described. In this case series, we examine the feasibility and outcome for ALPPS in the setting of repeated liver resection for CRLM. It is unknown whether the regenerative response after ALPPS completion is still obtainable for another resection of liver parenchyma.

**Materials and methods:**

All patients that underwent resection for recurrence for CRLM after the completion of ALPPS procedure were included in the study. Operation details, volumetric analysis and complications (according to Clavien-Dindo) were assessed for stage-1, stage-2 and the resection for recurrence. Demographics and survival were analyzed for each patient. Data are reported as median (interquartile rage), mean (standard error), and numbers with proportions (%), where appropriate. Statistical analysis was performed using SPSS v23© for Mac.

**Results:**

Out of 68 ALPPS procedures during the study period from 11/2010 until 12/2017, we performed 46 ALPPS procedures for CRLM, of those 42 were completed (91%). Overall, six patients underwent resection for recurrence. During stage-1 the majority underwent classic ALPPS with a low morbidity (CCI of 10,5). The mean sFLR before stage-2 was 33% and only extended resections were performed during stage-2, with half of the patients receiving an additional atypical resection of the future liver remnant.

After completion of stage-2 the mean time to recurrence was 328 days. The majority of patients underwent atypical resection for recurrence (n=5). Of those, one patient had additional microwave ablation. One patient even received a segmentectomy. Mean operation time was 193 Minutes. The perioperative morbidity was low with a CCI of 5,6 and no perioperative mortality occurred. Furthermore, no posthepatectomy liver failure was observed. Two patients even received a third hepatectomy for a second recurrence with no perioperative mortality. The one- and three-year survival was 83% and 56%, respectively.

**Table:** 

Days until Recurrence, mean (SE)	328	(76)
OR Time, mean (SE)	193	(25)
Atypical Resection, n (%)	4	(67)
Atypical Resection plus MWA, n (%)	1	(17)
Segmentectomy, n (%)	1	(17)
Comprehensive Complication Index, mean (SE)	5,6	(5,6)
Perioperative Mortality, n (%)	0	(0)

**Conclusion:**

This is the first study about resection for recurrence after the completion of ALPPS. Even after the extensive regenerative response after ALPPS procedure, we show that the surgical approach in this setting is feasible with no perioperative mortality. Furthermore, the survival of those patients seem to be comparable with the current literature for patients undergoing repeated liver resections.

### Lymph node involvement in the ductal adenocarcinoma of the pancreas: A survival analysis

(Abstract ID: 713)

S.-A. Safi^1^, L. Dizdar^1^, A. Rehders^1^, W. T. Knoefel^1^, A. Krieg^1^

^1^*Universitätsklinikum Düsseldorf*

**Background:**

Overall survival after surgery for pancreatic ductal adenocarcinoma (PDAC) remains poor. While some studies suggest that positive nodal status (N1) is one of the most important prognostic factors after margin-negative (R0) resection, other data imply that nodal disease is not associated with prognosis. Thus, the aim of this study was to investigate the prognostic value of lymph node (LN) staging systems such as the pN categorization, metastatic LN ratio (LNR) and log odds of positive LNs (LODDS). In addition, we evaluated the prognostic relevance of LN metastasis in different LN stations by using the different LN staging systems.

**Materials and methods:**

Clinicopathological data from 308 patients who underwent pancreatic resection for PDAC between 2003 and 2018 were analyzed. Histopathologic reports were re-assessed to gather data on the nodal status. Both LNR (ratio of positive LN to examined LN) and LODDS [log(positive LN+0.5)/(total LN+0.5)] were calculated from the peripancreatic and total LN yield, respectively. In addition a separate LN mapping was performed on the interaortocaval (IAC), hepatoduodenal ligament (HDL), portal vein (PV), celiac trunk (CT) and superior mesenteric artery (SMA) stations. Individual cut off levels and subgroups were set both for LNR and LODDS by the median and quartiles respectively. Overall survival (OS) was determined for all patients by the Kaplan-Meier method and Cox regression.

**Results:**

Of the 308 patients, 264 patients (85,7%) underwent partial pancreaticoduodenectomy (pPD). Moreover, 14 (4,5%) and 29 patients (9,4%) received total pancreatectomy and oncologic pancreatic tail resection, respectively. 248 patients (80,5%) showed LN infiltration (N1), while 46 patients (15%) presented with distant metastasis (M1). In contrast to the pN status, multivariate analysis identified(MV) M1 situation, positive surgical margins (R1) and tumor localization as independent prognostic factors. Of note, LNR and LODDS were significantly associated with worse overall survival (OS). Both, LNR and LODDS that were specifically assessed in the peripancreatic LNs correlated with poor survival in UV and MV analysis. However, LNR turned out to be a stronger independent prognostic factor than LODDS. In further subgroup analyses including only R0 resected PADCs independently of the localization (head, body, tail), R0 resected pancreatic head cancer or pancreatic head cancer independently of resection margins, again pN failed to be a prognostic factor. Importantly, LNR remained to be an independent prognostic factor in the total and peripancreatic LN. In contrast, the LODDS predicted survival only for the total LN yield. In addition, LN mapping showed positive LNs in the IAC, HDL, PV, CT and SMA stations for 42, 26, 12, 17 and 18 patients, respectively. Interestingly, positive SMA lymph nodes correlated with poor OS in MV analysis [HR=3,0 (95% CI 1.4-6.4)]. Importantly, hospital stay and morbidity showed no significant difference in cases with extended compared to standard LAD.

**Conclusion:**

In contrast to pN, LNR and LODDS are powerful prognostic factors in PDAC. Therefore, LNR and LODDS should be included in pathologic reporting after PDAC resection and taken into consideration for the prognostic assessment of PDAC patients. Moreover, we show that positive IAC LNs had no impact on survival, in our cohort of PDAC patients, supporting an extended LAD for PDAC.

### Preoperative Computertomography predicts Portal Vein Infiltration in the Ductal Adenocarcinoma of the Pancreas Head

(Abstract ID: 717)

S.-A. Safi^1^, S. Heueveldop^1^, A. Rehders^1^, W. T. Knoefel^1^, A. Krieg^1^

^1^*Universitätsklinikum Düsseldorf*

**Background:**

Pancreatic ductal adenocarcinoma (PDAC) is a highly aggressive malignancy and most patients present with locally advanced disease. To achieve tumor clearance, venous resection of the portal vein (PV) or superior mesenteric vein (SMV) during partial pancreatoduodenectomy (pPD) has steadily been gaining acceptance. It is unclear whether preoperative radiologic assessment is a viable tool to identify patients with PV/ SMV involvement. Nevertheless, previous studies have shown that intraoperative re-resection to achieve secondary margin clearance does not influence overall surival, thus survival in primary R0 resections is superior. In addition further pathological studies have shown, that the medial resection margin, the adjacent site of the PV/SMV, is the main site of microscopic tumor infiltration (R1). Consequently, the aim of this study was to investigate the correlation of pre-operative CT morphological and post-operative histopathological findings. Furthermore, clinicopathological parameters were analyzed to identify significant survival determinants.

**Materials and methods:**

We reviewed the records of patients, who underwent pPD between 2003 and 2018 in our Institution. We herein analyzed clinicopathological data, morbidity, mortality and survival outcome. Pre-operative CT scans were retrospectively re-analysed by an experienced radiologist in a blinded fashion and correlated with histopathological and clinical findings.

**Results:**

Pre-operative CT scans were available for re-assessment in 179 patients whom received pPD for PDAC. 4 patients received neoadjuvant treatment. 132 patients were staged UICC IIB (8th edition). Complete tumour clearance was achieved in 141 patients. 74 patients received portal vein resection during pPD. All patients received an adjuvant treatment regime. In 113 patients (63%, group NIF), tumour contact with the PV/SMV was not visible pre-operatively, whereas in 67 patients (37%, group IF) tumour contact was suspected. In both groups, clinicopathological variables were homogeneously distributed. In group NIF, venous resection was performed in 35 patients, but venous infiltration was confirmed histopathologically in only 5 patients (14%). In group IF, venous resection was performed in 38 patients, with an actual infiltration rate of 66%. Suspected venous infiltration correlated significantly with actual infiltration (p<0.01). Portal vein resection was not a predictive factor for prolonged hospitalization (average 28 vs 31 days). On univariate analysis, factors influencing OS included UICC, tumour grade, R-status and CT-suspected portal vein infiltration. On multivariate analysis only R-status was associated with poor OS.

**Conclusion:**

Pre-operative assessment of PV/SMV infiltration correlated with histopathological findings. In survival analysis, only R status was an independent prognostic factor. PV/SMV resection did not increase morbidity nor prolong hospitalization. Furthermore, previous studies showed no survival benefit after intraoperative re-resection to achieve secondary margin clearance This suggests that PV/SMV resections should be performed when tumor infiltration is suspected to obtain primary margin clearance in order to increase local tumor control and improved survival outcome.

### Recurrence patterns in PDAC – An international cohort study

(Abstract ID: 721)

K. Honselmann^1^, I. Pergolini^2^, C. Fernandez-del Castillo^2^, M. Sandini^2^, L. Bolm^1^, U. F. Wellner^1^, A. L. Warshaw^2^, K. Lillemoe^2^, T. Keck^1^, C. Ferrone^2^

^1^*Uniklinik Lübeck*

^2^*Massachusetts General Hospital and Harvard Medical School, Boston*

**Background:**

Pancreatic ductal adenocarcinoma (PDAC) is predicted to become the second leading cause of cancer death by 2030. This is mostly to due to early local and distant metastasis, even after surgical resection. Knowledge about patterns of recurrence in different patient populations could offer new therapeutic avenues.

**Materials and methods:**

Clinicopathologic data were collected for 546 patients who underwent resection of their PDAC between 2005 and 2016 from two tertiary university centers. Patients were divided into an upfront resection group (n=394) and a neoadjuvant group (n=152).

**Results:**

Recurrence was common among resected PDAC patients and differed in time and occurence based on lymph node status (upfront surgery pN0: 55%,16 months vs. pN1 77%,10 months, p<0.001 and neoadjuvant group pN0: 64%, 21 months vs. pN1 78%, 11 months, p=0.040, p<0.0001). Irrespective of lymph node status or neoadjuvant therapy, distant metastasis (upfront surgery pN0 63% vs.pN1 62%, p=0.553 and neoadjuvant group pN0 76% vs. pN1 64%, p=0.326) was the most frequent type of recurrence. This was also true for specific sites of recurrence (lung only, liver only, lung and liver, and multiple sites) as first site of recurrence (upfront surgery: pN0 : lung only: 22%, liver only:49%, lung&liver, 3%, other/multiple sites: 27% vs. pN1: lung only: 25%, liver only:36%, lung&liver,7%, other/multiple sites: 32%, p=0.718, neoadjuvant group: pN0 : lung only: 24%, liver only:38%, lung&liver, 6%, other/multiple sites: 32% vs. pN1: lung only: 27%, liver only:44%, lung&liver, 2%, other/multiple sites: 27%, p=0.568).

**Conclusion:**

Time to and total number of recurrence was significantly shorter and smaller in PDAC patients with lymph node positive disease. However, patterns of recurrence for pN0 and pN1 patients were identical.

### Modified ante situm liver resection without use of cold perfusion nor veno-venous bypass – A Single Center Experience

(Abstract ID: 741)

F. Oldhafer^1^, K. I. Ringe^1^, K. Timrott^1^, O. Beetz^1^, W. Ramackers^1^, S. Cammann^1^, J. Klempnauer^1^, F. W. R. Vondran^1^, H. Bektas^2^

^1^*Medizinische Hochschule Hannover*

^2^*Klinikum Bremen-Mitte*

**Background:**

Treatment of liver malignancies invading the hepatic veins and/or inferior vena cava is a challenge even for the experienced HPB-surgeon. The ante situm technique allows for luxation of the liver in front of the situs to improve access to the hepatocaval confluence and the vena cava but usually includes cold perfusion and veno-venous bypass to perform tumor resection. Experience with modified ante situm resection relying only on total vascular occlusion at Hannover Medical School is reported.

**Materials and methods:**

Retrospective analysis of 15-year experience with ante situm resection without application of neither cold perfusion nor veno-venous bypass is presented.

**Results:**

Ante situm technique was applied on 8 patients without cold perfusion nor veno-venous bypass. Five patients were treated due to intrahepatic cholangiocellular cancer and 1 case each for mixed cholangio-/hepatocellular carcinoma, colorectal liver metastasis and pheochromocytoma. Trisectorectomy (n=4), left hemihepatectomy, right hepatectomy, atypical resection or mesohepatectomy (each n=1) was performed in combination with dissection of suprahepatic/retrohepatic vena cava/hepatic veins. Venous reconstruction was achieved by reimplantation of hepatic veins with/without vascular replacement using allogeneic donor veins or PTFE-grafts. Median total vascular occlusion of the liver was 23 minutes. Severe morbidity occurred in 3 patients (Dindo-Clavien >3A). R0-status was achieved in 6 cases with a median overall survival of 33.5 months.

**Conclusion:**

Ante situm liver resection can be applied without cold perfusion nor veno-venous bypass with acceptable morbidity and mortality in selected cases. However, this procedure remains challenging due to the need for complex vascular reconstruction.

### Risk factors for pancreas-specific complications after pancreatic surgery – multivariate analysis of six randomised controlled trials

(Abstract ID: 784)

Z. M. Zoll^1^, R. Klotz^2^, F. J. Hüttner^2^, C. Skolik^3^, A. Sander^3^, M. W. Büchler^2^, M. K. Diener^2^

^1^*Heidelberg*

^2^*Department of General, Visceral and Transplantation Surgery, Heidelberg*

^3^*Universitätsklinikum Heidelberg*

**Background:**

Despite substantial improvements in surgical practice leading to decreased mortality in recent decades, pancreatic surgery is still associated with substantial rates of postoperative morbidity of up to 50 %. Postoperative morbidity comprises predominantly surgical or pancreas-specific complications, such as postoperative pancreatic fistula, bile leakage, delayed gastric emptying, postpancreatectomy haemorrhage, intra-abdominal fluid collections or abscess. These complications can result in reduced quality of life of patients, are associated to prolonged hospital stay and increased health care costs and might delay adjuvant chemotherapy or further necessary treatment.

**Materials and methods:**

The current analysis includes individual patient data of six high quality randomised controlled trials on different pancreatic surgical interventions, which were conducted by the Study Center of the German Surgical Society (SDGC).

Cumulative frequency of individual, postoperative complications was calculated. Risk factors for pancreas-specific complications were identified by uni- and multivariate analysis. Covariates included baseline factors (e.g. age, gender, BMI, smoking status, diabetes, ASA-classification, immunosuppressants, comorbidities) and surgical factors (e.g. length of surgery, blood loss, texture of the pancreas).

A multivariate logistic regression analysis and the Cox regression will be applied. Two-sided P values will be computed and P <= 0.05 will be considered statistically significant.

**Results:**

Data on a total of 1918 patients were included in this analysis. 1112 were male, mean age was 61.2 years (95% CI 60.7 - 61.8) and average BMI was 25.0 kg/m2 (95% CI 24.8 - 25.2). 962 patients underwent a partial pancreatoduodenectomy, 515 patients received a distal pancreatectomy, in 170 cases a duodenum-preserving head resection was performed, 79 patients received a total pancreatectomy and in 192 cases other operations were performed.

Clinically relevant postoperative pancreatic fistula occurred in 11.6 % after partial pancreatoduodenectomy and 20.9 % after distal pancreatectomy. 24.0 % of patients after partial pancreatoduodenectomy and 3.8 % after distal pancreatectomy developed delayed gastric emptying. Postpancreatectomy haemorrhage was described in 9.1 % after partial pancreatoduodenectomy and 5.7 % after distal pancreatectomy.

Furthermore 14.2 % had an intra-abdominal fluid collection after partial pancreatoduodenectomy and 27.1 % after distal pancreatectomy. In 3.0 % of the cases a bile leakage was described after partial pancreatoduodenectomy and in 1.1 % after distal pancreatectomy.

Analysis of risk factors is currently ongoing, however results will be presented at the surgical congress.

**Conclusion:**

The aim of this post-hoc analysis is to identify clinically relevant risk factors for postoperative morbidity and mortality after pancreatic surgery based on data from six high quality randomised controlled trials. The results might be the basis for a more reliable preoperative risk assessment, patient counselling and an individualized risk reduction in the future.

### Prognostic value of inflammation-based scores in patients wih adenocarcinoma of the pancreas prior to surgery with curative intention

(Abstract ID: 805)

M. C. Langheinrich^1^, J. Burlein^1^, G. F. Weber^1^, S. Merkel^1^, R. Grützmann^1^, S. Kersting^1^

^1^*Universitätsklinikum Erlangen*

**Background:**

Pancreatic cancer is one of the leading causes of cancer death worldwide, with an overall 5-year survival rate of less than 5%. Survival rates vary among patients, especially with advanced pancreatic cancer. The nutritional status plays an important role in prognosis of different type of cancers, the immunonutritional status is reflected via the prognostic nutritional index (PNI), calculated on serum albumin and peripheral lymphocyte count. Systemic inflammatory response parameters, neutrophil to lymphocyte ratio (NLR), lymphocyte to monocyte ratio (LMR), consider the pro-tumorigenic properties of neutrophils respectively while considering the protective effect of lymphocytes. The purpose of this study was to determine the prognostic value of different easily available parameters for a better risk stratification in pancreatic cancer patients.

**Materials and methods:**

From 2010 to 2016, we conducted a retrospective analysis of 167 patients with pancreatic cancer who underwent surgery for curative intention. Routine laboratory measurements, including white blood cell count: neutrophil, lymphocyte, monocyte and serum albumin were performed prior to surgery. The NLR, LMR and PNI: serum albumin (g/l) + 0,005*total lymphocyte count (per mm³) were calculated. Receiver operating characteristic (ROC) analysis and Youden index were used to identify the best cutoff values. Survival curves were determined by the Kaplan-Meier method.

**Results:**

Among the 167 patients, 103 (62%) received a tumor resection and 64 (38%) patients not. The majority (66%) had no neoadjuvant therapy prior to surgery. Overall, 125 patients presented with NLR <=5.4 (75%), 42 patients with NLR > 5.4 (25%); 115 patients with a PNI >36,5 (69%) and 52 patients with PNI <=36,5 (31%). Patients with NLR >5.4 had a 2-year-survival of 15 months, significantly (p<0,002) worse compared to patients with NLR <= 5.4 and a 2-year-survival of 30 month. Regarding just the subgroup of patients with no tumor resection (n=64), there was no significant correlation between NLR and 2-year survival. The PNI correlated significantly with worse OS in this group (p<0,001), 6 months versus 16 months.

**Conclusion:**

NLR and PNI, simple and easily to derive markers, are useful prognostic indicators for OS in patients with pancreatic cancer. Further independent prospective study's are needed to help guide risk stratification for treatment decisions, especially to find out patients who did not benefit from surgery.

**Picture:**
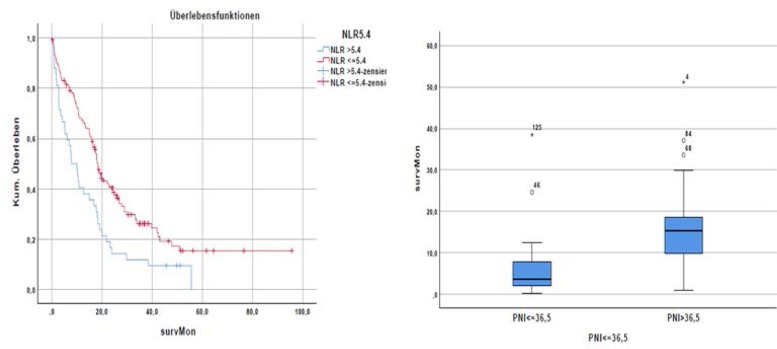
Overallsurvival NLR; OS and PNI distribution for subgroup patients with no tumor resection

### Incidence and treatment of portal vein thromboses after portal vein resections in pancreatic surgery

(Abstract ID: 830)

M. Weniger^1^, Z. Lu^2^, A. Doll^1^, Y. Miao^2^, J. Werner^1^, J. D'Haese^1^

^1^*Uniklinik München*

^2^*The First Affiliated Hospital of Nanjing Medical University, Nanjing*

**Background:**

With increasing numbers of patients treated for locally advanced and borderline resectable pancreatic cancer, the number of portal vein resections (PVR) is increasing. While the operative technique is clearly defined, data for the postoperative management after PVR after scarce. Furthermore, it is unclear whether patients with PVR should receive anticoagulation beyond standard thromboprophylaxis after surgery. Thus, the present study examines the incidence, risk factors, and prevention of PVT after PVR.

**Materials and methods:**

Data on all patients with PVR and pancreatic surgery of any kind between 01/2014 and 12/2017 from two pancreatic centers was collected. Subsequently, patient data was analyzed for the incidence of PVT, operative and non-operative risk factors, and thromboprophylaxis.

**Results:**

Overall, 132 patients with PVR were analyzed. Of those, 4.6% (n=6) had PVT within 30 days (PVT<30d) postoperatively. Neither the type of PVR nor any other of the investigated risk factors was associated with PVT <30d. PVT<30d was not associated with a change in hospital length of stay or mortality. Additionally, therapeutic dosing of thromboprophylaxis or thromboprophylaxis for more than 4 weeks postoperatively had no effect on the incidence of PVT<30d.

**Conclusion:**

PVR can be done safely with a low risk of PVT. Standard postoperative thromboprophylaxis with prophylactic dosing is sufficient to prevent PVT.

### Brg1 promotes liver regeneration after partial hepatectomy via regulation of cell cycle

(Abstract ID: 831)

B. Wang^1^, B. Kaufmann^1^, T. Engleitner^1^, M. Lu^1^, C. Mogler^1^, V. Olsavszky^2^, R. Öllinger^1^, C. Géraud^2^, Z. Cheng^1^, R. Rad^1^, R. Schmid^1^, N. Hüser^1^, H. Fr iess^1^, G. von Figur a^1^, D. Hartmann^3^

^1^*Klinikum Rechts der Isar der TU München*

^2^*Universitätsmedizin Mannheim*

^3^*Klinik und Poliklinik für Chirurgie, München*

**Background:**

Brahma-related gene 1 (Brg1), a catalytic subunit of the SWItch/Sucrose Non-Fermentable (SWI/SNF) complex, is known to be involved in proliferative cell processes. Liver regeneration is initiated spontaneously after injury and leads to a strong proliferative response. The aim of the study was to investigate the role of Brg1 in liver regeneration following partial liver resection in mice.

**Materials and methods:**

In this study, a hepatocyte-specific Brg1 gene knockout mouse model was used to analyse the role of Brg1 in liver regeneration by performing a 70% partial hepatectomy (PH). Liver regeneration was analysed by liver/body weight ratio and cell proliferation rate. Different cellular pathways were analysed by RNA sequencing.

**Results:**

After PH, Brg1 was significantly upregulated in wildtype mice. Mice with hepatocyte-specific Brg1 gene knockout showed a significantly lower liver to body weight ratio 48 h post-PH concomitant with a lower hepatocellular proliferation rate compared to wildtype mice. In addition, proliferation rate of the hepatocytes was significantly higher in wildtype mice after PH compared to the proliferation rate of hepatocyte-specific Brg1 gene knockout mice. RNA sequencing demonstrated that Brg1 controlled hepatocyte proliferation through the regulation of several cell cycle genes.

**Conclusion:**

The data of this study reveal a crucial role of Brg1 for liver regeneration by promoting hepatocellular proliferation through modulation of cell cycle genes and, thus, identify Brg1 as potential target for future therapeutic approaches.

### HGF signaling plays a vital role in the early stage of liver regeneration after PHx in mice

(Abstract ID: 836)

X.-J. Zhang^1^, B. Wang^1^, V. Olsavszky^2^, K. Schledzewski^2^, C. Géraud^2^, N. Hüser^1^, H. Friess^1^, G. von Figura^1^, D. Hartmann^3^

^1^*Klinikum Rechts der Isar der TU München*

^2^*Universitätsmedizin Mannheim*

^3^*Klinik und Poliklinik für Chirurgie, München*

**Background:**

Hepatocyte growth factor (HGF) is a complete hepatic mitogen and is considered an important initiator of liver regeneration. Liver regeneration after partical hepatectomy (PHx) is known to be a complex process where liver sinusoidal endothelial cells (LSECs) play an important role. LSECs are one of the most important liver cell types that produce HGF, but the exact contribution of LSECs to liver regeneration remains to be defined.

**Materials and methods:**

In order to investigate the effects of HGF signaling on liver regeneration, Stab2-Cretg/wt;HGFfl/fl mice, where HGF is specifically knocked out in LSEC, were used. These mice underwent an 70% PHx and the kinetics of liver-to-body weight ratio, hepatocyte proliferation, HGF/c-MET signaling pathways and cell-cycle-associated genes were analyzed at different time points after PHx. In addition, RNA sequencing was performed.

**Results:**

We demonstrated that HGF-LSECKO mice showed a significantly reduced liver-to-body weight ratio compared to the control group at 72 hours after PHx. HGF-LSECKO mice had a higer mortality after PHx and the proliferation of hepatocytes was significantly impaired at 48 hours after PHx in HGF- LSECKO mice.

**Conclusion:**

HGF signaling plays a vital role in the early stage of liver regeneration after PHx in mice. This signaling pathway is not only essential for liver regeneration after injury, but also crucial for the growth of the liver.

### Postoperative infectious complications after liver resection

(Abstract ID: 885)

B. Wellge^1^, A. Heumann^1^, L. Fischer^1^, J. Izbicki^1^, J. Li^1^

^1^*Universitätsklinikum Hamburg-Eppendorf*

**Background:**

Although mortality associated with liver surgery has decreased, high morbidity rates are still of major concern. This study aimed to identify the prevalence of, and risk factors for infectious complications after liver surgery.

**Materials and methods:**

A prospective database of 493 consecutive liver resections performed by the University Hospital of Hamburg-Eppendorf between 07/12 and 01/2017 was analyzed. Infectious complications were classified in non-liver related (NLR) infections and liver related infections (LR). Risk factors that were significantly associated with infectious complications in univariable models were included in a multivariable logistic regression model.

**Results:**

Infectious complications occurred in 138 of 493 patients (28.0%) of which 98 (19.9%) could be classified as NLR and 40 (8.1%) as LR (details table 1). The infections could be classified according to WHO in A: 108 (21.9 %), B: 20 (4.1%) and C: 10 (2.0%). Surgical site infections occurred in 46 patients [9.3%; WHO A1: 34 (6.9%), A2: 8 (1.6%), A3: 4 (0.8%)]. Ten independent risk factors for infectious complications after liver resection could be identified: male sex, age 70 years or more, body mass index at least 25 kg/m2, diabetes mellitus, chemo therapy < 4 weeks before surgery, surgical approach, performance of ALPPS-procedure, performance of an enterohepatic anastomosis, liver disease.

**Table:** 

Non-liver related infections	laboratory signs for infection w/o focus	n=49	34.3 %
	pneumonia	n=19	13.3 %
	urinary tract infection	n=28	19.6 %
	i.v. catheter related infections	n=7	4.9 %
liver related infections	bilioma	n=12	8.4 %
	peritonitis / abscess	n=18	12.6 %
(n=143 (5 colocalized))	cholangitis	n=10	7.0 %

Infections complications after liver resection

**Conclusion:**

Infectious complications after liver resection are still a major concern and a broad spectrum of patient, disease and surgery related risk factors could be identified. The majority of occurring infections are not directly liver related and might be addressed by focused assessment and optimization of perioperative care like in an enhanced recovery after surgery program (ERAS).

### The prognostic value of immune cell infiltration in cholangiocarcinoma

(Abstract ID: 891)

V. Branchi^1^, A. Jafari^1^, B. Jürgensen^1^, P. Lingohr^1^, M. Gonzelez-Carmona^1^, G. Kristiansen^1^, J. C. Kalff^1^, M. Toma^1^, H. Matthaei^1^

^1^*Universitätsklinikum Bonn*

**Background:**

Biliary tract cancer (BTC) is an aggressive malignant neoplasm with extremely few therapeutic options. The characterization of the immune cell-related microenvironment is crucial in order to identify novel therapeutic targets. The aim of this study was to assess prognostic factors associated with immune cell infiltration in BTC.

**Materials and methods:**

Tumor samples from 45 patients who had undergone surgical resection for BTC between 2014 and 2017 at the University Hospital Bonn were investigated. Using immunohistochemistry, we identified tumor-infiltrating T lymphocytes (CD4 , CD8 ), natural killer cells (perforin ) as well as tumor regulatory T cells (CD103 ). Furthermore, the overall quantity and relative cell density were analyzed. Clinical data were obtained and the clinicopathological impact of immune cell infiltration was evaluated.

**Results:**

Tumors with a high density of infiltrating T lymphocytes were characterized by a longer overall survival (25±7.7 months vs. 28±5.6 months; P=0.039). This positive association between T lymphocyte infiltration and survival was particularly evident in the subgroup of extrahepatic malignancies (OS 25±4.5 months vs. 34±3.4 months; P=0.048). The strongest association with overall survival was found within the CD8 relative density, which turned out to be an independent prognostic factor (P=0.019). The CD4 /CD8 ratio was similar in extrahepatic and intrahepatic BTC. In our preliminary data, a high density of CD103 cells correlated with longer overall survival.

**Conclusion:**

The immune microenvironment plays a crucial role in cancer development and response to therapy. Our study underlines the complexity of the tumor infiltrating T cell population, which represents a promising target for immunomodulatory agents.

### Implementing an enhanced recovery protocol for elective duodenopancreatectomy: Is it worth it?

(Abstract ID: 908)

B. Wellge^1^, N. Küsters^1^, C. Trepte^1^, L. Nawrath^1^, J. Izbicki^1^, M. Bockhorn^1^

^1^*Universitätsklinikum Hamburg-Eppendorf*

**Background:**

Although mortality associated with pancreatic surgery has decreased dramatically, high morbidity rates are still of major concern. Enhanced recovery after surgery (ERAS) protocols have been developed for most major abdominal surgeries. Specific guidelines also for duodenopancreatectomy have been published, but mainly by extrapolating data collected from colorectal surgery.

**Materials and methods:**

An ERAS protocol for pancreatic surgery has been implemented successfully at the University Hospital in Hamburg. Consecutive perioperative data of 121 unselected patients undergoing duodenopancreatectomy could be prospectively entered in the ERAS database from 02/16 till 05/18. The control group consisted of 36 historical duodenopancreatectomies of the pre-ERAS era, perioperative data was assessed retrospectively.

**Results:**

The ERAS protocol could be successfully implemented for pancreatic surgery in our hospital, the adherence to the protocol starting from 24.0% in the pre-ERAS era constantly increased to 67.9% in 2018. Regarding the different perioperative phases, the adherence is highest in the pre-operative phase: 94%, still 82% in the intra-operative phase but just 38% in the regarding the postoperative phase.

Nevertheless, by the implementation of an ERAS protocol in pancreatic surgery, median length of stay could be reduced by 4.4 days (19.3 vs. 14.9d) as well as severe complications by 5.5% (CD >=IIIa: 36.1 vs. 30.6%) without negatively affecting the readmission rate (11.1 vs. 7.4%). Pancreatic fistulas could be reduced by 6.5% (22.2 vs 15.7%), as well as postoperative obstipation by 9.4% (11.1 vs 1.7%). Evaluation of ASA and PPOSSUM Score indicates even patients of a slightly higher perioperative risk in the ERAS-group.

**Table:** 

	pre-ERAS	ERAS
TV 30 data	11.1 %	9.9 %
Surgical revison	30.5 %	24.8 %
overall perioperative complication	58.3 %	74.4 %
severe perioperative complication (ClavienDindo:>=IIIa)	65.7 %	70.3 %
ASA 3-4	65.7 %	70.3 %
PPOSSUM Score	1.2 %	6.8 %

**Conclusion:**

The implementation of an ERAS-program for pancreatic surgery is feasible and successful. Pure extrapolation of data from other ERAS organ protocols is not sufficient.

To further develop the ERAS program we need to make sure that we are part of the same ERAS program. If everybody establishes its own modified ERAS protocol, ERAS will not be more than a pure marketing tool.

### Endoscopic necrectomy of infected WON – development of an effective therapeutic algorithm based on clinical peri-interventional case management experiences obtained in a monocentric study population

(Abstract ID: 927)

K. Jäger^1^, F. Füldner^1^, F. Meyer^2^, U. Will^1^

^1^*Municipal Hospital (“SRH Wald-Klinikum Gmbh”), Gera*

^2^*Universitätsklinikum Magdeburg*

**Background:**

Necrotizing pancreatitis is the most severe clinical course of acute pancreatitis which is still associated with a substantial morbidity and mortality. The formation of "Walled-Off Necrosis" (WON) is relatively common and in this case, a secondary infection can occur. Minimally invasive endoscopic and surgical interventions have priority in the therapy. In this context, percutaneous and endoscopic ultrasonography (EUS)-guided internal drainages are preferred.

**Materials and methods:**

To further improve the treatment, an algorithm is required for an appropriate approach. Therefore, the monocentric population of our institution was investigated retrospectively in prospectively collected peri-interventional data (study design). Overall, 105 patients with necrotizing pancreatitis and infected WON undergoing translumenal endoscopic necrectomy (inclusion criteria) at the Municipal Hospital ("SRH Wald-Klinikum GmbH") at Gera (Germany) from 2004 to 2017 were identified.

**Results:**

In total, 68 men and 37 women were enrolled in the study (median age, 59 years). Mean hospital stay was 44 days. Fourty five % of the patients were treated at the intensive care unit; 86 % of the cases presented with a bacterial infection of the WON whereas in 40 %, mycotic infection was found. First interventional measure was performed after a mean time period of 28 days; in 54 %, this was a percutaneous drainage. Internal drainages were used in 46 patients (43.8 %) after a mean time period of 35 days. The combination of percutaneous and internal drainages permitted an excessive lavage, which was performed in 57 patients (54.3 %). Eighty seven individuals (82.9 %) were treated by internal (transluminal) endoscopic necrectomy and in 8 subjects (9.2 %), a percutaneous necrectomy was additionally performed. In only 20 patients (19 %), complete necrectomy was achieved. It was found that for the therapeutic success, long-term internal drainage was substantially important. Approximately 30 % of patients were discharged with an AxiosTM stent (Boston Scientific) and 60 % with pigtail drainages in situ. Complications such as bleeding or perforation occurred in 8 cases (7.6 %). Eleven patients underwent surgical intervention (rate, 10.5 %). Mortality was 7.6 % (n=8). The stents were removed after 3 months. There was a recurrence of cyst or WON in 8 patients (rate, 7.6 %), only 4 patients (3.8 %) needed endoscopic re-treatment.

**Conclusion:**

Step-up approach to be used as the basic algorithm in acute necrotizing pancreatitis comprising external drainage, internal/external drainage (+ rinsing via drainage) and endoscopic necrectomy appears feasible, efficient and safe. In case of infected necrosis, intensive care and antibiotic treatment are as important as the drainage of the WON. In septic patients, percutaneous drainage is preferred. In stable patients, internal drainage or the combination of internal and percutaneous drainage was/were used. The endoscopic necrectomy was performed if the clinical status had not improved despite a sufficient internal and external drainage. The complete necrectomy is not an indispensable prerequisite for a favorable outcome but, however, the sufficient long-term drainage is essential.

## DGAV: Metabolic surgery / Bariatric surgery

### The effect of bariatric surgery on the direct oral anticoagulant rivaroxaban: the extension study

(Abstract ID: 62)

D. Kröll^1^, Y. Borbèly^1^, P. Nett^1^, D. Bertaggia^1^, L. Alberio^1^, D. Candinas^1^, G. Stirnimann^1^

^1^*University Hospital of Bern, Inselspital, Bern*

**Background:**

Thromboembolic disease is a potentially serious complication in bariatric surgery patients. Direct oral anticoagulants (DOAC) have been investigated in orthopedic surgery patients. DOAC data after bariatric surgery is still limited to the early postsurgical period. Whether postsurgical mid-term adaptations due to anatomical and physiological alterations influence drug pharmacology is currently not known. The aim of this study was to investigate the influence of weight loss and type of bariatric surgery on mid-term postsurgical pharmacokinetic and pharmacodynamic parameters of rivaroxaban.

**Materials and methods:**

In this monocentric study, bariatric patients received a single oral dose of rivaroxaban (10 mg) six to eight months after Sleeve Gastrectomy (SG) or Roux-en-Y-gastric bypass (RYGB). Pharmacokinetic and pharmacodynamic parameters were assessed and compared with pre-bariatric surgery results.

**Results:**

We included 6 RYGB and 6 SG patients. Percent excess weight loss (%EWL) was 71.4% (interquartile range 56.4, 87.9) in the SG group and 76.6% (64.5, 85.7) in the RYGB group. Rivaroxaban mean areas under the curve (AUC) 6 to 8 months after the bariatric procedure (922.4 μg*h/L, coefficient of variation 43.2) were comparable to those measured preoperatively (952.6.4 μg*h/L, 16.8). There was no relevant difference between the two surgical procedure groups. Rivaroxaban led to a decrease of prothrombin fragments F1+2 over 12 h after oral intake confirming in vivo efficacy.

**Conclusion:**

Significant weight loss and altered anatomy after RYGB and SG procedures do not appear to affect the pharmacokinetics and pharmacodynamics of prophylactic rivaroxaban. A single dose of Rivaroxaban was well tolerated and considered safe in this trial.

### Is the sleeve gastrectomy feasible in an outpatient / day surgery concept? Results of a pilot study. First experience in Germany

(Abstract ID: 164)

A. Georgiev^1^, O. El-Zaidi^1^, V. Christogianni^1^, R. Dukovska^1^, M. Büsing^1^

^1^*Knappschaftkrankenhaus Recklinghausen*

**Background:**

Bariatric interventions are generally performed in full inpatient settings in Germany with a residence time of 2-7 days. For the gastric band system, which is hardly pursued today, we were able to establish a purely outpatient treatment in a previous examination (> 100 cases). In the meantime, the first publications from the USA, France and Canada show that sleeve gastrectomy in selected patients can also be carried out on an outpatient basis without increased risk. By the very low rate of complications of the procedure, the question arises as to whether sleeve gastrectomy can also be successfully performed in Germany as an outpatient / day surgery operation. As part of the surgical preparation, individual patients ask the question about an ambulatory sleeve gastrectomy.

**Materials and methods:**

According to the patient's request, an ambulatory sleeve gastrectomy was considered according to the following criteria. ASA I (normal, otherwise healthy patient), BMI 40 kg / m2 (+/- 5 kg / m2), adult accompanying person, stay postoperatively within 20 km radius of the hospital, possibility to search the hospital at any time and the outpatient check-up on the following day. Telephone accessibility over 24 hours. Operative and postoperative setting: opiate-free anesthesia and postoperative analgesia, uncomplicated intraoperative course. Unremarkable postoperative course (Hb, O2 saturation), low analgesic demand, mobilization, undisturbed oral fluid intake (> 500ml), continuing patient request for outpatient surgery. Participation in a patient survey.

**Results:**

The inclusion criteria were met by 14 patients aged 21-57 years. In 13 cases, the sleeve gastrectomy was performed as bariatric first intervention. In one case, implantation of gastric banding and removal had preceded. The intraoperative and postoperative criteria were met by all patients. All patients were discharched after 7-12 hrs. The planned outpatient check-ups were made, in-patient admissions were in no case necessary. According to the survey, all of the patients would be ready for an outpatient sleeve gastrectomy again.

**Conclusion:**

There is an increasing interest of the patients to have sleeve gastrectomy performed on an outpatient basis in Germany. Larger studies, such as in Canada (n = 300 cases) were able to prove the safe practicability of the sleeve gastrectomy in a selected patient population. Unplanned hospitalizations were required in about 10% of cases. Our pilot study proves that, with appropriate patient selection and undisturbed postoperative course, sleeve gastrectomy can be performed on an outpatient basis (up to 12 hours) or in a day surgery concept (up to 24 hours).

### Markers for septic complication after bariatric surgery

(Abstract ID: 250)

J. Drozdzynski^1^, A. Bernsmeier^1^, J.-N. Kersebaum^1^, F. Richter^1^, M. Ahrens^1^, C. Schafmayer^1^, T. Becker^1^, J. H. Beckmann^1^

^1^*Universitätsklinikum Schleswig-Holstein, Kiel*

**Background:**

Bariatric surgery is an integral part in the treatment of obesity. Beside a significant weight reduction, obesity surgery leads to a significant improvement of co-morbidities and quality of life. Surgical procedures are highly standardized while complication rates are very low. The early detection of septic complications is essential in order to be able to react in time if necessary. Furthermore, the safe exclusion of possible complications would be important in allowing patients to be discharged promptly for further outpatient care. Postoperative parameters such as CRP and heart rate are used in everyday clinical practice with still unclear validity.

**Materials and methods:**

Retrospective analysis of the postoperative course of 617 patients who underwent bariatric surgery at the University Hospital Schleswig-Holstein Campus Kiel between 2007 and 2015. Laboratory and clinical controls were performed on days 1 and 2. C-reactive proteins, leukocytes and heart rate were investigated with regard to septic postoperative complications and the occurrence of staple line leaks and anastomotic insufficiencies.

**Results:**

In the cohort, 25 patients experienced a staple line leak or anastomosis insufficiency (4%). The ROC curve analysis shows that the CRP value on day 2 is formally very well qualified as a parameter to detect or exclude these complications (AUC 0.858). A sensitivity of 71% and a specificity of 82% could be achieved with a cut off level of the CRP value of 100 mg/l. Heart rate, on the other hand, proved to be unsuitable as a parameter to detect septic complications or insufficiencies (AUC 0.573).

**Table:** 

anastomotic or staple line leak	n	CRP d2/ mg/l	WBC d2/ mm3	HF d2/ bpm
none	592	69,3±49,9	9,4±2,6	80,1±10,5
yes	25	204,0±115,3	12,6±5,3	87,2±20,2
	617	0,000031*	0,013975*	0,133148*

*Comparisons of variables were performed with Student’s t-test. A P value < 0.05 was considered statistically significant.

**Conclusion:**

The use of laboratory parameters such as CRP values is in fact a useful method for identifying septic complications. A high CRP value should increase the attention of the attending physicians and possibly lead to further examinations up to diagnostic laparoscopy. Due to the generally low probability of insufficiencies, however, a positive predictive value of 20% and a negative predictive value of 98% with a 96% probability of uncomplicated courses are not pathbreaking.

An isolated evaluation of individual postoperative parameters for the recognition or exclusion of postoperative insufficiencies after bariatric interventions is not recommended. The clinical view of the treating physician under evaluation of laboratory and clinical parameters is crucial.

### New tailored transgastric approaches to access the hepatobiliary system in patients after bariatric surgery – a tertiary care bariatric center experience

(Abstract ID: 486)

D. Kröll^1^, A. Müller^1^, Y. Borbèly^1^, P. Nett^1^, D. Candinas^1^, J. Maubach^1^, R. Wiest^1^

^1^*University Hospital of Bern, Inselspital, Bern*

**Background:**

In bariatric surgery patients, pancreaticobiliary access via endoscopic retrograde cholangiopancreatography (ERCP) is technically challenging. The optimal approach for evaluation and treatment (e.g., of biliary stones) in these patients has been debated. Laparoscopy assisted ERCP (LA-ERCP) as a standard of care and EUS-directed gastroenterostomy (EDGE) or hepaticogastrostomy (HGS) by placement of a temporary lumen-apposing stent as novel techniques have been described.The objective of this study was to evaluate safety and efficacy of three different endoscopic approaches (LA-ERCP and ultrasound (EUS) guided transgastric ERCP) supported by conventional duodenoscope for the treatment of biliary diseases in bariatric patients.

**Materials and methods:**

In this retrospective review, consecutive patients with Roux-en-Y Gastric bypass (RYGB), Sleeve gastrectomy (SG) or biliopancreatic diversion (BPD) who underwent an LA-ERCP, EDGE or a HGS at a tertiary care reference center for bariatric surgery from 2013 to 2018. Patient demographics, type of procedure and indication, data regarding cannulation and therapeutic intervention of the common bile duct (procedure success) and clinical outcomes were extracted.

**Results:**

A total of 16 patients were included. Indications for LA-ERC, EDGE or HGS were mostly choledocholithiasis (77%), and in a few cases papillitis stenosans. Eight patients underwent concomitant cholecystectomy combined with LA-ERC. Procedure success was achieved in 100%. Moderate adverse events were identified in 18.7% of patients (all ERC related).

**Conclusion:**

This case series indicates that ERCP via a transgastric approach (LA-ERCP or EUS-guided EDGE or HGS) is a minimally invasive, effective and safe method to access the biliary tree in patients after bariatric surgery. These techniques offer an appealing alternative way of treatment compared to percutaneous transhepatic cholangiography and drainage or deep enteroscopy assisted ERCP. In bariatric patients who had a cholecystectomy, EUS-guided techniques are the preferred treatment options for biliary indications in our center.

### Gastrobronchial fistula after Sleeve Gastrectomy: 3 different approaches

(Abstract ID: 639)

V. Christogianni^1^, R. Dukovska^1^, J. C. Halter^1^, P. Bemponis^1^, R. Riege^1^, K. A. Husemeyer^1^, M. Reiser^2^, M. Büsing^1^

^1^*Knappschaftkrankenhaus Recklinghausen, Recklinghausen*

^2^*Klinikum Vest-Paracelsus Klinik Marl*

**Background:**

Gastrobronchial fistula after bariatric surgery is a rare late complication, whose treatment is very challenging and usually demands an operative and endoscopic approach. Due to a proximate staple line leak which is not sufficiently treated, a chronic subphrenic collection can be formed, resulting in some cases in the break of the diaphragm barrier and evoking in alterations of the respiratory tract.

**Materials and methods:**

We present three female patients, who underwent a sleeve gastrectomy in external hospitals and were transferred to us in order to treat a proximal staple line leak. Demographics, previous surgeries, clinical presentation, time of the diagnosis of the fistula, treatment methods and outcome were presented.

**Results:**

All of the patients were female the average age was 32,33 years and the average bmi at the time of the gb fistula was 27. In all patients a laparoscopic sleeve gastrectomy was performed due to morbid obesity with a resulting proximal staple line leak. One patient had undergone a previously mason operation with unsatisfactory results concerning the weight loss. In only one patient a diagnostic laparoscopy to drain the abdominal cavity after the diagnosis of the leak was performed. The average time of the diagnosis of the gb fistula was 14,33 months, although there was a wide range of occurrence from 3 to 36 months. All of the patients showed in the ct scan a fluid selection on the upper left abdominal cavity. In the youngest patient the fluid selection was persistent since the diagnosis of the proximal staple line leak for at least 2 years prior to the the diagnosis of the gb fistula and no further action was undertaken due to lack of symptoms. Productive cough and fever were the two major symptoms. All patients were treated with a combined endoscopic and operativ approach-including endo vac therapy, endoscopic ballon dilation and fibrin glue as also laparoscopy/laparotomy, abscess drainage (3 patients ) and bypass operation (2 patients), thoracoscopy and thoracotomy with atypical left lower lobectomy. Treatment was successful in one case in 1 month, with a resulting chronic fistula in the other two. No mortalities were reported.

**Conclusion:**

Gastrobronchial Fistula is a severe mostly late complication after sleeve gastrectomy. In our series a combination of both endoscopic and operativ approach was needed in order to initiate the healing process. The late time of the diagnosis usually results in a prolognated hospitalization.

### Long-term follow-up of biliopancreatic diversion with duodenal switch in super obese patients older than 60 years of age

(Abstract ID: 649)

P. Nett^1^, D. Kröll^1^, Y. Borbély^1^, D. Candinas^1^

^1^*Universitätsspital Bern*

**Background:**

Indications and long-term outcome of biliopancreatic diversion with duodenal switch (BPD-DS) in older adults suffering from super obesity remain controversial. The aim of this study was to evaluate safety and long-term outcomes of this bariatric procedure in older patients with super obesity.

**Materials and methods:**

Patients aged >=60 years who underwent (BPD-DS) between January 2001 and December 2011 were included and had at least 5 years of follow-up. This is a single-center retrospective study of a prospectively collected database (University Hospital, Switzerland).

**Results:**

Of all 246 patients undergoing (BPD-DS), 23 patients (9%) were older than 60 years and super-obese (body mass index; BMI>50 kg/m2). The majority of the procedures were laparoscopic with two conversions (9%) to open surgery. Almost all patients (19/23; 83%) underwent a concomitant cholecystectomy. Mean age and BMI were 62.8±3.1 years and 55.2±7.9kg/m2, respectively. Average follow-up time after BPD-DS was 96.8±31.3 months. At baseline, 86% (20/23) of the patients had arterial hypertension, 74% (17/23) had type 2 diabetes mellitus, and 43% (10/23) had obstructive sleep apnea syndrome. There was no 30-day mortality. Complication rate (Dindo-Clavien category 3 and 4) was 9% (2/23): one leak that could be managed conservatively and one bleeding requiring transfusion. Mean percent excess weight loss (%EWL) at 2, 5 and 10 years after BPD-DS was 55.7±28.2, 57.3±22.1, and 51.9±31.4. Remission rates of arterial hypertension, type-2 diabetes mellitus, and obstructive sleep apnea syndrome were 22%, 47%, and 28% after 5 years, respectively.

**Conclusion:**

BPD-DS is safe and effective in improving obesity-related comorbidities in older patients suffering from super obesity. Age alone should not preclude older patients from getting the best bariatric procedure for obesity and its related comorbidities.

### Secondary hyperparathyroidism and vitamin D insuffiency after biliopancreatic diversion with duodenal switch for morbid obesity: An underestimated phenomenon?

(Abstract ID: 653)

P. Nett^1^, Y. Borbély^1^, D. Kröll^1^, D. Candinas^1^

^1^*Universitätsspital Bern*

**Background:**

Biliopancreatic diversion with duodenal switch (BPD-DS) is considered to be one of the most effective bariatric procedures resulting in a sustainable long-term weight loss and a high remission rate of obesity-related comorbidities. Besides its excellent long-term outcome, BPD-DS can lead to severe diarrhea and micronutrient deficiencies in the long-term based on the malabsorptive character of the procedure. Therefore, the risk for secondary hyperparathyroidism due to malabsorption needs to be determined in this population.

**Materials and methods:**

Data from all 246 patients undergoing BPD-DS between January 2001 and December 2011 were prospectively collected (University Hospital, Switzerland). Life-long micronutrient supplementation consisted according to the American Society for Metabolic and Bariatric Surgery (ASMBS) guidelines of a multivitamin-mineral supplementation on a daily base covering 200% of the daily value. It contained 2’000 IU of vitamin D3, and 2’400 mg of calcium.

**Results:**

Of all 246 patients, 195 (79.3%) had at least 5 years of follow-up. The majority of the procedures were laparoscopic. Almost all patients (168/195; 86.2%) underwent a concomitant cholecystectomy. Mean age and BMI were 42.8±9.2 years and 48.3±9.1kg/m2, respectively. Average follow-up time after BPD-DS was 85.8±35.9 months. Of 195 patients, 102 (52.3%) showed laboratory signs of a secondary hyperparathyroidism during the follow-up. Although vitamin D levels improved with increased vitamin D3 supplementation in 2007, the rate of secondary hyperparathyroidism increased.

**Conclusion:**

Despite routine postoperative calcium and vitamin D3 supplementation, secondary hyperparathyroidism is common after BPD-DS. These rates suggest current supplementation guidelines are not sufficient in preventing secondary hyperparathyroidism. Further work is needed to better define the sequelae of long-term hyperparathyroidism.

### SADI-S – Single Anastomosis Duodeno-Ileal Bypass with Sleeve Gastrectomy – two stage or Single-Step procedure for Super-Superobese Patients – technical aspects

(Abstract ID: 803)

R. Zorron^1^, B. Ruschen^1^, V. Atanassov^1^, M. Specht^1^

^1^*Klinikum Ernst von Bergmann Potsdam, Berlin*

**Background:**

Super-Superobese patients (BMI higher then 60kg/m2) are of difficult primary bariatric management. Issues regarding exposure and technical difficult anastomosis led to the choice of a 2-stage procedure, usually a sleeve gastrectomy as the first step. In the majority of cases, patients have an unsatisfactory EWL (excess weight loss) after 2 years and high rates still remain in obesity class III.

**Materials and methods:**

Patients with BMI over 60kg/m2 without esophageal reflux symptomatic were scheduled for a single-step or two-stage SADI-S procedure. The video describes the technical steps of the procedure, and identified key issues to perform this technique safely. 1. Position: Patient supine, the surgeon stands at the right side to perform sleeve resection, switches to the left side to perform the anastomosis. 2. Six trocars were inserted. 3. The omentum is separated from the greater curvature close to the stomach using harmonic reaching the left crus and sleeve gastrectomy is performed over a 42-Fr Bougie. 4. First segment of the duodenum is stapled. 5. The small bowel loop for the anastomosis is identified going backwards 300cm from the ileocecal junction. 6. Anastomosis is performed in a 2 row fashion or using linear stapler. A drain is placed for the duodenal stump.

**Results:**

SADI-S was performed in this selected group of patients and the technical issues were identified. The anastomosis can be performed using the linear stapler when possible, or a two-layer hand sewing suture. Covering the sleeve staple line with a synthetic, bioabsorbable staple line reinforcement tissue is advised to avoid staple line complications.

**Conclusion:**

This new technique is a promising option in our surgical armamentarium to provide effective therapy for obesity patients classified as super-superobese. In the literature, performing SADI-S as a single procedure showed better EWL than patients submitted to 2 stage procedures.

### C-reactive protein is an early marker for leakage after obesity and metabolic surgery

(Abstract ID: 844)

P. Jamadar^1^, S. Chiappetta^1^, N. Runkel^1^

^1^*Sana Klinikum Offenbach*

**Background:**

Infectious complications are the most important cause of perioperative morbidity and mortality after obesity and metabolic surgery. Immediate diagnosis and therapy are mandatory to improve patients’ outcome.

The aim of this study was to analyze whether C-reactive protein (CRP) and leucocyte count (LC) are prognostic markers of infectious complications.

**Materials and methods:**

Patients who underwent laparoscopic sleeve gastrectomy (SG), Roux-en-Y gastric bypass (RYGB) or One anastomosis/Mini-gastric bypass (OAGB/MGB) as primary treatment for severe obesity were included. CRP in mg/l and LC in x10³/ μl were measured preoperatively, on postoperative days 1 (POD 1) and 4 (POD 4). The patients were retrospectively divided into an uncomplicated group and into an infectious group. Latter was defined by postoperative infectious complications according to the Clavien-Dindo Classification.

**Results:**

471 patients underwent SG (n = 237), RYGB (n = 153) or OAGB/MGB (n = 67). Fourteen patients (3.1%) had infectious complications, seven of which (1.5%) had leaks. LC and CRP increased in all patients postoperatively. On POD 1, CRP was 23 +/- 19.1 mg/l in uncomplicated and 66.3 +/- 68.3 mg/l in complicated patients (p = 0.0001), and LC was 11 x10³ +/- 5.6 in uncomplicated patients and 12.1 +/- 5 / μl in complicated patients (p = 0.4682). On POD 4, CRP was 41.1 +/- 26.2 mg/l in uncomplicated and 154.3 +/- 118.1 mg/l in complicated patients (p = 0.0001), and LC was 8.2 x10³ +/- 6 in uncomplicated patients and 9.8 +/- 4.8 / μl in complicated patients (p = 0.3238). Mean CRP in the leakage group was 93.6 +/- 82.2 mg/l on POD 1 and 284.3 +/- 78.2 mg/l on POD 4. On analysis of ROC and AUC curves, the cut-off for CRP on POD 1 was 94.5 mg/l, with 56 % sensitivity and 99% specificity and the cut-off for CRP on POD 4 was 149.5 mg/l, with 50 % sensitivity and 99.6 % specificity for leakage.

**Conclusion:**

Both, CRP and LC increased postoperatively, but only CRP showed a significant difference between the complicated and uncomplicated group. Only a high CRP is predicative of infectious complications. A CRP >= 94.5 mg/l on POD 1 or >= 149.5 mg/l on POD 4 is highly suspicious of leakage and should prompt immediate clinical action.

## DGAV: MIS/Robotics

### True single port cholecystectomy with ICG Cholangiography through only one 15 mm Trocar using the new surgical platform „SymphonX“ – first human case series

(Abstract ID: 180)

R. R. Datta^1^, G. Dieplinger^1^, P. Plum^1^, R. Kleinert^1^, R. Wahba^1^, F. Gebauer^1^, D. Stippel^1^, C. J. Bruns^1^, H. Fuchs^1^

^1^*Uniklink Köln*

**Background:**

Minimally invasive single port surgery was associated with large incisions up to 2-3cm, complicated handling due to the lack of triangulation, and instrument crossing. Aim of this prospective study was to perform true singleport surgery (cholecystectomy) without the use of assisting trocars using a new surgical platform that allows for triangulation incorporating robotic features and to measure the perioperative outcome and cosmetic results.

**Materials and methods:**

The new technology has been introduced to our academic center as first European site after FDA and CE clearance. In patients with cholecystitis and cholecystolithiasis, the operation is performed through only one 15mm trocar. For patients safety, intraoperative cholangiography using intravenous ICG and a standard Stryker 1588 5mm camera was performed.

**Results:**

Symphonx was used in n=10 patients for abdominal surgery (4 females, median age 45.2 [32-77], median BMI 32.7 [29-35]. A total of 4 patients underwent surgery using no additional than the 15mm trocar; in the remaining patients, one assisting instrument (3-5mm) was used. Mean OR time was 103.6 [83-122] minutes. The postoperative course was uneventful in 9 patients, one patient required postoperative interventional drainage of a bilioma 1 month postoperatively. The cosmetic result was excellent. No intraoperative complications occurred.

**Conclusion:**

This is the first human case series using the SymphonX platform for abdominal surgery without assisting instruments.

Laparoscopic cholecystectomy in patients with cholecystitis and cholecystolithiasis using the symphonx platform through only one 15mm trocar is feasible and safe. The cosmetic result is promising. Further recruiting of patients for validation of the new technology is necessary and ongoing.

**Picture:**
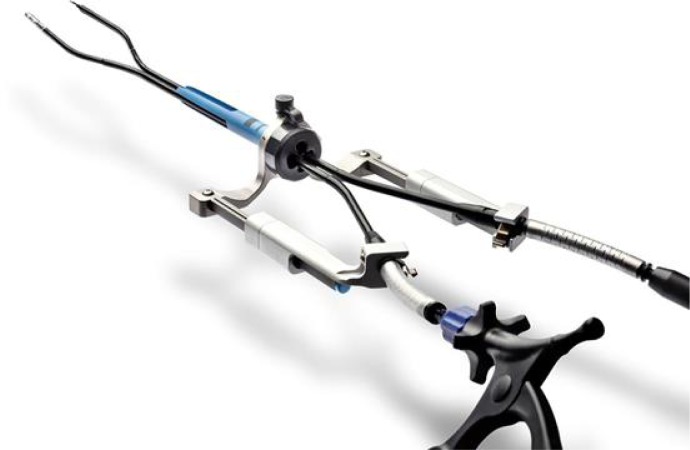
Fortimedix System

### Minimally invasive Ivor Lewis esophagectomy with robotic assistance and ICG fluorescence – modular step up approach and two anastomotic techniques

(Abstract ID: 205)

H. Fuchs^1^, W. Schröder^1^, F. Gebauer^1^, J. Leers^1^, C. J. Bruns^1^

^1^*Uniklinik Köln*

**Background:**

Minimally invasive technologies have improved outcomes after esophagectomy and the use of robotic technology in Europe is rapidly increasing. Aim of this study is to evaluate the introduction of new technologies in a center of excellence for upper gastrointestinal surgery.

**Materials and methods:**

A standardized teaching protocol of a complete OR team was performed in simulation and animal models at the center for the future of surgery (San Diego, CA) and IRCAD (Strasbourg, France) to receive certification as console surgeons. Starting 02/2017 the davinci xi and stryker ICG laparoscopy systems were introduced at our academic center (certified center of excellence for surgery of the upper gastrointestinal tract, n>300 esophageal cases/year). After simple training procedures based on our minimally invasive expertise were performed, difficulty was increased based on a modular step up approach to safely perform robotic assisted Ivor Lewis esophagectomy.

**Results:**

From 02/2017 - 09/2018, a total of 50 upper gastrointestinal robotic cases including 30 esophagectomies for cancer were performed. All cases were performed safely without operation-associated complications. Level of difficulty was increased based on our modular step up approach without quality compromises. Video documentation using the new technology is provided and two anastomotic techniques are presented.

**Conclusion:**

The standardized training protocol and our modular step up approach allowed safe introduction of the new technology used. All cases were performed safely without operation-associated complications.

**Picture:**
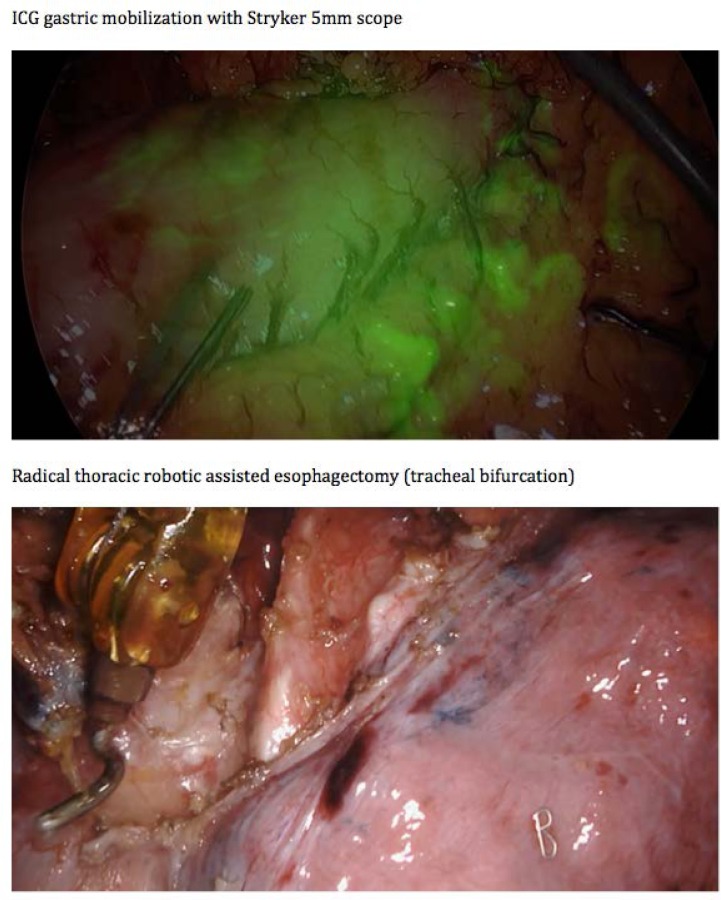


### Which factors attribute to the surgeons' learning curve in robotic general surgery?

(Abstract ID: 347)

K. Schulte^1^, M. Heise^1^

^1^*Sana Klinikum Lichtenberg, Berlin*

**Background:**

Robotic adult general surgery is confined to specialist centres. It is seen as a significant future development of surgery. However, it has cost implications, potential patient benefits are unclear and there are medico-legal implications with a new technique when there are established data on outcomes with open surgery. We sought to determine what factors attribute to the learning curve for robotic surgery.

**Materials and methods:**

Numerous studies evaluated surgeons learning curves from 1999-2018 in robotic in colorectal, bariatric, biliary and solid organ surgery (right/left hemicolectomy, pancreatectomy, cholecystectomy). A total of ten studies were included describing potential factors impacting surgeons’ learning curves.

**Results:**

The studies demonstrated that there is a significant learning curve in the performance of robotic general surgery, especially during the first 20 patients.

One study showed that users with more than 20-hours of experience performed significantly better in a robotic training model than novices with less than two hours of experience, independent of prior experience.

One study demonstrated a significant time reductions of robotic hemicolectomy time during the first ten cases to a 50% decrease after 30 cases (P=0.001).

Three studies mentioned the importance of stepwise training programmes and simulators in the development of robotic skills.

**Conclusion:**

Significant learning curve improvements are achieved within the first 20 cases. The training of young surgeons in simulated scenarios and in a stepwise fashion can avoid the complications encountered during the introduction of robotic surgery.

### Hyperspectral Imaging: A novel imaging technique for perfusion measurement in esophagectomy

(Abstract ID: 429)

A. Studier-Fischer^1^, F. M. Schwab^1^, K.-F. Kowalewski^1^, B. P. Müller-Stich^1^, F. Nickel^1^

^1^*Universitätsklinikum Heidelberg*

**Background:**

Anastomic insufficiency results in high morbidity and mortality after esophagectomy and is therefore challenging surgeons worldwide. This is not merely due to technical difficulties regarding surgical experience and anatomical accessibility, but rather a consequence of the ambiguity regarding anastomic blood supply and healing tendencies. Hence, we present the development and usage of augmented hyperspectral imaging (AHSI) as well as first results of its application in esophagectomy.

**Materials and methods:**

The TIVITA^™^ tissue system Hyperspectral Imaging (HSI) Camera was used in a porcine model. An esophagectomy was performed in minimally invasive technique, but with open approach for reasons of standardization. After laparotomy, a gastric conduit was formed with linear staplers. Through small incisions magnets were inserted into the conduit. The size and position of these magnets were identical to the linear stapler that is usually used for the thoracic anastomosis (Fig.1f) and they allowed for reversible ischemia evaluation.

For the purpose of this study, the O2 saturation index was measured. Conventional HSI - lacking any harmful radiation or contrast agent - was combined with fluorescence-guided surgery (FGS) in that additional dyes such as indocyanin green (ICG) were applied in order to gain extended tissue evaluation through AHSI. The coding language Python enables to calculate dye-specific indices beyond conventional HSI. In order to correlate the obtained graphical data to clinical outcome, lactate and lactate dehydrogenase - as marker for ischemic areas - were quantified in blood samples drawn from corresponding regions.

**Results:**

There are 2 hypotheses regarding the blood supply of the gastric conduit. Hypothesized blood supply type I is trans-mucosal. Consequently a wider gastric conduit and a rather aborally stapling position (>3 cm to oral margin) would be beneficial (Fig.1b). Hypothesized blood supply type II is trans-arterial (A. gastroepipl. dextra), traveling sideways from the curvatura major towards the former curvatura minor and resulting in the opposite conclusion (Fig.1c).

After 2 minutes of artificial ischemia, O2 saturation dropped significantly on the left side of the magnets. Further insights exceeding this abstract also support a trans-arterial supply. These preliminary results in 4 animals will be evaluated in continuation with a larger number of experimental studies.

**Conclusion:**

Blood supply of the gastric conduit is mainly provided through the A. gastroepiploica dextra and advocates for a thin gastric conduit with an aborally linear stapling position for esophageal reconstruction.

**Picture:**
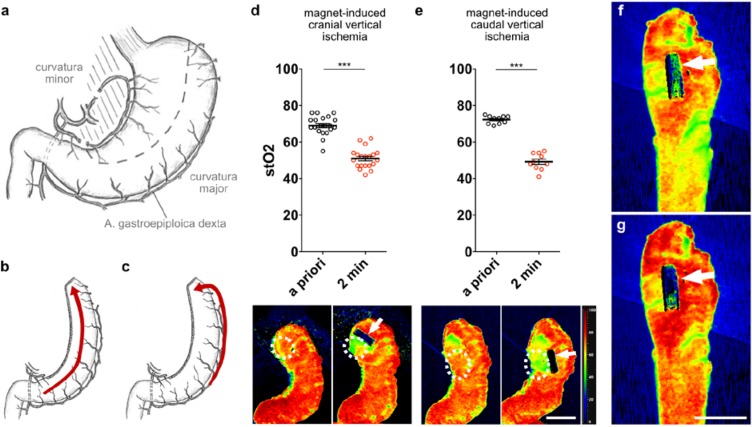
HSI for esophagectomy

### 3D versus 2D visualization and its influences on the technical and manual surgical performance in a robotic simulator

(Abstract ID: 459)

K. Wierschula^1^, OJ. Muensterer^1^

^1^*Universitätsmedizin Mainz*

**Background:**

The medical sector is an innovative field in which progresses are made every day. In conventional 2D laparoscopic surgery surgeons often struggle with the disadvantage of a loss of depth perception. The growing development of 3D laparoscopic equipment and the industry who advertise the advantages of the new method lead us to the important question if the 3D technique is superior to the conventional 2D systems. We wanted quantitatively compare the effects of a 3D sight with the conventional 2D laparoscopic optics with regard to the potential benefits of the factors time and manual surgical performance.

**Materials and methods:**

For this prospective study we used the dV Trainer of Mimic Technologies, Inc. One group of 25 participants who passed three exercises with six repetitions, three with 3D technique and three with 2D optics. The participants were randomized to start either with 3D optics or 2D technique and alternating the optics between 3D and 2D after every exercise. At the end of the exercise the Computer of dV Trainer gave us an evaluation of the completed exercise. This evaluation included the important factors time (in seconds), economy of motion (in centimeters) and master workspace range (in centimeters) and other factors, these were includes in the calculation of the Overall Score. We used the Overall Score for the valuation of the participant´s performance.

**Results:**

The results showed a superiority of the new 3D technique over the conventional 2D system. The exercises were performed faster, with less problems regarding instrument collisions or instruments out of view and with a better manual performance. The Overall Score was significantly higher with 3D optics than with 2D (exercise Pick and Place p 0.0023, average 2D 742,26, average 3D 885,33; exercise Ring Walk p 0.00052, average 2D 443,53, average 3D 567,05; exercise Stacking Challenge p 0.0008, average 2D 33,43, average 3D 46,38).

**Conclusion:**

Similar to other publication our results showed a superiority of the 3D technique over 2D conventional laparoscopic optics. However past studies about the new technical progresses with 3D laparoscopic optics, including our own study, had a limited number of participants. The results of this study can be seen as a first step of the evaluation of new systems, however studies with larger populations of participants, with different qualifications, are necessary for a correct and reliable outcome.

### Comparison of a surgeon-controlled robotic camera holder with conventional assistants in simulated standard- and exploratory procedures

(Abstract ID: 488)

J. Bucher^1^, K. Bruewer^2^, L. J. Dietz^3^, J. Hidding^3^, N. Trebesius^3^, M. Schoenberg^3^, J. Werner^3^, K. Karcz^3^

^1^*Klinikum der Universität München Campus Großhadern, München*

^2^*Helios Klinikum Dachau*

^3^*Uniklinik München*

**Background:**

Cost pressure in the medical sector requires new strategies to economize surgical procedures. Robotic camera steering devices (RCSDs) are designed to replace the human camera assistant in minimally invasive procedures and thus might facilitate "solo surgery". Clinical experience shows that standard laparoscopic procedures can safely be performed as solo-surgery aided by RCSDs. More and more hospitals reduce surgical staff during nights or on weekends and might start using RCSDs to perform exploratory laparoscopies solo. Most RCSDs require active steering by the surgeon and necessarily increase workload. No evidence exists concerning exploratory or emergency procedures with RCSDs.

**Materials and methods:**

45 novice participants were trained according to the FLS-curriculum before entering the study and then were randomized into two groups. We compared the performance during "exploratory" laparoscopic tasks on box-trainers aided by an RCSD controllable by head-movements or by human camera assistants.

**Results:**

Performance in simulated standard procedures was comparable. In simulated exploratory-procedures, we saw significantly better performance-scores in the conventional-group vs. RCSD-group. The strongest factor for these differences was the longer camera-adjusting time in the RCSD-group vs. conventional-group (PEG-task: 208±51sec vs. 170±36sec, p=0.005; suture-task: 563±126sec vs. 454±201sec, p=0.041).

**Conclusion:**

These results, obtained on surgical simulators indicate, that the solo-approach to standard surgical tasks, facilitated by an RCSD controllable by head-movements can most likely be viewed as safe. Exploratory procedures with a relevant chance for complications or procedures that require rapid and complex camera movements should rather be performed with a human camera assistant.

### The bottom-to-up approach in robotic right colectomy plus complete mesocolic excision (CME) – a video vignette

(Abstract ID: 613)

J. Schulte am Esch^1^, S.-I. Iosivan^1^, T. Kolokotronis^1^, F. Gutierrez^1^, C. Förster^2^, A. Nasser^1^, T. Benhidjeb^1^, H. Sarikaya^1^

^1^*Evangelisches Klinikum Bethel, Bielefeld*

^2^*KRH Klinikum Nordstadt, Hannover*

**Background:**

We present and discuss technical advances with the novel bottom-to-up robotic right colectomy plus CME utilizing suprapubic port placement on the basis of a video presentation.

**Materials and methods:**

Using a representative example of the procedure advances and pitfalls of the technique will be highlighted based on our experience with 25 consecutive cases performed with this robotic technique of RC. The 4-step of the procedure facilitate a dissection guided by embryonal planes rather than searching those planes as will be become clear in the course of this video presentation.

**Results:**

The substrate for improvements with the presented approach including oncological aspects like enhanced yield in lymph nodes if contrasted to classic medial to lateral approach will be imparted.

**Conclusion:**

The here presented standardized robotic four-step approach of RC combined with CME may bear the potential of exceeding a minimal invasive technique of RC from the stage of ‘being easier than laparoscopy’ to an oncological advanced concept.

### Transoral endoscopic thyroid surgery via vestibular approach (TOETVA) – two year experience, technical details and tips

(Abstract ID: 622)

E. Karakas^1^, A. Anuwong^2^, K. Ketwong^2^, A. Kounnamas^1^, S. Schopf^3^, P. Kühn^1^, G. Klein^4^

^1^*Krankenhaus Maria Hilf, Alexianer GmbH, Krefeld*

^2^*Police Hospita , Bangkok*

^3^*Krankenhaus Agatharied, Hausham*

^4^*Landesklinikum Wiener Neustadt*

**Background:**

Transoral (para-)thyroid surgery was first described by German study groups. Meanwhile and optimized Transoral Endoscopic Vestibular Approach (TOETVA) has been implemented by Anuwong. We report on our two year experience in Austria and Germany and will present a video of the minimally invasive technique.

**Materials and methods:**

TOETVA was implemented by our Austrian German study group in June 2017 supported by Dr. Anuwong. Ince then we operated on patients with single thyroid nodules, sporadic primary hyperparathyroidism or thyroglossal duct cyst. TOETVA was performed using 3 laparoscopic ports, laparoscopic instruments and ultrasonic or bipolar devices inserted at the oral vestibule. Surgical outcome and complications were evaluated.

**Results:**

Until now, no conversion to open surgery was necessary. Average tumour size was 2.1cm. Temporary hoarseness occurred in two patients. No mental nerve injury occurred. Transient hypoparathyroidism was evident after successful parathyroidectomy and in one patient after thyroidectomy. 15 patients developed a slight postoperative chin hematoma. No infection occurred.

**Conclusion:**

TOETVA is feasible and safe. The transoral approach shows promise for patients who are motivated to avoid a visible neck scar. After successful implementation in Austria and Germany further transoral operations are destined in selected patients.

### Improved lymph node yield with bottom-to-up vs. medial-to-lateral approach in robotic right colectomy plus complete mesocolic excision (CME) – an experience from 32 consecutive cases

(Abstract ID: 625)

J. Schulte am Esch^1^, S.-I. Iosivan^1^, L. M. Jünemann^1^, T. Kolokotronis^1^, C. Förster^1^, A. Nasser^1^, H. Sarikaya^1^, T. Benhidjeb^1^

^1^*Evangelisches Klinikum Bethel, Bielefeld*

**Background:**

Several studies have demonstrated a direct correlation between lymph node yield and survival after colectomy for cancer. Complete mesocolic excision (CME) in right colectomy (RC) reduces local recurrence but is technically demanding. Here we present and discuss technical advances with bottom-to-up robotic right colectomy plus CME utilizing suprapubic port placement and compare results to the classical medial-to-lateral approach.

**Materials and methods:**

32 consecutive patients (median age 75y (51-90y)) following robotic right colectomy with oncological intention to treat and admitted at our center from 5/2016 to 8/2018 were analysed in this retrospective study. Surgery was realized with the DaVinci Xi^®^ system placing the 4 robotic trocars in suprapubic position along a horizontal line plus 1 assist trocar in the left lateral abdomen. Patients were divided in group A with medial-to-lateral (n=7) and group B with bottom-to-up approach (n=25). In group A the right colon was initially dissected on the fascia of Toldt after incision of the peritoneum at the origin of the right mesocolon medial of the supra mesenteric vessels followed by transection of the ileo-colic vessels plus lateral dissection of the peritoneal fixation of the coecum and descending colon. In group B, we applied the novel robotic 4-step bottom-to-up approach of RC guided by embryonal planes in the process of retrocolic mobilization with suprapubic port placement. In step 1 CME was initiated with caudolateral mobilization of the right colon between the retrofascial and the mesofascial interface following Toldt’s fascia continued ventral of the duodenum and up to the Trunk of Henle. Subsequently, dissection was performed down-to-up right of the middle colic vessels with central vessel ligation (CVL) in step 2. Latter was eased by the orthograde view along the super mesenteric vessels. Subsequent to separation of the transverse retromesenteric space and mobilization of the hepatic flexure in step 3, the transverse mesocolon was transected right of the middle colic vessels in step 4. Ileo-transversostomies were performed in all cases side-to-side extra corporeally via the incision for specimen extraction in the left upper quadrant.

**Results:**

The two groups were comparable concerning age, co-morbidity (ASA), operating time, mayor complications, ICU and hospital stay respectively. We experienced no mortality, anastomotic leak or conversion in this patient cohort. We observed 1 trocar-incisional hernia and 1 post-OP blood-infusion-dependent anemia in each group, latter without relevant intra- or post-OP blood loss. 2 wound infections and 1 transient chylus fistula developed in group B. The yield in lymph nodes was superior in the bottom-to-up-group with a median of 34,0 nodes (14-86) in comparison to the medial-to-lateral-group with 14,0 nodes (9-29; p=0,002).

**Conclusion:**

The here presented standardized robotic four-step suprapubic approach combined with down-to-up mesocolic mobilization and subsequent CME plus CVL demonstrated to be safe even in rather elderly patients. The utilization of robotic systems in the bottom-to-up-technique may not just target a simplification of the minimally invasive procedure of RC. It may bear the potential of exceeding a minimal invasive technique of RC from the stage of ‘being easier than laparoscopy’ to an oncological advanced concept especially if compared to the ‘classical’ medial-to-lateral strategy. Robotic systems may provide the technical requirements to combine advantages of both open and minimal invasive surgical concept in oncologic RC.

**Picture:**
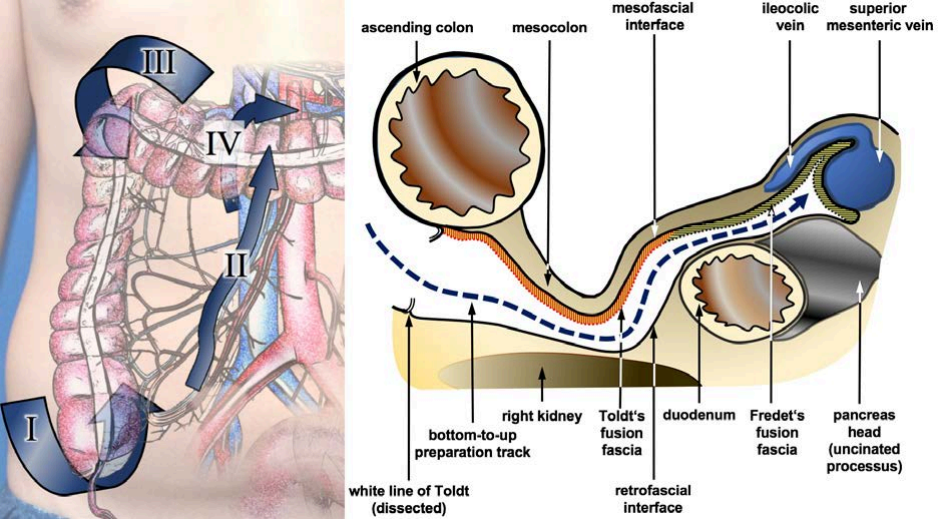
4 key steps in robotic RC with bottom-to-up-dissection, CME and CVL (left) and here relevant layers and structures of the retro-mesocolic space.

### Laparoscopic resection of a left upper quadrant mass leading to a surprise diagnosis

(Abstract ID: 687)

H. Bonatti^1^, R. Erlich^2^

^1^*Meritus, Hagerstown*

^2^*Memorial Sloan Kettering, Basking Ridge*

**Background:**

Resplenectomy is most commonly done for treatment of recurrent idiopathic thrombocytopenic purpura (ITP) refractory to medical management due to regrowth of a missed accessory spleen.

**Materials and methods:**

The clinical course of a 66 year old male who had undergone open splenectomy for traumatic rupture 40 years ago and developed a nodule close to the left adrenal gland while receiving chemotherapy including docetaxel/gemcitabine, pazopanib, eribulin, temozolomide, doxorubicin/olaratumab for metastatic leiomyosarcoma is described.

**Results:**

On surveillance CT-scan a 3.5cm mass compatible with a soft tissue tumor was found close to the tail of the pancreas. During laparoscopy dense adhesion of the omentum to the abdominal wall and stomach from his previous splenectomy were divided. The colon was identified, the lesser sack opened through the gastrocolic ligament and the splenic flexure was taken down. Superior and dorsal to the tail of the pancreas next to the left adrenal gland the mass was identified and carefully dissected out. The vascular pedicle, which originated from a side branch of the splenic artery and splenic vein at the tail of the pancreas was stapled. The gastric fundus showed multiple nodules and therefore, a modified sleeve gastrectomy was done; also a 2cm nodule in segment 5 of the liver and an omental nodule were removed. The tumors and gastrectomy specimen were placed into an endobag and removed through a periumbilical miniincision. The patient recovered well from the procedure without any complications. Pathology revealed no sarcoma metastases but accessory spleens in all specimens.

**Conclusion:**

Re-growth of splenic tissue after splenectomy for trauma is a rare condition but should be considered. This may lead to splenism with multiple implants within the abdomen. In our patient this seems to have been triggered by chemotherapy for his sarcoma resulting in extramedullary hemopoesis. Laparoscopic removal of accessory spleens can be safely done.

### Robotic-Hybrid vs Laparoscopic-Hybrid Esophagectomy: A Case-Matched Analysis

(Abstract ID: 864)

L. Giulini^1^, C. Keil^1^, A. Dubecz^1^, M. Papp^2^, H. J. Stein^1^

^1^*Klinikum Nürnberg Nord*

^2^*Universität für Veterinärmedizin Budapest*

**Background:**

Robotic-assisted esophagectomy for cancer has been increasingly employed world-wide, however, the benefits of this technique compared to laparoscopic assisted esophagectomy are unclear. Since 2016, robotic-hybrid (R-HMIE) esophagectomy has increasingly replaced laparoscopic-hybrid esophagectomy (HMIE) as the standard of care in our institution. Aim of this study was to compare these procedures.

**Materials and methods:**

Over a 24-month period, 199 patients underwent esophagectomy for cancer at our institution. Out of these patients, 51 underwent a robotic-assisted hybrid technique. Each patient was matched according to gender, age, comorbidity and ASA-classification, tumor stage und localization, histology and neoadjuvant treatment with a patient who underwent HMIE. Perioperative parameters and results were extracted from our institutional database and compared among the two groups.

**Results:**

After the matching-procedure, 88 patients could be included in the study. Between laparoscopic and robotic-hybrid esophagectomy no significant differences could be found in operating-time (median 281 vs 300 minutes), R0-resection rate (n = 42 vs 42), harvested lymph-nodes (median 28 vs 24 nodes), length of hospital (median 19 vs 17 days) and ICU stay (median 7 vs 6.5 days). Regarding surgical complications no difference could be observed either (n = 42 vs 44), nevertheless life-threatening complications (Clavien-Dindo 4 or 5) occurred less frequently after robotic-assisted procedure (n = 6 vs 3). No gastric-conduit necrosis was observed for both groups.

**Conclusion:**

Minimal-invasive esophagectomy still remains a challenging operation with high morbidity even in a high-volume institution. According to our experience, robotic-hybrid esophagectomy should be considered as a feasible and safe option. It showed comparable results with the laparoscopic-hybrid procedure even after a short learning period.

## DGAV: Upper gastro-intestinal tract

### Minimal-Invasive Versus Open Esophagectomy for Esophageal Cancer or Cancer of the Gastroesophageal Junction: Comparison of Postoperative Outcomes and Long-Term Survivals Using Propensity Score Matching Analysis

(Abstract ID: 85)

M. Biebl^1^, S. Knitter^1^, T. Hofmann^1^, S. Chopra^1^, C. Denecke^1^, M. Bahra^1^, J. Pratschke^1^, A. Andreou^1^

^1^*Charité - Universitätsmedizin Berlin - CVK*

**Background:**

Radical esophagectomy for patients with cancer is crucial for achieving prolonged survivals. The role of minimal-invasive esophagectomy (MIE) in comparison to conventional open resection for esophageal cancer or cancer of the gastroesophageal junction needs further investigation.

**Materials and methods:**

Clinicopathological data of patients who underwent oncologic thoracoabdominal esophagectomy between 2010 and 2017 were assessed. Postoperative outcomes und long-term survivals of patients following MIE were compared with those of patients undergoing conventional open esophagectomy (OE) after 1:1 propensity score matching.

**Results:**

During the study period, 236 patients underwent esophagectomy for esophageal cancer or cancer of the gastroesophageal junction. After excluding 22 hybrid procedures, 82 patients who underwent MIE were compared with a matched cohort of 82 patients who underwent OE. The rate of patients with esophageal cancer (55% vs. 68%) or cancer of the gastroesophageal junction (45% vs. 32%, P = .077), preoperative chemotherapy (85% vs. 74%, P = .080) or radiotherapy (44% vs. 45%, P = .931), and adenocarcinoma (62% vs. 57%) or squamous cell carcinoma (38% vs. 43%, P = .460) was comparable between the two groups (MIE vs. OE). MIE was associated with a higher median number of harvested lymph nodes (28 vs. 16.5, P < .0001), reduced need for transfusions (7% vs. 41%, P <.0001), lower overall (48% vs. 71%, P = .003) and major postoperative morbidity (24% vs. 46%, P = .003), reduced pulmonary (39% vs. 60%, P = .008) and cardiovascular complications (11% vs. 23%, P = .038) compared to OE. Postoperative 90-day mortality was lower after MIE than after OE (3% vs. 12%, P = .040). After a median follow-up time of 24 months, MIE showed comparable 3-year disease-free survival rates (62% vs. 63%, P = .773) in comparison to OE.

**Conclusion:**

MIE is associated with lower postoperative morbidity and mortality, resulting in similar disease-free survival rates compared to those achieved with the conventional OE. Our data suggest that MIE should be preferably performed in patients with esophageal cancer or cancer of the gastroesophageal junction.

### The impact of comorbidities on outcomes of patients undergoing resection for gastric or esophageal carcinoma

(Abstract ID: 86)

S. Knitter^1^, A. Andreou^1^, S. Chopra^1^, A.-C. Heilmann^1^, J. Spenke^1^, T. Hofmann^1^, C. Denecke^1^, M. Bahra^1^, J. Pratschke^1^, M. Biebl^1^

^1^*Charité - Universitätsmedizin Berlin - CVK*

**Background:**

Selection of appropriate candidates for upper gastrointestinal (GI) cancer surgery and perioperative management have been increasingly improved in the recent years. However, the impact of medical comorbidities on postoperative and long-term outcomes in patients undergoing resection for gastric or esophageal cancer remains unclear.

**Materials and methods:**

Clinicopathological data of patients who underwent resection for gastric or esophageal cancer between 2005 and 2015 were evaluated. A classification of comorbidities defined as the Comorbidities Score (CS) was used to facilitate assessment of the risk for increased postoperative morbidity, mortality and diminished overall survival following resection for upper GI cancer.

**Results:**

Curative resection for gastric and esophageal cancer was performed in 705 patients. Transthoracic esophagectomy, extended gastrectomy, total gastrectomy, subtotal gastrectomy, and the merendino procedure was performed in 45%, 21%, 10%, and 2% of the patients, respectively. CS describes the presence of no (Grade 0), one (Grade A), two (Grade B), three (Grade C), or four (Grade D) concomitant pathologic conditions from different organ systems including cardiovascular, metabolic, pulmonary, renal, and hepatic diseases. Advanced CS was associated with higher complications rates (P =.001) and independently predicted higher major complications rate in multivariate analysis (odds ratio [OR] 1.5, 95% confidence interval [CI] 1.0-2.1, P = .042). However, higher CS did not result in worse postoperative mortality (P = .281). CS was also associated and with a lower rate of patients returning to intended adjuvant systemic treatment (P = .028) and had a negative impact on overall survival (61, 44, 40, and 26 months for CS 0, A, B, and C, respectively, P = .135), even though statistical significance was not reached.

**Conclusion:**

CS is associated with a higher risk for major postoperative complications following resection for gastric or esophageal cancer and may be used as a prediction tool for intensified patient preparation before surgery. Modern perioperative care protocols and successful management of complications are essential to facilitate completion of multimodal treatment concepts enabling optimum outcome for upper GI cancer.

### Epithelial-to-mesenchymal transition markers are associated with increased aggressiveness of gastric cancer leading to extra-capsular growth of lymph node metastases

(Abstract ID: 165)

F. Bösch^1^, S. Heublein^2^, P. Ganschow^1^, A. Bazhin^1^, M. Guba^1^, J. Werner^1^, J. Neumann^3^, M. K. Angele^1^

^1^*Uniklinik München*

^2^*Universitätsklinik Heidelberg*

^3^*Klinikum der LMU München*

**Background:**

Extra-capsular growth (ECG) describes the extension of neoplastic cells beyond the lymph node capsule and is associated with inferior survival rates. Moreover, our previous studies revealed that the growth pattern of lymph node metastases (intra- (ICG) vs. extra-capsular growth) is an independent risk factor in gastric cancer. Epithelial-to-mesenchymal transition (EMT) is regarded to be a major step within metastasis formation and increased expression of EMT markers represents higher tumor invasiveness. Therefore, aim of the present study was to analyze EMT markers within gastric cancer with ECG or ICG.

**Materials and methods:**

The examined patient population comprises 199 patients with lymph node positive gastric cancer who received a primary gastrectomy. On the basis of growth pattern of the lymph node metastases, patients were either in the ICG or ECG group. RNA of the primary tumors was isolated and 96 genes associated with EMT were analyzed, using the RT² Profiler PCR Array (Qiagen, Germany).

**Results:**

Seventy-three patients (36.7%) had ECG and 126 patients (63.3%) had ICG. Patients with ECG had a median survival of 16.21 months whereas patients without ECG had a median survival of 37.07 months (p<0.001). Gene expression analysis revealed that tumors leading to ECG had differentially expressed genes. Three EMT associated genes (EGFR, PLEK2, SNAI1) were significantly upregulated in gastric cancer leading to ECG. Within the ECG group there was at least a two-fold upregulation and a 0.5-fold downregulation of eleven and three genes, respectively.

**Conclusion:**

An extracapsular growth pattern of affected lymph nodes is associated with poor patient survival. The upregulation of typical EMT associated genes suggests that extracapsular growth is an expression of the tumor's increased ability to metastasize. Further studies are needed to analyze the differentially regulated genes and their impact on gastric cancer.

### Laryngopharyngeal pH Monitoring (Restech) may predict a successful surgical outcome for regurgitation and oropharyngeal symptoms after laparoscopic antireflux surgery

(Abstract ID: 211)

D. T. Müller^1^, L. Knepper^1^, W. Schröder^1^, F. Gebauer^1^, C. J. Bruns^1^, J. M. Leers^1^, H. Fuchs^1^

^1^*Uniklinik Köln*

**Background:**

Laryngopharyngeal pH-monitoring (Restech) is a relatively new reflux testing device that needs more validation. It was developed to detect both liquid and acidic gas vapor, and the more consistent pharyngeal placement may lead to more reliable results, especially when laryngopharyngeal symptoms such as cough, hoarseness and globus sensation are present. Aim of this study is to determine if Restech can identify patients with a successful outcome for certain symptoms after antireflux surgery.

**Materials and methods:**

In our esophageal center of excellence, more than 300 esophageal surgeries are performed annually. All patients undergoing minimally invasive or open upper gastrointestinal surgery are prospectively entered in our IRB approved database and undergo a routine check-up program with postoperative surveillance following surgery. All patients with benign disease received a complete diagnostic work-up for gastroesophageal reflux including symptom evaluation, endoscopy, 24-hour impedance pH-metry, high resolution manometry and Restech. Only patients with a complete dataset and oropharyngeal reflux symptoms were offered inclusion into this study and evaluated using 24-h laryngopharyngeal and simultaneous esophageal impedance pH-monitoring.

**Results:**

A total of 155 [99 females] consecutive patients with suspected gastroesophageal reflux disease and oropharyngeal symptoms that were seen between 10/2013 and 08/2018 were included and underwent 24-h laryngopharyngeal with concomitant esophageal pH-monitoring.A total of 24 of these patients with laryngopharyngeal symptoms underwent laparoscopic antireflux surgery from 10/2013 - 02/2018 and had a complete follow up. Restech evaluation was abnormal in 62.5% (n=15, mean RYAN Score upright 121.73 [35.1-386.05], mean RYAN Score supine 5.74 [2.17-50.62]). Two out of preoperatively 12 [16.7%] patients with a pathologic Restech test complained about regurgitation after surgery versus 4 out of preoperatively 8 [50%] patients with a normal Restech result. No patient with an abnormal Restech result out of preoperatively 11 complained about extraesophageal symptoms after the surgery versus 1 from preoperatively 4 [25%] patients with a negative Restech.

**Conclusion:**

An abnormal Restech result better identifies a successful outcome for regurgitation and extraesophageal symptoms after antireflux surgery. All patients had a complete resolution of extraesophageal symptoms after surgery.

**Picture:**
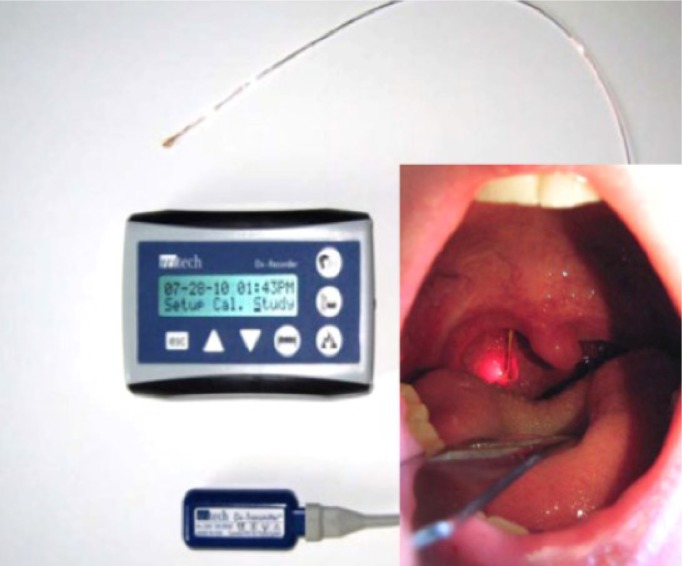


### The value of a negative preop Restech test for postop results – Gastrointestinal function testing using the new minimally invasive laryngopharyngeal PH probe

(Abstract ID: 213)

D. T. Müller^1^, L. Knepper^1^, W. Schröder^1^, F. Gebauer^1^, C. J. Bruns^1^, J. M. Leers^1^, H. Fuchs^1^

^1^*Uniklinik Köln*

**Background:**

Laryngopharyngeal pH-monitoring (Restech) is a relatively new reflux testing device that needs more validation. It was developed to detect both liquid and acidic gas vapor, and the pharyngeal probe placement may lead to more reliable results in patients with laryngopharyngeal symptoms.A negative Restech result could be used as a screening instrument for gastroesophageal reflux and help decide whether patients should be included into a diagnostic pathway or benefit from a PPI therapy. Aim of this study is to examine the value of negative Restech test results using a large patient collective.

**Materials and methods:**

In our esophageal center of excellence, more than 300 esophageal surgeries are performed annually. All patients undergoing minimally invasive or open upper gastrointestinal surgery are prospectively entered in our IRB approved database and undergo a routine check-up program with postoperative surveillance following surgery. All patients with benign disease received a complete diagnostic work-up for gastroesophageal reflux including symptom evaluation, endoscopy, 24-hour impedance pH- metry, high resolution manometry and Restech. Only patients with a complete dataset and oropharyngeal reflux symptoms were offered inclusion into this study and evaluated using 24-h laryngopharyngeal and simultaneous esophageal impedance pH-monitoring.

**Results:**

A total of 155 [99 females] consecutive patients with suspected gastroesophageal reflux disease and oropharyngeal symptoms that were seen between 10/2013 and 08/2018 were included and underwent 24-h laryngopharyngeal with concomitant esophageal pH-monitoring.Restech evaluation was negative for reflux (=normal) in 55.5% (n=86, mean RYAN Score upright 2.5 [2.12-8.57], mean RYAN Score supine 2.2 [2.17-5.86]). In 45.3% of Patients with a normal Restech evaluation, 24-hour pH-metry was pathologic (n=39, mean DeMeester Score 55.98 [14.8-254.9]). Nearly half of the patients with a normal RYAN and a normal DeMeester Score (n=20) did not complain about heart burn but only oropharyngeal symptoms. No patient with a normal RYAN Score and a normal DeMeester Score underwent antireflux surgery.

**Conclusion:**

As shown in earlier research, Restech and 24-hour pH do not necessarily need to correspond. More women than men presented with oropharyngeal reflux related symptoms.A negative Restech examination combined with a negative 24-hour pH metry may help to support the decision for or against antireflux surgery but is alone not suitable as a negative screening tool for GERD.

**Picture:**
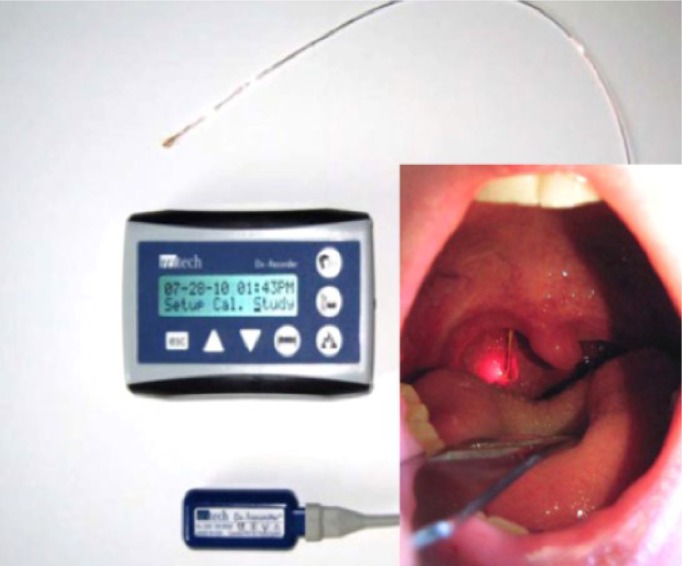


### Prolonged preoperative intravenous chemotherapy reduces overall survival in patients with peritoneal metastasis of gastric cancer, suitable for cytoreductive surgery and hyperthermic intraperitoneal chemotherapy

(Abstract ID: 325)

A. Brandl^1^, M. Biebl^1^, P. Thuss-Patience^1^, A. Arnold^1^, W. Raue^2^, J. Pratschke^1^, B. Rau^1^

^1^*Charité - Universitätsmedizin Berlin CVK, Berlin*

^2^*Allgemeines Krankenhaus Celle, Celle*

**Background:**

Patients with peritoneal metastases of gastric cancer have a poor prognosis and median survival of 7 months. This study compared treatment options and outcomes based on the Peritoneal Cancer Index (PCI).

**Materials and methods:**

This retrospective analysis included patients with gastric cancer treated between August 2008 and December 2017 with synchronous peritoneal metastases only diagnosed by laparoscopy. The three treatments were as follows: 1) cytoreductive surgery (CRS) and hyperthermic intraperitoneal chemotherapy (HIPEC) in combination with pre- and postoperative systemic chemotherapy (n=58), 2) laparotomy/laparoscopy without CRS, but HIPEC in combination with pre- and postoperative systemic chemotherapy (n=11) and 3) systemic chemotherapy only (n=19).

**Results:**

A total of 88 patients aged 54.6±10.9 years with mean PCI of 14.3±11.3 were included. The PCI was significantly lower in group 1 (8.3±5.7) than in group 2 (23.9±11.1, p<0.001) and group 3 (27.3±9.3; p<0.001). Mean time from diagnosis to laparoscopy was 5.2±2.9 months. The median overall survival was 9.8±0.7 for group 1, 6.3±3.0 for group 2 and 4.9±1.9 months for group 3 (p<0.001). Predictors for deteriorated overall patient survival included >4 cycles of preoperative chemotherapy (HR 4.49; p<0.001), lymph node metastasis (HR 3.53; p=0.005), PCI >=12 (HR 2.11; p=0.036), and incompleteness of cytoreduction (HR 4.30; p=0.001) in patients treated with CRS&HIPEC.

**Conclusion:**

CRS&HIPEC showed convincing results in selected patients with PCI <12 and complete cytoreduction. Prolonged duration (>4 cycles) of preoperative intravenous chemotherapy reduced patient survival in patients suitable for CRS&HIPEC.

### Preemptive endoluminal vacuum drainage to reduce anastomotic leakage after esophagectomy

(Abstract ID: 336)

H. M. Schmidt^1^, C. Gubler^1^, D. Vetter^1^, P. Müller^1^, B. Morell^1^, D. Raptis^2^, C. Gutschow^1^

^1^*Universitätsspital Zürich*

^2^*Royal Free Hospital London*

**Background:**

Anastomotic leakage (AL) remains a major cause of morbidity in foregut surgery. In many centers, endoluminal vacuum therapy (EVT) after esophagectomy has become the mainstay of therapy for AL after esophagectomy. A novel concept is to use this technology in a preemptive setting with the aim of reducing AL formation and postoperative morbidity.

**Materials and methods:**

Preemptive EVT (pEVT) was performed in consecutive patients undergoing minimally invasive esophagectomy with cervical (n=1) or high intrathoracic (n=18) anastomosis between November 2017 and July 2018. PEVT was performed during surgery immediately after completion of esophago-gastrostomy. Routine sponge-removal was performed three to six (median 5) days after esophagectomy. Records of patients were reviewed with respect to demographic characteristics, oncological parameters, surgical procedures, and the postoperative course up to 30 days after surgery. Endpoints of this study were adverse events related to pEVT, postoperative morbidity and AL rate, defined according to the definitions of the Esophageal Complications Consensus Group (ECCG). The Clavien-Dindo (CD) classification, and the Comprehensive Complication Index (CCI) were used to categorize and grade complications and morbidity.

**Results:**

There were 20 anastomoses at risk in 19 patients. Mortality after 30 days was 0% and anastomotic healing was uneventful in 19 of 20 anastomoses at risk in this series (95%). One high-risk patient after minimally invasive Ivor Lewis esophagectomy had a minor contained AL that healed uneventfully after a second course of pEVT for 5 days. One patient (5.3%) experienced major morbidity (Clavien-Dindo >= CD grade IIIb) unrelated to anastomotic healing. He required surgical revision with shortening of the gastric tube and open re-anastomosis (with pEVT) after 12 days because of redundancy of the interponate with failure of transition to oral diet. Except early proximal dislodgement in one patient (5.3%), there were no adverse events attributable to pEVT. The median postoperative ICU and hospital stay was 1 (IQR 1-2) and 14 (IQR 11-16) days, respectively. The median CCI at 30 days after surgery was 20.9 (IQR 0-26.2).

**Conclusion:**

PEVT appears to be a safe procedure that may have the potential to improve surgical outcome, particularly in high-risk patients undergoing esophagectomy. Further research is required to elucidate the true potential of this technique in the preemptive setting.

### Comparison of Pressurized Intra Peritoneal Aerosol Chemotherapy (PIPAC) and intravenous chemotherapy with intravenous chemotherapy only in patients with synchronous peritoneal metastases of gastric cancer

(Abstract ID: 341)

A. Brandl^1^, M. Alberto^1^, M. Schomaker^1^, M. Biebl^1^, J. Pratschke^1^, P. Thuss-Patience^1^, B. Rau^1^

^1^*Charité - Universitätsmedizin Berlin CVK*

**Background:**

Patients with peritoneal metastases of gastric cancer have a poor prognosis and a median survival of 7 months. Recently, patients with extensive metastatic disease treated with intensive intravenous chemotherapeutic regime were not able to exceed a median survival of 11 months. During the last years, the evidence supporting regional application of chemotherapy, especially Pressurized Intra Peritoneal Aerosol Chemotherapy (PIPAC) is growing.

**Materials and methods:**

This retrospective analysis included patients with synchronous peritoneal metastases of gastric cancer (n=15) treated with PIPAC (Cisplatin: 7,5 mg/m2; Doxorubicin 1,5mg/m2) and alternating two cycles of intravenous chemotherapy (FLOT or second line chemotherapy) between December 2016 and March 2018. The study cohort was compared with a historic cohort of patients (n=16) with synchronous peritoneal metastases of gastric cancer treated with systemic chemotherapy between 2010 and 2016 and diagnosed by laparoscopy at our institute.

**Results:**

We performed in total 26 (min. 1 - max. 4) PIPAC procedures in 15 patients. The demographic analysis showed significant difference in patient age (49.0 ± 8.0 vs. 59.7 ± 10.4; p=0.006), while other factors like BMI, Peritoneal Cancer Index, Her2 positivity, gender, and ASA classification showed no significant difference. The median overall survival of patients treated with PIPAC and systemic chemotherapy was 17.0 months compared to 7.0 months in patients treated with systemic chemotherapy only (p<0.001). The mean operative time of PIPAC procedures was 75 minutes. Perioperative morbidity was low (7.7%) with no reoperation and no intensive care unit admission. Mortality was 0%. Five patients could not complete the suggested three cycles due to tumour progression (n=3 peritoneal; n=1 liver metastases) or massive adhesions (n=1).

**Conclusion:**

PIPAC with 7,5 mg/m2Cisplatin and 1,5 mg/m2Doxorubicin with alternating two cycles of intravenous chemotherapy is a safe therapeutic regimen with low morbidity and mortality in patients with peritoneal metastases of gastric cancer. This therapy showed significant higher median survival in this retrospective study. Further randomized control trials are necessary to evaluate the effect of PIPAC in these patients.

### Comparison of Percutaneous Transhepatic Cholangiodrainage versus surgical treatment of patients with duodenal stump insufficiency after gastric resection

(Abstract ID: 414)

A. Gabersek^1^, M. Weitzendorfer^1^, F. Singhartinger^1^, A. Majerus^1^, P. Mandal^1^, R. Bittner^1^, E. Emmanuel^1^, O. Koch^1^

^1^*Universitätsklinikum Salzburg*

**Background:**

Duodenal stump insufficiency is a life-threatening complication in patients who have undergone gastric resection. Treatment of duodenal stump insufficiency is complicated and it is not clear which therapeutic method leads to faster clinical recovery and shortens the length of hospital stay. Aim of the study was to compare the outcomes of duodenal stump insufficiency after treatment with a percutaneous transhepatic cholangiodrainage (PTCD) with surgical intervention.

**Materials and methods:**

Retrospective analysis of all patients who have developed duodenal stump leakage between January 2007 and June 2018 at the University Hospital of Paracelsus Medical University Salzburg, after undergoing total gastrectomy, Billroth II resection or subtotal gastrectomy. Patients were divided into two groups according to the type of treatment they have received - a conservative group treated with a PTCD and a surgical group treated with a relaparotomy and surgical closure of the duodenal stump. The length of hospital stay and the number of days spent at the intensive care unit after relaparotomy or after becoming a PTCD were analyzed in both groups. Further analysis involved the tracking of CRP value, total bilirubin, aspartat-aminotransferase (AST), alanin-aminotransferase (ALT), gamma-glutamyl-transferase (GGT), alkaline phosphatase, amylase, lipase and leukocytes on the day of the intervention (surgery/becoming a PTCD) as well as on the first day, one week, two weeks, and one month after the intervention.

**Results:**

In the study period 17 patients (9 male and 8 female patients, mean age 67,3 years) developed duodenal stump leakage after surgery. Seven patients were treated with PTCD and ten patients underwent relaparotomy. Mean length of the hospital stay in the group treated with PTCD was 51,7 days and in the relaparotomy group 53,6 days (p=0,904). Mean number of days spent at the intensive care unit in the PTCD group compared to surgical group was 11 versus 23,0 days (p=0,235). Comparison of the tracked blood values showed faster normalization of alkaline phosphatase within the first two weeks with mean value of 138 U/L in the surgery group compared to the mean value of 264 U/L in PTCD group (p=0,040). Also significantly faster normalization of Lipase within the first week was noted in patients after surgery (mean 21,7 versus 82,0 U/L; p=0,004). Comparison of remaining blood values showed no statistically significant differences between the two groups.

**Conclusion:**

PTCD therapy of duodenal stump insufficiency after gastric resection seems to be the treatment of choice, since it leads, to faster clinical recovery, shorter hospital and intensive care unit stay. Further studies are required to confirm the benefit of conservative therapy in comparison to surgical reintervention for treatment of duodenal stump insufficiency.

### The role of hormones in symptoms and pathophysiology of reflux-associated diseases of the esophagus

(Abstract ID: 482)

F. X. Singhartinger^1^, L. Wahl^1^, O. O. Koch^1^, K. Emmanuel^1^, M. Weitzendorfer^1^

^1^*Universitätsklinikum Salzburg*

**Background:**

GERD is an important topic since 10-20 percent of the population suffer from its symptomes in Europe and the US. However, its pathophysiology is not totally understood. Few studies suggest that hormones play a role, especially in the motility of the lower esophagus, but data is rare. Furthermore, the reasons for other motility disorders like achalasia is partly unknown. In this prospective study we evaluated the correlation between hormones (TSH, fT3, fT4, calcitonin, gastrin and VIP) and GERD as well as its gastrointestinal symptoms.

**Materials and methods:**

100 consecutive patients with typical reflux symptoms, atypical reflux symptoms and dysphagia where hospitalized for diagnostic evaluation. Questionnaires to evaluate quality of life (GIQLI) and reflux symptoms (reflux symptom index and symptom check list) were handed out. Patients routinely underwent serum analysis (TSH, fT3, fT4, calcitonin, gastrin and VIP), 24-hours-pH-impendance monitoring and high resolution manometry. After high resolution manometry patients where sub-divided into three groups with minor motility disorders, major motility disorders and normal values according to the Chicago classification.

**Results:**

Complete data was available from patients (male:female 44:42, median age 56). Motility disorders were found in 38 out of 86 patients. A pathological DeMeester was found in 45 patients with median score of 35. There have been no correlations between the DeMeester score and the hormones. Of the 86 patients, the median LES pressure was 23,6 mmHg, but no correlations were found between the different hormone levels and the LES-pressure. But a strong inverse relation between calcitonin and the Integrated Relaxation Pressure (IRP) of the esophagogastric junction (EGJ) was found (r= -0,492; p=0,000). No correlations were found between hormone levels and the mean scores of GIQLI, RSI and SCL. But positive correlations were found between VIP and GI-Symptoms (r=0,298; p= 0,011), as well as correlations between fT3 and dysphagia (r=0,283, p=0,016). Within the group II, TSH and fT4 correlated with outcomes of the SCL-questionnaire. fT4 correlated with the Typical-symptoms (p=0,002), as well as the GI-symptoms (diarrhea, constipation, flatulence) (p=0,039). TSH correlated with the Typical-symptoms (p=0,007). A regression analysis confirmed that these outcomes were no coincidence. Further within group II a correlation between VIP and the SCL-Gas-Bloat-Symptom (p=0,072) was found.

**Conclusion:**

Few studies and case reports suggest a connection between GERD and hormones. We found no correlation between TSH, fT3, fT4, gastrin, VIP and calcitonin and GERD, though, calcitonin seems to have an effect of the lower esophageal sphincter. Further studies need to be done to evaluate the role of hormones in motility disorders of the upper GI.

### A safe and feasible method for esophagojejunostomy intracorporeal hand-sewn reconstruction in totally laparoscopic total gastrectomy

(Abstract ID: 500)

H. Huang^1^

^1^*Fudan University Shanghai Cancer Center, Shanghai*

**Background:**

In laparoscopic total gastrectomy, esophagojejunostomy using a circular stapler has become the preferred method. However, placing a purse-string suture in the distal esophagus and inserting an anvil is a technically demanding procedure. Conventional laparoscopic assisted total gastrectomy (LATG) need an auxiliary incisionfor esophagojejunostomy sometimes this incision is not too small. Totally laparoscopic total gastrectomy (TLTG) is a very difficult operation for the difficulty associated with esophagojejunostomy during this procedure without auxiliary incision. Although several techniques have been reported to overcome this issue, a reliable technique hasnot yet been established. We successfully performed intracorporeal esophagojejunostomy using a complete hand-sewn reconstruction, and have shown its favorable outcomes compared with those of conventional laparoscopic assisted total gastrectomy. Here we describe our technique in the video.

**Materials and methods:**

After transection of the esophagus, a complete hand-sewn reconstruction was performed, first all seromuscular layer suture jejunum to esophagus at the left side and right side with 3-0 antibacterial suture, then suture bowl and esophagus full-thickness with a 3-0 bidirectional barbed wound-closure device completely with hand-sewn. When sew the posterior wall from the left to the right side of the cut end in an inside-to-inside direction, and then sew the anterior wall from the right side to the left side in an outside-to-outside direction. Finally, intracorporeal esophagojejunal anastomosis was performed using hand-sewn.

**Results:**

In totally laparoscopic total gastrectomy, reconstruction using this method was performed for 13 patients with gastric cancer. There were no serious intraoperative complications, one case need conversion to open surgery. There were no anastomotic leakage and stenosis occurred. The meantime for esophagojejunal anastomosis was 43 min for the 12 patients who received complete hand-sewn successfully.

**Conclusion:**

The advantage of this technique is economical from omitting of stapler. And it is more minimally invasive without auxiliary incision. Complete hand-sewn esophagojejunal reconstruction in totally laparoscopic total gastrectomy is safe and feasible with minimal invasiveness.

### Prognostic implications by the eighth edition of the UICC-classification for gastric cancer patients – A comparative analysis between a German and an Eastern Asian specialized treatment center

(Abstract ID: 501)

N. Samm^1^

^1^*Klinikum Rechts der Isar der TU München*

**Background:**

The validity of the eighth edition of the UICC staging system for gastric cancer has been evaluated only in Asian cohorts, and not reported in European cohorts so far. The aim of this study was to evaluate the prognostic performance of the eighth edition UICC staging system in both German and Korean cohorts.

**Materials and methods:**

A total of 6,121 (526 from Germany, 5595 from Korea) patients treated for gastric cancer were reclassified according to the eighth edition. Survival according to the UICC stages was estimated by the Kaplan Meier method and compared with log-rank tests. A Cox proportional hazards model was fitted adjusting for demographic and clinicopathological factors, and ROC analysis was conducted.

**Results:**

The German cohort had different characteristics in age, tumor size, location, Lauren classification, stage, and type of surgery compared to the Korean cohort. The eighth edition staging system did not provide significant survival differences between each adjacent stage in the German cohort, but did in the Korean cohort. Multivariate analyses revealed that the eighth edition staging system was an independent prognostic factor and C-statistics were greater than 0.78 in both German and Korean patients. The results were comparable to the UICC seventh edition. (C-statistics: 0.768 vs. 0.767 in the German, and 0.789 vs. 0.785 in the Korean cohort for seventh vs. eighth edition).

**Conclusion:**

The eighth edition UICC staging-system showed prognostic value in predicting survival of gastric cancer patients in both German and Korean cohorts. However, the predictive ability of the eighth edition was similar to that of the seventh edition in both cohorts.

### Two-stage esophagectomy as inividualized therapy for esophageal cancer – indication and outcome

(Abstract ID: 561)

I. Bartella^1^, F. Fuchs^1^, M. Bagheri^1^, C. J. Bruns^1^, W. Schröder^1^

^1^*Uniklinik Köln*

**Background:**

Anastomotic leakage is the most important surgical complication following esophagectomy for esophageal cancer, leading to increased morbidity and mortality. A major cause of leakage is impaired healing due to ischemia of the gastric tube that is used for reconstruction of the gastrointestinal tract. A possible therapy option for such patients, but also for high-risk patients due to other comorbidities, is the two-stage esophagectomy with ischemic conditioning. The benefit of this individualized therapy is currently under discussion. The aim of this retrospective analysis was to summarize possible indications for a two-stage esophagectomy and to present its outcome.

**Materials and methods:**

Clinical data of all patients who underwent a two-staged esophagectomy for esophageal cancer between 05/2016 and 04/2018 were reviewed. Two-stage esophagectomy consists of a laparoscopic gastric mobilization with partial devascularisation and an open transthoracic esophagectomy with intrathoracic reconstruction (hybrid Ivor-Lewis esophagectomy) after an interval of 4-5 days. Comorbidities, indication for the procedure as well as short-term outcome were recorded and anylysed by using SPSS (version 25). Complications were defined according to ECCG criteria and classified according to Clavien-Dindo (CD).

**Results:**

34 of 347 patients (9.8%) underwent a two-staged transthoracic Ivor-Lewis esophagectomy. 85.3% of the patients had a history of nicotine and 32.4% of alcohol addiction. 13 patient (38.2 %) had a stenosis of the celiac trunc (TC) and the superior mesenteric artery (SMA). The most recorded comorbidities were cardiac (73.5%), pulmonary (29.4%), renal (14.7%) and liver (5.9%) diseases. The decision for a two-stage esophagectomy was made in 61.8% preoperatively because of a TC respectively AMS stenosis (52.4%) or cardiopulmonary comorbidities (47.6%). Intraoperative decision for a two-stage procedure was made due to a previously unknown liver fibrosis (38.5%), intraoperative cardiopulmonary complications (30.8%), a macroscopically impaired gastric perfusion after devascularisation (23.1%) and a suspected metastasis (7.6%). 12 patients (35,3 %) had an uneventful postoperative course (CD 0). 17 patients (50,0%) had ,minor’ (CD <= IIIA) and 4 patients (11,8%), major complications’ (CD IIIB- IVB). One patient died due to massive aortic bleeding (CD V). Three patients (8.8%) developed an anastomotic leakage (Type II according to ECCG criteria) and one patient a conduit necrosis. The median hospital stay was 15 days.

**Conclusion:**

Two-stage esophagectomy is a feasible and safe surgical procedure for patients with increased risk of postoperative complications due to a high number of comorbidities. Postoperative complication rates are comparable to recently published large registry data (,ESOData benchmarking’).

### Neuromodulation of the esophagus to address Gastroesophageal Reflux Disease in postbariatric patients

(Abstract ID: 603)

Y. Borbély^1^, G. Plitzko^1^, D. Kröll^1^, P. C. Nett^1^, D. Candinas^1^

^1^*Universitätsspital Bern*

**Background:**

Gastroesophageal Reflux Disease (GERD) has a high prevalence in bariatric patients. Whilst 85% of patients with preoperative GERD after Roux-Y Gastric Bypass (RYGB) are symptom-free, sleeve gastrectomy (SG) as the most commonly performed bariatric procedure results in considerable de novo GERD and may worsen preexisting GERD.

Given the absence of anatomical abnormalities, options in patients with persistent GERD after RYGB are limited. Further, post-SG patients do not have good treatment options except for more invasive, anatomy-altering conversion to RYGB.

Neuromodulation of the esophagus has shown to improve outcomes in GERD patients. Consisting of an implantation of two electrodes beneath the lower esophageal sphincter, it respects the anatomy, improves lower esophageal sphincter pressures and esophageal motility.

This study evaluates the efficacy of esophageal neuromodulation in postbariatric patients with GERD not controlled under maximum dose PPI therapy.

**Materials and methods:**

Data of all consecutive patients undergoing implantation of an EndoStim-system (ES) in a postbariatric setting a university hospital were recorded in a prospective computer database and reviewed retrospectively. Preoperative GERD evaluation consisted of questionnaires, gastroscopy, upper GI series and functional esophageal testing with 24h-pH-impedance-manometry. Postoperative evaluation included questionnaires, gastroscopy after 1 year and 24h-pH-impedance-manometry after 9 months.

**Results:**

15 patients post-SG and 4 patients post-RYGB underwent implantation of ES. Mean follow-up was 1.8 y.

Post-SG: mean Body Mass Index (BMI) was 37.2±9.4 kg/m2, mean age 44.4±12.5 y and mean interval to initial SG 2.7±1.3 y. GERD-HRQL scores (50 max) improved from 38.3 to 6.4, mean esophageal acid exposure (% time of pH <4) improved from 13.8% to 4.5%.

Post-RYGB: mean Body Mass Index (BMI) was 34.6±12.4 kg/m2, mean age 63.6±13.7 y and mean interval to initial RYGB 4.2±2.8 y. GERD-HRQL scores (50 max) improved from 39.5 to 5.2, mean esophageal acid exposure from 23.8% to 3.6% (in 3 patients, one had non-acidic reflux).

**Conclusion:**

Neuromodulation of the esophagus results in a significant improvement of GERD-symptoms and esophageal pH-exposure, both in patients after SG and RYGB. It provides a valid option to address GERD without the need to alter the existing anatomy.

### Postoperative recovery after surgery for cancers of the esophagus and stomach: Does preoperative patients’ condition predict outcome?

(Abstract ID: 614)

M. Thomaschewski^1^, H. Meyer^1^, O. Kopeleva^1^, T. Keck^1^, R. Hummel^1^

^1^*Uniklinik Lübeck*

**Background:**

Esophageal and gastric cancers usually occur at a higher age, and patients often present significant comorbidities. In combination with the risk profile of esophageal and gastric cancer surgery, the preclinical condition is relevant for morbidity and mortality after esophagectomy and gastrectomy. We aimed with the current project to investigate if postoperative physical recovery is as well significantly affected by preclinical conditions of the patient, and whether the patients’ condition might be a surrogate for the need for extensive rehabilitation.

**Materials and methods:**

We conducted a retrospective study on 108 consecutive patients that underwent esophagectomy (n=56) or gastrectomy (n=52) between 2013 and 2017. We analysed the impact of patients’ condition, tumor stage and treatment and complications on ICU time, respirator time, physical recovery in hospital and discharge conditions.

**Results:**

Median age of the predominant male population (80% / 65%) was 65 respectively 72 years for esophagectomy and gastrectomy. 84 respectively 100% of cases represented adenocarcinomas. Preoperative risk was assessed as normal / increased / high risk using the Cologne Risk Score in 2% / 36% / 63% respectively 6% / 41% / 53% of cases, and ASA score was 2 / 3 / 4 in 20% / 73% / 7% respectively 19% / 71% / 10%. Preoperative patients` conditions (determined by ASA and Cologne Risk Score), BMI, age, neoadjuvant therapy, tumor stage and tumor entity (squamous cell carcinoma versus adenocarcinoma) had no impact on postoperative ICU stay, respirator time, physical recovery and discharge condition. Postoperative complications determined by Clavien Dindo Score negatively affect both discharge condition and physical recovery, whereas age negatively affects discharge conditions (p<0.005).

**Conclusion:**

Preoperative patients’ condition seems to have no significant impact on recovery after esophagectomy of gastrectomy. However, complications affect recovery negatively.

### Robot-assisted gastric GIST resection: A single center experience

(Abstract ID: 631)

C. Wassmer^1^

^1^*Universitätsspital Genf*

**Background:**

Gastrointestinal stromal tumors (GIST) are the most common mesenchymal tumors, representing 1- 3% of all gastrointestinal cancer. Surgical resection is the only curative treatment. Minimally invasive approaches such as laparoscopic and robotic-assisted resections for gastric GIST have proved to be oncologically and surgically safe. We report here a case series of robot-assisted gastric GIST resections.

**Materials and methods:**

We performed a retrospective analysis of all gastric GIST resected between 2007 and 2018 in our center.

**Results:**

19 patients underwent robot-assisted gastric resection for GIST, 12 females and 7 males. Median age was 59 years (range 38-79) and median BMI was 27.5kg/m2 (range, 18.6-41.3). Median tumor size was of 5 cm (range, 1.8-9). 13 were on the posterior wall and 7 were proximal (fundus or cardia). All tumors were completely resected (R0). We noted one conversion to open resection because of a positive margin requiring a radical resection. Median operative time was 163 minutes (range, 90-436). We reported no postoperative complications within 90 days after surgery. The median follow-up was 8 months (range, 1-115) and we reported no oncological recurrence.

**Conclusion:**

Our case series confirm that robotic-assisted resection is safe and offers the same oncological results as the others approaches (open and laparoscopically) for gastric GIST.

### The Correrelation between different sizes of circular Stapler used/utilize in Esophagojejunostomy during total Gastrectomy and the rate of Anastomotic Leakage

(Abstract ID: 640)

M. Elshafei^1^, J. Fleischmann^1^, R. Grützmann^1^, G. F. Weber^1^

^1^*Universitätsklinikum Erlangen*

**Background:**

Existing research recognizes the critical role played byAnatomic leakage (AL) of Esophagojejunostomy after total gastrectomy, to be one of the most severe complications, as it has accentuated the problem of significantly prolonging hospital stays, and thereby, increasing the mortality rate. Recently, the concern for evaluating post-operative complications have gained substantial importance with the increased survival rate of patients after gastrectomy. Therefore, this study aims to assess the correlation between the different sizes of the circular stapler and the rate of Anastomotic leak.

**Materials and methods:**

Through our retrospective analysis, we have conducted an investigation on 391 Patients with Gastric Cancer who underwent total gastrectomy, which focuses on the influence of the circular stapler size 21/25 mm (Group 1) compared to 28/29 mm (Group 2) in terms of the postoperative rate of Anastomotic leakage

**Results:**

Clinical anastomotic leakage was compared in (Group 1= 21/25mm) of 169 Patients to (Group 2=28/29mm) of 222 Patients. The leakage incidence was 8.9 and 4.1 % respectively. Therefore, the stapler size 28/29mm had statistically significant impact on reducing the rate of AL.

**Conclusion:**

The application of the 28/29 mm circular stapler size for the Esophagojejunostomy in the total gastrectomy operations shows significant lower rates of AL, and thereby, the association between the size of the stapler and the rate of AL exists.

### Surgical management of benign esophageal perforations in 77 patients: A single centre analysis over a 15-year period

(Abstract ID: 790)

K.-F. Karstens^1^, E. Bellon^1^, M. Tachezy^1^, J. R. Izbicki^1^, T. Ghadban^1^, F. G. Uzunoglu^1^, M. Bockhorn^1^, M. Reeh^1^

^1^*Universitätsklinikum Hamburg-Eppendorf*

**Background:**

Esophageal perforations are associated with high morbidity and mortality. Different non-operative and operative treatment options have been proposed according to the etiology, severity and systemic response. This study focuses on the impact of different surgical treatments in benign esophageal perforations in a single tertiary centre over a 15-year period.

**Materials and methods:**

From 2002 to 2017 patients with surgically managed esophageal perforation were identified from our database. Patients with esophageal malignancies were excluded. Etiology, clinical data, treatment and outcome were analyzed.

**Results:**

A total of 77 patients were identified. The majority of perforations were iatrogenic (52%) followed by Boerhaave’s syndrome (20.8%). Most ruptures were found in the distal third of the esophagus (61%) measuring less than 3cm (59.7%). Patients were either treated with exploration and drainage (7.8%), primary suture and patch reinforcement (36.4%), resection and restoration of continuity (24.7%) or resection with esophagostoma and feeding jejunostomy (31.2%). In-hospital mortality was 16.9%. Initiation of therapy after 24h significantly correlated with sepsis (p<0.0001). A correlation between both an increasing perforation length with sepsis (p=0.012) and in-hospital mortality with sepsis (p=0.048) was observed. In septic patients an increased number of discontinuity resections were performed (p<0.0001).

**Conclusion:**

Esophageal perforations are associated with high mortality and larger ruptures are associated with worse outcome. Rapid diagnosis and treatment are crucial for patient survival. The advantage of surgical management lies in the rapid control of the septic focus in an already critical ill patient. Though, the kind of surgical technique needs to be adjusted to the individual situation.

### Peroral endoscopic Myotomy (POEM) versus Laparoscopic Heller Myotomy (LHM) for Treatment of Primary Idiopathic Achalasia: Results from the prospectively randomized multicenter trial

(Abstract ID: 801)

B. von Rahden^1^, Y. Werner^2^, B. Hakanson^2^, J. Martinek^3^, A. Bredenoord^4^, R. Bisschops^5^, H. Messmann^6^, J. Kersten^2^, C.-T. Germer^7^, A. Pazdro^2^, M. Schijven^2^, P. Fockens^2^, G. E. Boeckxstaens^2^, A. Repici^2^, T. Rösch^2^

^1^*Universitätsklinikum Salzburg*

^2^*Universitätsklinikum Hamburg-Eppendorf*

^3^*Institute for Clinical and Experimental Medicine (IKEM) , Praha 4*

^4^*Academic Medical Centre, Amsterdam*

^5^*Katholieke Universiteit Leuven*

^6^*Universitätsklinikum Augsburg*

^7^*Universitätsklinikum Würzburg*

**Background:**

Endoscopic balloon dilatation (EBD) and laparoscopic Heller myotomy (LHM) have been used for in the symptomatic therapy of idiopathic achalasia; comparative results have been conflicting with one large randomized study showing equivalence with EBD being repeated. In an attempt to further minimize surgical trauma, peroral endoscopic myotomy (POEM) has been introduced with promising results in early studies and superior results to EBD in a recent randomized trial.

**Materials and methods:**

We randomly assigned patients with primary achalasia to either POEM or LHM with Dor hemifundoplicatio. Primary outcome was the rate of cases with clinically successful therapy at 2 years follow-up as determined by an Eckardt Score of <= 3 (this score ranging from 0-12 indicates combined severity of symptoms such as dysphagia, regurgitation, chest pain and weight loss). Secondary outcomes were manometric pressure of the lower esophageal sphincter, and complications including the development of reflux.

**Results:**

The study population included 221 patients from 8 centers who were randomly assigned to POEM (n=112) or LHM (n=109). Demographic data at baseline in both study groups were similar for age, gender and prior therapy (botulinum toxin injection, balloon dilatation). Most common treatment related severe AE were aspiration during anaesthesia, pneumothorax, mucosal perforation requiring additional therapy and were similar in both groups (4.1% overall). Clinical follow-up is still pending in 14 patients. Clinical success at 2 years (without further therapy) was achieved in 81.9% with POEM and in 80.4% with LHM. Similarly, there was no difference in manometric assessment of the lower esophageal sphincter between POEM (mean IRP 11.3 mg) and LHM (mean IRP 11.5 mg) at 2 years. The rate of endoscopic reflux esophagitis was higher with POEM after 3 months (50.5% LA A/B and 6.3% LA C/D for POEM vs 16.5% LA A/B and 3.3% LA C/D for LHM), which was not significant anymore after two years (40.0% LA A/B and 4.7% LA C/D for POEM vs 23.7% LA A/B and 5.2% for LHM; p=0.05). Rates of acid exposure times >4.5% at 2 years on pHmetry were not significantly different either, but more patients took low-dose proton pump inhibitors daily (39.2% after POEM versus 18.8% after LHM, p< 0.05). Patient-reported quality of life after 2 years was similar between the groups (POEM mean GIQLI score: 117.4 versus LHM mean: 114.7).

**Conclusion:**

POEM has a similarly high efficacy and good safety profile compared to LHM with respect for the treatment of patients with symptomatic achalasia; it can be regarded as an excellent alternative. Higher rates of mild reflux with POEM were observed especially short-term.

### Incidence and outcome of esophageal and gastric cancer after liver transplantation

(Abstract ID: 915)

S. Chopra^1^, C. Gustorff^1^, C. Denecke^1^, E. M. Teegen^1^, A. Andreou^1^, J. Pratschke^1^, M. Biebl^1^

^1^*Charité - Universitätsmedizin Berlin CVK*

**Background:**

Patients after liver transplantation (LT) have an increased risk for malignancies. This includes tumors of the upper gastrointestinal tract. We identified and analyzed all patients who developed a tumour of the upper gastrointestinal tract after perceiving LT at our institution. Aim of this study is to describe the characteristics of the affected patients and the outcome after therapy.

**Materials and methods:**

Between 1988 and July 2018 2855 LTs were performed at Charité Campus Virchow-Klinikum. All patients with diagnosed esophageal or gastric cancer after LT were identified. We analyzed the type of tumour, staging, grading, oncological as well as type of surgical therapy and survival.

**Results:**

A total of 23 patients developed a tumour of the upper gastrointestinal tract after LT. In the LT cohort the male ratio was 60.1% while LT patients with upper GI tumors were all male . In 22 cases LT had been necessary due to alcoholic cirrhosis, 6 patients had HCV-coinfection and one patient showed an additional α1-antitrypsin deficiency. The only patient without alcoholic cirrhosis suffered from cryptogenic cirrhosis. In the complete LT cohort alcoholic cirrhosis was found in 21.8% of patients. The mean age at the time of the LT was 52.9 years (CI 39-61 years). The mean age at time of cancer diagnosis was 61.8 years (CI 55.9-67.3). 20 patients suffered from esophageal cancer, while three patients developed gastric cancer. 15 tumours were squamous cell carcinomas, six adenocarcinomas and one tumour of neuroendocrine origin. All of these cases were diagnosed in an advanced UICC stage (III or greater) of which seven were in metastatic stage IV. 14 patients received therapy including radical surgery. Ivor Lewis esophagectomy was performed in 10 cases. One patient received McKewon esophagectomy and three patients underwent gastrectomy. The average survival after cancer diagnosis was 39 months (CI 7.7-70.7). Patients who underwent surgery showed a prolonged survival, lasting on average 50 months (CI 5.0-94.8), whereas patients with conservative treatments survived on average 17 months (CI 8.3-25.8).

**Conclusion:**

The 23 surveyed patients with tumours of the upper gastrointestinal tract after LT were all male. Another risk factor is alcoholic liver disease affecting 22 of 23 patients. The incidence of upper GI tumors is strongly elevated among LT patients. Outcome is slightly improved in the surgery group with few patients showing long time survival. A more aggressive endoscopy program for LT patients should be implemented to diagnose tumors at an earlier stage.

## DGAV: Transplantation

### Resolution of pulmonary hypertension after orthotopic liver transplantation in a patient with hereditary haemorrhagic telangiectasia

(Abstract ID: 74)

C. Kim-Fuchs^1^, A. Vogt^2^, M. Lüdi^2^, D. Candinas^1^, V. Banz^1^

^1^*Universitätsklinik für Viszerale Chirurgie und Medizin, Bern*

^2^*Universitätsklinik für Anästhesiologie und Schmerztherapie, Bern*

**Background:**

Hereditary Haemorrhagic Telangiectasia (HHT) is a rare autosomal dominant disorder. Clinical diagnosis of HHT is based on at least three out of four Curacao criteria: 1) spontaneous or recurrent epistaxis 2) multiple telangiectasia especially in the lips, oral mucosa, fingers and nose 3) visceral arteriovenous malformation and 4) positive family history (first-degree relative).

**Materials and methods:**

We report of a 72-year old female suffering from HHT undergoing orthotopic liver transplantation (OLT). Preoperatively, the patient’s left-right shunt due to extensive arteriovenous malformations in the liver and concomitant pulmonary hypertension (PHT) were of great concern, raising discussions about the adequate indication for OLT.

**Results:**

Five years prior to OLT, the patient was diagnosed with increasingly symptomatic PHT "associated with" HHT. Over the years, PHT turned from mild to severe and the patient started developing cardial cachexia with 11kg weight loss in 7 months. Preoperative right heart catheterization yielded a mean PAP of 49mmHg and PCWP of 14mmHg. Medical treatment included Sildenafil (3x10mg) and Lecardipin (1x5mg). PHT remained stable under this treatment but the patient necessitated repeated hospitalization due to sepsis with extensive biliary liver abscesses, the largest being 7.5cm in segment VII. The patient underwent several ERCP and stent placements for extensive choledocho- and hepaticolithiasis with both abnormally dilated and stenotic bile ducts. In time, biliary outflow of the right (posterior) bile ducts was only ensured via a biliary-cutaneous drainage. In an interdisciplinary approach, OLT was evaluated as the only treatment option with a reasonable success rate to overcome the increasingly problematic persistent infectious complications due to bile duct degeneration and vascular compromise.

During the operation the patient’s liver showed classical signs of very extensive intrahepatic arterio-venous shunts with massive, multiple arteriovenous collaterals in the liver hilum. The common hepatic artery and an accessory left hepatic artery were of impressive diameter, with substantial blood flow. Following dissection and ligation of the hepatic arteries, PHT resolved almost instantly. OLT was carried out in standard piggy-back technique. The intra- and postoperative course was uneventful, in particular without reoccurrence of any infectious complications.

**Conclusion:**

This case clearly shows that OLT solves rather than complicates concomitant pulmonary hypertension in patients suffering from HHT. Initial doubts arose as to whether or not she would even qualify for OLT due to severe PHT in the setting of her HHT. However, with no other feasible treatment options and taking into account, that the majority of her PHT would likely resolve after removal of her old liver with the intrahepatic arterio-venous shunts, an interdisciplinary decision was taken to go ahead with the transplant. The very favourable outcome with a now completely asymptomatic patient (no PHT and no infectious problems) shows, that the decision to transplant was justified.

### Impact of eleven prognostic scores on intra- and extrahepatic recurrence of hepatocellular carcinoma after liver transplantation

(Abstract ID: 130)

A. Bauschke^1^, A. Altendorf-Hofmann^1^, H. Kessler^1^, A. Koch^1^, M. Tautenhahn^1^, U. Settmacher^1^

^1^*Universitätsklinikum Jena*

**Background:**

Tumor recurrence is the most frequent cause of death after liver transplantation for hepatocellular carcinoma. We selected ten other prognostic classifications to evaluate their potential to predict the risk of recurrence after LT for HCC as compared to the Milan classification. All of the other scores have not been compared one with another in a single cohort.

**Materials and methods:**

Data of 147 consecutive patients transplanted at our department between 1996 and 2014 were analyzed and staged for morphological and functional scores of underlying liver disease. For long-term follow up, we analyzed separately intrahepatic (within the liver ± distant metastases) and extrahepatic (distant metastases only) recurrence.

**Results:**

The median survival time for all patients was 106 months. The 5- and 10-year observed survival rates were 61% and 43%, respectively. The observed cumulative 5- and 10-year recurrence rates were 37% and 39%, respectively, 10- year intrahepatic and extrahepatic recurrence rates were 12% and 27%, respectively. Median survival time after diagnosis of first recurrence was 7.5 (0-120) months; 2 months and 18 months for all, intra- and extrahepatic recurrence, respectively.

**Conclusion:**

UCSF-, Up to seven-, Shanghai Fudan- or Duvoux-classifications can identify patients with a cumulative 10 year recurrence rate below 20%. The pre-therapeutic AFP level should be considered in addition to the geometry of the intrahepatic lesions.

### Role of CatS/PAR2 for the rejection process in murine renal transplantation

(Abstract ID: 225)

B. Ehle^1^, Y. Lei^2^, S. Kumar^2^, S. Müller^1^, J. Bucher^1^, H.-J. Anders^1^, J. Andrassy^1^

^1^*Uniklinik München, München*

^2^*Klinikum der LMU München*

**Background:**

Cathepsin S is involved in peptide loading to the MHC class II and thus important for antigen presentation. CatS can also be secreted by activated macrophages and neutrophils and activates protease-activated receptor-(PAR)-2 on the endothelial cells. We hypothesized that targeting CatS/Par2 would have a dual suppressive effect on kidney allograft rejection by limiting alloantigen presentation as well as vascular damage.

**Materials and methods:**

Murine kidney transplantation was performed in the syngeneic (B6 to B6) and allogeneic setting (Balb/c to B6). Mice were either treated with CatS inhibitor or vehicle. To study the effects of Par2 deficiency, we performed kidney transplantation using C57BL/6.Par2-/-. Therapeutic effects were assessed by histopathology, immunohistochemistry and RT-PCR.

**Results:**

At 10 days allografts showed severe acute rejection with strongly induced mRNA levels of CatS and numerous inflammatory genes. CatS inhibition significantly ameliorated the acute rejection process. Immunostaining showed suppressed CD8 cell infiltration into grafts, reduced mRNA expression levels of inflammatory genes. Allografts from Par2-deficient mice showed less histological damage and less graft infiltrating CD8 cells as compared to their wildtype controls.

**Conclusion:**

These data show that CatS/Par2 is critically involved in the pathogenesis of allograft rejection

### Successful two-stage liver transplant in children: An option in dire situations

(Abstract ID: 379)

M. Berger^1^, E. Lurz^1^, S. Schäfer^1^, B. Heineking^1^, N. Haas^1^, K. Reiter^1^, M. Guba^1^, J. Werner^1^

^1^*Uniklinik München*

**Background:**

Liver transplant (LT) is the only available cure for end stage liver disease (ESLD). In adults, total hepatectomy with portocaval shunt and subsequent LT as a two-stage procedure following a prolonged anhepatic phase is an accepted approach in the presence of toxic liver syndrome. Although the procedure is well described in adults, literature in children is absent.

**Materials and methods:**

Clinical case report

**Results:**

We here describe a case of a 2-year old boy who, while awaiting liver transplantation for ESLD from biliary atresia and failed Kasai, developed toxic liver syndrome with subsequent multiorgan failure and cardiopulmonary instability. Too sick to transplant, he underwent full hepatectomy with portocaval shunt placement and was taken back to the ICU, were he dramatically stabilized in the subsequent hours. In the mean time, a liver of poor quality had been accepted from a 58 yo old obese female as a bridging organ, and this organ was split ex situ for a left lateral segment. The child was successfully transplanted after an anhepatic phase of 12 hours. As expected, the graft showed only decent synthetic function and poor biliary clearance, however, the child stabilized further to come entirely of pressors and to recover all organ functions. After 14 days, the child underwent a second transplant, which was a left lateral segment from a 26-old male donor from an ex vivo split. Despite excellent graft function, naturally, the child had a challenging postoperative course. He was discharged home in good conditions at 4 months and was well with a fully working graft nine months following transplantation.

**Conclusion:**

In dire situations of toxic liver syndrome with multiorgan failure, total hepatectomy with portocaval shunt placement and LT as a two-stage procedure is a feasible option not only in adults, but also in children.

### Organ shortage, patient survival and waiting list mortality – developments since implementation of MELD-based liver allocation in Germany

(Abstract ID: 384)

P. V. Ritschl^1^

^1^*Charité - Universitätsmedizin Berlin CVK*

**Background:**

The Model for End-stage liver disease (MELD) based allocation system has been implemented in Germany in 2006 in order to reduce waiting list mortality. Purpose of this study is to evaluate post-transplant outcome and waiting list mortality - especially under the aspect of increasing organ shortage in Germany.

**Materials and methods:**

All patients undergoing liver transplantation (LT) in Germany from 2004 to 2015 were assessed retrospectively using the electronic record system of Eurotransplant (ET). The study period was divided into three time sections (A: Pre-MELD 2004-2006; B: post-MELD low donor 2007-2010; C: post-MELD high donor 2011-2012). During this period 21444 patients were registered patients on the waiting list for liver transplantation in Germany.

**Results:**

From 2004 to 2015 a total of 12762 LTs were performed in Germany. After MELD implementation, the median matchMELD at time of LT increased from 17 to 28 in 2015. Donation rate increased after 2004 and remained stable from 2006 to 2011 (around 14 per million inhabitants), but decreased afterwards considerably to 10.4 organ donors/million in 2015. Compensatory, during this period, median donor age increased from 44 to 53 years (p<0.001) and the percentage extended donors (age>=65years) increased from 11.1% to 25.4%. The ratio of used liver donors to reported donors was found to be notably higher in Germany (around 85% since MELD implementation) compared to other ET countries (around 77%). Comparing the different time periods 3-year patient survival in group A was 72.2%, 67.4% group B in group B and then remained constant at 69.1% in group C 2011-2012 (A vs. B, p<0.001; B vs. C, p=0.282). When analyzing patients who died on waiting list or were removed due to poor health status (=mortality), the absolute number was constant over the years (median 388; IQR 334-470; p=0.63). However, the quotient of mortality and actively listed patients increased noticeably from 0.16 to 0.26 (p=0.0045).

**Conclusion:**

Organ shortage lead to looser acceptance of marginal organs since MELD implementation. Despite an initial increase of organ donors survival declined after MELD implementation in Germany with no benefit for waiting list mortality.

### Preemptive chest tube in liver transplantation – a single center experience

(Abstract ID: 386)

P. V. Ritschl^1^

^1^*Charité - Universitätsmedizin Berlin CVK*

**Background:**

Pleural effusion is the most common complication in the immediate postoperative course after liver transplantation and frequently chest drain placement is required. Aim of our study was to investigate the incidence of drainage requiring pleural effusions after liver transplantation and to analyze intervention-related complications.

**Materials and methods:**

This is a retrospective observational study from a high volume liver transplant center in Germany. Adult liver transplant recipients between 2009 and 2016 were analyzed for pleural effusion formation, its therapy and consecutive complications after liver transplantation. Primary outcome was defined as the need for placement of chest drain within the first 10 post-operative days. Furthermore, complications associated with chest drain, occurrence of pneumonia and need for blood products prior to intervention were analyzed.

**Results:**

Overall, 597 patients were included, of which 361 (60.5%) had at least one chest drain within the first 10 post-operative days. Patients with a MELD >25 were more frequently affected (75.7% vs. 56.0%, p<0.001). Typically, chest drains were placed at the intensive care unit (ICU) (68.3%) or in the operating room (14% during transplantation, 11% in the context of reoperations). In total, 97.0% of the patients received a right-sided chest drain, presumably caused by local irritations. Due to poor liver function, one third of patients staying in the ICU needed pre-interventional optimization of coagulation. Out of 361 patients receiving a chest drain 14 (3.7%) suffered from post-interventional hemorrhage and 6 (1.4%) from pneumothorax requiring further medical treatment. Comparing the setting of the placement, less complications were observed when performed in the operating room during transplantation or reoperation as compared to the placement at ICU (1/116 (0.9%) vs. 20/316 (6.3%); p=0.019).

**Conclusion:**

Pleural effusion is the most common complication after liver transplantation requiring intervention in the majority of the cases, especially in high-MELD patients. Routinely placed chest drain during liver transplantation may reduce complications, avoid unnecessary coagulation products and may prevent pneumonia.

### Hand-Assisted laparoscopic donor nephrectomy PERiumbilical versus Pfannenstiel incision and return to normal physical ACTivity (HAPERPACT): Study protocol for a randomized controlled trial

(Abstract ID: 443)

E. Khajeh^1^, Y. Kulu^1^, B. Müller-Stich^1^, O. Ghamarnejad^1^, G. Polychronidis^1^, M. Golriz^1^, F. Nickel^1^, M. Zeier^1^, M. W. Büchler^1^, A. Mehrabi^1^

^1^*Universitätsklinikum Heidelberg*

**Background:**

Hand-assisted laparoscopic living donor nephrectomy (HALDN) using a periumbilical or Pfannenstiel incision was developed to improve donor outcome after a kidney transplant. The aim of this study was to investigate two methods of hand assistance and kidney removal during HALDN and their effect on the time it takes for the donor to return to normal physical activity.

**Materials and methods:**

This study was initiated in November 2017 and is expected to last for 2 years. To be eligible for the study, donors must be more than 20 years of age and must not be receiving permanent pain therapy. Only donors with a single artery and vein in the graft are being enrolled in this trial. Donors with infections or scars in the periumbilical or hypogastric area, bleeding disorders, chronic use of immunosuppressive agents, or active infection will be excluded. Donors will be randomly allocated to either a control arm (periumbilical incision) or an intervention arm (Pfannenstiel incision). The sample size was calculated as 26 organ donors in each group. The primary endpoint is the number of days it takes the donor to return to normal physical activity (up to 4 weeks after the operation). Secondary endpoints are intraoperative outcomes, including estimated blood loss, warm ischemia time, and duration of the operation. Postoperative pain will be assessed using the visual analog scale, rescue analgesic use, and peak expiratory flow rate. Length of hospital stay, physical activity score, time to return to work, donor satisfaction, cosmetic score, postoperative complications, and all-cause mortality in living donors will also be reported. Delayed graft function, primary non-function, serum creatinine levels, and glomerular filtration rate will also be assessed in the recipients after transplantation. The trial was registered at ClinicalTrials.gov under registration number NCT03317184 on 23 October 2017.

**Conclusion:**

This is the first randomized controlled trial to compare the time it takes the living donor to return to normal physical activity after HALDN using two different types of incision. The comprehensive findings of this study will help decide which nephrectomy procedure is best for living donors with regard to patient comfort and satisfaction as well as graft function in the recipient after transplantation.

### Prophylactic Onlay Mesh Reinforcement Reduces Wound Complications after Kidney Transplantation

(Abstract ID: 449)

C. W. Michalski^1^, S. Mohammadi^1^, E. Khajeh^1^, O. Ghamarnejad^1^, M. Sabagh^1^, F. Pianka^1^, M. Golriz^1^, Y. Kulu^1^, F. Kallinowski^1^, M. Zeier^1^, C. Morath^1^, M. K. Diener^1^, M. W. Büchler^1^, A. Mehrabi^1^

^1^*Universitätsklinikum Heidelberg*

**Background:**

Despite advances in surgical methods, incidence of wound complications after kidney transplantation (KTx) is still considerable. In this study we investigated the impact of prophylactic mesh reinforcement on the incidence of wound complications and short-term fascial dehiscence in KTx patients.

**Materials and methods:**

Forty-six patients were included in the no-mesh group and 23 patients received onlay mesh reinforcement. Multivariate analysis was performed to determine predictors of SSI, wound/fascial dehiscence after KTx.

**Results:**

Wound complications were reported in 43.5% and SSI in 33.3% of patients. Longer operation time was independently associated with the incidence of SSI (odds ratio (OR) 2.06, 95% confidence interval (CI) 1.078-3.972, p=0.029). There was no association between mesh placement and SSI incidence (OR 0.26, 95% CI 063-1.084, p=0.064). Mesh placement significantly decreased the risk of wound complications (OR 0.12, 95% CI 0.027-0.627, p=0.011). Lower albumin levels (OR 0.81, 95% CI 0.675-0.975, p=0.026) and longer operation time (OR 2.82, 95%CI 1.263-6.327, p=0.011) were associated with increased incidence of wound complications.

**Conclusion:**

Mesh reinforcement decreased the risk of overall wound complications and short-term fascial dehiscence without increasing the risk of SSI in KTx patients. These findings alongside with long-term results have to be evaluated in a randomized trial setting.

### PREventive Effect of FENestration with and without Clipping on Post-Kidney Transplantation Lymphatic Complications (PREFEN): study protocol for a randomized controlled trial

(Abstract ID: 452)

M. Golriz^1^, E. Khajeh^1^, O. Ghamarnejad^1^, S. Sabagh^1^, S. Mohammadi^1^, M. Zeier^1^, A. Mehrabi^1^

^1^*Universitätsklinikum Heidelberg*

**Background:**

Peritoneal fenestration during kidney transplantation (KTx) is a simple method for preventing post-kidney transplantation lymphatic (PKTL) complications. Recent studies have evaluated the effectiveness of clipping with metallic clips following fenestration on lymphocele formation and lymph leakage after prostate cancer surgery and laparoscopic retroperitoneal lymph node dissection. In the proposed study, we aim to evaluate and compare the effect of fenestration and fenestration with clipping on post-KTx lymphocele and lymphorrhea formations.

**Materials and methods:**

To be eligible for the study, donors must be more than 18 years of age and must not be recipients of KTx from living donors. Reciepients underwent combined transplantation (e.g. pancreas-kidney transplantation) will be excluded from this trial. Recipients with inability to comply with study and/or follow-up procedures will also be excluded. Recipients will be randomly allocated to either a fenestration arm or a fenestration with clipping arm. The sample size was calculated as 39 patients in each group. The primary endpoint is the PKTL complications (e.g. lymphorrhea and lymphocele). Secondary endpoints are intraoperative complications, duration of the operation, length of hospital stay, postoperative complications, all-cause mortality, rate and severity grade of post-KTx lymphorrhea/lymphocele formation, lymphocele/lymphorrhea recurrence, kidney function and retransplantion rate. All recipients will be followed up until postoperative six months. The trial was registered at ClinicalTrials.gov under registration number NCT03682627 on 24 September 2018.

**Conclusion:**

This is the first randomized controlled trial to compare the effect of fenestration and fenestration with clipping on post-KTx lymphocele and lymphorrhea formations. The comprehensive findings of this study will help decide which preventive method is best for reducing the rate/severity grade of PKTL complications.

### SEALIVE: The use of technical vessel-sealing devices for recipient hepatectomy in liver transplantation: Study protocol for a randomized controlled trial

(Abstract ID: 460)

P. Houben^1^, E. Khajeh^1^, U. Hinz^1^, P. Knebel^1^, M. K. Diener^1^, A. Mehrabi^1^

^1^*Universitätsklinikum Heidelberg*

**Background:**

The surgical technique used in liver transplantation has undergone constant evolution in an effort to develop a safe, highly standardized procedure. Despite this, the initial step of recipient hepatectomy has not been the focus of clinical research thus far. Due to advanced coagulopathy in liver transplant recipients, this part of the operation still carries the risk of severe hemorrhage. This trial is designed to compare an electrothermal bipolar vessel sealing device (LigaSure^™^) and an ultrasound dissector (HARMONIC ACE^®^+7) with standard surgical techniques during the recipients' hepatectomy in liver transplantation.

**Materials and methods:**

In a single-center, prospective, randomized, controlled, parallel, three-armed, confirmatory, open trial, LigaSure^™^ and HARMONIC ACE^®^+7 will be compared with standard surgical techniques that use titanium clips and conventional knot-tying ligations during recipient hepatectomy in liver transplantation. Intraoperative total blood loss is the primary endpoint of the trial. Secondary endpoints include blood loss during hepatectomy, the duration of both the hepatectomy and the entire surgical procedure, and blood transfusion requirements of the procedure. To generate reliable data, intraoperative blood loss will be recorded with respect to all rinse fluids during surgery, ascites, and by weighing used swabs. At 80% power and an alpha of 0.025 for both of the experimental groups, 23 subjects will be analyzed per protocol in each study arm in order to detect clinically relevant reduction of intraoperative blood loss. The intention-to-treat analysis will include 69 patients. The follow-up period for each patient will be 90 days for safety reasons, whereas all clinical outcomes will be measured within the first 10 postoperative days. The trial was registered at ClinicalTrials.gov under registration number NCT 03323242 on October 26, 2017.

**Conclusion:**

To our knowledge, this is the first prospective, randomized trial comparing two innovative technical methods of vessel sealing and dissection with standard techniques for recipient hepatectomy. This will be done to detect relevant reduction of intraoperative blood loss during liver transplant. The results of the trial are expected to improve patient outcome and safety after liver transplant and to increase the general safety of this procedure.

### Infections after solid organ transplantation: Is there a benefit for mTOR-I or CNI as basic immunosuppressant? A systematic review and metaanalysis

(Abstract ID: 529)

S. Wolf^1^, M. Lauseker^2^, J. Werner^2^, M. Guba^2^, J. Andrassy^2^

^1^*Universitätsklinikum Augsburg*

^2^*Uniklinik München*

**Background:**

Side effects of the immunosuppressive therapy after solid organ transplantation are well known. Naturally, immunosuppressed patients are more susceptible to infections. Recently, significant benefits were shown for mTOR-Is with respect to CMV infections in comparison to CNIs. With other infections, i.e. pneumonitis the situation may look different.

We aimed to investigate the differences of mTOR-Is and CNIs with respect to the overall incidence of infections after renal transplantation.

**Materials and methods:**

The current literature was searched for prospective randomized controlled trials in solid organ transplantation. There were 526 trials screened of which 12 could be included (pts. = 6246). The 1- year incidence of infections, patient and graft survival was assessed in metaanalyses.

**Results:**

Metaanalysis on 1-year incidence of infections showed a signifant benefit of an mTOR-I based therapy versus CNI’s (RR 0.86, CI 0.77-0.96, p=0.009). After separating trials in mTOR-I based therapy either with or without CNI’s, this effect remained stable only when mTOR-Is were given with CNIs (mTOR-I w/o CNIs vs. CNIs: RR 0.97, CI 0.81-1.16, p=0.73; mTOR-I with CNIs vs. CNIs: RR 0.80, CI 0.69-0.92, p=0.002).

There was no difference between mTOR-I and CNI therapy in respect of patient (RR 1.18, CI 0.84- 1.67, p=0.34) and graft survival (RR 1.05, CI 0.70-1.58,p=0.80).

**Conclusion:**

The numerical incidence of posttransplant infections, as we already know for CMV-infections, seems to be lower under mTOR-I based therapy, especially if combined with CNI’s. Nevertheless a more detailed description of infections in future randomized trials should be pursued. With currently used mTOR-I-based regimen patient and graft survival is not different compared to CNI therapies.

### Acute liver failure due to Thyroid storm in a HIV-positive patient with undiagnosed Grave’s disease

(Abstract ID: 796)

M. Mogl^1^, M. Alkhazraji^1^, E. M. Teegen^1^, C. Bures^1^, R. Öllinger^1^, J. Pratschke^1^, P. E. Goretzki^1^

^1^*Charité - Universitätsmedizin Berlin CVK*

**Background:**

Hyperthyroidism and its treatment may induce liver failure, in extremely rare cases leading to liver transplantation. We report on a case of previously undiagnosed Grave’s disease with thyroid storm leading to acute liver failure and consecutive transplantation. Interestingly, the patient had underlying HIV-infection, which may also lead to elevation of liver enzymes.

**Materials and methods:**

This case describes a 34-year-old female without any prior history of thyroid disease who was referred to our hospital with progressive jaundice and severe headache over the past few days in addition to elevated AST, ALT, and Bilirubin levels.

The patient had been diagnosed with HIV 2 years before. A diagnosis of thyroid storm due to untreated Grave’s disease with consecutive acute liver failure was made.

She had an emergency thyroidectomy, but nevertheless, went into hepatic failure and underwent successful liver transplantation. Since then a regular follow-up over the past 2 years in the out-patient clinic shows stable liver function, normal TSH and excellent quality of life.

**Conclusion:**

Thyroid storm (also known as thyrotoxic crisis) is a rare life-threatening condition representing the severe end of the spectrum of thyrotoxicosis and is characterized by compromised organ function. The mortality rate approaches 10-20%. Thyroid storm occurs in 1-2% of individuals with hyperthyroidism. It is most commonly seen in the context of underlying Grave's hyperthyroidism but can complicate thyrotoxicosis of any etiology.

In the literature, seven cases of thyroid storm and acute liver failure were reported. In only two cases, the patients underwent thyroidectomy and liver transplantation. Both patients survived and had no complications after both surgeries. In the other five cases, the patients were managed only medically and two of the five patients died. None of these patients was diagnosed with an underlying viral infection.

This case represents acute liver failure leading to an emergency thyroidectomy and liver transplantation as a complication of thyroid storm in this HIV-positive female patient with untreated Grave’s disease.

### Liver Transplantation for Budd-Chiari Syndrome: A Single Center Experience

(Abstract ID: 855)

A. Tekbas^1^, R. Fahrner^1^, U. Settmacher^1^

^1^*Universitätsklinikum Jena*

**Background:**

Budd-Chiari Syndrome (BCS) is a rare disease characterized by the obstruction of the hepatic venous outflow from the intrahepatic venules to the suprahepatic portion of the inferior vena cava (IVC). The etiology is multifactorial and can be divided into primary BCS (PBCS) with thrombosis of the hepatic vein due to haematological disease or tumor and secondary BCS (SBCS) due to the compression of the hepatic vein through physical obstruction. The subsequent increase in hepatic sinusoidal pressure leads to portal hypertension and liver failure. Anticoagulant and antithrombotic medications as well as porto-systemic shunting procedures are effective treatments. However, liver transplantation (LT) is the preferable and definitive treatment option with a 5-year survival rate of 70%. The purpose of this retrospective analysis was to report the outcome of liver transplantation for BCS in our center.

**Materials and methods:**

We analysed the medical records of all patients undergoing LT in our Department between 2004 and 2018. 15 from a total of 698 patients (2.1%) were diagnosed with BCS. Our focus was besides of patients characteristics on the longterm survival as well as the investigation of the postoperative outcome regarding a relaps and/or further obligatory medical treatments. Median and standard deviation (SD) express the quantitative variables.

**Results:**

The transplantations were performed between 2006 and 2017. The mean patient age at the time of LT was 48±10 years, corresponding to the mean donor age of 53±15 years. The majority of the patients was female (n=11, 73.3%). The mean pretransplant waiting period was 260±349 days (range 1-1093 days). 7 patients (46.7%) presented an acute liver failure allowing for high urgency (HU) listing. The mean lab Meld score at the time of transplant was 15±8 for all patients, however 20±9 for the HU- listed patients. The majority of the patients suffered from PBCS (n=9, 60%). The reason for developing SBCS in n=6 (40%) patients was polycystic liver disease (n=5, 33.3%) and hemangioma (n=1, 6.7%). Living donor liver transplantation was performed for 2 patients (13.3%), the rest received a cadaveric organ. The last follow up of all patients in our center was in 2018. Multi organ failure with consecutive death occurred in one case of factor V Leiden mutation with necessity of retransplantation due to acute liver failure after delivery. 1 to 12 years after transplantation, 93.3% of the patients present a good quality of life. However, further 2 patients were subject to re-transplantation because of graft failure. All patients transplanted for PBCS continue with anticoagulant therapy. Furthermore, 5 patients with myeloproliferative disease continuously take zytostatic medication (hydroxycarbamide).

**Conclusion:**

We can conclude a good outcome of LT for BCS in our center. However, despite LT a further medical treatment was necessary in all patients with haematological disease. But due to the small number of patients the conclusions are of limited evidence. Therefore, a multi-center analysis would be necessary to enlighten the specific challenge in LT for BCS.

### Persistence of de novo DSA is associated with chronic lung allograft dysfunction and reduced survival after lung transplantation

(Abstract ID: 896)

M. Schmitzer^1^, J. R. Hermawan^1^, L. Strakeljahn^1^, N. Kneidinger^1^, H. Winter^2^, A. Dick^1^, C. Schneider^1^, S. Michel^1^, E. Speck^1^, R. Hatz^2^, J. Behr^1^, T. Kauke^1^

^1^*Uniklinik München*

^2^*Universitätklinikum Heidelberg*

**Background:**

De novo donor-specific anti-HLA-antibodies (dnDSA) appear frequently after lung transplantation. The impact of dnDSA on chronic lung allograft dysfunction (CLAD) and survival is still a matter of debate. In recent studies there was a growing interest in the course over time of dnDSA after transplantation.

**Materials and methods:**

We investigated the clinical relevance of HLA-antibodies on lung allograft outcome prospectively in 89 recipients who were transplanted between 2013 and 2015. The median follow-up time was 42 months with a minimum follow-up time of 3 years per patient.The presence of HLA-antibodies was analyzed by Luminex Single Antigen Bead assay regular prior and after transplantation (3 weeks, 3, 6, 9 and 12 months and then every 6 months) and on demand in case of graft dysfunction.

**Results:**

After transplantation 37% of the patients (n=33) developed dnDSA. In 12 of these patients (36%) dnDSA persisted throughout the surveillance period whereas in 20 of these patients (60%) dnDSA disappeared after a median of 139 days. In one patient transient DSA turned up again. Interestingly, 78% of dnDSA appeared within the first year after transplantation and time to first DSA appearance was shorter in patients with transient compared to patients with persistent DSA (median 42 days vs. 242 days; p=0.076). The immunosuppressive regimen was changed more frequently from tacrolimus to cyclosporine A in patients with dnDSA (p=0.031).

Bronchiolitis obliterans syndrome (BOS) occurred in 18% of dnDSA negative patients and in 33% of dnDSA positive patients (p=0.142). Patients with persistent DSA developed BOS significantly more often compared to DSA negative patients (56% vs. 18%, p=0.030). There was no statistical difference between one year and three year survival of patients with and without dnDSA (88% vs. 91% and 74% vs. 75%). Remarkably, one year and three year survival of patients with persistent DSA was only 67% and 46% respectively. The Kaplan-Meier survival analysis showed a significant reduced survival of patients with persistent DSA compared to patients with transient DSA and without DSA (log-rank p=0.001 and p=0.012).

**Conclusion:**

Persistence of de novo DSA after lung transplantation is an important risk factor for dismal survival and is associated with a higher incidence of CLAD after lung transplantation.

### Long term expirience with the EUROTRANSPLANT Senior Program (ESP)

(Abstract ID: 912)

H.-M. Tautenhahn^1^, S. Brückner^2^, H.-M. Hau^2^, C. Pipiale^2^, P. Felgendreff^1^, M. Bartels^3^, D. Seehofer^1^, U. Settmacher^1^

^1^*Universitätsklinikum Jena*

^2^*Universitätsklinikum Leipzig*

^3^*Helios Park-Klinikum Leipzig*

**Background:**

The ESP (Eurotransplant senior program), introduced in 1999 by EUROTRANSPLANT, is supposed to shorten the waiting period for elder recipients. The aim of this retrospective analysis was to evaluate the results of patients in our transplant unit after kidney transplantation with donor/recipient pairs aged 65 years and older, especially regarding outcome, long-term kidney function and complications.

**Materials and methods:**

Among over 850 kidneys transplanted in our centre, 77 (9.1%) patients exceeded an age of 65 years and were included in the ESP. The median age of the recipients was 68 years (SD ±3.5 years), the one of the donors was 71 years (SD ±4.1 years). The median follow-up-time covered 60 months (SD ±44 months).

**Results:**

In average the time from first dialysis to transplantation was 34 months. By topographic allocation and short shipping ways we reached a minimization of cold ischemia time to 10.5 hours (SD ±3.8 hours). In our study group we detected the following postoperative complications: 15 patients (19.5%) suffered from postoperative bleeding, 5 (5.2%) suffered from graft thrombosis, 22 (28.6%) showed disorders of wound healing, 12 (15.6%) lymphoceles and 2 (2.6%) deep vein thrombosis. After transplantation 16 patients (20.8%) required dialysis again. At the last follow-up the median creatinine level was 195.1 μmol/l (SD ±90.2μmol/l). The survival rate within the median follow-up-time amounted to 66.2%.

**Conclusion:**

In spite of a higher complication rate, caused mainly by atherosclerotic vascular disease in donor as well as in recipient, the patient and transplant survival is comparable with the one from younger patients. Therefore, ESP became routine in our centre. Our results suggest that short cold ischemia time and short waiting time are the main parameters predicting a good patient and transplant survival.

### Postoperative infections following intestinal transplantation

(Abstract ID: 913)

B. Kern^1^, A. Moll^1^, B. Sawitzki^1^, A. Pascher^1^, J. Pratschke^1^, U. A. Gerlach^1^

^1^*Charité - Universitätsmedizin Berlin CVK*

**Background:**

Due to the high immunosuppression infections still account for major morbidity in the short- and longterm after intestinal (ITX) or multivisceral transplantation (MVTX) and are reported to be the single most relevant cause of death within the first year.

**Materials and methods:**

Retrospectively, we analysed the 1-year postoperative course of all patients who had undergone ITX or MVTX between 2000-2017. Bacterial, viral and fungal infections were studied considering time of onset, duration, treatment, immunosuppression, rejection rate and outcome.

**Results:**

41 patients (median age 37.9±10.1 years) underwent either ITX (n=23) or MVTX (n=18). Induction included Tacrolimus, Steroids and Infliximab, maintenance immunosuppression included Tacrolimus and Mycophenolat Mofetil or Sirolimus/Everolimus. 1-year patient survival was 78%, the 1-year infection-related mortality rate was 7%, in 15% enteral virus infections were mistaken for rejection. Bacterial infection rate was 97% with a peak incidence at 16.2±38.7 days post-TX unrelated to rejections, treatment included resistence-tested double antibiotic regimen, mortality rate was 5%. Viral infection rate was 60% with a peak incidence at 39.6±43.1 days, correlating with antirejection therapy, treatment included virostatic therapy and intravenous immunoglobulins. One patient (2%) died of an EBV-related posttransplant lymphoproliferative disorder. Four patients developed invasive Aspergillosis at 53.1±76.6 days, requiring triple antifungal therapy and surgical debridement, they all survived.

**Conclusion:**

The infection-related mortality rate in our cohort was low. Bacterial infections were common after ITX/MVTX but rarely lethal. Viral infections were associated to antirejection therapy and risked the misdiagnosis of rejection, resulting in overimmunosuppression and higher morbidity. Invasive Aspergillosis was not associated to mortality despite using triple antifungal therapy and surgery.

### Analysis of different types of arterial conduits in liver transplantation and their association with hepatic artery thrombosis and inferior graft and patient survival

(Abstract ID: 918)

S. Chopra^1^, F. Gronau^1^, M. Biebl^1^, J. Pratschke^1^, R. Öllinger^1^

^1^*Charité - Universitätsmedizin Berlin CVK*

**Background:**

During orthotope liver transplantation (LT) in cases with unfavorable arterial conditions, interpositioning of an arterial conduit (AC) that is either anastomosed with the infrarenal (IR) or supraceliac (SC) aorta is a common solution. AC formation is associated with increased rates of Hepatic Artery thrombosis (HAT). An inferior graft and patient survival remains uncertain due to contradictory results of previous studies.

**Materials and methods:**

In this study, 493 Patients who underwent LT between 2010 and 2017 at the Charité - Universitätsmedizin Berlin, Campus Virchow-Klinikum. This analysis includes 58 cases of liver retransplantation. All patients were analyzed and compared regarding the prevalence of HAT, graft and patient survival between the standard revascularization group and the AC-group, as well as the effect of the different types of positioning of the AC.

**Results:**

An AC was used in 23 cases (4.6 %) encompassing 17 patients undergoing liver retransplantation. The use of an AC was not associated with an inferior graft and patient survival, when the AC was anastomosed with the infrarenal aorta, as it was performed in 10 cases. A HAT occurred in 21 cases (4.5 %) of all patients undergoing LT without AC and in 3 cases with an AC (13%) . Graft survival in the conduit group was significantly inferior than in the non-AC-group. Interestingly, the graft survival in the infrarenal conduit subgroup was not significantly lower than in the non-AC-group (p = 0.067).

**Conclusion:**

An AC does not represent a significant risk factor for HAT especially if an infrarenal positioning is chosen and should be considered as an alternative in cases, when standard reconstruction is not possible.

## DGAV: Lower gastro-intestinal tract

### Elective vs. early elective surgery in diverticular disease: A retrospective study on the optimal timing of non-emergency treatment

(Abstract ID: 58)

B. Warwas^1^, B. Schneider^2^

^1^*Universitätsklinikum Bonn*

^2^*GFO Kliniken, Betriebststäte St. Marien, Bonn*

**Background:**

This study set out to compare the in-hospital outcomes of early elective and elective laparoscopic sigmoidectomy due to diverticulitis.

**Materials and methods:**

We examined the data for 378 diverticulitis patients who received an elective laparoscopic sigmoid resection between 2008 and 2012. We divided the patients into two groups: elective (group A, n = 278) and early elective (group B, n = 100). Patients in group A received surgery during the inflammation-free interval, and those in group B immediately after treating the attack with IV antibiotics for a mean period of 8 days (IQR = 3).

**Results:**

Overall mortality was 0%. The mean operation duration was the same in both groups being 77.5 and 80 min respectively. There was no significant difference in the outcomes between the two groups, measured using the Clavien-Dindo classification of surgical complication (CCSC; p = 0.992). A revision due to complications was necessary in 16 cases (group A) and six cases (group B) (p = 0.820). The conversion rate to open surgery was low (six individuals in group A, vs. four in group B; p = 0.331). Patients in group B suffered significantly fewer diverticulitis attacks (three in group A, vs. two in group B; p = 0.026).

**Conclusion:**

Our study showed no difference in outcome between elective and early elective cases. Operation durations were optimal in both cases and were 50% shorter than those recorded in the literature. An early elective operation represents a good treatment option, especially for patients suffering from complicated diverticulitis.

### To what extent is the low anterior resection syndrome (LARS) associated with quality of life as measured with the EORTC C30 and CR38 quality of life questionnaires?

(Abstract ID: 117)

J. Kupsch^1^, S. Stelzner^1^

^1^*Städt. Klinikum Dresden Friedrichstadt*

**Background:**

Treatment of rectal cancer often results in disturbed anorectal function, which can be quantified by the Low Anterior Resection Syndrom (LARS) score. This study investigates the association of impaired anorectal function as measured with the LARS score with quality of life (QoL) as measured with the EORTC-QLQ-C30 and CR38 questionnaires.

**Materials and methods:**

All stoma-free patients who had undergone sphincter preserving surgery for rectal cancer from 2000- 2014 in our institution were retrieved from a prospective database. They were contacted by mail and asked to return the questionnaires. QoL was evaluated in relation to LARS and further patient- and treatment factors using univariate and multivariate analysis.

**Results:**

Of the eligible patients (n=331), 261 (78.8%) responded with a complete LARS score. Mean score for global QoL according to the EORTC-QLQ-C30 questionnaire was 63 ±21 for all patients. If LARS was present, mean score decreased to 59 ±20 in contrast to 69 ±20 in patients without LARS (p<0.001). In regression analysis, LARS was furthermore associated with reduced physical, role, emotional, cognitive, and social functioning as well as impaired body image, more micturition problems, and poorer further perspectives. It was not related to sexual function or sexual problems. The variance explained by LARS in the differences of QoL was approximately 10%.

**Conclusion:**

The presence of LARS after rectal resection for cancer is negatively associated with global health as well as many other aspects of QoL. Preserving anorectal function and treatment of LARS are potential measures to improve QoL in this patient group.

### Influence of sigmoid colectomy on quality of life in patients with uncomplicated and complicated diverticulitis of the colon

(Abstract ID: 163)

M. Sohn^1^

^1^*Klinikum Bogenhausen, München*

**Background:**

Diverticulitis of the sigmoid colon counts to the commonest abdominal disorders in the western world. Allocation for a certain strategy is stage based or individualized, according to the respective national guideline. A meaningful, but so far insufficiently recognized aspect for an individualized therapeutical decision making is patients health related quality of life (QoL). The presented study intends to analyze QoL in patients with conservative respectively surgical treatment for complicated and uncomplicated diverticulitis in the long-term.

**Materials and methods:**

Consecutive patients, hospitalized between October 2009 to November 2015 for primarily conservative treatment of uncomplicated and complicated diverticulitis of the left colon were included into the analysis. Therein, patients with and without elective surgical treatment were identified. Disease severity was staged in accordance to the Classification of Diverticular Disease (CDD) and modified Hinchey classification. Patients with perforated disease and generalized peritonitis were excluded. A retrospective chart review of all included patients was conducted. Moreover, patients were asked by telephone to answer a standardized interview and the Short form 36 questionnaire for assessing current quality of life.

**Results:**

Between October 2009 and November 2015, 392 patients (184 M:208 F, mean age: 60,5 (27-91) underwent overall 429 hospitalizations for primarily conservative treatment of acute diverticulitis. 279 patients (M: 138, F: 141; Age mean 60,5 years, Range 27-91 years) filled out questionnaires regarding quality of life after a median follow-up period of 37.8 months (range 15-85 Months). 62 patients underwent sigmoid resection during the initial treatment. Overall, 99 patients had undergo surgery for diverticular disease between the initial hospital stay and the time of filling out the questionnaire. Distribution of patients according to the CDD Classification is shown in Table 1. In uncomplicated disease, no differences in quality of life were found for non operated and operated patients. In complicated disease with sealed perforation -/+ microabscess (CDD stage 2a) SF36 subscales "social functioning" and "role emotional" were significantly superior in the non operated group. The same was shown in complicated disease with pericolic macroabscess (CDD stage 2b). Therein, non operated patients had significant higher results in the subscales "social functioning", "role emotional" and moreover in the subscale "vitality". Other subscales were without differences for both, CDD stage 2a and 2b.

**Conclusion:**

Elective surgery has no significant positive influence on long-term quality of life in patients with uncomplicated and complicated diverticulitis if perforated disease is excluded. Conservative treatment seems to have partly positive influence on quality of life in patients with compicated disease (CDD 2a and 2b). The number of patients with pericolic abscesses, who underwent non-surgical strategy was to low for the assessment of reliable results. However, indication for elective surgery must be made carefully and restrictive in uncomplicated disease and in case of microabscess due to the limited positive impact on patients wellbeing.

**Picture:**
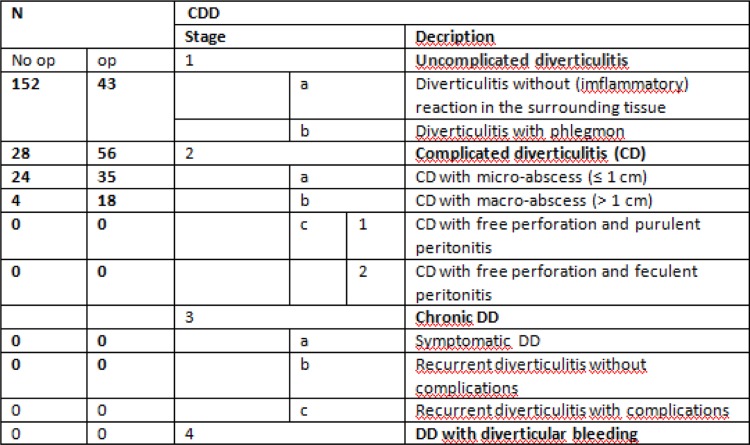
Distribution of CDD stages

### First description of endoscopic negative pressure therapy of the urinary tract with an open-pore film drainage (OFD) – a new urological therapy option for a large urinary bladder defect after abdominoperineal rectal extirpation

(Abstract ID: 173)

G. Loske^1^, R.-U. Kiesow^1^, M. Kurzidem^1^, T. Schorsch^1^, C. T. Müller^1^

^1^*Katholisches Marienkrankenhaus Hamburg gGmbH*

**Background:**

Fistulas of the urinary bladder are a rare complication of rectal surgery. After abdomino-perineal rectal extirpation due to rectal cancer, an 82-year-old patient developed urinary secretion along the perineal wound. Secretion was caused from a large bladder defect of the Trigonum vesicae. Passive transurethral, suprapubic and mono-J. Discharge of urine were without any therapeutic success.

Endoscopic negative pressure therapy was adapted for intravesical application. A new small-caliber open-pore film drainage (OFD) enables negative pressure treatment in the bladder. The OFD can be inserted through small openings, therefore it can be introduced via a suprapubic access into the bladder.

**Materials and methods:**

The OFD was made from a thin open-pore double-layered film (Suprasorb^®^ CNP, Drainage Film, Lohmann & Rauscher International GmbH & Co. KG, 56579 Rengsdorf) and a drainage (Ventrol, 18 Char, 120 cm, Mallinckrodt Medical, Ireland). The distal end of the drainage was wrapped once with the drainage film which was fixed with a suture.

Using conventional endoscopic techniques, the OFD was introduced into the bladder and discharged from the suprapubic incision. OFD was connected to an electronic vacuum pump and a continuous suction in the bladder was applied to the drain the urine in an active manner with negative pressure.

**Results:**

Immediately after application of the negative pressure, the urinary bladder collapsed, and the urine was completely drained in active manner with the OFD and passively via the transurethral Mono-J catheters. The perineal urine secretion of the wound ceased immediately after the start of therapy. After 18 days of urinary negative pressure therapy, the defect was healed to the sacral cavity, the perineal fistula, and the large bladder defect. Then negative pressure application therapy was stopped, and the OFD was changed to a conventual suprapubic bladder catheter. The OFD was changed once during the treatment period. After removal of all catheters, the patient had a normal void.

**Conclusion:**

The OFD allows negative pressure application in the urinary bladder for active urine diversion. Urine can now be permanently and completely drained, even against gravity and capillary force. A negative pressure therapy of the urinary tract was made possible. This opens up new urological therapy options, which will be developed in the future.

**Picture:**
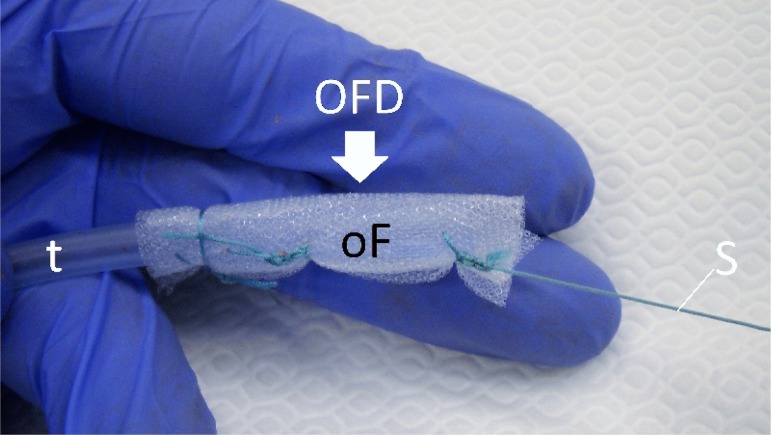
Open-pore film drainage (OFD) was used as an active urine-catheter in order to drain urine in active manner with negative pressure. OFD has a small caliber (5mm). It can be placed suprapubic. For construction a thin double-layered open-pore film (oF) was wrapped surround the distal end of a drainage tube.

### Optimised surgery and MRI-based multimodal therapy in rectal carcinoma: Quality indicators in the OCUM-trial

(Abstract ID: 218)

S. Merkel^1^, R. Ruppert^2^, H. Ptok^3^, J. Straßburg^4^, C. A. Maurer^5^, P. Brosi^6^, J. Sauer^7^, J. Baral^8^, M. Kreis^9^, D. Wollschläger^10^, P. Hermanek^1^, T. Junginger^10^

^1^*Universitätsklinikum Erlangen*

^2^*Klinikum Neuperlach*

^3^*Universitätsklinikum Magdeburg*

^4^*Vivantes Klinikum im Friedrichshain, Berlin*

^5^*Hirslanden Klinik Beau-Site, Bern*

^6^*Universitätsspital Zürich*

^7^*Klinikum Hochsauerland, Arnsberg*

^8^*Städtisches Klinikum Karlsruhe*

^9^*Charité - Unversitätsmedizin Berlin CBF*

^10^*Johannes Gutenberg-Universität Mainz*

**Background:**

Based on pre-therapeutic magnetic resonance imaging (MRI) a restrictive use of neoadjuvant chemoradiation (nCRT) in rectal cancer was examined in a prospective multicentre observational study. The strict observation of the quality indicators is the prerequisite of this study.

**Materials and methods:**

1021 patients with the following inclusion criteria were analysed: Rectal carcinoma cT2-cT4, any cN, cM0, treatment between 2007 and 2015. Carcinomas in the middle and lower third with a minimal distance of <=1mm from the mesorectal fascia, all cT4 carcinomas and cT3 carcinomas of the lower third were classified high risk and received nCRT followed by total mesorectal excision (TME). All other patients with a minimal distance >1mm and those in the upper third were classified low risk and underwent TME alone. Quality indicators of process and outcome quality were analysed. For the analyses of outcome quality 545 patients with a possible follow-up of at least 3 years were included.

**Results:**

328 patients (32.1%) had nCRT followed by surgery, 493 patients (48.3%) had primary surgery, while there were 200 patients (19.6%) with protocol deviations.

The median number of regional lymph nodes examined was 20 (1-79). It was significantly lower in patients with nCRT before surgery than in patients with primary surgery (median 18 vs 23; p<0.001)

154 patients (15.1%) had an abdominoperineal excision (APE). APEs were significantly more frequent in patients with nCRT (22.9%) and in patients with protocol deviation (27.5%) than in low risk patients who had primary surgery according to the protocol (4.9%).

A good TME quality (mesorectal or intramesorectal plane) was confirmed by the pathologists in 1004 specimens (98.3%). This was more frequently in patients of the primary surgery group (99.8%) than in patients with nCRT (97.3%) or protocol deviations (96.5%).

A negative pathologic circumferential margin (pCRM) resulted in 93.8%. Again this result was more frequently in patients who had primary surgery (97.8%) compared with nCRT followed by surgery (90.5%) or protocol deviations (89.5%).

R0 resection was possible in 997 patients (97.6%). It was highest in patients with primary surgery (99.0%) compared to patients with nCRT (96.0%) or protocol deviations (97.0%).

In the following quality indicators no significant difference between the three groups were found: anastomotic leak (overall 3.3%), postoperative morbidity (27.8%), reoperation (9.2%), relaparotomy (7.2%), in-hospital mortality (1.4%), 30-day mortality (1.2%), 90-day mortality (1.9%).

The 3-year rate of locoregional recurrences (LR) of all patients was 2.4%. While it was very low in patients treated according to the study protocol (nCRT followed by surgery 1.2%; primary surgery 1.3%) it was significantly higher in patients with protocol deviations (6.3%; p=0.009). This was due to patients who had surgery alone instead of surgery after nCRT (LR 14.3%) not to patients with nCRT or nRT instead of primary surgery (LR 0%).

The 3-year rate of disease-free survival for all patients was 79.7%. It was highest in the low risk group with primary surgery (82.6%) and lower in the group with nCRT (76.3%) or protocol deviation (78.6%). The difference between the three groups failed the level of significance.

**Table:** 

		n	3-year rate	95% CI	p
Locoregional recurrence	nCRT followed by surgery	174	1.2%	0-3.0	
	Primary surgery	254	1.3%	0-2.7	
	Protocol deviation	117	6.3%	1.8-10.8	0.009
Disease-free survival	nCRT followed by surgery	174	76.3	70.0-82.6	
	Primary surgery	254	82.6	77.9-87.3	
	Protocol deviation	117	78.6	71.2-86.0	0.098

**Conclusion:**

The quality indicators of process and outcome quality confirmed excellent surgical quality in this study. As expected, there were even better results in the low risk group.

### Restriction of neoadjuvant radiochemotherapy to high-risk patients with rectal carcinoma: Oncological outcome in the OCUM rectal cancer trial

(Abstract ID: 282)

R. Ruppert^1^, T. Junginger^2^, H. Ptok^3^, J. Straßburg^4^, C. A. Maurer^5^, P. Brosi^6^, J. Sauer^7^, J. Baral^8^, M. Kreis^9^, D. Wollschläger^1^, P. Hermanek^10^, S. Merkel^10^

^1^*Klinikum Neuperlach, München*

^2^*Universitätsmedizin Mainz*

^3^*Universitätsklinikum Magdeburg*

^4^*Vivantes Klinikum im Friedrichshain, Berlin*

^5^*Hirslanden Klinik Beau-Site, Bern*

^6^*Universitätsspital Zürich*

^7^*Klinikum Hochsauerland, Arnsberg*

^8^*Städtisches Klinikum Karlsruhe*

^9^*Charité - Unversitätsmedizin Berlin CBF*

^10^*Universitätsklinikum Erlangen*

**Background:**

Based on pre-therapeutic magnetic resonance imaging (MRI) a restrictive use of neoadjuvant chemoradiation (nCRT) in rectal cancer was examined in a prospective multicentre observational study. Oncologic results were examined with special interest on locoregional and distant recurrences.

**Materials and methods:**

545 patients with the following inclusion criteria were analysed: Rectal carcinoma cT2-cT4, any cN, cM0, treatment between 2007 and 2012, possible follow-up of at least 3 years. Carcinomas in the middle and lower third with a minimal distance of <=1mm from the mesorectal fascia, all cT4 carcinomas and cT3 carcinomas of the lower third were classified high risk and received nCRT followed by total mesorectal excision (TME). All other patients with a minimal distance >1mm and those in the upper third were classified low risk and underwent TME alone.

**Results:**

The 3-year rate of locoregional recurrences (LR) of all patients was 2.4%. While it was very low in patients treated according to the study protocol (nCRT followed by surgery 1.2%; primary surgery 1.3%) it was significantly higher in patients with protocol deviations (6.3%; p=0.009). This was due to patients who had surgery only instead of surgery after nCRT (LR 14.3%) not to patients with nCRT or nRT instead of primary surgery (LR 0%). Additional significant risk factors for locoregional recurrences in univariate analyses were a positive circumferential resection margin in MRI (mrCRM) (LR 5.1 vs 1.3%; p<0.001), positive pathological circumferential resection margin (pCRM: LR 12.3 vs 1.5%; p<0.001), pathological stage III or yIII (LR 6.0 and 6.4%) and age >=80 years (LR 11.9%).

The 3-year rate of distant metastases (DM) of all patients was 12.4%. It was 8.9% in low risk patients treated with primary surgery and 17.3% in high risk patients who had nCRT followed by surgery. In patients with protocol deviations it was 13.5% (p=0.018). There was no significant difference between patients who had surgery only instead of surgery after nCRT (DM 17.0%) and patients with nCRT or nRT instead of primary surgery (DM 10.4%; p=0.945%). Additional significant risk factors for distant metastases in univariate analyses were a positive mrCRM (DM 20.0 vs 8.5%; p<0.001), pathological stage III or yIII (DM 20.1 and 35.3%) and abdominoperineal excision (DM 20.8 vs 10.7; p=0.002). A positive pCRM just missed the level of significance (DM 25.2 vs 11.5%; p=0.052).

**Table:** 

		n	3-year rate (%)	95% CI	p
Locoregional recurrences	nCRT followed by surgery	174	1.2	0-3.0	
	Primary surgery	254	1.3	0-2.7	
	Protocol deviations	117	6.3	1.8-10.8	0.009
Distant metastases	nCRT followed by surgery	174	17.3	11.6-23.0	
	Primary surgery	254	8.9	5.4-12.4	
	Protocol deviations	117	13.5	7.0-20.0	0.018

**Conclusion:**

This study justifies the restrictive use of nCRT to patients with a high risk of locoregional recurrences, providing highly accurate MRI diagnostics, TME surgery, standardized pathological examination of resected specimens and a continuous quality management. However, the results of distant metastasis are not satisfactory. A new study concept is currently being developed.

### Quality of primary surgical resection is a key prognostic factor in patients with cytoreductive surgery and HIPEC for peritoneal carcinomatosis from colorectal cancer

(Abstract ID: 303)

C. S. Seefeldt^1^, P. Thomaidis^1^, J. Lange^1^, D. R. Bulian^1^, M. M. Heiss^1^, M. A. Ströhlein^1^

^1^*Kliniken Köln GmbH, Köln*

**Background:**

Cytoreductive surgery with complete macroscopic resection (CC-0/CC-1) and Hyperthermic chemoperfusion have been established as standard treatment for resectable peritoneal carcinomatosis from colorectal cancer. Despite successful complete CC-0 resection, a relevant number of patients show early relapse with limited prognosis. A possible explanation may be patient´s history before CRS: PC is often found as unforeseen diagnosis during elective surgery or emergency surgery when HIPEC treatment is not available and complete cytoreduction is not performed. Therefore especially the role of quality of primary surgery remains unclear. The aim of study is to investigate der prognostic influence of treatment factors like inductive chemotherapy, second look laparotomy with CRS and HIPEC, initial emergency surgery, surgery at presence of ileus and, last but not least, primary R0 resection in comparison to primary incomplete R2 resection.

**Materials and methods:**

We investigated a series of 51 patients with peritoneal carcinomatosis from colon cancer, who all underwent complete cytoreduction (CC-0/CC-1) and HIPEC either during primary surgery (15) or by second look concept (36). Overall survival, PCI, primary R-status, T- and N-Stage, treatment in an emergency surgery setting, extent of PC and systemic chemotherapy were assessed. Log-rank analysis, student´s t-test and multivariate regression analysis were used for statistical calculation.

**Results:**

Primary R2 resection vs. primary complete R0/R1 resection, (18.6 vs. 49.6 months, p=0.001), high PCI (p=0.03) and ileus (26.4 vs. 55.6 months, p=0.007) were found to be associated with impaired survival, whereas other factors like ascites, emergency surgery, primary vs. secondary CRS+HIPEC and inductive chemotherapy had no significant effect. Multivariate regression analysis showed primary incomplete R2 resection vs. complete R0/R1 resection to be an independent prognostic parameter (p=0.003).

**Conclusion:**

The quality of primary surgery in terms of complete cancer resection is a significant prognostic factor in patients with peritoneal carcinomatosis from colon cancer. Secondary CC-0/CC-1 cytoreduction and HIPEC after initial incomplete cancer resection are not able to compensate primary resection failure. Therefore, patients with first diagnosis of peritoneal carcinomatosis during elective or emergency surgery should be either completely resected or transferred to specialized centers, when complete resection is not manageable.

### Influence of the preoperative BMI and weight loss on the oncological outcome after resection of colorectal carcinoma (UICC 0-III)

(Abstract ID: 338)

C. Falch^1^, J. Johannink^1^, T. Skibnewski^1^, A. Königsrainer^1^, A. Kirschniak^1^

^1^*Universitätsklinikum Tübingen*

**Background:**

More than half of German adults are overweight, and the trend is rising. Obesity is a risk factor for the development of colorectal carcinoma. But its influence on the post-therapeutic oncological outcome is still not clear. Preoperative weight loss appears to negatively affect the oncological outcome of tumors in the upper gastrointestinal tract. The data available for colorectal carcinoma are sparse and also not clear. The aim of this study is to investigate the influence of the preoperative body mass index (BMI) and weight loss on the oncological outcome after resection of colorectal carcinomas.

**Materials and methods:**

A retrospective database analysis and follow-up of 901 patients resected for a primary colorectal carcinoma (UICC 0-III) between 2004 and 2014 at the University Hospital of Tübingen was performed. Only patients with clear resection margins (R0) were considered for analysis. The patients were assigned to the groups underweight (UW), normal weight (NW), overweight (OW) or obese (OBESE), based on the preoperative BMI analogous to the WHO classification. A preoperative weight loss was defined as an unwanted weight loss of >=3 kg.

**Results:**

Of 488 patients with a carcinoma of the colon, 2.7% were underweight (BMI 17.9 kg/m2), 41% normal weight (BMI 22.6 kg/m2), 38.7% overweight (BMI 27.4 kg/m2) and 17.6% obese (BMI 32.7 kg/m2). Of these patients, 65 (13.3%) showed preoperative weight loss (6kg (range 3-30kg)). The follow-up period was 44 months. Tumor-free survival of underweight patients was significantly shorter compared to overweight (P = 0.021) and obese patients (P = 0.015) (5-year tumor-free survival: UW 61.5%, NW 80%, OW 83%, OBESE 86%). Of 413 patients with rectal cancer, 1.8% were underweight (BMI 16.8 kg/m2), 38.7% normal weight (BMI 22.8 kg/m2), 38.7% overweight (BMI 26.8 kg/m2) and 20.8% obese (BMI 31.5 kg/m2). Of these patients, 47 (11.4%) had preoperative weight loss (8kg (range 3- 25kg)). The follow-up period was 47 months. Patients with preoperative weight loss had significantly lower overall survival (P = 0.017) and a lower tumor-free survival (P = 0.071) compared to patients without weight loss. In the multivariable Cox regression analysis, preoperative weight loss in colorectal carcinomas is an independent risk factor for tumor recurrence [RR 1.677 (95%CI 1.042-2.698) (P = 0.036)] and mortality [RR 1.486 (05% CI 0.981 2.249) (P = 0.061)].

**Conclusion:**

Both, underweight patients as well as patients with preoperative weight loss show a worse oncological outcome after resection of colorectal carcinomas. Further studies are needed to identify subgroups of patients suffering from colorectal carcinoma who benefit from pre- and perioperative nutrition therapy.

### Damage control surgery versus initial definite surgery in acute bowel perforation: A Single-Center retrospective analysis

(Abstract ID: 344)

R. R. Ossami Saidy^1^, I. Aliyeva^1^, R. Öllinger^1^, F. Aigner^1^, J. Pratschke^1^, W. Schulte^1^

^1^*Charité - Universitätsmedizin Berlin CVK*

**Background:**

Emergent surgery is the standard of care in patients presenting with acute bowel perforation. While basic surgical principles including resection of the injured bowel segment, source control and restoration of gastrointestinal function are generally well accepted, optimal surgical management with respect to the timing of gastrointestinal reconstruction remains controversial and heterogeneous: Initial damage control surgery with delayed reconstruction as well as immediate reconstruction during initial emergent surgery are performed.

**Materials and methods:**

All patients diagnosed with acute, non-traumatic, intestinal bowel perforation who underwent emergent surgery in our center between 01/2012 and 07/2018 (approx. n=600) were retrospectively analyzed regarding initial surgical strategy and subsequent morbidity and mortality (Group DMG_CTRL: patients undergoing initial damage control surgery; Group DEF_SRG: patients undergoing definite bowel reconstruction during initial operation). Clinical, histopathological, microbiological, laboratory findings during initial operation as well as numbers of needed re-operations, severity of surgical complications and length of hospital stay were compared between surgical strategies. Patients suffering from traumatic bowel injury and those that were managed without gastrointestinal resection (e.g., perforation located proximal to the ligament of Treitz) were excluded. Statistical analysis was performed using SPSS.

**Results:**

Preliminary data of n=34 patients showed n=12 in group DMG_CTRL and n=22 in group DEF_SRG with similar median age (60 versus 61 years). In most cases, intestinal perforations were located in the colon (62%, n=21). Severity of peritonitis showed a significant difference between groups with more cases of mild peritonitis in the DEF_SRG group (nDEF_SRG =14 versus nDMG_CTRL =5, p<0.05) and, respectively, more severe peritonitis in the DMG_CTRL group (nDEF_SRG =1 versus nDMG_CTRL =6, p<0.05). Primary skin closure was performed in nDEF_SRG =19 versus nDMG_CTRL =0. Rate of surgical complications showed no significant differences, however, number of follow-up surgeries until final wound closure was achieved differed significantly (DEF_SRG: 1,4 operations vs. DMG_CTRL: 3,25 operations, p<0.05). In initial microbiological findings, non multi-resistant enterococci specimen were most common in both groups, however, follow-up findings after re-operations showed an increase in fungi specimen in the DMG_CTRL group from 25% to 58% of cases but not in the DEF_SRG group (42% to 42% cases).

**Conclusion:**

Different surgical strategies with distinct advantages and disadvantages exist for the treatment of acute bowel perforations. As medical etiologies and patients comorbidities regarding this frequent surgical disease vary substantially, patient subpopulations that benefit from one approach over the other have to be identified using large patient collectives. Here, we aim to identify patient characteristics and risk factors of these two surgical approaches to establish parameters to be used in future prospective randomized clinical trials.

### The Water-holding Procedure: Assessment of continence prior restoring intestinal continuity

(Abstract ID: 392)

F. Schwandner^1^, U. Klimars^1^, M. Gock^1^, E. Klar^1^, F. Kühn^2^

^1^*Universitätsmedizin Rostock*

^2^*Klinikum der LMU München*

**Background:**

A defunctioning stoma can become necessary in a relevant number of patients undergoing gastrointestinal surgery. As a matter of course, patients seek an early closure of the stoma. However, pre-operative management of these patients varies and prediction of continence after stoma removal can become challenging. Patients can be fully continent despite low manometric pressures and vice versa. An easy and reliable way to predict continence after stoma reversal would improve patients` management and outcome.Although frequently performed in various surgical centers in Germany, there is no published data on the water-holding test. Hence, this is the first study evaluating the role of the test in clinical practice.

**Materials and methods:**

We performed a prospective clinical study to evaluate the role of the water-holding procedure as a predictor of continence prior to stoma reversal. Fifty-two patients with an ostomy who successfully passed the water holding test were consecutively enrolled in this study between October 2013 and February 2016. Anorectal manometry was performed in all patients prior to stoma reversal. After stoma removal patients were followed up for 6 months. Postoperative incontinence was determined using the Wexner incontinence score.

**Results:**

A total of 52 patients (38 males, 14 females) were included at an average age of 59 (range: 33-83) years. Most frequent indications for intestinal deviation were rectal cancer surgery, IBD-related surgery or surgery for diverticular disease. Low anterior rectal resection was performed in 17 patients (32.7%), a proctocolectomy in 9 (17.3%), a colectomy in 9 (17.3%) and a recto-sigmoid resection in 7 patients (13.5%). Median time from stoma creation to reversal was 206 days (range; 48-871 days). All patients had successfully passed the standardized water-holding test. At the same time, around 70% of patients (36 of 52) had low preoperative manometric pressure values. The average postoperative Wexner incontinence score was at 3.3, 2.7 and 2.3 after 14, 60 and 180 days, respectively. After 6 months, all patients reported on a sufficient sphincter function.

**Conclusion:**

A standardized water-holding test can function as an easy and reliable method to assess fecal continence before stoma reversal. In case of a sufficient water-holding test the risk for postoperative anal incontinence seems to be low. On the other hand, pre-operative manometric pressure levels do not appear to predict post-operative continence.

### Hand-sewn vs. Stapled Anastomoses – hyperspectral analyse of perfusion

(Abstract ID: 425)

B. Jansen-Winkeln^1^, R. Thieme^1^, I. Gockel^1^, H. Köhler^2^

^1^*Universitätsklinikum Leipzig AöR*

^2^*Universität Leipzig*

**Background:**

Even though hand-sewn and stapled anastomoses are daily routine for surgeons, we know little about the differences between these techniques. Clinically, both techniques are equivalent in anatomically well accessible areas. Hyperspectral imaging enables us to investigate the tissue perfusion contactless.

**Materials and methods:**

We examined 30 anastomoses in three groups Side-to-side stapled ileo-ileal anastomosis (n = 10), end-to-end hand sewn ileo-ileal anastomosis (n = 10) and hand-sewn ileo-colic anastomosis (n = 10) with intraoperative hyperspectral imaging to see differences between hand-sewn and stapled anastomosis.

**Results:**

In all patients the anastomoses healed without problems. The mean StO2 of the ROI in stapled anastomosis was significantly higher (median, 78%; range, 65% to 93%) compared to hand-sewn ileo-ileal anastomoses (p = 0.007) and ileo-colic anastomoses (p < 0.001).

**Conclusion:**

Intraoperative HSI showed significant differences of tissue oxygenation in hand-sewn and stapled gastrointestinal anastomosis. Limited blood flow does not seem to be the only risk factor for anastomotic leakage.

### Identification of the ideal anastomotic position with Hyperspectral Imaging

(Abstract ID: 426)

B. Jansen-Winkeln^1^, N. Holfert^1^, H. Köhler^1^, C. Chalopin^2^, I. Gockel^1^

^1^*Universitätsklinikum Leipzig AöR*

^2^*Universität Leipzig*

**Background:**

Determination of the resection margin during colorectal resections depends on the experience of the surgeon and macroscopic colon perfusion in the area of the associated mesocolon. Hyperspectral imaging is able to examine the tissue perfusion contactless and non-invasively.

**Materials and methods:**

The hyperspectral camera can record a complete light spectrum from 500 nm to 1000 nm at any point of the image. Evaluation of the absorption spectra of the remitted light allows to draw conclusions about several tissue parameters. Analysis software provides a RGB image and 4 false color images representing physiologic parameters of the recorded tissue area intraoperatively. These parameters contain tissue oxygenation (StO2), perfusion- (NIR Perfusion Index), organ hemoglobin- (OHI) and tissue water index (TWI).

We measured the perfusion of the intestine of 20 patients after central devascularization and before seperation of the marginal artery. In a second step, the marginal arcade was severed and the surgical resection line marked. During the following 15 minutes, HSI was performed each minute to assess the above mentioned parameters.

**Results:**

The hyperspectral camera visualized the margin of perfusion in 18 out of 20 patients precisely. In the other 2 patients, the perfusion difference could be displayed with additional evaluation software. The determination of the surgical resection area was corrected proximally in 5 cases. The biggest drop in perfusion after devascularization took place in less than 2 minutes.

**Conclusion:**

Determination of the resection margin by HSI provides the surgeon with an objective desicion aid for assessment of the best possible perfusion and ideal anastomotic area in colorectal surgery.

### Visualisation of induced reversible and persistend jejunal ischemia through hyperspectral Imaging

(Abstract ID: 430)

A. Studier-Fischer^1^, F. M. Schwab^1^, K.-F. Kowalewski^1^, H. G. Kenngott^1^, B. P. Müller-Stich^1^, F. Nickel^1^

^1^*Universitätsklinikum Heidelberg*

**Background:**

Intraoperative evaluation of tissue perfusion is essential in surgery. A variety of intraoperative imaging techniques that address this issue. They can be separated into methods using ionizing radiation such as X-ray, digital fluoroscopy or CT scans with contrast agent, and methods without radiation such as sonography or selective oximetry. However, there is no real-time imaging technique which is capable of visualizing tissue perfusion and its change over time during surgery. Several years ago the fluorescent dye indocyanine green (ICG) was introduced to provide a solution. Although it founded the idea of fluorescence-guided surgery (FGS), it is thus far not established for regular operative settings. This was due to a series of challenges such as insufficient optical systems, an unstandardized evaluation, the limitation of being able to measure only one single perfusion situation after ICG application, and finally the financial costs of the substance.

Hyperspectral Imaging (HSI) addresses the majority of these difficulties in that it has powerful non-laparoscopic optics for detection of diverse tissue parameters, mathematical algorithms for the depiction of numerical values, the possibility of multiple measurements for changes over time as well as independence from contrast agents. The aim of this study was to visualize intestinal tissue perfusion over time and to compare results to ICG evaluation.

**Materials and methods:**

For this experimental animal study in a porcine model, the TIVITA^™^ tissue system HSI Camera from Diaspective Vision GmbH was used. After laparotomy, the jejunum was identified and positioned for sequential measurements. Mesotomy was performed over a distance of 15 cm with Ligasure^®^ for irreversible ischemia on a first jejunal segment, while surgical clamps were placed on the mesentery of a second jejunal segment in order to cause reversible ischemia.

Both segments were then recorded every 2 minutes for 20 minutes in total. Next, the clamps causing reversible ischemia were released to allow for reperfusion of jejunal segment number 2, while jejunal segment number 1 was still irreversibly ischemic. Again, both segments were recorded every 2 minutes for 20 minutes. The jejunum was left to rest within the abdominal cavity for a duration of 4 hours until ischemia had fully developed. Finally, both jejunal parts were again positioned for sequential measurements and 5 mg of ICG i.v. were applied.

**Results:**

In 2 calculated index pictures from HSI (stO2 index for superficial oxygen saturation and NIR index = near-infrared for deep perfusion) strong changes were seen during ischemia and reperfusion especially with regards to reactive hyperaemia. When comparing HSI results (Fig.1e) to results from visual inspection (Fig.1f) and results from ICG (Fig.1g), HSI was the most critical imaging technique. Visual inspection was less critical and ICG was the least critical for evaluation of sufficient perfusion. Consequently, in case of a resection of ischemic intestinal segments, HSI evaluation is advocating for the best surgical outcome. The hypothesis of these preliminary data needs to be further investigated in larger clinical studies and correlated to clinical outcomes.

**Conclusion:**

HSI is a ready-to-use system for intraoperative real-time evaluation of tissue perfusion for specific organs, predominantly jejunum, ileum and colon. Its sensitivity for ischemic regions is greater than visual inspection or ICG application and has the potential to be beneficial for surgical outcomes.

**Picture:**
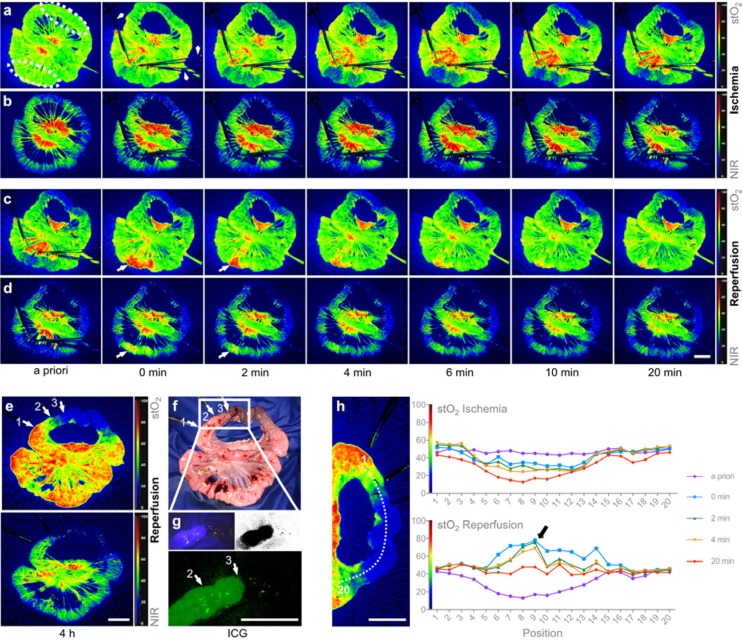
HSI for jejunal ischemia

### LARS is associated with lower anastomoses but not with the transanal approach in patients undergoing rectal cancer resection

(Abstract ID: 494)

F. Filips^1^, T. Haltmeier^2^, D. Candinas^1^, L. Brügger^1^, P. Studer^1^

^1^*University Hospital Bern*

**Background:**

Low Anterior Resection Syndrome (LARS) is a frequentl defecatory disorder in patients after Low Anterior Resection (LAR) with Total Mesorectal Excision (TME). Over the past years, transanal (ta) TME evolved as a promising technique for low rectal pathologies to overcome the difficulties encountered with the abdominal approach in a narrow pelvis. However, the impact of the transanal approach on functional outcomes has not been sufficiently investigated yet. This study evaluates LARS scores in our cohort of patients after taTME compared to a historic group that underwent LAR using the abdominal approach.

**Materials and methods:**

A total of 41 patients (n=21 taTME, n=20 abdominal approach) with low and mid rectal adenocarcinoma were included. LARS scores are routinely recorded in our outpatient clinic after LAR/TME. LARS scores of the historic group with abdominal approach were collected by chart review and telephone interviews. LARS scores 6 month after reversal of the protective ileostomy were analyzed for this study.

**Results:**

Overall, 19.5% of patients presented without LARS symptoms at the 6-month follow-up. 43.9% presented with minor and 36.6% with major LARS. No significant association of the T-stage, N-stage and neo-adjuvant radiotherapy and LARS scores was found. Also, LARS scores in patients with colo-anal hand-sewn compared to stapled anastomosis were not significantly different (30.6±8.9 vs. 25.9±7.5, p=0.161). The mean distance of the anastomosis from the anal verge was 3.9±2.0cm. The anastomosis was significantly lower in the taTME group compared to the control group (3.0±1.6 vs. 4.6±2.0cm, p=0.007). LARS scores were significantly higher in patients with an anastomosis <= 4cm from the anal verge compared to patients with an anastomosis > 4cm (30.0±7.6 vs. 20.0±8.1, p=0.001). However, LARS scores in the taTME group compared to the control group did not show a statistical difference (29.5±9.6 vs. 24.9±7.9, p=0.104).

**Conclusion:**

Overall, the majority of patients in this small cohort presented with some degree of LARS symptoms after LAR. As previously described, LARS scores significantly increased with an anastomosis 4cm from the anal verge or below. Although anastomoses were lower in the taTME compared to the control group, taTME was not significantly associated with LARS 6 month after closure of the protective ileostomy. (Another 20 patients will be included into the two cohorts for the DGCH 2019 presentation.)

### Gender Comparison of clinical, histopathological, therapeutical and outcome factors in 185.967 Colon Cancer patients

(Abstract ID: 516)

R. B. Schmuck^1^, M. Gerken^2^, E.-M. Teegen^1^, M. Klinkhammer-Schalke^2^, I. Krabs^1^, F. Aigner^1^, J. Pratschke^1^, I. Rau^1^, S. Benz^1^

^1^*Charité - Universitätsmedizin Berlin CVK*

^2^*Arbeitsgemeinschaft Deutscher Tumorzentren e.V., Berin*

**Background:**

Colorectal carcinomas represent the third most cause of cancer related deaths in Germany both in man and women. The risk is significantly higher in men compared to women. Nevertheless, detailed gender comparisons between both sexes are scarce. As gender is a well-established crucial factor for outcome in other diseases, this analysis aimed to compare clinical, histopathological, therapeutical and outcome factors in patients diagnosed with colon cancer between 2000 and 2016.

**Materials and methods:**

The common dataset of colorectal cancer patients of the quality conference of the DKG (Deutsche Krebs Gesellschaft) and ADT (Arbeitsgemeinschaft Deutscher Tumorzentren) was analyzed, only patients suffering from colon cancer were included in the study. This retrospective population-based cohort study includes patients diagnosed with colon cancer in Germany between 2000 and 2016 included in the datasets. All cases of verified adenocarcinoma of the colon without second tumor and with radical surgery were considered as well as a separate group of patients with UICC stage I-III only (ACO).

**Results:**

A total of 185.967 patients were included in the study of which 85.685 were female and 100.282 were male. 116.528 patients were included in the ACO group. The portion of women diagnosed with colon cancer decreased from 2000 to 2016 and the portion of very old patients is especially high in women. The localization in women tends to be right-sided and in women, second tumors of colorectal origin are less common.

Women tend to have more neuroendocrine carcinomas, a higher tumor grading and a higher UICC stage (especially III nodal-positive) at diagnosis of primary colon cancer. We could detect a slightly better overall and recurrence free survival in women in the ACO group compared to men in univariable regression analyses even though women do receive chemotherapy less frequently compared to men. After risk-adjustment in multivariable regression analysis survival advantage in women is even more pronounced and highly significant (HR 0.795, lower 95% 0.780, upper 95% 0.811).

**Conclusion:**

We could detect various variables differing significantly between men and women regarding clinical, histopathological, therapeutical and outcome factors. We believe that it is crucial to consider gender as a key factor in diagnosis and treatment of colon cancer. Further more, we recommend to stratify randomized trials for gender.

### Enterocutaneous fistula in severely active Crohn’s disease: Preoperative anti-TNF-alpha treatment to limit bowel resection – report of a case

(Abstract ID: 575)

P. Wilhelm^1^, A. Kirschniak^2^, J. Johannink^2^, S. Kaufmann^2^, T. Klag^2^, J. Wehkamp^2^, C. Falch^2^

^1^*Universitätsklinikum Tübingen*

^2^*University of Tuebingen*

**Background:**

Interenteric or enterocutaneous fistulas based on Crohn's disease often require extended or repeated intestinal resections which are associated with high morbidity and may result in a functional small intestine. Anti-inflammatory therapy with tumor necrosis factor alpha inhibitors (anti-TNF-alpha) has positive impact on fistulizing Crohn's disease. We describe a case of a 32-year-old male with an eight-year history of Crohn's disease suffering from enterocutaneous fistulas in severely active inflammation.

**Materials and methods:**

The patient’s clinical course and data of therapy monitoring before bowel resection were reviewed and compared to the pre-therapeutic findings. The literature on anti-TNF-alpha treatment in active inflammation was surveyed. In addition, the reports of surgery and histopathological work-up were evaluated and a clinical follow-up was performed.

**Results:**

A 32-year-old male with an eight-year history of Crohn's disease and condition after previous ileocaecal and sigmoid resection at the age of 28 presented with increasing pain in the middle-right abdomen for four weeks. Laboratory and radiologic assessment detected elevated C-reactive protein and presence of a conglomerate of inflammatory thickened and narrowed small intestine involving the neoterminal ileum and enteroenteric fistulas as well as a prestenotic distention of the small intestine. Ileocolonoscopy showed a stenosing inflammation of the neoterminal ileum and normal mucosa in the colon. The histopathological findings of biopsies taken presented ulceration and atrophia of the neoterminal ileum. After initial antiinfective therapy as a result of an interdisciplinary decision preoperative anti-TNF-alpha treatment was performed to achieve limited bowel resection. After declining of inflammation, limited bowel resection was carried out successfully.

**Conclusion:**

Strategies for limiting the extent of resection in cases of interenteric or enterocutaneous fistulas based on Crohn's disease are urgently necessary. Anti-inflammatory therapy with tumor necrosis factor alpha inhibitors (anti-TNF-alpha) has positive impact on fistulizing Crohn's disease. Therefore, preoperative therapy with anti-TNF-alpha might potentially reduce inflammation in severely active Crohn’s disease and subsequently limit the extent of resection. We describe an impressive case in which such therapeutic approach was carried out.

### Accuracy of MRI in preoperative staging of middle and low rectal cancer

(Abstract ID: 596)

L. Danihel^1^, M. Rajcok^1^, K. Mosna^1^, V. Belan^2^, M. Kukucka^1^, M. Novisedlakova^3^, I. Urbanova^4^, M. Schnorrer^1^

^1^*Faculty of Medicine, Comenius University in Bratislava*

^2^*Dr. Magnet – Magnetic Resonance Kramáre, Bratislava*

^3^*Oncology department University hospital, Bratislava*

^4^*Klinikum Traunstein, Traunstein*

**Background:**

Multimodal treatment of rectal cancer, with the combination of neoadjuvant chemoradiotherapy (CRT) followed by surgical resection increases local control in locally advanced tumors and has become the standard approach. Nowadays all patients in stage II and higher are indicated to neoadjuvant therapy. Neoadjuvant therapy poses a great advance in the treatment of colorectal cancer patients, however, it also bears negative side effects. Up to date information anticipate the possibility of selecting a group of patients from stage II and III, that could be profiting from primary surgical resection of the tumor without neoadjuvant therapy.

**Materials and methods:**

The patient data in this study were obtained from hospital documentation of 3rd Surgical Clinic of Comenius University in Bratislava from September 2013 to April 2018. In this prospective study the significance of magnetic resonance imaging (MRI) in the staging of middle and low rectal cancer as well as in indication of neoadjuvant therapy was analyzed. This study included tumors ranging from T1 to T3b according to TNM staging with free circumferential resection margin (CRM), distance from mesorectal fascia more than 5 mm as well as free intersphincteric plane, while the N stage was not considered. A p value of < 0,04 was considered to represent statistical significance. The medical records of these patients were analyzed after approval of the institutional review board.

**Results:**

This study includes 96 patients with primary tumor resection. Group of patients with low rectal tumor (0-6 cm from anus) comprises 32 patients (33 %), and group of patients with mid rectal tumor (6-12 cm) consists of 64 patients (67%). When comparing the accuracy of preoperative MRI and definitive histopathological examination the overall success rate was 73 % with overestimation in 16 % and underestimation in 11 % of cases. The final results show overstaging in T-staging using MRI examination in 19 % of patients with mid rectal cancer (12 patients) and in 13 % with lower rectal cancer (4 patients). Understaging of cancer was seen in 13 % of patients with low rectal cancer (4 patients), 9 % of mid rectal cancer (6 patients). The analysis yields statistically significant difference in T-staging of low and mid rectal tumors (p=0,04). N-staging in preoperative diagnosis is currently one of the most discussed topics. Through MRI examination it is possible to estimate the increase in lymph node size, however, it is not possible to specify if the enlargement is a result of metastases or reactive changes. From 96 patients with invasive carcinoma the preoperative MRI examination and postoperative histological evaluation of lymph nodes showed conformity in 38 cases (40 %). Most of the time MRI lead to overstaging, which was observed in 49 cases (51 %), with complete negativity of lymph nodes proven in histological examination in 38 cases. On the other hand, understaging of lymph nodes was observed only in 9 cases (9 %). The results show statistically significant differences in N- staging (p=0,003).

**Conclusion:**

The accurate assessment of T-staging of tumor, CRM and distance from mesorectal fascia are key factors in correct patient management. Magnetic resonance plays an important role in T-staging of rectal tumors, however, there are admittedly issues in N-staging of tumors which should lead to reevaluation of neoadjuvant therapy. We see the future of preoperative staging in accurate T-staging together with assessing the circumferential resection margin and distance of tumor from mesorectal fascia. The result of our analysis supported by numerous other international studies shows the importance of revising the current guidelines.

## DGAV: Varia

### A Systematic Review and Meta-analysis of Physical Exercise Prehabilitation in Major Abdominal Surgery

(Abstract ID: 186)

P. Heger^1^

^1^*Universitätsklinikum Heidelberg*

**Background:**

Physical exercise prehabilitation is a proposed strategy to improve postoperative outcomes in patients undergoing major abdominal surgery. The aim of this systematic review was to investigate the effect of preoperative exercise training compared to standard care in major abdominal surgery.

**Materials and methods:**

Randomized controlled trials comparing prehabilitation with standard care were identified by a systematic literature search of MEDLINE and CENTRAL. Qualitative and quantitative analyses of the perioperative outcomes were conducted and meta-analyses perfomed.

**Results:**

A total of eight trials including 442 patients met the inclusion criteria. These trials investigated the effect of prehabilitation in liver, colorectal, gastroesophageal and major abdominal surgery in general. Quantitative analyses of all included trials showed a significant reduction of postoperative pulmonary complications (OR 0.37; 0.20 to 0.67; p=0.001) as well as postoperative overall morbidity (OR 0.52; 0.30 to 0.88; p=0.01) in the prehabilitation group compared to standard care. The length of hospital stay showed no significant differences between the groups (MD -0.58; -1.28 to 0.13; p=0.11). The risk of bias differed between the trials and three of the included trials showed moderate to high risk of bias.

**Conclusion:**

Prehabilitation including a physical exercise intervention can lead to a reduction of postoperative pulmonary complications as well as overall morbidity compared to standard care patients in major abdominal surgery. Further well-designed RCTs are needed for evaluation of the positive effects of physical exercise prehabilitation.

### Intrauterine negative pressure therapy (IU-NPT): First description of a new therapeutic approach in a case of peritonitis after caesarean section

(Abstract ID: 253)

C.-H. Wulfert^1^, A. Abdel-Kawi^1^, S. Borstelmann^1^, W. Schulze^1^, T. Schorsch^1^, H. Schmidt-Seithe^1^, G. Loske^1^

^1^*Katholisches Marienkrankenhaus Hamburg gGmbH*

**Background:**

We describe a novel intra-uterine negative pressure therapy (IU-NPT) in a patient with peritonitis due to a uterine near-rupture after re-re-re-caesarean section. In 4-quadrant peritonitis a laparotomy, lavage and intra-abdominal NPT with an open-pore drainage film (Suprasorb^®^ CNP drainage film, Lohmann & Rauscher, Germany) was performed. The planned relaparotomy showed a defect on the anterior uterine wall, which was excised and sutured. In the subsequent second relaparotomy the defect sutures were dehiscent again. For the local treatment of the septic uterine focus we initiated an IU-NPT for the first time.

**Materials and methods:**

For IU-NPT two types of open-pore film drainages (OFD) were used. For a first period a "lost OFD" (xOFD) was constructed by cutting off the distal end (20 cm length) of a 18 Charrière gastric tube. First, the proximal end of this piece of drainage was wrapped with a piece of open-pore polyurethane foam. Then the drainage was completely enveloped with a thin double-layered drainage film (Suprasorb^®^ CNP). The one half of the xOFD, which was coated only with the film, had a diameter of 6 mm. This part was inserted into the cavum uteri via the gaping defect and the other half of the xOFD remained in the lower abdomen. The xOFD was in direct contact with the intra-abdominal drainage material. The abdominal cavity was set under negative pressure with an electronic vacuum pump (-75 mmHg). Via the xOFD negative pressure was directed into the intrauterine cavum.

The second and last cycle of IU-NPT was done from vaginal. Therefore the distal end of a gastric tube was wrapped with a layer of the thin drainage film to construct a vaginally-evacuated OFD (vOFD). After dilation of the obliterated cervix uteri the vOFD was inserted vaginally along the cervix into the uterine cavity. The implanted xOFD was removed surgically. The uterine defect was closed by a suture over the embedded vOFD. A negative pressure of 75 mmHg was applied to the vOFD by an electronic pump.

**Results:**

After insertion of the xOFD, the negative pressure could be transmitted to the uterine cavity by abdominal NPT. After 3 days of IU-NPT the xOFD was removed by relaparotomy. The local wound conditions were good. Transvaginally a vOFD was inserted after cervix dilatation and the uterine defect was closed with suture again. The abdominal negative pressure therapy was stopped. After 8 days, the vOFD was removed by pulling the drainage. The endoscopic examination of the uterus cavum showed collapsed cavity with erosive suction pattern and closed uterus defect. The duration of the IU-NPT was 11 days in total. Four days after the end of the IU-NPT, the patient was discharged without complaint.

**Conclusion:**

Suture failure after cesarean section with peritonitis is a rare complication. Negative pressure therapy was successfully performed in the uterine cavity for the first time in this indication. The IU-NPT is made possible by novel small-lumen open-pored film drainages. The IU-NPT is a new organ-preserving therapy for uterine defects.

**Picture:**
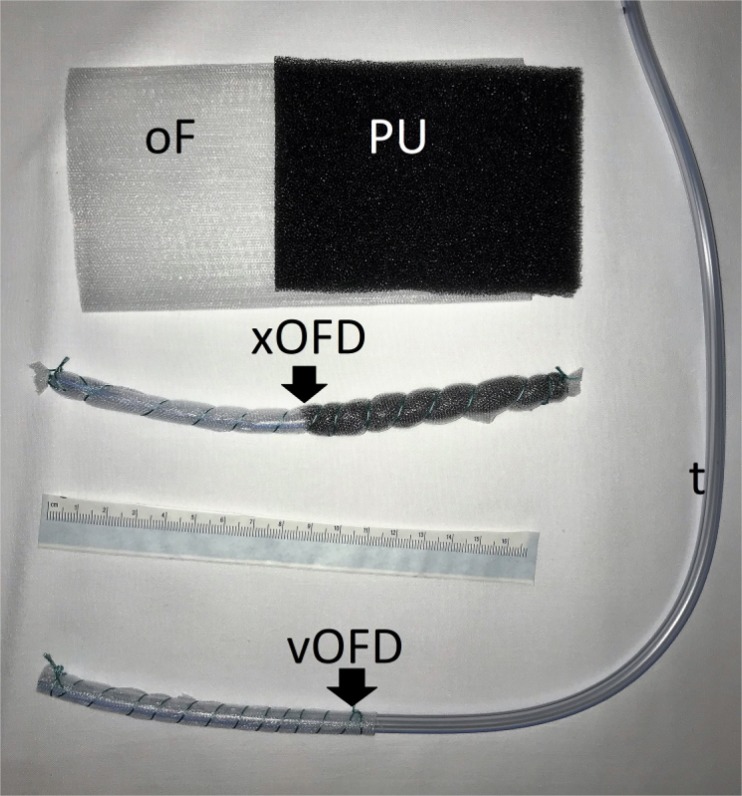
Material for IU-NPT.

### Analysis of outcomes and predictors of long-term survival following resection for retroperitoneal sarcoma

(Abstract ID: 276)

T. Malinka^1^, M. Nebrig^1^, M. Bahra^1^, A. Sick^1^, K. Hoppe^1^, J. Pratschke^1^, A. Andreou^1^

^1^*Charité - Universitätsmedizin Berlin CVK*

**Background:**

Retroperitoneal sarcomas (RPS) include a heterogeneous group of rare malignant tumors and numerous treatment algorithms have been controversially discussed until today. The aim of the present study was to examine postoperative and long-term outcomes after resection for RPS.

**Materials and methods:**

Clinicopathological data of patients who underwent resection for RPS between 2006 and 2014 were assessed and predictors for overall survival (OS) and disease-free survival (DFS) were identified.

**Results:**

Sixty patients underwent resection for RPS. Postoperative mortality and morbidity rates were 2% and 46%, respectively. After a median follow-up time of 74 months, 5-year OS and DFS rates were 58% and 34%, respectively. Distant metastasis (5-year OS: 67% vs. 0%, P = 0.001) and poor tumor differentiation (5-year OS: G1: 92% vs. G2: 54% vs. G3: 43%, P = 0.015) were significantly associated with diminished OS in univariate and multivariate analyses. When assessing DFS, vascular involvement (5-year DFS: 39 vs 33%, P = 0.044), poor tumor differentiation (5-year DFS: G1:63% vs. G2: 24% vs. G3: 22%, P = 0.013), and positive resection margins (5-year DFS: 53% vs. 10%, P = 0.014) were significantly associated with inferior DFS in univariate and multivariate analyses.

**Conclusion:**

Distant metastasis and high-grade tumors indicated poor OS, while vascular involvement, tumor grade, and resection margins are the most important predictors of DFS. Whereas multimodal treatment strategies are progressively established, surgical resection remains the mainstay in the majority of patients with RPS, even in cases with vascular involvement or multifocality.

### Requirements and real world data in German residents OR catalogues – Results of a survey among the participants of the “Warnemünde Practical Course for Visceral Surgery”

(Abstract ID: 290)

A. A. Röth^1^, C. Klinger^2^, C.-T. Germer^3^, T. Keck^4^, J.-P. Ritz^5^, B. Vollmar^6^, H.-J. Buhr^2^, M. Mille^7^

^1^*Uniklinik RWTH Aachen*

^2^*Deutsche Gesellschaft für Allgemein-, Viszeral- und Transplantationschirurgie, Berlin*

^3^*Universitätsklinikum Würzburg*

^4^*Uniklinik Lübeck*

^5^*Helios Kliniken Schwerin*

^6^*Universitätsmedizin Rostock*

^7^*Helios-Klinik Erfurt*

**Background:**

In Germany, the requirements for surgical residency and the surgical board examination are defined by the Federal Medical Association. Apart from the theoretical knowledge, this includes practical skills and a defined spectrum of procedures that have to be performed in order to be admitted to the exam. The exam itself does not test practical skills and there is no structured curriculum advising which procedures should be performed in which year of residency.

Aim of this study was to investigate how many procedures are being performed by residents at which stage of residency.

**Materials and methods:**

We performed a questionnaire based evaluation with a structured CRF that was handed out to the participants of the "Warnemünde Practical Course for Visceral Surgery" 2017 and 2018.

We included only participants with 8 or less years of experience in surgery (i.e. residents and young fellows), which resulted in 191 returned questionnaires, including 66 from smaller community hospitals (Grund-und Regelversorger), 70 from mid-sized regional hospitals (Schwerpunktversorger), 29 from non-university tertiary care hospitals (Maximalversorger) and 26 from university hospitals. 84 participants were male and 108 female. The average experience in performing surgery was 4.13 years. The survey examined the number of procedures performed in conventional and laparoscopic appendectomy, conventional and laparoscopic cholecystectomy as well as conventional and laparoscopic colonic resections.

**Results:**

The procedures (prcd.) most often performed were laparoscopic appendectomy and laparoscopic cholecystectomy (median 20 resp. 30 prcd.). Laparoscopic colonic resections were only very seldomly performed by residents and young fellows (149 participants with 0 prcd., 23 participants with 1 or 2 prcd.). Most of the procedures were performed less often in the university hospitals (e.g. 20 appendectomies vs. 28.5 in community hospitals, 30 in mid-sized regional and 30 in non-university tertiary care hospitals). When investigating at which state of residency the procedures were performed, we found that often there is a gap between the 6th year of residency and the first years as a fellow when the number of surgeries increases significantly. This is especially obvious in general hospitals. Although 10 colon procedures are required for the German board examination, the Q3 interval for colonic resections was below this number in all hospital levels. Young fellows as well did not reach this number except for the fellows working in major regional hospitals. When comparing male and female residents, there is also a trend that female residents seem to perform fewer procedures. This is especially the case when comparing the number of laparoscopic cholecystectomies in university hospitals (median 30 resp. 15 prcd.).

**Conclusion:**

The number of procedures that are being performed during residency in Germany is rather low and differs significantly from the requirements set by the Federal Medical Association. Structured curricula with defined operations throughout several periods of the residency might help to address this dilemma.

### Oxidative stress-derived cytosolic dsDNA results in AIM-2 inflammasome-derived IL-1β release and induces postoperative ileus

(Abstract ID: 296)

K. J. Hupa^1^, R. Schneider^1^, K. Stein^1^, M. Lysson^1^, B. Schneiker^1^, V. Hornung^2^, J. C. Kalff^1^, S. Wehner^1^

^1^*Universitätsklinikum Bonn*

^2^*Klinikum der LMU München - Campus Großhadern, München*

**Background:**

Abdominal surgery commonly leads to a transient postoperative intestinal dysmotility, known as postoperative ileus (POI). We have previously shown that enteric glial cells respond to IL-1 receptor type I signaling, indicating a pivotal role of this mechanism in POI development. Herein, we studied the role and of the two IL-1 receptor agonists IL-1α and IL-1β in the POI development, describe their cellular origin and identified the underlying molecular mechanism.

**Materials and methods:**

C57BL6 wildtype, IL-1α-/- and IL1β -/-, ASC-/-, Caspase-1-/-, Nlrp3-/-, Nlrc4-/- and AIM2-/- mice underwent a standardized intestinal manipulation (IM). In some experiments chimeric mice were generated by bone marrow transplantation between wildtype and IL-1α or IL1β knockout mouse strains before IM. Three and 24h after IM, cytokine expression, leukocyte infiltration and gastrointestinal transit (GIT) were assessed. Myenteric plexus cell cultures were analyzed for IL-1 mediated cell activation.

**Results:**

In wildtype animals, IM led to a significant induction of chemokine expression and leukocyte influx into the muscularis externa compared to sham-operated controls. GIT was also significantly decelerated by IM. IL1α and IL1β deficient mice demonstrated a reduced postoperative leukocyte infiltration and an accelerated GIT. Mice deficient for IL-1α in radio-resistant cells but not in hematopoietic radio-sensitive cells were protected from POI while IL-1β deficiency in hematopoietic cells but not resident cells protected mice from POI. A proinflammatory gene expression profile identified IL-1α as a danger-associated molecular pattern (DAMP) molecule, acting already in the early phase of POI. In contrast, IL-1β derived from infiltrating monocytes in the late phase POI and strictly depended on Caspase-1 and ASC and was abrogated in AIM-2-/- but not Nlrp3-/- or Nlrp4-/- mice. The AIM-2 inflammasome was activated by cytosolic dsDNA and the microbiome as well as oxidative stress were identified as major triggers.

**Conclusion:**

Our results show that IL-1β signaling in POI induces a biphasic response with IL-1α acting as a DAMP in the early phase of POI. IL-1β originated from monocytes in the late phase POI and depends on AIM-2 inflammasome activation which is triggered by the microbiome and endogenously by oxidative stress. Interaction in these pathways may be a promising strategy to prevent POI in humans.

### Cellularity in low-grade Pseudomyxoma peritonei impacts recurrence-free survival following cytoreductive surgery and hyperthermic intraperitoneal chemotherapy

(Abstract ID: 771)

P. Horvath^1^, C. Yurttas^1^, A. Königsrainer^1^

^1^*Universitätsklinikum Tübingen*

**Background:**

Documentation of cellularity in Pseudomyxoma peritonei (PMP) is not performed on a regular basis in everyday clinical practice, but is recommended by the PSOGI (Peritoneal Surface Oncology Group International). We investigated the impact of cellularity in PMP following cytoreductive surgery (CRS) and hyperthermic intraperitoneal chemotherapy (HIPEC) on recurrence-free survival.

**Materials and methods:**

Data from 25 patients with low-grade (American Joint Committee on Cancer grade G1) PMP were retrospectively evaluated. Cellularity was categorized as acellular mucin, scant (<2% cellularity), moderate (2%-19% cellularity) or high cellularity (>20% cellularity). Impact of cellularity, PCI, CC-score and HIPEC regimen on recurrence-free and overall survival was primarily assessed.

**Results:**

Assessment of cellularity showed acellular mucin in ten patients (40%), scant cellularity in 11 (44%) patients, moderate cellularity in one (4%) patient and high cellularity in three (12%) patients. Median PCI was 15 (range, 1-39). A CC-0 score was achieved in 13 (52%) patients and a CC-1 score was achieved in 12 (48%) patients. After a median follow-up of 25 (range, 2-74) months all patients were still alive. Overall, four (16%) patients suffered from recurrent disease after a median of 38 (range, 36-60) months. PCI above 17 (p=0.03) and moderate and high cellularity (p=0.007) were statistically significantly associated with recurrent disease. CC-score and HIPEC compound used did not impact on recurrence-free survival.

**Conclusion:**

Recurrent disease occurs more often in patients with PCI values above 17 and with moderate and high cellularity in low-grade PMP. Pathological assessment of cellularity is crucial for identification of patients at risk for recurrence.

### Development of a protocol for long-term functional maintenance of exteriorized portal venous catheter after major liver resection in a porcine large animal model

(Abstract ID: 820)

E.-M. Wittauer^1^, F. Oldhafer^1^, O. Beetz^1^, C. Schumacher^1^, K. Johanning^1^, A. Bleich^1^, F. W. R. Vondran^1^

^1^*Medizinische Hochschule Hannover*

**Background:**

Development of portal venous pressure is of great importance for clinical outcome after liver resection and liver transplantation. Furthermore, portal venous access might be of interest for functional analyses of the liver. Thus, a permanent portal venous catheter represents an interesting tool to study liver regeneration and metabolism in a large animal model since perioperative pressure monitoring (e.g. after major liver resection) and long-term access for blood withdrawl are enabled. Unfortunately, functionality of implanted catheters often is impaired by early thrombosis or fibrin capping. Aim of this study thus was to establish a safe anticoagulation protocol for long-term use of portal venous catheters with low risk of bleeding in pigs.

**Materials and methods:**

In 22 adult LEWE Minipigs (423± 74 days old; weight of 43.0± 7. 7kg) a central venous and a portal venous catheter were implanted into dextra internal jugular and portal vein, respectively. Both catheter hubs were tunneled subcutaneously in direction to the scapula. All Minipigs received left trisectionectomy, in four cases also partial right lateral sectionectomy was performed. We tested three different protocols for long-term functional maintenance of the portal venous catheter. Group1 (n=5) received saline solution i.v. with a flow rate of 2ml/h, group 2 (n=7) received saline solution i.v. with a flow rate of 10ml/h and enoxaparin injection s.c. (anti-Xa-level of 0.3 - 0.8) twice daily and group 3 (n=10) received saline solution with 2 I.E. Heparin/ml i.v. with a flow rate of 2ml/h from postoperative day (POD) 3. Solutions were administered with mini infusion pumps via portal venous catheter.

**Results:**

Mean resected mass of liver parenchyma per animal was 300.2 ±46.8 g. This represents a resection of 51.7% and was comparable among all groups. All central venous catheters remained in situ fully functional until the end of experiment. One externalized portal venous catheter accidentally got lost due to getting caught on the grid of the box on postoperative day 12. Median functional maintenance of portal venous catheters in groups 1 and 2 were 7 and 5 days, respectively. In group 3 all portal venous catheters were fully functional until median 28. 5 days (end of experiment; range of 27 - 30 days). There were no bleeding complications. In groups 1 and 2 thrombotic material was found within the lumen of the portal venous catheters and an extraluminal thrombus of 2cm length around catheter tip was seen in 2 cases. Similar findings could not be observed in group 3.

**Conclusion:**

Continuous portal venous infusion of low-dose heparin-saline solution early after major liver resection seems to be a safe and successful anticoagulation protocol for long-term functional maintenance of exteriorized portal venous catheters. In addition, it is a stress- and painless therapy in comparison to subcutaneous injections, which seems advantageous in a chronic large animal model.

### Surgical Procedure Manager – surgical work flow optimization and procedure standardization with the help of a software tool?

(Abstract ID: 921)

L. Timmermann^1^, R. Öllinger^1^, M. Schmelzle^1^, M. Bahra^1^, J. Pratschke^1^

^1^*Charité - Universitätsmedizin Berlin CVK*

**Background:**

Surgical Procedure Manager (SPM) is a software tool for standardization, work flow optimization and surgical training using a precast precept defining particular steps of surgical procedures mostly eligible for procedures with a low variability of sequence and execution of certain steps and used instruments. It includes a list and visualization of required instruments, intended surgical steps as well as a time module starting with the positioning of a patient on the table. SPM thereby is considered not only to decrease time needed for the procedure itself but also transfer times and anesthesia.

**Materials and methods:**

SPM (Surgical Process Institute Deutschland GmbH, Leipzig) was implemented for several surgical procedures, currently including laparoscopic cholecystetomy, PPPD, and kidney transplantation. Experienced surgeons defined certain steps of each procedure and the desired time and instruments needed for each step. Both, experienced surgeons as well as trainees performed the procedures using SPM: each predefined step is announced by the system vocally to optimize the work flow of surgeons in training and assisting personnel. The information serves the OR administrators and anesthesiologist for planning following cases in this particular OR. Recording begins with positioning the patient on the table and establishing sterile conditions. Each finished step during surgery is then testified by the leading surgeon via pressing a foot pedal. Continuous comparison of actual and desired time frames for certain steps allows constant feedback and an accurate forecast of the expected end of the procedure.

**Results:**

The SPM can easily be used by every surgeon performing one of the standardized procedures. It has been used for 33 procedures in the time between April and August 2018 with an evaluable amount of 45%. SPM was started and not sufficiently finished in the other 55%. The using rate increased after a first feedback round in May to a peak level of 33% for the average of all included procedures in July. The laparoscopic cholecystectomy and the PPPD reached a using rate of 50% each. Due to simultaneously performed procedures the ultimate using rate for July was calculated with 75% using only one tower. Particularly surgeons in training and the assisting personnel as well as students and nurses subjectively report about advantages in the usage of SPM especially for determination of procedure key and endpoints and thereby the workflow during the certain procedure as well as coordinating anesthesia and procedures to follow.

**Conclusion:**

SPM is easy to use and our first experiences are promising: transparency of real-time information serves work flow optimization. Both, the team within the OR as well as external coordinators were able to improve process parameters within an OR of a large University hospital. Nevertheless, SPM usage is subject to a learning curve. Furthermore, only procedures with a low variability of sequence, techniques and necessary instruments, such as the laparoscopic cholecystectomy, appeared to be feasible.

## DGAV: Health Services Research

### Colorectal Cancer Centers in Germany: Does certification yield to a survival benefit?

(Abstract ID: 35)

V. Völkel^1^, T. Draeger^2^, M. Gerken^2^, A. Fürst^1^, M. Klinkhammer-Schalke^2^

^1^*Caritas-Krankenhaus St. Josef, Regensburg*

^2^*Universität Regensburg*

**Background:**

Hospitals treating colorectal tumors can obtain a certification by the DKG if they meet high quality-standards. It is yet unclear if surgery at such Colorectal Cancer Centers leads to a survival benefit.

**Materials and methods:**

Data for this retrospective cohort study derives from an official German cancer-registry, which collects medical and demographical information of all tumor patients registered within a political district of 1.1 million inhabitants. To compare 3-year-survival rates of center- and non-center-cases 2312 patients who had their colorectal adenocarcinoma removed between 2010 and 2013 were included in Kaplan- Meier- and multivariate Cox-regression-analysis. Additionally, center cases were checked against 1690 cases before 2008 from the same hospitals using relative survival methods to control for temporal changes in life expectancy.

**Results:**

Comparing crude survival rates center-cases show a significant survival benefit over patients from other hospitals (3-year-overall-survival-rate: 71.6% vs. 63.6%, p= 0.001). This superiority can still be observed after adjusting for relevant confounders like age or localization (reference: non-center-cases, HR= 0.81, CI: 0.67 - 0.98). In contrast to this, there is no statistically relevant difference between 3-year-relative survival rates of center-cases (78,1%) and pre-certification-patients (78.2%, p= 0.33). Similar results are obtained computing adjusted excess-hazard-ratios (reference: pre-certification-patients, EHR= 1.05, CI: 0.94 - 1.18).

**Conclusion:**

Treatment at Colorectal Cancer Centers is associated with superior survival. However, the receipt of a certification did not alter patients’ survival rates visibly, presumably because the later centers began to implement the DKG’s high standards even before 2004.

**Picture:**
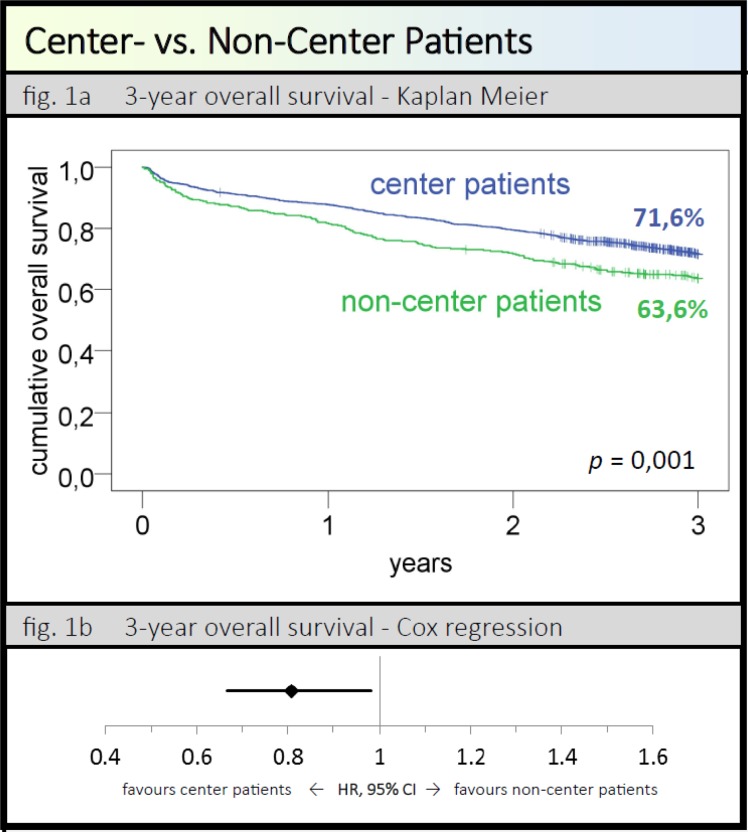
3-year-overall-survival center vs. non-center patients

### Minimal case load requirements and centralisation of esophageal and pancreatic resections in Germany: Status Quo in 2015 and outlook

(Abstract ID: 618)

R. Hummel^1^, M. Thomaschewski^1^, O. Kopeleva^1^, T. Keck^1^, C. Krauss^2^, C. Schmidt^3^

^1^*Uniklinik Lübeck*

^2^*CÆSTLEY Software, Neuwittenbek*

^3^*Universitätsmedizin Rostock*

**Background:**

Based on the international literature, there is no doubt that centralisation of complex high risk procedures such as esophageal and pancreatic resections in high volume centres results in better outcome of patients. In Germany, minimal case load requirements are defined since 2004, but implementation into clinical practice seems insufficient. The current study aimed to analyse the Status Quo in 2015 and provides an outlook into a potential future care scenario.

**Materials and methods:**

Based on the minimal case load requirements as given by the Federal Joint Committee (Gemeinsamer Budnesausschuss G-BA), DRG-data sets for the year 2015 (DRG-Statistik) were requested from the German Federal Statistical Office (Deutsches Statistisches Bundesamt, DESTATIS). Levels of centralisation of esophageal and pancreatic resections in Germany in 2015 were assessed on federal (Bund) and local (Bundesländer) levels. Based on these data, potential scenarios of future medical care were developed and discussed.

**Results:**

The current analysis showed that - from the viewpoint of patients - centralisation of esophageal and pancreatic resections in Germany seems fairly advanced with 92% respectively 79% of all pancreatic respectively esophageal resections being performed in centres that fulfil current minimal case load requirements and perform more than 10 cases per year. However, only 55% (pancreatic resections) respectively 36% (esophageal resections) of hospitals actually met minimal case load requirements in 2015 what implicates that a high number of hospitals (esophageal resections:265 hospitals; pancreatic resection: 290hospitals) perform a very low number (less than 10) of resections per year. On the other hand, an immediate and complete implementation of the minimal case load requirements of 10 resections per year as given by the Federal Joint Committee would mandate re-allocation of 870 esophageal and 945 pancreatic resections from hospitals that do not fulfil minimal case load requirements to those who do. Postulating an even distribution of these procedures to the remaining hospitals, the re-allocation could increase numbers of resections in single hospitals by around 6-22% for pancreatic, and by around 13-48% for esophageal resections.

**Conclusion:**

The current study demonstrated that centralisation of esophageal and pancreatic resections in Germany is already fairly good from the viewpoint of patients. However, from the hospitals view, there is much more work to be done, and re-allocation of procedures need thorough planning.

### Meta-analysis of postsurgical exercise training for survival in colorectal cancer surgery

(Abstract ID: 685)

M. Haensig^1^, P. Simon^2^, D. Pfirrmann^2^, J. Stütz^2^, B. Hillen^2^, I. Gockel^1^

^1^*Clinic and Policlinic of Visceral-, Transplant-, Thoracic- and Vascular Surgery, Leipzig*

^2^*Department of Sports Medicine, Rehabilitation and Disease Prevention, Mainz*

**Background:**

Several studies suggest that exercise training reduces the risk of colorectal cancer (CRC) development, however, limited evidence is available on the role of postsurgical exercise training in CRC surgery patients.

**Materials and methods:**

We performed a systematic data search in PubMed and Cochrane Library databases for relevant articles before Jun 2018. We adopted adjusted estimates to calculate pooled hazard risks (HRs) with 95% confidence intervals (CI) by the random-effects model.

**Results:**

Our meta-analysis included 9 studies (n=10694 pts.) with a median follow-up of 5.5 years (IQR 5.0-11.6 yrs). Pooled adjusted hazard ratio of colorectal cancer-specific mortality reduction by postsurgical exercise training was 0.51 (95% CI, 0.33 to 0.89). Mortality was reduced from 14.7% to 5.3% in women and from 14.8% to 7.9% in men. The effect seems to be more pronounced in women. Also patients with a higher BMI or positive 1st degree family history benefit from postsurgical training.

**Conclusion:**

Postsurgical exercise training seems to be more effective than prior to colorectal cancer surgery. There was no significant difference between high- and low-intensive exercise training. However, patients prone to a relapse or death within 6 months after study inclusion, were excluded and therefore the results might be alleviated (intention-to-treat).

### Validating the global surgery accessibility indicator: Comparing geographical modeling to patient reported travel times to a rural district hospital in Eastern Rwanda

(Abstract ID: 712)

M. Gruendl^1^

^1^*Harvard University / Universitaet Freiburg, Poesing*

**Background:**

Surgical conditions account for 30% of the global burden of disease, yet 5 billion people don’t have access to safe, affordable and timely surgical and anaesthesia care. In 2015, The Lancet Commission on Global Surgery (LCoGS) recommended 6 key indicators to evaluate the strength of a country’s surgical system. The set of core surgical indicators measures provider density, operative volume, surgical safety, financial effects and timely access. The last indicator is defined as the percentage of the population who can access, within 2 hours, a facility capable of performing all three of the bellwether procedures, defined as a caesarean section, laparotomy and open fracture repair. Long travel times for women to reach maternal services are a known predictor of poor maternal outcomes, particularly for cesarean deliveries. The most accurate method for assessing two-hour access in low- and middle-income countries has not yet been well described in the literature. In high income countries, Geographical Information System (GIS), a system of capturing, storing, checking, integrating, manipulating, analyzing and displaying data which are spatially referenced to the earth has been popular. While GIS may accurately estimate patient travel times in high income countries there is little validation of this approach in sub-Saharan Africa.The aim of our study was to compare the accuracy of GIS estimates to patient-reported travel times for patients travelling to a district hospital in rural Rwanda for emergency obstetric care.

**Materials and methods:**

Our study includes all women, >18 years, undergoing a cesarean delivery from June 2017 to February 2018 at Kirehe District Hospital. Data collectors interviewed patients prior to discharge to collect baseline demographic and economic data. Data was collected using REDCap, a secure, web-based application designed to support data capture for research studies in areas with low connectivity, using Android tablets.In our analyses, we compared patient reported and GIS estimated travel times. For patient reported travel time, we only included time in transit (time from home to health center and health center to hospital) and did not include patient waiting times at the health center and hospital. We believe this is the most comparable to the GIS estimated travel times which would also not include any delays in the estimates. We excluded three patients from analyses because their data were outliers deemed to be caused by data entry errors.

**Results:**

A total of 664 patients were included. The majority of patients used multiple modes of transportation (walking = 47.9%, public = 74.2%, private transportation = 2.9% and ambulance 71.1%). The total transport time reported by patients, not including waiting at the health center, was longer than the time estimated by the standard GIS model, with a regression coefficient of 1.49 [95% CI: 1.40 - 1.57], i.e. GIS estimates could be multiplied by 1.49 to yield a better approximation of patient reported travel times.When the GIS model was modified to take journeying via the assigned health center into account, GIS estimates were much closer to travel times reported by patients [b = 1.12; 95% CI: 1.05 - 1.18]

**Table:** 

Variable	n (%)
n	664
Age (median [IQR])	26.00 [23.00, 31.00]
Education level	
No education	59 (8.9)
Primary education	470 (70.8)
Secondary or higher education	135 (20.3)
Household monthly income	
0 - 10,000 Rwf	518 (78.0)
10,000 - 20,000 Rwf	69 (10.4)
20,000 - 30,000 Rwf	26 (3.9)
>30,000 Rwf	51 (7.7)
Modes of transportation used from home to health center*	
Walking	183 (27.6)
Public	477 (71.8)
Private	12 (1.8)
Ambulance	8 (1.2)
Modes of transportation used from health center to hospital*	
Walking	162 (24.4)
Public	36 (5.4)
Private	9 (1.4)
Ambulance	467 (70.3)

**Conclusion:**

Typical approaches to estimate patient travel time significantly underestimate patient-reported travel times. Modifying GIS models to take travel via a health center into account, the most common pathway for patients, would likely yield more accurate estimates.

**Picture:**
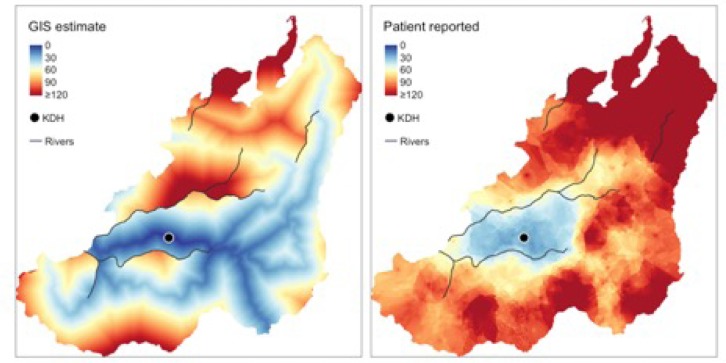
Map comparison of patient reported and GIS estimated travel times.

### Prognostic relevance of neoadjuvant radiochemotherapy in N rectal cancer with limited tumor infiltration of the wall < 12 cm above the anocutaneous line (ACL)

(Abstract ID: 718)

H. Ptok^1^, D. Jacob^1^, F. Meyer^1^, H. Lippert^1^, I. Gastinger^1^

^1^*Universitätsklinikum Magdeburg*

**Background:**

There are no data on the prognostic improvement by neoadjuvant (neoadj.) radiochemotherapy (RCTx) in rectal cancer (Ca) with positive nodal status (cN ).

**Materials and methods:**

From the data obtained in the multicenter observational study on the treatment of rectal Ca (Institute of Quality Assurance in operative Medicine, Otto-von-Guericke University with University Hospital, Magdeburg-Germany), in particular, patients with the intention of a radical resection of a cT2 rectal cancer (< 12 cm above ACL) and no adjuvant RCTx but also no distant metastases were analyzed. Patients were subdivided into 4 groups (Gr.): Gr. 1 [n=1,112]: cT2, pN0, no neoadj. RCTx; Gr. 2 [n=352]: cT2, pN , no neoadj. RCTx; Gr. 3 [n=231]: cT2, ypN0, neoadj. RCTx; Gr. 4 [n=61]: cT2, ypN , neoadj. RCTx). 5-year(yr)-local recurrence rate (LRR) and 5-yr-distant metastases rate (DMR) were determined and compared among groups.

**Results:**

Patients of Gr. 1 & 2 were significantly (signif.) older (p<0.001), had more cardial risk factors (p=0.011) and their tumor lesions were higher above ACL than in patients of Gr. 3 & 4 (p<0.001). There were no further differences with regard to pretherapeutic patient- or tumor-associated parameters comparing the groups. Histopathological findings revealed a signif. larger diameter of the tumor lesion in patients of Gr. 1 & 2 (p<0.001) and a signif. greater number of investigated lymph nodes (p<0.001). Comparing the groups, there were no signif. differences of TME quality (p=0.444) and portion of pCRM( ) resections (p=0.572).

After a median follow-up of 54 months, (y)pN patients showed a signif. higher 5-yr-LRR (Gr. 2: 7.3 %; Gr. 4: 7.2 %) compared to (y)pN0 patients (Gr. 1: 3.7 %; Gr. 3: 2.8 %). Patients of Gr. 3 had the lowest 5-yr-DMR (8.9 %) - Patients of Gr. 1: 17.2 % (Gr. 1 vs. Gr. 3: p=0.048). The highest rate was found in patients of Gr. 2 (25.8 %; Gr. 2 vs. Gr. 1: p=0.001) & Gr. 4 (24.4 %; Gr. 4 vs. Gr. 1: p=0.069).

**Conclusion:**

The data seem to indicate that neodj. RCTx may provide - in addition to the local effect - also a systemic effect by the upfront treatment such as neoadj. RCTx (in particular, with regard to limited metastatic tumor growths), which should be subject of further analyses.

